# Total Synthesis of Ganoapplanin Enabled by a Radical Addition/Aldol Reaction Cascade

**DOI:** 10.1021/jacs.4c08291

**Published:** 2024-08-07

**Authors:** Nicolas Müller, Ondřej Kováč, Alexander Rode, Daniel Atzl, Thomas Magauer

**Affiliations:** Department of Organic Chemistry and Center for Molecular Biosciences, University of Innsbruck, 6020 Innsbruck, Austria; Department of Organic Chemistry and Center for Molecular Biosciences, University of Innsbruck, 6020 Innsbruck, Austria; Department of Organic Chemistry, Palacký University Olomouc, 77900 Olomouc, Czech Republic; Department of Organic Chemistry and Center for Molecular Biosciences, University of Innsbruck, 6020 Innsbruck, Austria

## Abstract

The total synthesis of the *Ganoderma* meroterpenoid ganoapplanin, an inhibitor of T-type voltage-gated calcium channels, is reported. Our synthetic approach is based on the convergent coupling of a readily available aromatic polyketide scaffold with a bicyclic terpenoid fragment. The three contiguous stereocenters of the terpenoid fragment, two of which are quaternary, were constructed by a diastereoselective, titanium-mediated iodolactonization. For the fusion of the two fragments and to simultaneously install the crucial biaryl bond, we devised a highly effective two-component coupling strategy. This event involves an intramolecular 6-*exo*-trig radical addition of a quinone monoacetal followed by an intermolecular aldol reaction. A strategic late-stage oxidation sequence allowed the selective installation of the remaining oxygen functionalities and the introduction of the characteristic spiro bisacetal structure of ganoapplanin.

*Ganoderma* meroterpenoids (GMs) are a class of structurally diverse natural products, comprising more than hundred members, isolated from fungi of the genus *Ganoderma*.^1^ Structurally, these secondary metabolites share a common hydroquinone motif, connected to a farnesyl- or geranyl-derived terpenoid scaffold. According to the known structural modifications, three subclasses have been identified: linear, polycyclic, and dimeric GMs (Figure 1). Linear GMs consist of a hydroquinone connected to either a C10 (e.g., lucidulactone B (**1**)^2^) or a C15 terpenoid chain (e.g., ganomycin A (**2**)^3^). Conversely, cochlearol B (**3**),^4^ applanatumol B (**4**),^5^ and ganodermaone A (**5**)^6^ contain a hydroquinone motif, connected to a polycyclic ring system. The subclass of dimeric GMs, as exemplified by applanatumin A (**6**)^7^ and ganoapplanin (**7**),^8^ consists of two hydroquinone units attached to a terpenoid backbone, further increasing the level of structural complexity. Several GMs exhibit notable bioactivity profiles including antioxidant, antifibrotic, and antimicrobial activities.^1^

Altogether, these features render GMs appealing targets for total synthesis.^9,10^ While polycyclic GMs, such as cochlearol B (**3**)^11–14^ and applanatumol B (**4**),^15,16^ have been synthesized numerous times, there are only few reports on the synthesis of dimeric GMs.^17,18^ Notably, the synthesis of ganoapplanin (**7**) has not been achieved so far.

Ganoapplanin (**7**), isolated by Qiu in racemic form from the fungus *Ganoderma applanatum* in 2016, stands out from a structural perspective.^8^ It features five contiguous stereo-centers, two of which are quaternary, and an unprecedented spiro bisacetal skeleton, constructed from a 6/6/6/6 tetracyclic system, including a tetra-*ortho* substituted biaryl motif (highlighted in blue) and a dioxatricyclo[4.3.3.0]dodecane scaffold.

In addition to its structural complexity, racemic **7** inhibits T-type voltage-gated calcium channels (IC_50_ = 36.6 *μ*M), showing potential as a lead compound for the development of novel therapeutics against neurodegenerative diseases (e.g., epilepsy and Parkinson’s disease).^19,20^

In recent years, our group has developed synthetic methods to access polysubstituted arenes^21^ and heteroarenes^22^ to facilitate the total syntheses of natural products structurally related to the GMs.^23,24^ However, the distinctive substitution pattern of ganoapplanin (**7**), particularly the highly congested central region, connecting the terpenoid scaffold with the tetra-*ortho* substituted biaryl motif, proved incompatible with those protocols. We therefore drew inspiration from the proposed biosynthesis of **7** (Scheme 1A),^8^ which involves the nucleophilic addition of phenol **8** to lingzhilactone (**9**) followed by bisacetalization and diazotation to yield **10**. A subsequent intramolecular Gomberg−Bachmann cyclization (Pschorr cyclization) via **I** and **II** then forges the C3−C3a biaryl bond. Suspecting that the bisacetal scaffold of **10** would be unstable and difficult to access by synthetic methods, we evaluated alternative strategies that would allow its late-stage installation. Finally, the proposed aryl radical **I** guided us toward a convergent strategy involving a radical addition/aldol reaction cascade. Herein, we present the realization of this strategy, resulting in the first total synthesis of ganoapplanin (**7**).

In our retrosynthesis (Scheme 1B), we decided to install both the lactone moiety of ganoapplanin (**7**) and the C4a oxygen functionality of chromene **11**, required for spiro bisacetal formation, via late-stage oxidation (vide infra).^25^ Further simplification of **12** provided dearomatized acetal **13**, which contains the retron for an intramolecular 1,4-addition/aldol reaction between quinone monoacetal **14** and aldehyde **15**. This strategy was inspired by the seminal work of Utimoto,^26^ a related recent report by Inoue^27^ and Li,^28^ which was envisioned to install the crucial C3−C3a biaryl and C1−C2 bonds in a single operation. Aldehyde **15** was envisioned to be accessible via a titanium(IV)-mediated iodolactonization of alkene **16**,^29,30^ which can be dissected to aldehyde **17** vinyl iodide **18**.

The southern fragment **15**^31^ was synthesized in five steps as shown in Scheme 2A. The sequence started with a one-pot Nozaki−Hiyama−Kishi (NHK) reaction between aldehyde **17**^32,33^ and vinyl iodide **18**^34,35^ to form the corresponding secondary alcohol, which was protected *in situ* to give TBS ether **16**. Treatment of **16** with Ti(O*t*-Bu)_4_, CuO and excess iodine, conditions previously reported by Taguchi,^29,30^ furnished bicyclic lactone **19** as a single diastereomer in up to 61% yield on decagram scale. The excellent diastereose-lectivity has previously been attributed to a chairlike transition state and stereoelectronic effects favoring an orthogonal alignment of the alkene and the allylic substituent (OTBS) for the 5-*exo*-trig cyclization of **lll** to **lV**. Conversion to the aldehyde was achieved by removing the methylester by employing Krapcho conditions (LiCl, H_2_O, DMSO, 150 °C), followed by allylation of the lactone and subsequent ozonolysis to yield aldehyde **15**.

Having achieved the synthesis of aldehyde **15**, we turned our attention to the construction of the aromatic fragment **14** (Scheme 2B). For this purpose, phenol **22**^36,37^ was initially protected as its methoxymethyl (MOM) ether with concomitant ester formation. The ester group was reduced by treatment with DIBAL-H to afford benzylic alcohol **23**. Exposure of **23** to a one-pot mesylation/bromination protocol yielded benzyl bromide **24**, which was then subsequently treated with excess 1,4-hydroquinone in the presence of potassium carbonate to furnish benzyl ether **25**.

Eventually, oxidative dearomatization using phenyliodine-(III)diacetate (PIDA) gave quinone monoacetal **14** in 82% yield.^38^ With access to the aromatic and terpenoid fragments **14** and **15**, the stage was set for their fusion. Initially, we investigated the intramolecular 1,4-addition of **14**. We found that metalation of **14** via metal/halogen exchange employing either *t*-BuLi or Turbo Grignard (*i*-PrMgCl•LiCl) only led to decomposition. However, treating **14** with azobis- (isobutyronitrile) (AIBN) and tributyltin hydride (*n*-Bu_3_SnH) at elevated temperatures successfully initiated the intended 1,4-addition. Unfortunately, efforts to achieve the subsequent aldol reaction by deprotonation with either LDA, KHMDS, or LHMDS at −78 °C followed by the addition of aldehyde **15** failed to yield the desired aldol-product (see the Supporting Information for details). We then focused on a one-pot procedure to realize the radical 1,4-addition/aldol reaction cascade. Eventually, we discovered that radical initiation with triethyl borane and oxygen^39^ in the presence of tributyltin hydride induced the intramolecular radical 1,4-addition^40^ of **14** and also promoted the intermolecular aldol reaction with aldehyde **15** to provide **13** as an inconsequential mixture of diastereomers in 81% yield.^26,27,41^ Triethyl borane plays a dual role in this process: (1) radical initiation to enable the 6-*exo*-trig cyclization of aryl radical **V** and (2) formation of boron enolate **V** to promote the aldol reaction with **15**. This sequence facilitated the convergent fusion of both fragments in high yields, establishing the crucial C3−C3a bond and the C1−C2 bond in a single step. It is important to note that the presence of both reagents, triethyl borane and tributyltin hydride, was critical for the reaction as the desired reactivity was not observed in the absence of either reagent. To the best of our knowledge, the substrate combination employed in this step is unprecedented in the chemical literature rendering it a unique transformation. Noteworthy, a stepwise process involving isolation of the 1,4-addition product and subsequent generation of boron enolate **V** in the presence of either dibutylboron triflate (*n*-Bu_2_BOTf) or dicyclohexylboron triflate (*c*-Hex_2_BOTf) were ineffective to afford **13** (see the Supporting Information for details). Surprisingly, attempts to perform the sequence in the presence of the C4a oxygen functionality failed and only the 1,4-addition product was formed (see the Supporting Information for details). Similar issues have been observed in previous attempts to install the C3−C3a bond via transition metal-catalyzed cross-coupling reactions. Consequently, we aimed for a late-stage oxidation to install the missing C4a phenol.

To form the biaryl motif, we then proceeded with the aromatization of **13** (Scheme 3). To this end, secondary alcohol **13** was first oxidized with Dess−Martin periodinane (DMP) to the ketone, followed by treatment with *p*-toluenesulfonic acid (*p*-TsOH) to give **26** in around 54% yield, although this process proved to be unreliable and difficult to reproduce. We found that exposure of the ketone to 1,8-diazabicyclo[5.4.0]undec-7-ene (DBU) leads to the formation of an inconsequential mixture of diastereomers, which upon treatment with *p*-TsOH undergoes smooth aromatization. Contrary to our initial expectations, the ^1^H NMR spectra of the 1,3-diketones obtained after DMP oxidation, as well as DBU treatment did not indicate any enol form. While this step clearly facilitates the acid-catalyzed aromatization, the exact role of DBU, presumably only epimerization, remains uncertain.

With synthetic access to phenol **26**, we then proceeded with the endgame of the synthesis. Debenzylation of **26** to alcohol **27** was affected by treatment with Pearlma’s catalyst under 50 bar hydrogen pressure. Oxidation of the resulting neopentylic alcohol **27** to the corresponding aldehyde was found to require protection of the phenol to prevent significant decomposition. Chemoselective acetylation of the phenol was achieved by treatment with acetic anhydride and triethylamine. Exposure to DMP yielded aldehyde **28**, which was converted to phenol **12** through sequential deprotections with bromotrimethylsilane (TMSBr) and hydrogen fluoride pyridine, respectively. We then attempted to install the missing oxygen functionality via selective C4a oxidation. After several failed attempts, we found that oxidation of **12** with phenyliodine bis(trifluoroacetate) (PIFA) in an aqueous mixture of acetone and acetonitrile led to smooth oxidation to give quinone **29**. The presence of an electron-rich aromatic system proved to be crucial for this oxidation, as no C4a oxidation could be achieved in the presence of the lactone at C7a. The conversion of **29** to its hydroquinone **VII** was accomplished using sodium dithionite, resulting in the formation of an unstable intermediate, presumably hemiacetal **VIII**. Immediate treatment with *p*-TsOH and trimethyl orthoformate in methanol formed spiro bisacetal **11** as a single diastereomer. We believe that the excellent diastereoselectivity is a result of anomeric stabilization, as observed in the single crystal X-ray structure of ganoapplanin (**7**) (see the Supporting Information for details). With access to the complete carbon scaffold of ganoapplanin (**7**), the final steps required a seemingly straightforward benzylic oxidation. Given the scarcity of reports on benzylic oxidations in the presence of unprotected phenols, we decided to convert phenol **11** into its acetyl ester **30**. Initial attempts to realize the oxidation of the benzylic position using standard conditions such as Jones reagent, 2,3-dichloro-5,6-dicyano-1,4-benzoquinone (DDQ) and MeOH or DDQ and *tert*-butyl hydroperoxide (TBHP) resulted in decomposition. However, employing a copper(l) mediated oxidation protocol in the presence of TBHP^42^ allowed for the formation of lactone **31** in 47% yield. Subsequent deacetylation was then affected by treatment with potassium carbonate in methanol to give ganoapplanin (**7**).

The choice of the employed protecting group proved to be crucial, as earlier attempts using benzyl protecting groups on the phenols failed to give **7** due to unsuccessful deprotections. The spectroscopic data for synthetic **7** were in full agreement with those reported in the literature.^8^ In summary, we have accomplished the first total synthesis of the dimeric *Ganoderma* meroterpenoid ganoapplanin (**7**). The bicyclic terpenoid fragment **15** was constructed employing a diastereoselective, titanium(IV)-mediated iodolactonization. For the fusion with arene component **14** we developed a highly efficient two-component radical 1,4-addition/aldol reaction sequence. Two late-stage oxidations facilitated the installation of the characteristic spiro bisacetal and lactone moieties of ganoapplanin (**7**). We expect that the efficiency of the developed key sequence will facilitate the synthesis of structurally related natural products.

## Supplementary Material

Supporting information

## Figures and Tables

**Figure 1:**
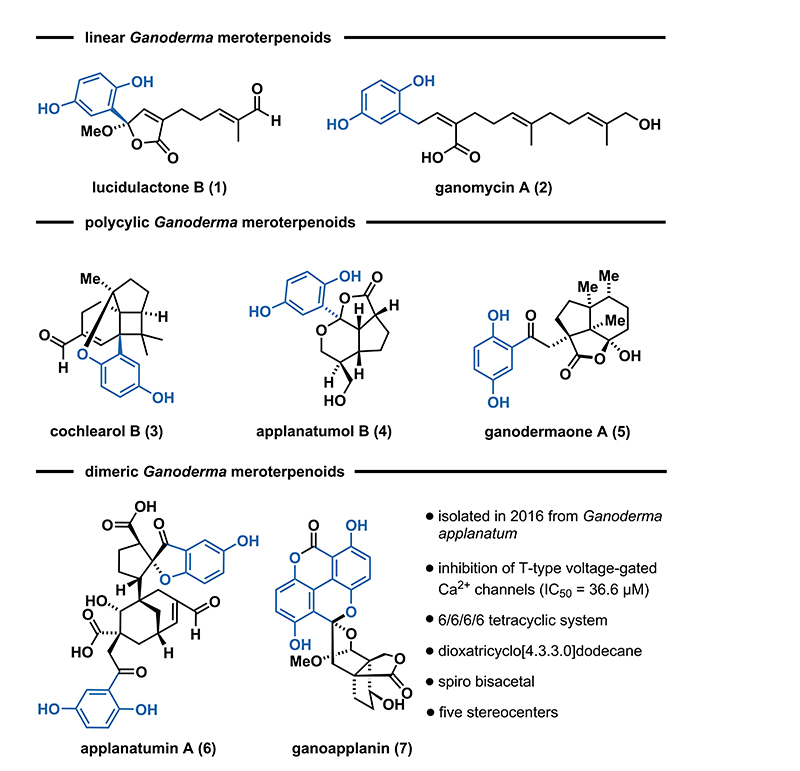
Selected structures of *Ganoderma* meroterpenoids and their classification.

**Scheme 1:**
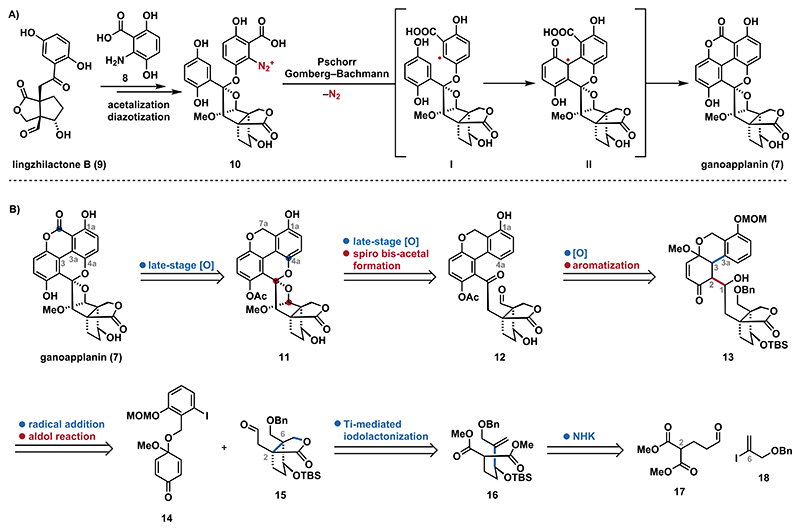
(A) Proposed Biosynthesis of Ganoapplanin (7) and (B) Retrosynthetic Disconnections

**Scheme 2:**
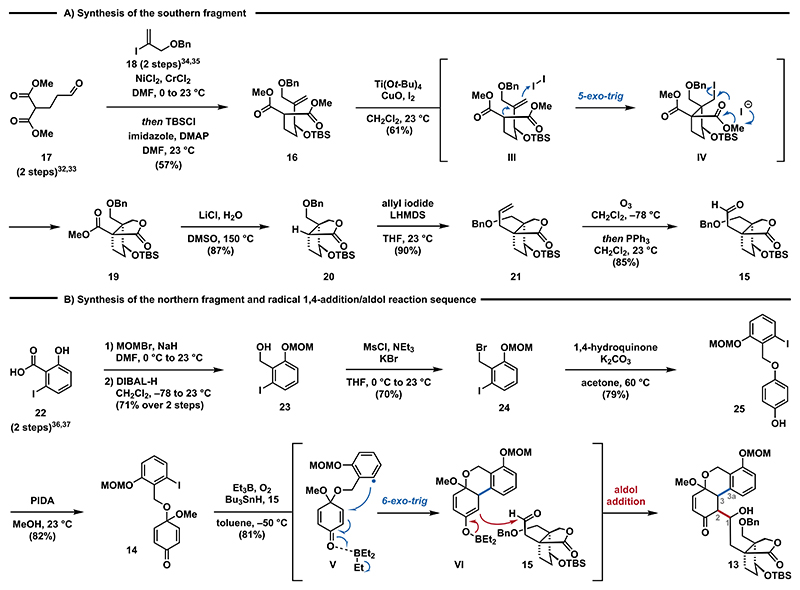
(A) Synthesis of Aldehyde 14 and a (B) Radical 1,4-Addition/Aldol Sequence

**Scheme 3:**
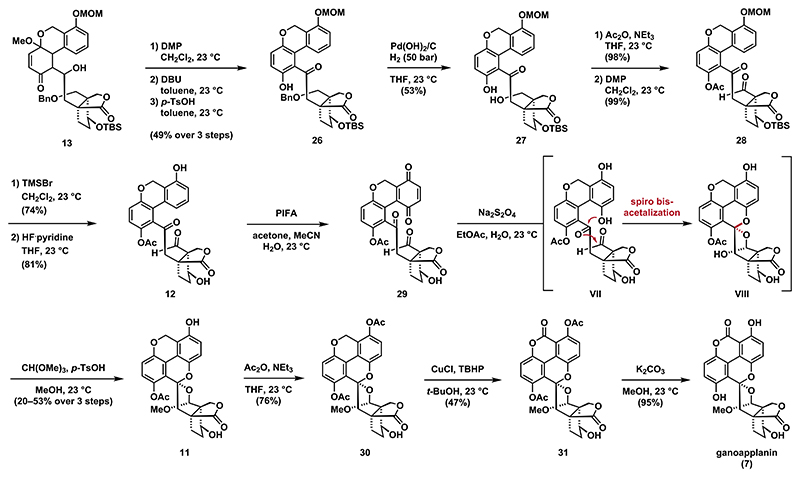
Late-Stage Oxidations and Completion of the Synthesis of Ganoapplanin
