# Supplementary material for: Total Synthesis of Ganoapplanin Enabled by a Radical Addition/Aldol Reaction Cascade
Source: J Am Chem Soc. 2024 Aug 7;146(33):22937–42. doi: 10.1021/jacs.4c08291 (PMC7616391; doi:10.1021/jacs.4c08291)

– SUPPORTING INFORMATION –

Total Synthesis of Ganoapplanin Enabled by a Radical-Addition/Aldol Reaction Cascade

Nicolas Müller[a,§], Ondřej Kováč[a,b,§], Alexander Rode[a], Daniel Atzl[a] and Thomas Magauer[a,*]

[a]Institute of Organic Chemistry and Center for Molecular Biosciences, University of Innsbruck, Innrain 80–82, 6020 Innsbruck, Austria

[b]Department of Organic Chemistry, Palacký University Olomouc, Tr. 17. Listopadu 12, 77900 Olomouc, Czech Republic

[§] Both authors contributed equally to this work.

[*]E-mail: Thomas.magauer@uibk.ac.at

Table of Contents

1. General experimental details **4**

2. Experimental section **5**

2.1. Synthesis of southern fragment 15 .... **5**

Alkenes S1 and S2 5

Aldehyde 17 6

Vinyl iodide S4 7

Benzyl trichloroacetimidate S6 8

Vinyl iodide 18 9

Alkene 16 10

Lactone 19 11

Lactone 20 12

Alkene 21 13

Aldehyde 15 14

2.2. Synthesis of northern fragment 14 .... **15**

Benzoic acid S7 15

Benzoic acid 22 16

Ester S8 17

Benzylic alcohol 23 18

Benzylic bromide 24 19

Phenol 25 20

Quinone monoacetal 14 21

2.3. Completion of the synthesis of ganoapplanin (7) **22**

Alcohol 13 22

Possible transition state for the aldol reaction 24

Phenol 26 25

Screened conditions for the aromatization of S9 27

Alcohol 27 28

Acetyl ester S10 29

Aldehyde 28 30

Phenol S11 31

Alcohol 12 32
Quinone 29........ 33
Phenol 11 34
Diastereoselectivity of the spiro bis-acetalization........ 36
Acetyl ester 30 37
Lactone 31 38
Ganoapplanin (7) 39
3. Additional studies towards the radical-addition/aldol reaction cascade 42
3.1. Screened conditions for the intramolecular radical-addition of quinone monoacetal 14 and the radical addition/aldol reaction cascade........ 42
3.2. Synthesis and application of quinone monoacetal S20 43
Aryl bromide S13........ 44
MOM-ether S14 45
Benzyl ether S15 46
Benzylic alcohol S16 47
Benzyl iodide S17........ 48
Benzyl bromide S18 49
Phenol S19 50
Quinone monoacetal S20 51
Attempts of the the radical-addition/aldol reaction cascade employing quinone monoacetal S20 52
3.3. Synthesis and application of quinone monoacetal S28 53
MOM-ether S24 54
Benzylic alcohol S25 55
Benzyl bromide S26 56
Phenol S27 57
Quinone monoacetal S27 58
Attempt of the the radical-addition/aldol reaction cascade employing quinone monoacetal S28 59
3.4. Synthesis and application of model substrate S36 60
Benzylic alcohol S32 61
Benzyl iodide S33........ 62
Phenol S34 63

Quinone monoacetal S35 64
Tricycle S36 65
Attempts of an aldol reaction employing tricycle S36 and aldehyde 15 66
4. References 67
5. ^{1}H and ^{13}C NMR Spectra 68

1. General experimental details

All reactions were carried out with magnetic stirring, and if moisture or air sensitive, under nitrogen or argon atmosphere using standard Schlenk techniques in oven-dried glassware (120 °C oven temperature). If required glassware was further dried under vacuum with a heat-gun at 650 °C. External bath thermometers were used to record all reaction temperatures. Low temperature reactions were carried out in a Dewar vessel filled with acetone/dry ice (T between –78 °C and 0 °C) or distilled water/ice (0 °C). High temperature reactions were conducted using a heated silicon oil bath or a metal block in reaction vessels equipped with a reflux condenser or in a pressure tube. Diethyl ether (Et_2O) and tetrahydrofuran (THF) were dried over molecular sieves (4Å) prior to use. All other solvents were purchased from Acros Organics as 'extra dry' reagents. All other reagents with a purity >95% were obtained from commercial sources (Sigma Aldrich, Acros, Alfa Aesar and others) and used without further purification unless otherwise stated.

Flash column chromatography (FCC) was carried out with Merck silica gel 60 (0.040–0.063 mm). Analytical thin layer chromatography (TLC) was carried out using Merck silica gel 60 F254 glass-backed plates or aluminum foils and visualized under UV-light at 254 nm. Staining was performed with ceric ammonium molybdate (CAM), an aqueous potassium permanganate solution or by staining with an aqueous para-anisaldehyde solution and subsequent heating.

NMR spectra (^{1}H NMR, ^{13}C NMR) were recorded in deuterated chloroform ($CDCl_3$), deuterated acetone ($(CD_3)_2CO$) or deuterated pyridine (C_5D_5N) on a Bruker Avance Neo 400 MHz spectrometer, a Bruker Avance II 600 MHz spectrometer, or a Bruker Avance 4 Neo 700 MHz spectrometer and are reported as follows: chemical shift δ in ppm (multiplicity, coupling constant *J* in Hz, number of protons) for ^{1}H NMR spectra and chemical shift δ in ppm for ^{13}C NMR spectra. Multiplicities are abbreviated as follows: s = singlet, d = doublet, t = triplet, q = quartet, p = quintet, br = broad, m = multiplet, or combinations thereof. Residual solvent peaks of $CDCl_3$ (δ_H = 7.26 ppm, δ_C = 77.16 ppm), $(CD_3)_2CO$ (δ_H = 2.05 ppm, δ_C = 29.84 ppm) and C_5D_5N (δ_H = 8.74 ppm, δ_C = 150.35 ppm) were used as internal reference. NMR spectra were assigned using information ascertained from COSY, HMBC, HSQC and NOESY experiments. For natural product shift comparisons of proton peaks within multiplets, the central resonance peak from the HSQC was used.

High resolution mass spectra (HRMS) were recorded on Thermo Scientific™ LTQ Orbitrap XL™ Hybrid Ion Trap-Orbitrap Mass Spectrometer at the Institute of Organic Chemistry and Center for Molecular Biosciences, University of Innsbruck.

Infrared spectra (IR) were recorded from 4000 cm^{-1} to 450 cm^{-1} on a Bruker™ ALPHA FT-IR Spectrometer from Bruker. Samples were prepared as a neat film or a film by evaporation of a solution in $CDCl_3$ or $((CD_3)_2CO)$.

All yields are isolated, unless otherwise specified.

2. Experimental section

2.1. Synthesis of southern fragment 15

Alkenes S1 and S2

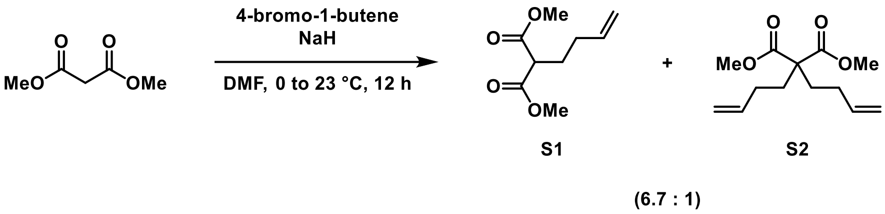

Note: The alkylation of dimethyl malonate was conducted in two parallel runs (each 378 mmol), which were combined after the work-up.

Dimethyl malonate (43.3 mL, 378 mmol, 1 equiv) was added dropwise to a suspension of sodium hydride (9.36 g, 390 mmol, 1.03 equiv, 60 wt% in mineral oil) in *N*,*N*-dimethylformamide (1.00 L) at 0 °C. After 30 min, 4-bromo-1-butene (44.0 mL, 416 mmol, 1.10 equiv) was added dropwise over 1 h at 0 °C to the reaction mixture, which was then warmed to 23 °C. After 12 h, aqueous saturated ammonium chloride solution (300 mL) and diethyl ether (500 mL) were added to the reaction mixture. The layers were separated, and the aqueous phase was extracted with diethyl ether (2 × 500 mL). The combined organic phases were washed with 10% aqueous lithium chloride solution (500 mL) and water (4 × 500 mL). The washed organic phases were dried over magnesium sulfate and the dried solution was filtered. The combined filtrates were concentrated under reduced pressure to give a crude mixture (69.8 g) of alkene **S1** and alkene **S2** (ratio 6.7:1) as a pale yellow oil, which was used in the next step without further purification. To obtain analytical data, a small aliquot of the crude reaction mixture was purified by flash column chromatography on silica gel (30% diethyl ether in petroleum ether) to give alkene **S1** and **S2**.

Analytical data for alkene **S1**:

^{1}H NMR (300 MHz, $CDCl_3$): δ = 5.76 (ddt, *J* = 16.9, 10.2, 6.3 Hz, 1H), 5.11 – 4.85 (m, 2H), 3.74 (s, 6H), 3.40 (t, *J* = 7.1 Hz, 1H), 2.22 – 1.86 (m, 4H).

^{13}C NMR (75 MHz, $CDCl_3$): δ = 169.9, 136.9, 116.2, 52.6, 51.0, 31.4, 28.1.

The obtained analytical data were in full agreement with those reported in the literature.[1]

Analytical data for alkene **S2**:

^{1}H NMR (400 MHz, $CDCl_3$): δ = 5.95 – 5.57 (m, 2H), 5.13 – 4.91 (m, 4H), 3.72 (s, 6H), 2.18 – 1.81 (m, 8H).

^{13}C NMR (101 MHz, $CDCl_3$): δ = 172.1, 137.6, 115.3, 57.2, 52.5, 31.9, 28.5.

The obtained analytical data were in full agreement with those reported in the literature.[2]

Aldehyde 17

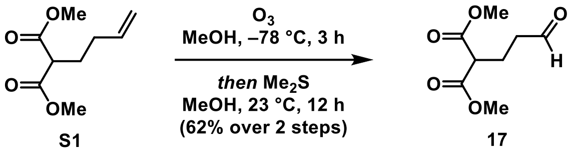

S1 17

*Note: The ozonolysis of the mixture of alkene **S1** and alkene **S2** was conducted in two parallel runs, which were combined after the work-up.*

Through a solution of alkene **S1** and alkene **S2** (assumed pure 25.0 g, 134 mmol, 1 equiv) in methanol (600 mL) and acetic acid (1.92 mL) was sparged a stream of ozone at –78 °C. After 3 h, the reaction mixture turned blue and then oxygen was sparged through the solution for 5 min. Dimethylsulfide (14.8 mL, 201 mmol, 1.50 equiv) was added at –78 °C and the reaction mixture was then warmed to 23 °C. After 12 h, the combined reaction mixtures were concentrated under reduced pressure to give crude aldehyde **17**, which was purified by flash column chromatography on silica gel (0 to 50% diethyl ether in petroleum ether) to give aldehyde **17** (31.4 g, 167 mmol, 62%) as a colorless oil.

<u>Analytical data for aldehyde **17**:</u>

^{1}H NMR (400 MHz, $CDCl_3$): δ = 9.75 (t, *J* = 1.1 Hz, 1H), 3.74 (s, 6H), 3.45 (td, *J* = 7.3, 0.9 Hz, 1H), 2.66 – 2.46 (m, 2H), 2.22 (qd, *J* = 7.3, 1.1 Hz, 2H).

^{13}C NMR (101 MHz, $CDCl_3$): δ = 200.5, 169.3, 52.7, 50.3, 41.0, 21.1.

The obtained analytical data were in full agreement with those reported in the literature.[3]

Vinyl iodide S4

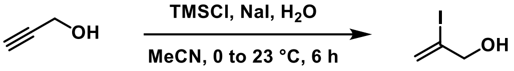

*Note: The preparation of vinyl iodide **S4** was conducted in three parallel runs (each 624 mmol), which were combined for purification by column chromatography.*

Trimethylsilyl chloride (95.0 mL, 749 mmol, 1.20 equiv) was added to a solution of sodium iodide (112 g, 749 mmol, 1.20 equiv) in a mixture of acetonitrile (100 mL) and water (6.75 mL, 374 mmol, 0.600 equiv) at 0 °C. After 10 min, propargyl alcohol **S3** (36.6 mL, 0.624 mol, 1 equiv) was added to the reaction mixture at 0 °C, which was then warmed to 23 °C. After 6 h, aqueous saturated sodium thiosulfate solution (300 mL) and ethyl acetate (300 mL) were added to the reaction mixture. The layers were separated, and the aqueous phase was extracted with ethyl acetate (2 × 500 mL). The combined organic phases were washed with aqueous saturated sodium chloride solution (500 mL) and the washed layers were dried over magnesium sulfate and the dried solution was filtered. The filtrate was concentrated under reduced pressure and the combined residues were purified by flash column chromatography on silica gel (20 to 50% ethyl acetate in petroleum ether) to give vinyl iodide **S4** (120 g, 656 mmol, 35%) as a yellow oil.

<u>Analytical data for vinyl iodide **S4**:</u>

^{1}H NMR (400 MHz, $CDCl_3$): δ = 6.39 (q, *J* = 1.7 Hz, 1H), 5.86 (dq, *J* = 2.2, 1.2 Hz, 1H), 4.18 (q, *J* = 1.3 Hz, 2H), OH not detected due to exchange events.

The obtained analytical data were in full agreement with those reported in the literature.[4]

Benzyl trichloroacetimidate S6

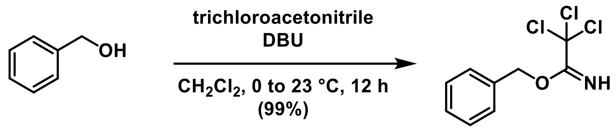

1,8-Diazabicyclo(5.4.0)undec-7-ene (DBU) (7.82 mL, 51.9 mmol, 0.100 equiv) was added slowly to a solution of benzyl alcohol **S5** (53.9 mL, 519 mmol, 1 equiv) and trichloroacetonitrile (130 mL, 1.30 mol, 2.50 equiv) in dichloromethane (1.50 L) at 0 °C, which was then warmed to 23 °C. After 12 h, the reaction mixture was concentrated under reduced pressure and the residue was purified by flash column chromatography on silica gel (20% ethyl acetate in petroleum ether) to give benzyl trichloroacetimidate **S6** (130 g, 510 mmol, 99%) as a yellow oil.

Analytical data for Benzyl trichloroacetimidate **S6**:

^{1}H NMR (400 MHz, $CDCl_3$): δ = 8.42 (s, 1H), 7.60 – 7.32 (m, 5H), 5.37 (s, 2H).

The obtained analytical data were in full agreement with those reported in the literature.[5]

Vinyl iodide 18

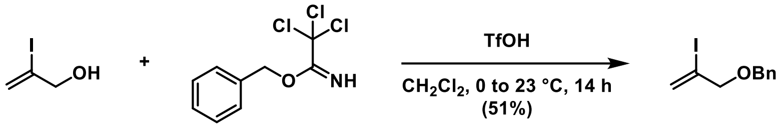

S4 **S6** **18**

Triflic acid (5.69 mL, 64.3 mmol, 0.200 equiv) was added dropwise to a solution of vinyl iodide **S4** (59.2 g, 321 mmol, 1 equiv) and benzyl trichloroacetimidate **S6** (146 g, 579 mmol, 1.80 equiv) in dichloromethane (1.10 L) at 0 °C, which was then warmed to 23 °C. After 14 h, aqueous saturated sodium thiosulfate solution (500 mL) was added to the reaction mixture at 0 °C. The layers were separated, and the aqueous phase was extracted with dichloromethane (2 × 500 mL). The combined organic phases were dried over magnesium sulfate and the dried solution was filtered. The filtrate was concentrated under reduced pressure and the residue was purified by flash column chromatography on silica gel (2 to 5% diethyl ether in petroleum ether) to give vinyl iodide **18** (45.0 g, 160 mmol, 51%) as a yellow oil.

Analytical data for vinyl iodide **18**:

^{1}H NMR (400 MHz, $CDCl_3$): δ = 7.45 – 7.29 (m, 5H), 6.43 (q, *J* = 1.6 Hz, 1H), 5.95 (q, *J* = 1.3 Hz, 1H), 4.55 (s, 2H), 4.10 (t, *J* = 1.4 Hz, 2H).

The obtained analytical data were in full agreement with those reported in the literature.[6]

Alkene 16

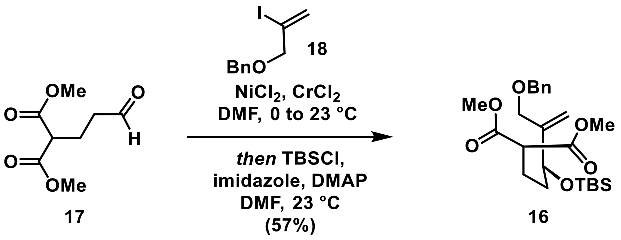

Note: N,N-dimethylformamide was degassed via freeze-pump thaw (three cycles) prior to use.

Note: The NHK reaction was conducted in four parallel runs (each 26.6 mmol), which were combined after the work-up.

A solution of aldehyde **17** (5.00 g, 26.6 mmol, 1 equiv) and vinyl iodide **18** (10.9 g, 39.9 mmol, 1.50 equiv) in degassed *N*,*N*-dimethylformamide (30.0 mL) was added to a suspension of chromium dichloride (10.8 g, 87.7 mmol, 3.30 equiv) and nickel dichloride (344 mg, 2.66 mmol, 0.100 equiv) in degassed *N*,*N*-dimethylformamide (130.0 mL) at 0 °C, which was then warmed to 23 °C. After 6 h, *tert*-butyldimethylsilyl chloride (8.01 g, 53.2 mmol, 2.00 equiv), imidazole (7.24 g, 106 mmol, 4.00 equiv) and 4-dimethylaminopyridine (325 mg, 2.66 mmol, 0.100 equiv) were added to the reaction mixture at 23 °C. After 12 h, 1 M aqueous hydrochloric acid (250 mL) and diethyl ether (250 mL) were added to the reaction mixture. The layers were separated, and the aqueous phase was extracted with diethyl ether (2 × 250 mL). The combined organic phases were washed with 1 M aqueous lithium chloride solution (500 mL) and water (500 mL). The washed organic layers were dried over magnesium sulfate and the dried solution was filtered. The filtrate was concentrated under reduced pressure and the combined residues were purified by flash column chromatography on silica gel (10% diethyl ether in pentane) to give alkene **16** (27.1 g, 60.0 mmol, 57%) as a yellow oil.

Analytical data for alkene **16**:

TLC (10% ethyl acetate in cyclohexane): R_f = 0.35 (UV, CAM).

^{1}H NMR (400 MHz, $CDCl_3$): δ = 7.38 – 7.28 (m, 5H), 5.20 – 5.11 (m, 2H), 4.50 (q, *J* = 12.0 Hz, 2H), 4.26 (t, *J* = 5.7 Hz, 1H), 4.10 – 3.89 (m, 2H), 3.71 (s, 6H), 3.35 (t, *J* = 7.5 Hz, 1H), 1.91 (p, *J* = 7.8 Hz, 2H), 1.64 – 1.50 (m, 2H), 0.89 (s, 9H), 0.05 (s, 3H), 0.01 (s, 3H).

^{13}C NMR (101 MHz, $CDCl_3$): δ = 169.9, 169.9, 147.4, 138.4, 128.5, 127.7, 127.6, 112.8, 73.4, 72.3, 70.1, 52.5, 52.5, 51.6, 34.0, 25.9, 24.6, 18.3, -4.6, -5.0.

IR (ATR, neat): 2953, 2929, 2856, 1739, 1454, 1251, 1087, 835, 776, 698 cm^{-1}.

HRMS (ESI) calc. for $C_{24}H_{38}O_6SiNa$ $[M+Na]^+$: 473.2330; found: 473.2330.

Lactone 19

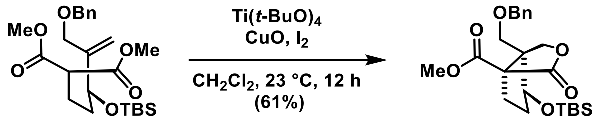

Note: The Titanium cyclization reaction was conducted in two parallel runs (each 29.3 mmol), which were combined after the work-up.

Titanium(IV) *tert*-butoxide (12.3 mL, 32.2 mmol, 1.10 equiv) was added to a solution of alkene **16** (13.2 g, 29.3 mmol, 1 equiv) in dichloromethane (290 mL) at 23 °C. After 15 min, iodine (29.7 g, 117 mmol, 4.00 equiv) and cupric oxide (2.85 g, 35.1 mmol, 1.20 equiv) were added to the reaction mixture at 23 °C. After 12 h, aqueous saturated sodium thiosulfate solution (200 mL) was added to the reaction mixture. The mixture was filtered over celite, the layers were separated and the aqueous phase was extracted with dichloromethane (2 × 250 mL). The combined organic layers were dried over magnesium sulfate and the dried solution was filtered. The filtrate was concentrated under reduced pressure and the combined residues were purified by flash column chromatography on silica gel (10% ethyl acetate in petroleum ether) to give lactone **19** (15.4 g, 35.4 mmol, 61%) as a yellow oil.

Analytical data for lactone **19**:

TLC (10% ethyl acetate in cyclohexane): R_f = 0.32 (UV, CAM).

^{1}H NMR (600 MHz, $CDCl_3$): δ = 7.36 – 7.33 (m, 2H), 7.31 – 7.28 (m, 1H), 7.26 – 7.24 (m, 2H), 4.63 (d, *J* = 9.3 Hz, 1H), 4.45 – 4.39 (m, 2H), 4.22 (dd, *J* = 8.5, 5.7 Hz, 1H), 4.01 (d, *J* = 9.3 Hz, 1H), 3.59 (s, 3H), 3.48 (d, *J* = 9.3 Hz, 1H), 3.35 (d, *J* = 9.2 Hz, 1H), 2.39 (ddd, *J* = 13.4, 11.4, 6.9 Hz, 1H), 2.24 (ddd, *J* = 13.5, 6.9, 3.3 Hz, 1H), 1.99 (dddd, *J* = 12.7, 6.8, 5.7, 3.3 Hz, 1H), 1.49 (dddd, *J* = 12.7, 11.3, 8.5, 6.9 Hz, 1H), 0.86 (s, 9H), 0.05 – 0.00 (m, 6H).

^{13}C NMR (151 MHz, $CDCl_3$): δ = 176.9, 169.2, 137.3, 128.4, 127.9, 127.6, 76.0, 73.5, 69.8, 68.7, 61.3, 58.0, 52.7, 32.7, 30.3, 25.6, 17.9, –4.5, –5.1.

IR (ATR, neat): 2954, 2929, 2856, 1778, 1743, 1252, 1147, 1036, 838, 778 cm^{-1}.

HRMS (ESI) calc. for $C_{23}H_{34}O_6SiK$ $[M+K]^+$: 473.1756; found: 473.1727.

Lactone 20

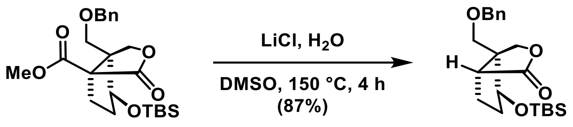

19 20

Lithium chloride (5.99 g, 140 mmol, 8.00 equiv) was added to a solution of lactone **19** (7.60 g, 17.5 mmol, 1 equiv) in dimethylsulfoxide (70 mL) and water (2.52 mL, 140 mmol, 8.00 equiv) at 23 °C, which was then heated to 150 °C. After 4 h, the reaction mixture was cooled to 23 °C and water (70 mL) and diethyl ether (70 mL) were added to the reaction mixture. The layers were separated, and the aqueous phase was extracted with diethyl ether (2 × 70 mL). The combined organic layers were dried over magnesium sulfate and the dried solution was filtered. The filtrate was concentrated under reduced pressure and the combined residues were purified by flash column chromatography on silica gel (15% ethyl acetate in petroleum ether) to give lactone **20** (5.72 g, 15.2 mmol, 87%) as a colorless oil.

Analytical data for lactone **20**:

TLC (15% ethyl acetate in cyclohexane): R_f = 0.39 (UV, CAM).

^{1}H NMR (400 MHz, $CDCl_3$): δ = 7.40 – 7.28 (m, 5H), 4.55 (d, *J* = 9.7 Hz, 1H), 4.53 (s, 2H), 4.15 (dd, *J* = 9.1, 5.3 Hz, 1H), 3.92 (d, *J* = 9.7 Hz, 1H), 3.42 – 3.37 (m, 2H), 2.88 – 2.84 (m, 1H), 2.07 – 1.99 (m, 1H), 1.96 – 1.85 (m, 2H), 1.64 – 1.57 (m, 1H), 0.86 (s, 9H), 0.05 – 0.01 (m, 6H).

^{13}C NMR (101 MHz, $CDCl_3$): δ = 180.8, 137.8, 128.6, 128.0, 127.8, 76.1, 73.6, 72.3, 69.9, 54.3, 46.0, 33.7, 26.1, 25.8, 18.0, –4.4, –5.0.

IR (ATR, neat): 2954, 2929, 2856, 1770, 1463, 1362, 1253, 1147, 1113, 1028, 866, 837, 777, 698 cm^{-1}.

HRMS (ESI) calc. for $C_{21}H_{32}O_4SiNa$ $[M+Na]^+$: 399.1962; found: 399.1934.

Alkene 21

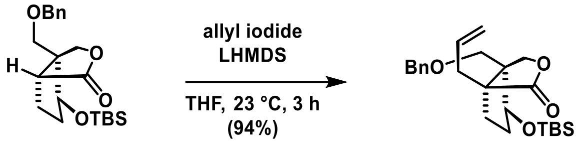

Lithium bis(trimethylsilyl)amide (40.2 mL, 1.00 M in tetrahydrofuran, 40.2 mmol, 3.00 equiv) was added to a solution of lactone **20** (5.05 g, 13.4 mmol, 1 equiv) and allyl iodide (12.3 mL, 134 mmol, 10.0 equiv) in tetrahydrofuran (150 mL) at 23 °C. After 3 h, water (150 mL) and ethyl acetate (150 mL) were added to the reaction mixture. The layers were separated, and the aqueous phase was extracted with ethyl acetate (2 × 150 mL). The combined organic layers were dried over magnesium sulfate and the dried solution was filtered. The filtrate was concentrated under reduced pressure and the combined residues were purified by flash column chromatography on silica gel (5% ethyl acetate in petroleum ether) to give alkene **21** (5.25 g, 12.6 mmol, 94%) as a slightly yellow oil.

Analytical data for alkene **21**:

TLC (5% ethyl acetate in cyclohexane): R_f = 0.29 (UV, CAM).

^{1}H NMR (600 MHz, $CDCl_3$): δ = 7.36 (dd, *J* = 8.3, 6.2 Hz, 2H), 7.33 – 7.28 (m, 3H), 5.87 (ddt, *J* = 17.2, 10.2, 7.2 Hz, 1H), 5.08 – 5.03 (m, 2H), 4.56 (d, *J* = 9.4 Hz, 1H), 4.49 (s, 2H), 4.27 (dd, *J* = 9.0, 6.2 Hz, 1H), 3.75 (d, *J* = 9.4 Hz, 1H), 3.52 (d, *J* = 9.4 Hz, 1H), 3.41 (d, *J* = 9.4 Hz, 1H), 2.55 (dd, *J* = 14.4, 7.4 Hz, 1H), 2.44 (ddt, *J* = 14.4, 7.1, 1.7 Hz, 1H), 2.07 (ddd, *J* = 13.2, 7.3, 2.5 Hz, 1H), 1.91 (dtd, *J* = 13.0, 6.6, 2.5 Hz, 1H), 1.62 (ddd, *J* = 13.2, 11.6, 6.8 Hz, 1H), 1.46 – 1.39 (m, 1H), 0.87 – 0.83 (m, 9H), 0.03 – 0.01 (m, 6H).

^{13}C NMR (151 MHz, $CDCl_3$) δ 182.2, 137.7, 133.9, 128.6, 128.0, 127.9, 118.5, 76.3, 73.6, 70.3, 68.6, 55.2, 54.3, 37.3, 33.5, 32.6, 25.8, 18.0, –4.3, –5.0.

IR (ATR, neat): 2954, 2929, 2856, 1765, 1463, 1362, 1252, 1144, 1124, 1029, 837, 698, 673 cm^{-1}.

HRMS (ESI) calc. for $C_{24}H_{37}O_4Si$ $[M+H]^+$:417.2456; found: 417.2449.

Aldehyde 15

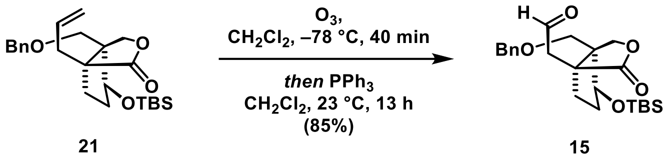

Through a solution of alkene **21** (3.81 g, 9.14 mmol, 1 equiv) in dichloromethane (100 mL) was sparged a stream of ozone at –78 °C. After 40 min, the reaction mixture turned blue and then oxygen was sparged through the solution for 5 min. Triphenylphosphine (7.20 g, 27.4 mmol, 3.00 equiv) was added at –78 °C and the resulting solution was warmed to 23 °C. After 13 h, the reaction mixture was concentrated under reduced pressure and the residue was purified by flash column chromatography on silica gel (15% ethyl acetate in petroleum ether) to give aldehyde **15** (3.26 g, 7.79 mmol, 85%) as a white solid.

Analytical data for aldehyde **15**:

TLC (15% ethyl acetate in cyclohexane): R_f = 0.25 (UV, CAM).

^{1}H NMR (600 MHz, $CDCl_3$): δ = 9.52 (s, 1H), 7.38 – 7.34 (m, 2H), 7.33 – 7.31 (m, 1H), 7.28 – 7.26 (m, 2H), 4.66 (d, *J* = 9.4 Hz, 1H), 4.46 (d, *J* = 11.7 Hz, 1H), 4.35 (dd, *J* = 11.1, 6.2 Hz, 2H), 3.82 (d, *J* = 9.4 Hz, 1H), 3.35 (d, *J* = 9.7 Hz, 1H), 3.29 (d, *J* = 9.7 Hz, 1H), 3.08 (dd, *J* = 18.7, 1.1 Hz, 1H), 2.90 (d, *J* = 18.7 Hz, 1H), 2.06 (dd, *J* = 13.0, 6.8 Hz, 1H), 1.91 – 1.86 (m, 1H), 1.64 (td, *J* = 13.2, 6.2 Hz, 1H), 1.50 – 1.43 (m, 1H), 0.87 (s, 9H), 0.06 – 0.01 (m, 6H).

^{13}C NMR (151 MHz, $CDCl_3$): δ = 199.9, 182.2, 137.3, 128.7, 128.3, 128.2, 75.3, 73.7, 69.7, 68.5, 54.0, 51.0, 48.2, 34.6, 31.5, 25.9, 18.1, –4.3, –5.0.

IR (ATR, neat): 2954, 2928, 2855, 1765, 1721, 1386, 1252, 1153, 1130, 1034, 871, 837, 777, 699 cm^{-1}.

HRMS (ESI) calc. for $C_{23}H_{34}O_5SiNa$ $[M+Na]^+$: 441.2068; found: 441.2080.

2.2. Synthesis of northern fragment 14

Benzoic acid S7

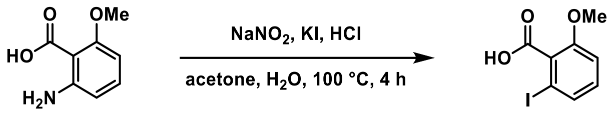

S7

Sodium nitrite (20.6 g, 299 mmol, 2.00 equiv) was added to a solution of 2-amino-6-methoxybenzoic acid (25.0 g, 150 mmol, 1 equiv) in a mixture acetone (100 mL), water (200 mL) and concentrated aqueous hydrochloric acid (80 mL) at 0 °C. After 30 min, potassium iodide (49.7 g, 299 mmol, 2.00 equiv) was added in small portions and the reaction mixture was warmed to 100 °C. After 4 h, the reaction mixture was cooled to 23 °C and aqueous saturated ammonium chloride solution (200 mL) and dichloromethane (200 mL) were added to the reaction mixture. The layers were separated, and the aqueous phase was extracted with dichloromethane (2 × 200 mL). The combined organic phases were dried over magnesium sulfate and the dried solution was filtered. The filtrate was concentrated under reduced pressure to give crude benzoic acid **S7** (41.6 g), which was used in the next step without further purification as a brown solid.

Analytical data for benzoic acid **S7**:

^{1}H NMR (400 MHz, $CDCl_3$): δ = 7.38 (dd, *J* = 7.8, 0.9 Hz, 1H), 7.02 (t, *J* = 8.2 Hz, 1H), 6.87 (dd, *J* = 8.4, 0.8 Hz, 1H), 3.80 (s, 3H).

^{13}C NMR (101 MHz, $CDCl_3$): δ = 171.2, 157.0, 132.1, 131.5, 129.1, 111.0, 92.5, 56.4.

The obtained analytical data were in full agreement with those reported in the literature.[7]

Benzoic acid 22

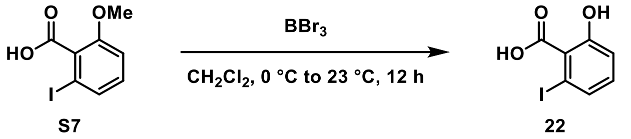

Boron tribromide (29.7 mL, 314 mmol, 2.10 equiv) was added to a solution of benzoic acid **S7** (assumed pure 41.6 g, 150 mmol, 1 equiv) in dichloromethane (1.00 L) at 0 °C, which was then warmed to 23 °C. After 12 h, the reaction mixture was cooled to 0 °C and 1 M aqueous hydrochloric acid (500 mL) was added to the reaction mixture. The layers were separated, and the aqueous phase was extracted with dichloromethane (2 × 500 mL). The combined organic phases were dried over magnesium sulfate and the dried solution was filtered. The filtrate was concentrated under reduced pressure to give crude benzoic acid **22** (39.5 g), which was used in the next step without further purification, as a brown solid.

<u>Analytical data for benzoic acid **22**:</u>

^{1}H NMR (400 MHz, $CDCl_3$): δ = 7.59 (dd, *J* = 7.5, 1.4 Hz, 1H), 7.00 (dd, *J* = 8.4, 7.5 Hz, 1H), 6.94 (dd, *J* = 8.4, 1.3 Hz, 1H), COOH and OH not detected due to exchange events.

Ester S8

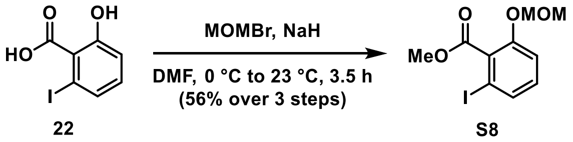

Sodium hydride (23.9 g, 598 mmol, 4.00 equiv, 60 wt% in mineral oil) was added in small portions to a solution of benzoic acid **22** (assumed pure 39.5 g, 150 mmol, 1 equiv) in *N*,*N*-dimethylformamide (1.00 L) at 0 °C. After 30 min, bromomethyl methyl ether (30.4 mL, 374 mmol, 2.50 equiv) was added to the reaction mixture, which was then warmed to 23 °C. After 3.5 h, the reaction mixture was cooled to 0 °C and water (500 mL) and ethyl acetate (500 mL) were added to the reaction mixture. The layers were separated, and the aqueous phase was extracted with ethyl acetate (2 × 500 mL). The combined organic phases were washed with water (3 × 500 mL). The washed organic layers were dried over magnesium sulfate and the dried solution was filtered. The filtrate was concentrated under reduced pressure and the residue was purified by flash column chromatography on silica gel (20% ethyl acetate in petroleum ether) to give ester **S8** (27.0 g, 83.7 mmol, 56% over 3 steps) as a yellow oil.

Analytical data for ester **S8**:

TLC (20% ethyl acetate in petroleum ether): R_f = 0.40 (UV, CAM).

^{1}H NMR (400 MHz, $CDCl_3$): δ = 7.37 (dd, *J* = 7.9, 0.9 Hz, 1H), 7.07 (dd, *J* = 8.5, 1.0 Hz, 1H), 6.96 (t, *J* = 8.2 Hz, 1H), 5.09 (s, 2H), 3.88 (s, 3H), 3.38 (s, 3H).

^{13}C NMR (101 MHz, $CDCl_3$): δ = 167.0, 154.4, 132.2, 131.8, 114.5, 94.9, 92.0, 91.9, 58.3, 56.5.

IR (ATR, neat): 2924, 2852, 2581, 1648, 1587, 1563, 1444, 1428, 1305, 1296, 1213, 1187, 1061, 910, 869, 803, 719, 694 cm^{-1}.

HRMS (ESI) calc. for $C_{10}H_{12}IO_4^+$ $[M+H]^+$: 322.9775; found: 322.9772.

Benzylic alcohol 23

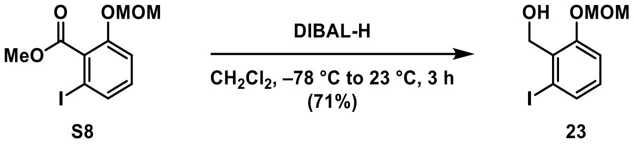

A solution of diisobutylaluminum hydride (1.00 M in hexanes, 180 mL, 180 mmol, 2.20 equiv) was added slowly to a solution of ester **S8** (26.3 g, 81.7 mmol, 1 equiv) in dichloromethane (400 mL) at –78 °C, which was then warmed to 23 °C after complete addition. After 3 h, the reaction mixture was poured into an aqueous saturated sodium potassium tartrate solution (1.00 L) and the mixture was stirred vigorously for 12 h. The formed suspension was then filtered over celite, the obtained layers were separated, and the aqueous phase was extracted with dichloromethane (2 × 500 mL). The combined organic layers were dried over magnesium sulfate and the dried solution was filtered. The filtrate was concentrated under reduced pressure and the residue was purified by flash column chromatography on silica gel (20 to 30% ethyl acetate in petroleum ether) to give benzylic alcohol **23** (17.0 g, 57.6 mmol, 71%) as a yellow solid.

Analytical data for benzylic alcohol **23**:

TLC (30% ethyl acetate in petroleum ether): R_f = 0.56 (UV, CAM).

^{1}H NMR (400 MHz, $CDCl_3$): δ = 7.51 (d, *J* = 7.8 Hz, 1H), 7.11 (d, *J* = 8.3 Hz, 1H), 6.95 (t, *J* = 8.1 Hz, 1H), 5.23 (d, *J* = 1.0 Hz, 2H), 4.88 (d, *J* = 6.9 Hz, 2H), 3.50 (d, *J* = 1.0 Hz, 3H), 2.30 (t, *J* = 6.9 Hz, 1H).

^{13}C NMR (101 MHz, $CDCl_3$): δ = 155.8, 133.1, 132.5, 130.7, 115.0, 101.0, 95.1, 65.0, 56.4.

IR (ATR, neat): 3417, 2929, 1583, 1567, 1450, 1403, 1252, 1203, 1154, 1080, 992, 922, 831, 773, 732, 612 cm^{-1}.

HRMS (ESI) calc. for $C_9H_{11}IO_3Na^+$ [M+Na]$^+$: 316.9645; found: 316.9642.

Benzylic bromide 24

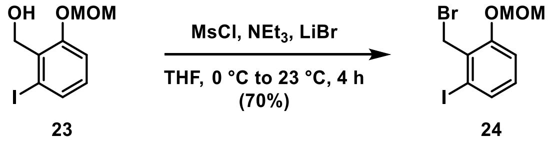

Triethylamine (20.1 mL, 144 mmol, 2.50 equiv) and methanesulfonyl chloride (11.2 mL, 144 mmol, 2.50 equiv) were added to a solution of benzylic alcohol **23** (17.0 g, 57.6 mmol, 1 equiv) in tetrahydrofuran (570 mL) at 0 °C. After 5 min, lithium bromide (50.1 g, 576 mmol, 10.0 equiv) was added to the reaction mixture at 0 °C, which was then warmed to 23 °C. After 4 h, aqueous saturated sodium bicarbonate solution (200 mL) and dichloromethane (200 mL) were added to the reaction mixture. The layers were separated, and the aqueous phase was extracted with dichloromethane (2 × 200 mL). The combined organic layers were dried over magnesium sulfate and the dried solution was filtered. The filtrate was concentrated under reduced pressure and the residue was purified by flash column chromatography on silica gel (2 to 5% ethyl acetate in petroleum ether) to give benzylic bromide **24** (14.4 g, 40.3 mmol, 70%) as a white solid.

<u>Analytical data for benzylic bromide **24**:</u>

TLC (5% ethyl acetate in petroleum ether): R_f = 0.45 (UV, CAM).

^{1}H NMR (400 MHz, $CDCl_3$): δ = 7.50 (dd, *J* = 7.9, 1.1 Hz, 1H), 7.08 (dd, *J* = 8.3, 1.1 Hz, 1H), 6.95 (t, *J* = 8.1 Hz, 1H), 5.26 (s, 2H), 4.76 (s, 2H), 3.52 (s, 3H).

^{13}C NMR (101 MHz, $CDCl_3$): δ = 155.5, 133.0, 131.2, 129.7, 114.3, 101.8, 94.5, 56.6, 34.2.

IR (ATR, neat): 3294, 2923, 2852, 2030, 2000, 1950, 1587, 1457, 1276, 1211, 1094, 890, 806, 602 cm^{-1}.

HRMS (ESI) calc. for $C_9H_{10}BrIO_2$ $[M–Br]^+$: 276.9720; found: 276.9718.

Phenol 25

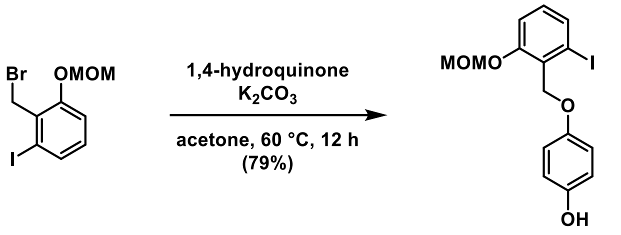

24 **25**

Potassium carbonate (8.15 g, 59.0 mmol, 1.00 equiv) was added to a solution of benzyl bromide **24** (21.1 g, 59.0 mmol, 1 equiv) and 1,4-hydrochinone (39.0 g, 354 mmol, 6.00 equiv) in acetone (590 mL) at 23 °C and the resulting mixture was warmed to 60 °C. After 12 h, the reaction mixture was cooled to 23 °C and concentrated under reduced pressure. Water (300 mL) and dichloromethane (300 mL) were added to the formed residue, the layers were separated, and the aqueous phase was extracted with dichloromethane (2 × 300 mL). The combined organic layers were dried over magnesium sulfate and the dried solution was filtered. The filtrate was concentrated under reduced pressure and the residue was purified by flash column chromatography on silica gel (20 to 30% ethyl acetate in petroleum ether) to give phenol **25** (18.1 g, 46.8 mmol, 79%) as a yellow solid.

Analytical data for phenol **25**:

TLC (20% ethyl acetate in petroleum ether): R_f = 0.29 (UV, CAM).

^{1}H NMR (400 MHz, $CDCl_3$): δ = 7.58 (dd, *J* = 7.9, 1.1 Hz, 1H), 7.16 (dd, *J* = 8.4, 1.1 Hz, 1H), 7.02 (t, *J* = 8.1 Hz, 1H), 6.98 – 6.92 (m, 2H), 6.83 – 6.77 (m, 2H), 5.20 (s, 2H), 5.16 (s, 2H), 4.61 (s, 1H), 3.48 (s, 3H).

^{13}C NMR (101 MHz, $CDCl_3$): δ = 156.2, 153.3, 149.9, 133.1, 131.3, 128.7, 116.5, 115.9, 114.9, 102.6, 94.9, 69.5, 56.3.

IR (ATR, neat): 3389, 2925, 1585, 1568, 1508, 1454, 1380, 1252, 1224, 1156, 1137, 1080, 993, 932, 873, 826, 769, 721 cm^{-1}.

HRMS (ESI) calc. for $C_{15}H_{15}IO_4$ $[M–I]^+$: 283.0941; found: 283.0935.

Quinone monoacetal 14

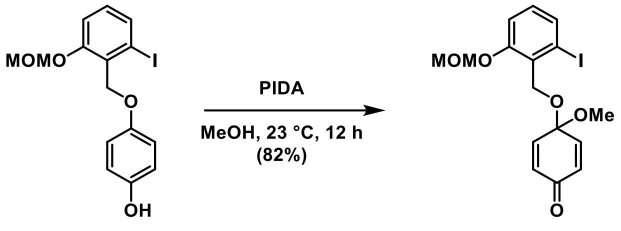

(Diacetoxyiodo)benzene (3.61 g, 11.2 mmol, 1.03 equiv) was added in small portions to a solution of phenol **25** (4.20 g, 10.9 mmol, 1 equiv) in dry methanol (55 mL) at 23 °C. After 12 h, water (150 mL) and diethyl ether (150 mL) were added to the reaction mixture. The layers were separated, and the aqueous phase was extracted with diethyl ether (2 × 100 mL). The combined organic layers were dried over magnesium sulfate and the dried solution was filtered. The filtrate was concentrated under reduced pressure and the residue was purified by flash column chromatography on silica gel (20% ethyl acetate and 2% triethylamine in petroleum ether) to give enone **14** (3.72 g, 8.93 mmol, 82%) as a yellow oil.

Analytical data for quinone monoacetal **14**:

TLC (20% ethyl acetate in petroleum ether): R_f = 0.40 (UV, CAM).

^{1}H NMR (400 MHz, $CDCl_3$): δ = 7.51 (dd, *J* = 7.9, 1.1 Hz, 1H), 7.09 (dd, *J* = 8.4, 1.1 Hz, 1H), 7.04 – 6.91 (m, 3H), 6.40 – 6.24 (m, 2H), 5.17 (s, 2H), 4.79 (s, 2H), 3.50 (s, 3H), 3.45 (s, 3H).

^{13}C NMR (101 MHz, $CDCl_3$): δ = 185.5, 156.2, 143.8, 133.1, 131.4, 129.9, 129.0, 114.7, 102.7, 94.9, 92.8, 64.4, 56.4, 51.0.

IR (ATR, neat): 2926, 1720, 1687, 1637, 1584, 1567, 1453, 1383, 1307, 1253, 1156, 1138, 1100, 1082, 965, 921, 846, 776 cm^{-1}.

HRMS (ESI) calc. for $C_{16}H_{18}IO_5$ $[M+H]^+$: 417.0193; found: 417.0186.

2.3. Completion of the synthesis of ganoapplanin (7)

Alcohol 13

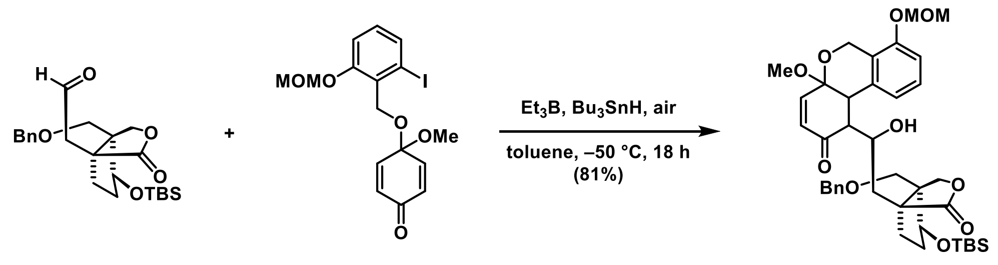

15 14 13

Note: The radical 1,4-addition/aldol sequence was conducted in two parallel runs (each 2.39 mmol) which were combined for purification by column chromatography.

Tributyltin hydride (1.20 mL, 4.30 mmol, 1.80 equiv) was added to a solution of aldehyde **15** (1.00 g, 2.39 mmol, 1 equiv) and quinone monoacetal **14** (1.59 g, 3.82 mmol, 1.60 equiv) in toluene (40.0 mL) at 23 °C. Triethylborane (9.55 mL, 1.00 M in tetrahydrofuran, 9.55 mmol, 4.00 equiv) was then added at –50 °C, followed by the addition of air (40.0 mL), which was bubbled through the solution. After 18 h, water (40 mL) and ethyl acetate (40 mL) were added to the reaction mixture. The layers were separated, and the aqueous phase was extracted with ethyl acetate (2 × 50 mL). The combined organic phases were dried over magnesium sulfate and the dried solution was filtered. The filtrate was concentrated under reduced pressure and the residue was purified by flash column chromatography on silica gel (20 to 30% ethyl acetate and 2% triethylamine in petroleum ether) to give alcohol **13** (2.74 g, 3.87 mmol, 81%) as a mixture of diastereomers as a pale yellow oil.

Analytical data for alcohol **13**:

TLC (30% ethyl acetate in petroleum ether): R_f = 0.19 (UV, CAM).

Note: As the diastereomers could not be isolated, the obtained analytical data refer to a complex mixture. We therefore only report the observed shifts.

^{1}H NMR (400 MHz, $CDCl_3$): δ = 7.40 – 7.09 (m), 7.08 – 7.00 (m), 7.01 – 6.89 (m), 6.78 (d, *J* = 7.6 Hz), 6.65 (d, *J* = 7.6 Hz), 6.27 (d, *J* = 10.5 Hz), 6.20 – 6.08 (m), 5.25 – 5.16 (m), 5.15 – 5.03 (m), 4.98 (dd, *J* = 16.1, 9.8 Hz), 4.80 – 4.72 (m), 4.71 – 4.63 (m), 4.51 (d, *J* = 9.4 Hz), 4.42 (d, *J* = 9.1 Hz), 4.30 (d, *J* = 11.9 Hz), 4.23 (d, *J* = 11.9 Hz), 4.21 – 4.12 (m), 4.09 (d, *J* = 11.5 Hz), 4.06 – 3.95 (m), 3.74 (dt, *J* = 4.3, 2.1 Hz), 3.61 (d, *J* = 9.4 Hz), 3.49 (s), 3.48 (d, *J* = 1.2 Hz), 3.47 (s), 3.42 (s), 3.38 (s), 3.31 – 3.25 (m), 3.21 (d, *J* = 9.3 Hz), 3.18 – 3.09 (m), 3.09 – 3.02 (m), 2.84 (d, *J* = 10.0 Hz), 2.73 – 2.65 (m), 2.65 – 2.59 (m), 2.10 – 1.91 (m), 1.86 (dd, *J* = 14.8, 3.1 Hz), 1.77 (dtd, *J* = 13.5, 7.0, 6.5, 3.3 Hz), 1.71 – 1.49 (m), 1.42 – 1.21 (m), 0.82 (s), 0.80 (s), -0.01 (s), -0.04 (s), -0.06 (s), -0.09 (s).

^{13}C NMR (101 MHz, $CDCl_3$): δ = 200.4, 199.2, 198.2, 183.2, 182.1, 152.9, 152.7, 142.8, 142.1, 140.1, 137.8, 137.5, 135.3, 133.3, 132.9, 132.3, 131.5, 131.0, 128.8, 128.6, 128.5, 128.5, 128.2, 128.1, 128.1, 128.0, 127.9, 127.9, 127.8, 127.8, 127.7, 127.4, 124.1, 121.8, 121.5, 121.3, 121.1, 112.0, 111.9, 111.6, 95.7, 94.9, 94.5, 94.4, 94.4,

94.0, 76.0, 75.9, 73.4, 73.4, 70.6, 70.3, 68.4, 68.1, 66.6, 65.6, 61.6, 60.6, 60.3, 58.0, 56.3, 56.3, 56.2, 56.0, 55.2, 54.9, 53.5, 53.4, 49.8, 49.6, 49.5, 44.9, 44.6, 43.1, 41.1, 39.6, 38.3, 34.4, 33.9, 32.8, 32.2, 25.8, 25.8, 18.0, 18.0, -4.3, -4.4, -5.1, -5.1.

IR (ATR, neat): 2956, 2926, 2857, 1765, 1683, 1592, 1496, 1410, 1391, 1253, 1209, 1134, 1108, 1058, 1024, 957, 922, 837, 760 cm^{-1}.

HRMS (ESI) calc. for $C_{39}H_{52}O_{10}SiNa^+$ $[M+Na]^+$: 731.3222; found: 731.3209.

Possible transition state for the aldol reaction

The aldol reaction proceeds via a boron-mediated six-membered transition state. However, we were unable to identify the major product from the resulting diastereomeric mixture. One possible transition state is shown in Scheme **1**, leading to diastereomer **13`**. Based on the report of Renaud[8] we assume the initial radical addition leads to the *cis*-fused ring system. In the subsequent aldol addition, we assume aldehyde **15** approaches enolate **II** from the top face to give the stereochemistry depicted for **13`**.

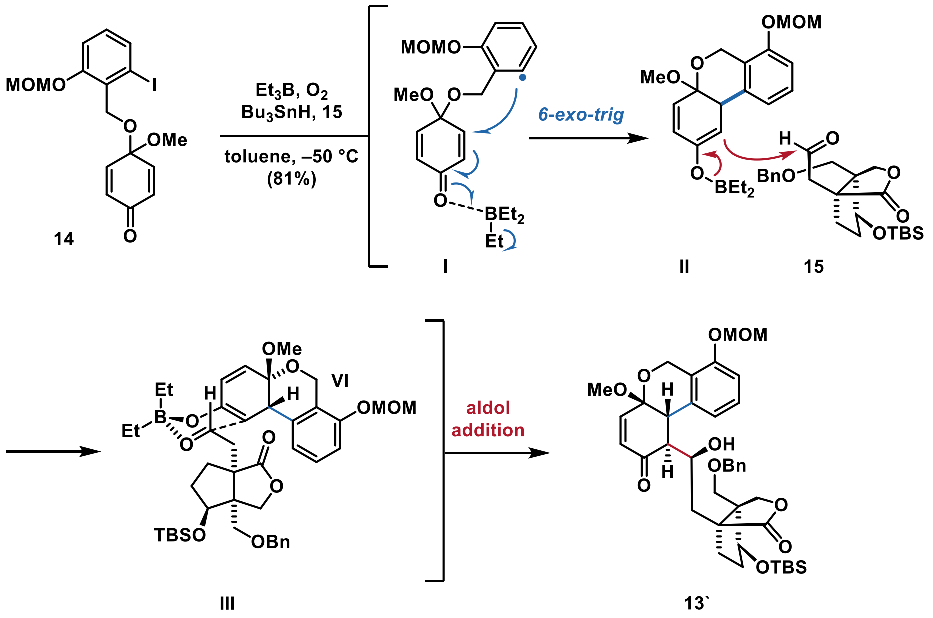

Scheme 1: Aldol reaction via six-membered transition state **III**.

Phenol 26

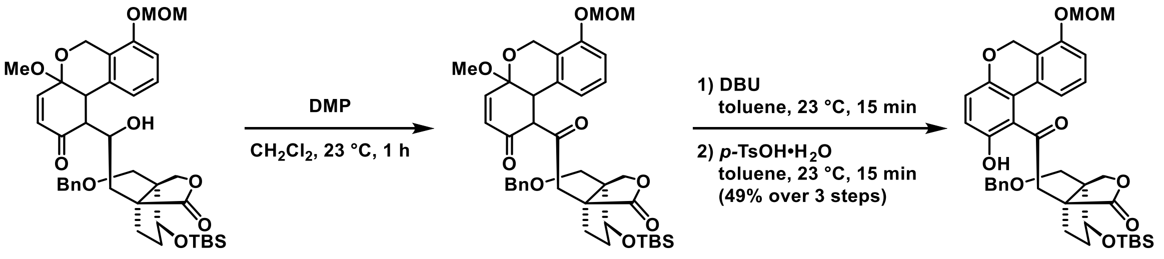

Dess–Martin periodinane (5.36 g, 12.6 mmol, 2.00 equiv) was added to a solution of alcohol **13** (4.48 g, 6.32 mmol, 1 equiv) in dichloromethane (60.0 mL) at 23 °C. After 1 h, aqueous saturated sodium thiosulfate solution (60 mL) was added to the reaction mixture. The layers were separated, and the aqueous phase was extracted with ethyl acetate (2 × 60 mL). The combined organic phases were dried over magnesium sulfate and the dried solution was filtered. The filtrate was concentrated under reduced pressure to give crude ketone **S9** as a brown solide, which was used in the next step without further purification.

1,8-Diazabicyclo(5.4.0)undec-7-ene (DBU) (1.91 mL, 12.6 mmol, 2.00 equiv) was added to a solution of crude ketone **S9** (assumed pure 4.46 g, 6.32 mmol, 1 equiv) in toluene (200 mL) at 23 °C. After 15 min, aqueous saturated sodium hydrogen carbonate solution (100 mL) was added to the reaction mixture. The layers were separated, and the aqueous phase was extracted with ethyl acetate (2 × 100 mL). The combined organic phases were dried over magnesium sulfate and the dried solution was filtered. The filtrate was concentrated under reduced pressure and the residue was dissolved in toluene (200 mL). *p*-Toluenesulfonic acid monohydrate (1.08 g, 5.69 mmol, 0.900 equiv) was added to the reaction mixture at 23 °C. After 15 min, aqueous saturated sodium hydrogen carbonate solution (100 mL) was added to the reaction mixture. The layers were separated, and the aqueous phase was extracted with ethyl acetate (2 × 100 mL). The combined organic phases were dried over magnesium sulfate and the dried solution was filtered. The filtrate was concentrated under reduced pressure and the residue was purified by flash column chromatography on silica gel (20 to 30% ethyl acetate in petroleum ether) to give phenol **26** (2.10 g, 3.11 mmol, 49% over 3 steps) as a yellow solid.

Analytical data for phenol **26**:

^{1}H NMR (400 MHz, $CDCl_3$): δ = 10.42 (s, 1H), 7.32 – 7.27 (m, 5H), 7.14 (d, *J* = 8.9 Hz, 1H), 7.10 (d, *J* = 8.3 Hz, 1H), 7.02 – 6.95 (m, 2H), 6.88 (d, *J* = 7.7 Hz, 1H), 5.37 (d, *J* = 12.8 Hz, 1H), 5.30 – 5.16 (m, 2H), 4.70 (d, *J* = 9.2 Hz, 1H), 4.39 – 4.22 (m, 2H), 4.02 (d, *J* = 11.5 Hz, 1H), 3.89 (d, *J* = 11.5 Hz, 1H), 3.70 (d, *J* = 9.2 Hz, 1H), 3.58 (d, *J* = 18.2 Hz, 1H), 3.52 (s, 3H), 3.41 – 3.16 (m, 2H), 3.10 (d, *J* = 10.0 Hz, 1H), 1.85 (d, *J* = 6.5 Hz, 1H), 1.74 (t, *J* = 6.2 Hz, 1H), 1.36 (t, *J* = 5.6 Hz, 2H), 0.84 (s, 9H), -0.00 (s, 3H), -0.06 (s, 3H).

^{13}C NMR (101 MHz, $CDCl_3$): δ = 206.4, 182.8, 154.7, 152.6, 149.6, 137.5, 130.7, 129.1, 128.3, 127.8, 127.3, 124.4, 122.8, 122.6, 120.8, 119.9, 118.7, 114.2, 94.9, 74.8, 72.9, 70.0, 68.0, 62.8, 56.4, 53.2, 53.0, 48.0, 35.0, 31.0, 25.7, 17.9, -4.5, -5.2.

IR (ATR, neat): 2955, 2856, 1765, 1632, 1574, 1437, 1361, 1247, 1154, 1127, 1034, 998, 937, 890, 860, 836, 776, 732, 699 cm^{-1}.

HRMS (ESI) calc. for $C_{38}H_{47}O_9Si^+$ $[M+H]^+$: 675.2984; found: 675.2965.

Screened conditions for the aromatization of S9

Table 1: Screened conditions.

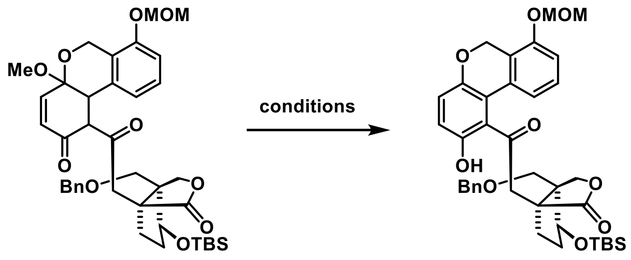

S9 **26**

Entry	Condition	Result
**1**	CSA (0.2 equiv), 0 °C to 23 °C, 3 h	Traces of **26** + decomposition
**2**	*p*-TsOH (0.2 equiv), 0 °C, 15 min	Traces of **26** + recovered **S9**
**3**	*p*-TsOH (0.2 equiv), 0 °C to 23 °C, 15 min	Traces of **26** + recovered **S9**
**4**	*p*-TsOH (0.2 equiv), 23 °C, 15 min (30 mg scale)	54% of **26**
**5**	*p*-TsOH (0.2 equiv), 23 °C, 15 min (200 mg scale)	Traces of **26**, mainly recovered **S9**

Alcohol 27

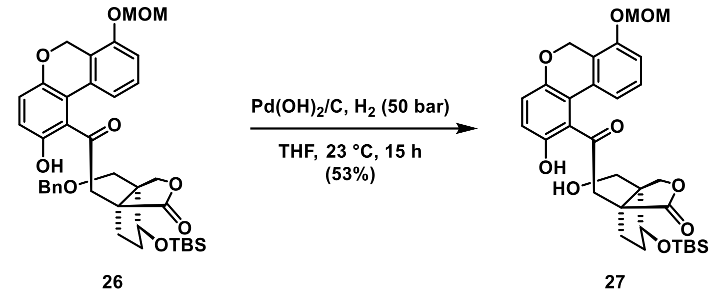

Palladium hydroxide on activated charcoal (1.58 g, 20 wt%, 2.25 mmol, 0.800 equiv) was added to a solution of phenol **26** (1.90 g, 2.82 mmol, 1 equiv) in tetrahydrofuran (12.0 mL) at 23 °C. The reaction vessel was placed into a high-pressure autoclave and exposed to hydrogen pressure of 50 bar. After 15 h, the hydrogen gas pressure was released and the reaction mixture was filtered through a pad of celite. The pad was washed with ethyl acetate (50 mL). The filtrate was concentrated, and the residue was purified by flash column chromatography on silica gel (20 to 30% ethyl acetate in petroleum ether) to give alcohol **27** (870 mg, 1.50 mmol, 53%) as a yellow solid.

Analytical data for alcohol **27**:

TLC (30% ethyl acetate in petroleum ether): R_f = 0.28 (UV, CAM).

^{1}H NMR (400 MHz, $CDCl_3$): δ = 9.79 (s, 1H), 7.34 (t, *J* = 8.0 Hz, 1H), 7.20 – 7.05 (m, 2H), 6.92 (d, *J* = 8.9 Hz, 2H), 5.26 (d, *J* = 1.4 Hz, 3H), 4.72 (d, *J* = 9.3 Hz, 1H), 4.31 – 4.19 (m, 1H), 3.89 (d, *J* = 9.3 Hz, 1H), 3.70 – 3.32 (m, 8H), 1.86 (t, *J* = 6.1 Hz, 1H), 1.78 (t, *J* = 6.2 Hz, 1H), 1.46 – 1.33 (m, 2H), 0.87 (s, 9H), 0.05 (s, 3H), 0.03 (s, 3H), OH not detected due to exchange events.

^{13}C NMR (101 MHz, $CDCl_3$): δ = 206.5, 182.7, 154.0, 152.9, 149.8, 130.9, 129.2, 124.0, 122.5, 122.5, 121.2, 119.9, 119.2, 114.4, 95.0, 75.4, 68.3, 63.2, 63.1, 56.5, 53.8, 52.8, 47.7, 34.9, 31.2, 25.8, 18.1, -4.2, -4.9.

IR (ATR, neat): 2956, 2929, 2856, 1765, 1736, 1700, 1602, 1574, 1474, 1436, 1297, 1247, 1197, 1155, 1127, 1046, 1023, 997, 940, 884, 859, 838, 776, 732, 674 cm^{-1}.

HRMS calc. for $C_{31}H_{41}O_9Si^+$ $[M+H]^+$: 585.2514; found: 585.2520.

Acetyl ester S10

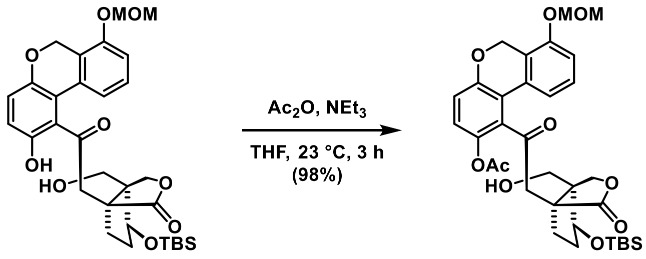

27 **S10**

Acetic anhydride (44.2 µL, 469 µmol, 1.10 equiv) and triethylamine (65.3 µL, 469 µmol, 1.10 equiv) were added to a solution of alcohol **27** (249 mg, 426 µmol, 1 equiv) in tetrahydrofuran (4.00 mL) at 23 °C. After 3 h, aqueous saturated sodium hydrogen carbonate solution (5 mL) and ethyl acetate (5 mL) were added to the reaction mixture. The layers were separated, and the aqueous phase was extracted with ethyl acetate (2 × 5 mL). The combined organic phases were dried over magnesium sulfate and the dried solution was filtered. The filtrate was concentrated under reduced pressure and the residue was purified by flash column chromatography on silica gel (20% ethyl acetate in petroleum ether) to give acetyl ester **S10** (262 mg, 418 µmol, 98%) as a pale yellow solid.

Analytical data for acetyl ester **S10**:

TLC (20% ethyl acetate in petroleum ether): R_f = 0.17 (UV, CAM).

^{1}H NMR (400 MHz, $CDCl_3$): δ = 7.33 (t, *J* = 8.1 Hz, 1H), 7.15 (dd, *J* = 8.4, 0.9 Hz, 1H), 7.09 (d, *J* = 8.9 Hz, 1H), 7.04 (d, *J* = 7.8 Hz, 1H), 6.99 (d, *J* = 8.8 Hz, 1H), 5.23 (s, 2H), 5.16 (s, 2H), 4.75 (d, *J* = 9.1 Hz, 1H), 4.43 – 4.27 (m, 1H), 4.01 (d, *J* = 9.1 Hz, 1H), 3.75 (dd, *J* = 10.8, 2.8 Hz, 1H), 3.57 (dd, *J* = 10.9, 2.3 Hz, 1H), 3.51 (s, 3H), 3.40 – 3.17 (m, 2H), 2.31 (s, 3H), 1.96 – 1.77 (m, 2H), 1.53 – 1.37 (m, 2H), 0.89 (s, 9H), 0.06 (s, 6H), OH not detected due to exchange events.

^{13}C NMR (101 MHz, $CDCl_3$): δ = 204.0, 182.1, 170.5, 153.8, 152.8, 141.5, 130.7, 129.5, 129.3, 124.4, 122.0, 121.5, 120.3, 119.6, 114.6, 94.9, 75.4, 68.4, 63.3, 62.8, 56.5, 53.8, 51.7, 48.7, 34.8, 31.3, 25.8, 21.0, 18.0, -4.2, -5.0.

IR (ATR, neat): 3472, 2927, 2856, 1765, 1701, 1593, 1573, 1440, 1369, 1252, 1189, 1154, 1127, 1003, 942, 887, 837, 778 cm^{-1}.

HRMS (ESI) calc. for $C_{33}H_{43}O_{10}Si$ $[M+H]^+$: 627.2620; found: 627.2616.

Aldehyde 28

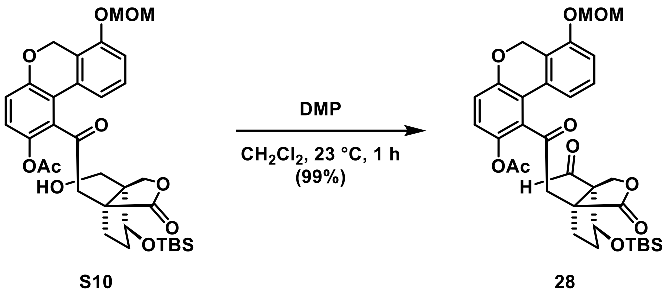

Dess–Martin periodinane (354 mg, 836 µmol, 2.00 equiv) was added to a solution of alcohol **S10** (262 mg, 418 µmol, 1 equiv) in dichloromethane (4.00 mL) at 23 °C. After 1 h, aqueous saturated sodium thiosulfate solution (5 mL) and dichloromethane (5 mL) were added to the reaction mixture. The layers were separated, and the aqueous phase was extracted with dichloromethane (2 × 5 mL). The combined organic phases were dried over magnesium sulfate and the dried solution was filtered. The filtrate was concentrated under reduced pressure and the residue was purified by flash column chromatography on silica gel (20 to 30% ethyl acetate in petroleum ether) to give aldehyde **28** (250 mg, 400 µmol, 99%) as a white solid.

Analytical data for aldehyde **28**:

TLC (20% ethyl acetate in petroleum ether): R_f = 0.24 (UV, CAM).

^{1}H NMR (400 MHz, $CDCl_3$): δ = 9.71 (s, 1H), 7.39 (t, *J* = 8.1 Hz, 1H), 7.15 (d, *J* = 8.4 Hz, 1H), 7.09 (dt, *J* = 8.9, 1.0 Hz, 1H), 6.97 (d, *J* = 8.9 Hz, 1H), 6.73 (d, *J* = 7.7 Hz, 1H), 5.27 – 5.20 (m, 2H), 5.20 – 5.07 (m, 2H), 4.98 (d, *J* = 2.3 Hz, 2H), 4.57 (dd, *J* = 10.5, 5.7 Hz, 1H), 3.51 (t, *J* = 1.1 Hz, 3H), 3.34 (d, *J* = 19.7 Hz, 1H), 3.03 (d, *J* = 19.8 Hz, 1H), 2.33 (s, 3H), 2.06 – 1.83 (m, 2H), 1.54 (m, 2H), 0.86 (s, 9H), 0.04 (s, 3H), -0.01 (s, 3H).

^{13}C NMR (101 MHz, $CDCl_3$): δ = 203.6, 201.8, 179.8, 170.3, 153.7, 152.7, 141.7, 129.9, 129.8, 129.0, 124.5, 121.6, 121.6, 120.7, 119.9, 114.7, 94.9, 78.2, 66.9, 63.3, 63.3, 56.4, 53.9, 48.3, 34.6, 31.8, 25.7, 20.9, 18.0, -4.5, -5.0.

IR (ATR, neat): 2928, 2856, 1768, 1721, 1593, 1440, 1371, 1253, 1185, 1154, 1127, 1085, 1037, 1003, 940, 884, 837, 77, 733, 673 cm^{-1}.

HRMS (ESI) calc. for $C_{33}H_{41}O_{10}Si$ $[M+H]^+$: 625.2464; found: 625.2461.

Phenol S11

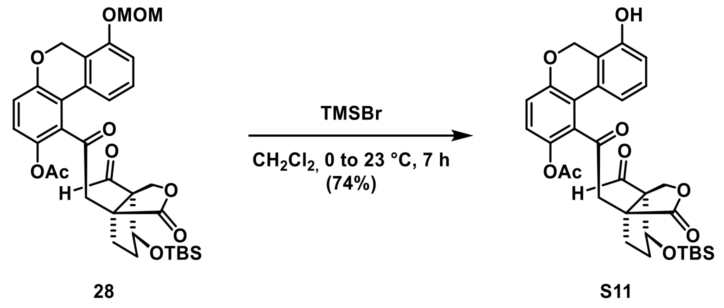

Note: The removal of the MOM protecting group was conducted in two parallel runs (each 132 µmol), which were combined for purification by column chromatography.

Bromotrimethylsilane (137 µL, 1.06 mmol, 8.00 equiv) was added to a solution of ether **28** (82.6 mg, 132 µmol, 1 equiv) in dichloromethane (1.60 mL) at 0 °C and was then warmed to 23 °C. After 7 h, water (10 mL) and dichloromethane (10 mL) were added to the reaction mixture. The layers were separated, and the aqueous phase was extracted with dichloromethane (2 × 5 mL). The combined organic phases were dried over magnesium sulfate and the dried solution was filtered. The filtrate was concentrated under reduced pressure and the combined residues from both reactions were purified by flash column chromatography on silica gel (30% ethyl acetate in petroleum ether) to give phenol **S11** (113 mg, 195 µmol, 74%) as a white solid.

Analytical data for phenol **S11**:

TLC (30% ethyl acetate in petroleum ether): R_f = 0.26 (UV, CAM).

^{1}H NMR (400 MHz, $CDCl_3$): δ = 9.71 (s, 1H), 7.30 (t, *J* = 8.1 Hz, 1H), 7.10 (d, *J* = 8.8 Hz, 1H), 6.98 (d, *J* = 8.8 Hz, 1H), 6.79 (d, *J* = 8.1 Hz, 1H), 6.67 (d, *J* = 7.7 Hz, 1H), 5.14 (s, 2H), 4.99 (d, *J* = 1.8 Hz, 2H), 4.93 (s, 1H), 4.57 (dd, *J* = 10.4, 5.6 Hz, 1H), 3.35 (d, *J* = 19.8 Hz, 1H), 3.03 (d, *J* = 19.8 Hz, 1H), 2.34 (s, 3H), 1.93 (m, 2H), 1.63 – 1.56 (m, 1H), 1.53 – 1.45 (m, 1H), 0.86 (s, 9H), 0.04 (s, 3H), -0.01 (s, 3H).

^{13}C NMR (101 MHz, $CDCl_3$): δ = 203.6, 201.9, 180.0, 170.3, 153.8, 150.9, 141.7, 129.8, 129.4, 124.6, 120.0, 120.0, 119.4, 116.0, 78.2, 67.0, 63.4, 63.2, 54.0, 48.4, 34.7, 31.9, 25.8, 22.8, 21.0, 18.0, 14.3, -4.5, -5.0.

IR (ATR, neat): 3394, 2926, 2855, 1765, 1746, 1723, 1596, 1446, 1378, 1253, 1188, 1133, 1037, 885, 839, 778, 674 cm^{-1}.

HRMS (ESI) calc. for $C_{31}H_{37}O_9Si$ $[M+H]^+$: 581.2201; found: 581.2199.

Alcohol 12

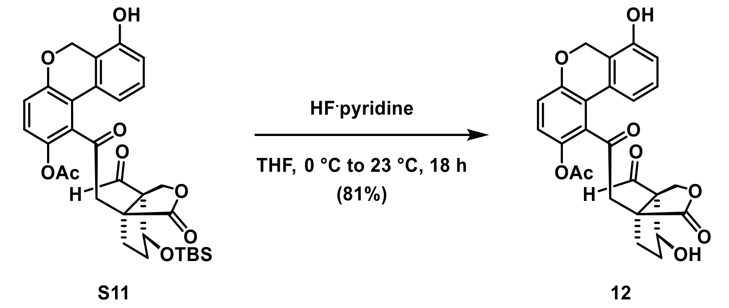

Pyridine hydrofluoride (≈70% hydrogen fluoride, ≈30% pyridine, 0.520 mL, 20.1 mmol, 300 equiv) was added to a solution of phenol **S11** (39.0 mg, 67.2 µmol, 1 equiv) in tetrahydrofuran (0.700 mL) at 0 °C, which was then warmed to 23 °C. After 18 h, aqueous saturated sodium hydrogen carbonate solution (20 mL) and ethyl acetate (10 mL) were added to the reaction mixture. The layers were separated, and the aqueous phase was extracted with ethyl acetate (2 × 5 mL). The combined organic phases were dried over magnesium sulfate and the dried solution was filtered. The filtrate was concentrated under reduced pressure and the residue was purified by flash column chromatography on silica gel (70% ethyl acetate in petroleum ether) to give alcohol **12** (25.5 mg, 54.7 µmol, 81%) as a yellow solid.

Analytical data for alcohol **12**:

TLC (80% ethyl acetate in petroleum ether): R_f = 0.46 (CAM).

^{1}H NMR (400 MHz, ($(CD_3)_2CO$)): δ = 9.72 (s, 1H), 7.24 (t, *J* = 8.0 Hz, 1H), 7.13 (d, *J* = 8.8 Hz, 1H), 7.09 (d, *J* = 8.8 Hz, 1H), 6.96 (dd, *J* = 8.2, 0.9 Hz, 1H), 6.65 (dd, *J* = 7.7, 0.9 Hz, 1H), 5.20 – 5.04 (m, 2H), 4.97 (d, *J* = 9.6 Hz, 1H), 4.83 (d, *J* = 9.6 Hz, 1H), 4.67 (dd, *J* = 11.2, 5.9 Hz, 1H), 3.41 (d, *J* = 20.1 Hz, 1H), 3.31 (d, *J* = 20.1 Hz, 1H), 2.27 (s, 3H), 2.03 – 1.94 (m, 1H), 1.90 – 1.71 (m, 2H), 1.58 – 1.40 (m, 1H), OH not detected due to exchange events.

^{13}C NMR (101 MHz, ($(CD_3)_2CO$)): δ = 204.1, 201.7, 180.6, 170.2, 154.4, 153.4, 142.8, 131.0, 130.3, 130.0, 125.5, 122.3, 120.2, 120.1, 119.1, 116.6, 76.6, 66.2, 63.8, 63.5, 54.4, 49.0, 35.3, 32.0, 20.8.

IR (ATR, neat): 3369, 2923, 2853, 2360, 1763, 1595, 1458, 1458, 1376, 1195, 1026, 800, 726 cm^{-1}.

HRMS (ESI) calc. for $C_{25}H_{23}O_9$ $[M+H]^+$: 467.1337; found: 467.1337.

Quinone 29

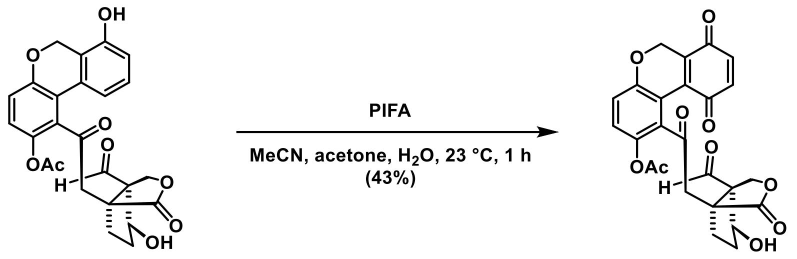

12 29

(Bis(trifluoroacetoxy)iodo)benzene (51.7 mg, 0.120 mmol, 2.20 equiv) was added to a solution of phenol **12** (25.5 mg, 54.7 µmol, 1 equiv) in a mixture of acetonitrile, acetone and water (1:1:1, 600 µL) at 23 °C. After 1 h, aqueous saturated sodium bicarbonate solution (5 mL) and ethyl acetate (5 mL) were added to the red reaction mixture. The layers were separated, and the aqueous phase was extracted with ethyl acetate (2 × 5 mL). The combined organic phases were dried over magnesium sulfate and the dried solution was filtered. The filtrate was concentrated under reduced pressure and the residue was purified by flash column chromatography on silica gel (60 to 70% ethyl acetate in petroleum ether) to give quinone **29** (11.3 mg, 23.5 µmol, 43%) as a red oil.

Analytical data for quinone **29**:

TLC (60% ethyl acetate in petroleum ether): R_f = 0.28 (UV, CAM).

^{1}H NMR (400 MHz, $CDCl_3$): δ = 9.56 (s, 1H), 7.18 (d, *J* = 9.0 Hz, 1H), 7.13 (d, *J* = 9.0 Hz, 1H), 6.87 (d, *J* = 10.1 Hz, 1H), 6.82 (d, *J* = 10.2 Hz, 1H), 5.01 (d, *J* = 14.5 Hz, 1H), 4.86 – 4.76 (m, 3H), 4.73 (dt, *J* = 10.9, 5.4 Hz, 1H), 4.08 (d, *J* = 19.6 Hz, 1H), 3.67 (d, *J* = 19.6 Hz, 1H), 2.47 (s, 3H), 2.20 (dd, *J* = 13.2, 6.5 Hz, 1H), 2.13 – 2.06 (m, 1H), 1.90 – 1.76 (m, 2H), 1.65 (tt, *J* = 12.1, 6.1 Hz, 1H).

^{13}C NMR (101 MHz, $CDCl_3$): δ = 201.3, 201.2, 185.1, 183.9, 180.3, 169.4, 155.8, 142.8, 137.2, 136.3, 134.8, 133.3, 131.6, 127.8, 121.1, 116.8, 76.2, 66.0, 62.9, 61.4, 54.8, 47.6, 35.0, 31.0, 21.5.

IR (ATR, neat): 3497, 2925, 1763, 1719, 1655, 1596, 1463, 1395, 1369, 1351, 1317, 1243, 1186, 1160, 1075, 1046, 883, 838, 735 cm^{-1}.

HRMS (ESI) calc. for $C_{25}H_{20}O_{10}Na^+$ $[M+Na]^+$: 503.0949; found: 503.0967.

Phenol 11

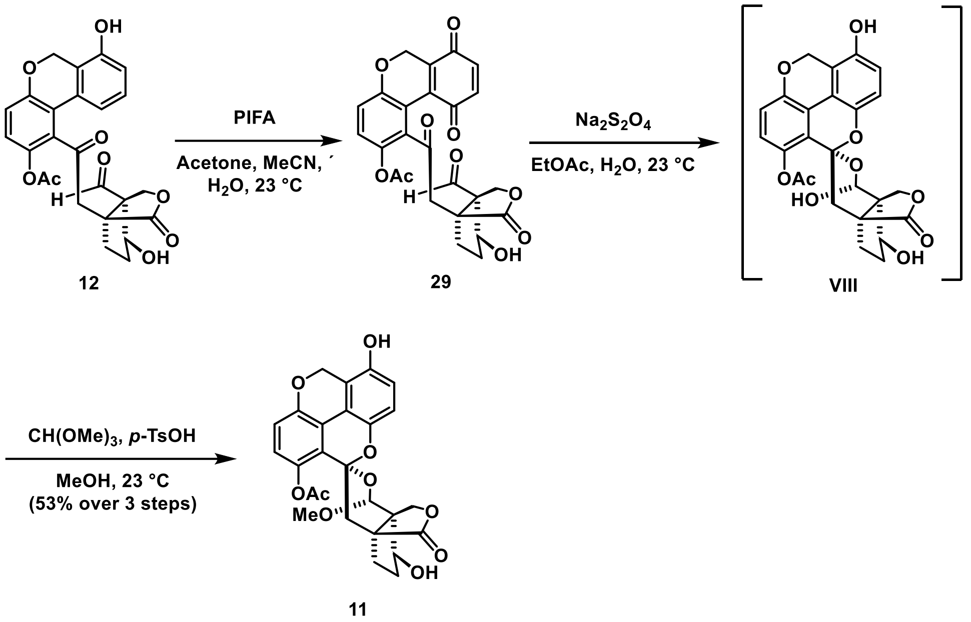

(Bis(trifluoroacetoxy)iodo)benzene (24.3 mg, 56.6 µmol, 2.20 equiv) was added to a solution of phenol **12** (12.0 mg, 25.7 µmol, 1 equiv) in a mixture of acetonitrile, acetone and water (1:1:1, 0.300 mL). After 4 h, aqueous saturated sodium bicarbonate solution (5 mL) and ethyl acetate (5 mL) were added to the red reaction mixture. The layers were separated, and the aqueous phase was extracted with ethyl acetate (2 × 5 mL). The combined organic phases were dried over magnesium sulfate and the dried solution was filtered. The filtrate was concentrated under reduced pressure to give crude quinone **29,** which was used in the next step without further purification.

Sodium dithionite (22.4 mg, 129 µmol, 5.00 equiv) was added to a solution of crude quinone **29** (assumed pure 12.4 mg, 25.7 µmol, 1 equiv) in ethyl acetate and water (1:1, 0.500 mL) at 23 °C. After 1 h, aqueous saturated sodium bicarbonate solution (5 mL) and ethyl acetate (5 mL) were added to the reaction mixture. The layers were separated, and the aqueous phase was extracted with ethyl acetate (2 × 5 mL). The combined organic phases were dried over magnesium sulfate and the dried solution was filtered. The filtrate was concentrated under reduced pressure to give a crude product (presumably phenol **VIII**).

p-Toluenesulfonic acid monohydrate (489 µg, 2.57 µmol, 0.100 equiv) was added to a solution of the crude phenol **VIII** (assumed pure 12.4 mg, 25.7 µmol, 1 equiv) in a mixture of methanol and trimethyl orthformate (1:1, 0.500 mL). After 4 h, water (5 mL) and ethyl acetate (5 mL) were added to the reaction mixture. The layers were separated, and the aqueous phase was extracted with ethyl acetate (2 × 5 mL). The combined organic phases were dried over magnesium sulfate and the dried solution was filtered. The filtrate was concentrated under reduced pressure and the residue was purified by flash column chromatography on silica gel (70 to 80% ethyl acetate in petroleum ether) to give phenol **11** (6.70 mg, 14.0 µmol, 53% over 3 steps) as a pale yellow solid.

Analytical data for Phenol **11**:

TLC (70% ethyl acetate in petroleum ether): R_f = 0.29 (UV, CAM).

^{1}H NMR (400 MHz, (($CD_3)_2CO$)): δ = 8.48 (s, 1H), 7.04 (d, *J* = 8.8 Hz, 1H), 6.98 (d, *J* = 8.8 Hz, 1H), 6.86 (d, *J* = 8.7 Hz, 1H), 6.71 (d, *J* = 8.7 Hz, 1H), 5.50 (d, *J* = 13.9 Hz, 1H), 5.10 (dd, *J* = 13.9, 0.8 Hz, 1H), 4.89 (d, *J* = 9.0 Hz, 1H), 4.72 (ddd, *J* = 9.0, 6.2, 4.5 Hz, 1H), 4.67 (s, 1H), 4.40 (d, *J* = 4.5 Hz, 1H), 4.03 (d, *J* = 9.1 Hz, 1H), 3.27 (s, 3H), 3.22 (d, *J* = 13.8 Hz, 1H), 2.71 (d, *J* = 13.7 Hz, 1H), 2.35 (s, 3H), 2.21 (ddt, *J* = 12.4, 5.5, 2.8 Hz, 1H), 2.11 (dd, *J* = 6.2, 2.7 Hz, 1H), 1.70 (ddd, *J* = 12.6, 11.0, 6.3 Hz, 1H), 1.58 – 1.45 (m, 1H).

^{13}C NMR (101 MHz, (($CD_3)_2CO$)): δ = 181.7, 168.7, 149.2, 147.1, 140.3, 139.8, 125.3, 122.5, 116.9, 116.8, 115.7, 115.3, 114.7, 114.3, 100.4, 97.0, 74.4, 66.2, 62.9, 55.8, 53.6, 48.5, 36.6, 35.9, 32.2, 20.4.

IR (ATR, neat): 3279, 2965, 2851, 1764, 1743, 1698, 1462, 1436, 1698, 1462, 1436, 1370, 1317, 1246, 1200, 1155, 1099, 1021, 978, 960, 854, 817 cm^{-1}.

HRMS (ESI) calc. for $C_{26}H_{25}O_{10}^{+}$ $[M+H]^{+}$: 497.1442; found: 497.1432.

Diastereoselectivity of the spiro bis-acetalization

A possible explanation for observed diastereoselectivity is the anomeric stabilization of the formed spiro bis-acetal. The reaction should be under thermodynamic control and provides the diastereomer for which anomeric stabilization is possible. The second acetal formation allows for additional anomeric stabilization (an axial-axial alignment for the spiro-acetal has two anomeric stabilizations). For clarity, we have shown the possible anomeric effects for ganoapplanin and **VIII**.

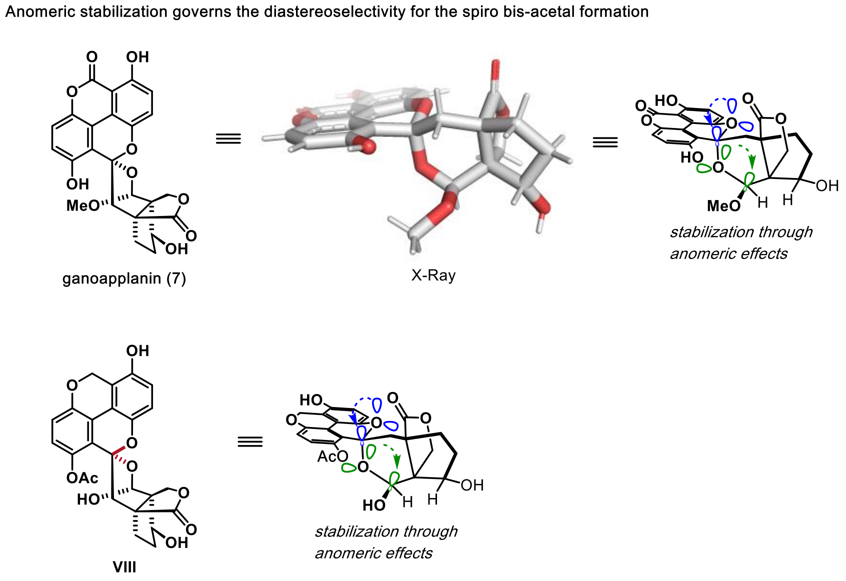

Figure 1: Anomeric stabilization of ganoapplanin (**7**) and **VIII**.

Acetyl ester 30

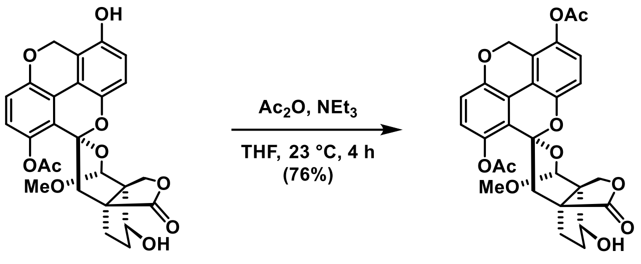

A solution of acetic anhydride (0.59 µL, 6.2 µmol, 1.1 equiv) in tetrahydrofuran (0.10 mL) and a solution of triethylamine (0.86 µL, 6.2 µmol, 1.1 equiv) in tetrahydrofuran (0.10 mL) were added to a solution of phenol **11** (2.8 mg, 5.6 µmol, 1 equiv) in tetrahydrofuran (0.30 mL) at 23 °C. After 4 h, aqueous saturated sodium hydrogen carbonate solution (3 mL) and ethyl acetate (3 mL) were added to the reaction mixture. The layers were separated, and the aqueous phase was extracted with ethyl acetate (2 × 3 mL). The combined organic phases were dried over magnesium sulfate and the dried solution was filtered. The filtrate was concentrated under reduced pressure and the residue was purified by flash column chromatography on silica gel (60% ethyl acetate in petroleum ether) to give acetyl ester **30** (2.3 mg, 4.3 µmol, 76%) as a white solid.

Analytical data for acetyl ester **30**:

TLC (60% ethyl acetate in petroleum ether): R_f = 0.25 (UV, CAM).

^{1}H NMR (400 MHz, $CDCl_3$): δ = 6.99 – 6.90 (m, 3H), 6.83 (d, *J* = 8.8 Hz, 1H), 5.15 – 5.11 (m, 2H), 5.08 (d, *J* = 13.7 Hz, 1H), 4.84 (td, *J* = 8.3, 2.7 Hz, 1H), 4.67 (d, *J* = 10.0 Hz, 1H), 4.17 (d, *J* = 10.0 Hz, 1H), 3.48 (s, 3H), 2.78 (d, *J* = 14.4 Hz, 1H), 2.33 (s, 3H), 2.31 (s, 3H), 2.31 – 2.22 (m, 3H), 1.89 – 1.78 (m, 1H), 1.69 (dt, J = 13.9, 8.3 Hz, 1H), OH not detected due to exchange events.

^{13}C NMR (101 MHz, $CDCl_3$): δ = 179.9, 169.6, 169.1, 149.2, 144.7, 140.4, 140.1, 125.5, 123.6, 121.8, 120.1, 117.9, 116.8, 115.4, 114.2, 100.5, 100.3, 74.2, 68.0, 63.4, 57.6, 54.0, 48.6, 36.3, 34.9, 30.7, 21.2, 20.9.

IR (ATR, neat): 2922, 2852, 1765, 1618, 1457, 1370, 1260, 1191, 1150, 1099, 1027, 961, 904, 854 cm^{-1}.

HRMS (ESI) calc. for $C_{28}H_{27}O_{11}^+$ $[M+H]^+$: 539.1548; found: 539.1545.

Lactone 31

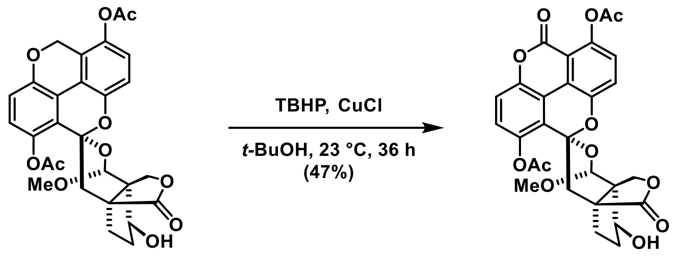

30 31

A solution of *tert*-butyl hydroperoxide (5.5 M in decane 16 µL, 85 µmol, 20 equiv) and copper(I) chloride (0.42 mg, 4.3 µmol, 1.0 equiv) were added to a solution of cyclic ether **30** (2.3 mg, 4.3 µmol, 1 equiv) in *tert*-butanol (0.30 mL) at 23 °C. After 36 h, the reaction mixture was filtered through a plug of celite. Water (1 mL) and ethyl acetate (1 mL) were added to the filtrate, the layers were separated, and the aqueous phase was extracted with ethyl acetate (2 × 1 mL). The combined organic phases were dried over magnesium sulfate and the dried solution was filtered. The filtrate was concentrated under reduced pressure and the residue was purified by flash column chromatography on silica gel (80% ethyl acetate in petroleum ether) to give lactone **31** (1.1 mg, 2.0 µmol, 47%) as a white solid.

Analytical data for lactone **31**:

TLC (80% ethyl acetate in petroleum ether): R_f = 0.31 (UV, CAM).

^{1}H NMR (700 MHz, $CDCl_3$): δ = 7.37 (d, *J* = 8.9 Hz, 1H), 7.28 (d, *J* = 8.7 Hz, 1H), 7.22 (d, *J* = 8.9 Hz, 1H), 7.19 (d, *J* = 8.7 Hz, 1H), 5.18 (s, 1H), 4.87 (td, *J* = 8.4, 2.6 Hz, 1H), 4.70 (d, *J* = 10.1 Hz, 1H), 4.20 (d, *J* = 10.0 Hz, 1H), 3.50 (s, 3H), 2.77 (d, *J* = 14.3 Hz, 1H), 2.43 (s, 3H), 2.37 (s, 3H), 2.35 – 2.24 (m, 4H), 1.89 (ddt, *J* = 13.4, 9.8, 8.1 Hz, 1H), 1.71 (dt, *J* = 14.1, 8.2 Hz, 1H), OH not detected due to exchange events.

^{13}C NMR (176 MHz, $CDCl_3$): δ = 179.7, 169.9, 169.2, 156.6, 147.3, 146.3, 145.3, 142.5, 127.2, 125.5, 123.3, 121.8, 119.9, 118.3, 112.4, 112.0, 100.8, 100.7, 74.1, 68.2, 57.8, 54.1, 48.6, 37.0, 34.6, 30.5, 21.2, 21.1.

IR (ATR, neat): 2922, 2852, 1769, 1749, 1454, 1370, 1189, 1027, 957, 908, 856 cm^{-1}.

HRMS (ESI) calc. for $C_{28}H_{25}O_{12}^+$ [M+H]$^+$: 553.1341; found: 553.1333.

Ganoapplanin (7)

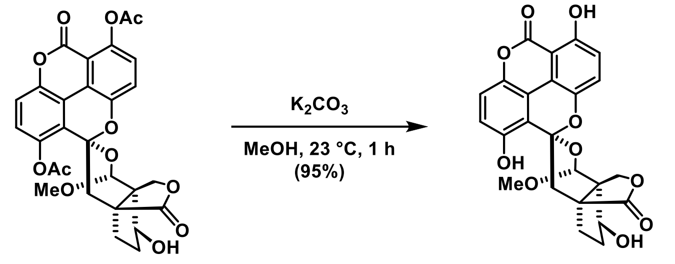

31 7

Potassium carbonate (1.1 mg, 8.0 µmol, 4.0 equiv) was added to a solution of lactone **31** (1.1 mg, 2.0 µmol, 1 equiv) in methanol (0.30 mL) at 23 °C. After 1 h, water (3 mL) and ethyl acetate (3 mL) were added to the red reaction mixture. The layers were separated, and the aqueous phase was extracted with ethyl acetate (2 × 3 mL). The combined organic phases were dried over magnesium sulfate and the dried solution was filtered. The filtrate was concentrated under reduced pressure and the residue was purified by flash column chromatography on silica gel (60 to 70% ethyl acetate in petroleum ether) to give ganoapplanin (**7**) (0.9 mg, 1.9 µmol, 95%) as a white solid.

Analytical data for ganoapplanin (**7**):

TLC (70% ethyl acetate in petroleum ether): R_f = 0.25 (UV, CAM).

^{1}H NMR (600 MHz, C_5D_5N): δ = 7.46 (d, *J* = 8.8 Hz, 1H), 7.29 (d, *J* = 8.9 Hz, 1H), 7.26 (d, *J* = 8.9 Hz, 1H), 7.13 (d, *J* = 8.8 Hz, 1H), 5.52 (d, *J* = 8.9 Hz, 1H), 5.14 – 5.09 (m, 1H), 4.89 (s, 1H), 4.47 (d, *J* = 5.7 Hz, 1H), 4.46 (d, *J* = 10.9 Hz, 1H), 3.36 (s, 3H), 3.29 (d, *J* = 14.0 Hz, 1H), 2.54 – 2.51 (m, 1H), 2.21 – 2.16 (m, 1H), 1.90 – 1.85 (m, 1H), 1.85 – 1.80 (m, 1H), OH not detected due to exchange events.

^{1}H NMR (600 MHz, $CDCl_3$): δ = 10.20 (s, 1H), 7.44 (s, 1H), 7.29 (d, *J* = 9.1 Hz, 1H), 7.20 (d, *J* = 9.0 Hz, 1H), 7.06 (d, *J* = 9.0 Hz, 1H), 7.02 (d, *J* = 9.0 Hz, 1H), 5.49 (s, 1H), 4.86 (d, *J* = 2.5 Hz, 1H), 4.66 (d, *J* = 10.3 Hz, 1H), 4.27 (d, *J* = 10.3 Hz, 1H), 3.72 (s, 3H), 2.71 (d, *J* = 14.9 Hz, 1H), 2.40 – 2.30 (m, 2H), 2.21 (ddd, *J* = 14.5, 11.0, 5.3 Hz, 1H), 1.93 (ddt, *J* = 14.5, 11.0, 7.4 Hz, 1H), 1.80 (d, *J* = 14.9 Hz, 1H), 1.70 – 1.64 (m, 1H).

Note: Due to the low solubility of ganoapplanin in chloroform, we were unable to measure a high quality ^{13}C spectrum.

^{13}C NMR (151 MHz, C_5D_5N): δ = 184.0, 164.5, 156.3, 152.0, 142.7, 140.6, 124.4, 120.0, 119.6, 118.0, 117.5, 116.6, 113.8, 104.5, 100.8, 99.0, 75.0, 67.5, 55.8, 54.4, 49.4, 37.7, 36.7, 33.5.

IR (ATR, neat): 2924, 2853, 1752, 1686, 1634, 1456, 1377, 1262, 1200, 1096, 1024, 963, 899, 857, 799 cm^{-1}.

HRMS (ESI) calc. for $C_{24}H_{21}O_{10}$ $[M+H]^+$: 469.1129; found: 469.1121.

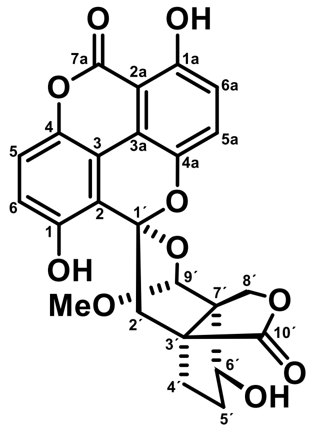

Table 2: Comparison of ^{1}H–NMR shifts in C_5D_5N for natural and synthetic ganoapplanin (**7**).

No	^{1}H–NMR (800 MHz, C_5D_5N) isolated ganoapplanin **ppm**	^{1}H–NMR (600 MHz, C_5D_5N) synthetic ganoapplanin **ppm**	Δ ppm
H-5a	7.46 (d, *J* = 8.8 Hz, 1H)	7.46 (d, *J* = 8.8 Hz, 1H)	±0.00
H-6a	7.13 (d, *J* = 8.8 Hz, 1H)	7.13 (d, *J* = 8.8 Hz, 1H)	±0.00
H-5	7.27 (d, *J* = 8.9 Hz, 1H)	7.26 (d, *J* = 8.9 Hz, 1H)	–0.01
H-6	7.28 (d, *J* = 8.9 Hz, 1H)	7.29 (d, *J* = 8.9 Hz, 1H)	+0.01
H-2´	3.30 (d, *J* = 14 Hz, 1H), 4.47 (d, *J* = 11.4 Hz, 1H)	3.29 (d, *J* = 14.0 Hz, 1H), 4.46 (d, *J* = 10.9 Hz, 1H)	–0.01, –0.01
H-4´	2.54 (m, 1H), 1.87 (m, 1H)	2.53 (m, 1H), 1.87 (m, 1H)	–0.01, ± 0.00
H-5´	2.20 (m, 1H), 1.86 (m, 1H)	2.21 (m, 1H), 1.86 (m, 1H)	+0.01, ± 0.00
H-6´	5.12 (dd, *J* = 9.1, 6.1 Hz, 1H)	5.11 (m, 1H)	–0.01
H-8´	5.53 (d, *J* = 9.0 Hz, 1H), 4.48 (d, *J* = 9.3 Hz, 1H)	5.52 (d, *J* = 8.9 Hz, 1H), 4.47 (d, *J* = 5.7 Hz, 1H)	–0.01, –0.01
H-9´	4.89 (s, 1H)	4.89 (s, 1H)	± 0.00
OCH_3	3.36 (s, 3H)	3.36 (s, 3H)	± 0.00
OH	10.84 (brs)	-	-
OH	12.59 (brs)	-	-

Table 3: Comparison of ^{13}C–NMR shifts in C_5D_5N for natural and synthetic ganoapplanin (**7**).

No	^{13}C–NMR (200 MHz, C_5D_5N) isolated ganoapplanin **ppm**	^{13}C–NMR (151 MHz, C_5D_5N) synthetic ganoapplanin **ppm**	Δ ppm
C-1a	156.2	156.3	+0.01
C-2a	104.4	104.5	+0.01
C-3a	119.6	119.6	±0.00
C-4a	140.6	140.6	±0.00
C-5a	124.4	124.4	±0.00
C-6a	117.4	117.5	+0.01
C-7a	164.4	164.5	+0.01
C-1	152.0	152.0	±0.00
C-2	113.7	113.8	+0.01
C-3	116.5	116.6	+0.01
C-4	142.6	142.7	+0.01
C-5	118.0	118.0	±0.00
C-6	120.0	120.0	±0.00
C-1´	98.9	99.0	+0.01
C-2´	37.6	37.7	+0.01

C-3´	54.3	54.4	+0.01
C-4´	36.6	36.7	+0.01
C-5´	33.4	33.5	+0.01
C-6´	74.9	75.0	+0.01
C-7´	49.3	49.4	+0.01
C-8´	67.5	67.5	±0.00
C-9´	100.7	100.8	+0.01
C-10´	184.0	184.0	±0.00
OCH_3	55.8	55.8	±0.00

3. Additional studies towards the radical-addition/aldol reaction cascade

3.1. Screened conditions for the intramolecular radical-addition of quinone monoacetal 14 and the radical addition/aldol reaction cascade

Table 4: Screened conditions.

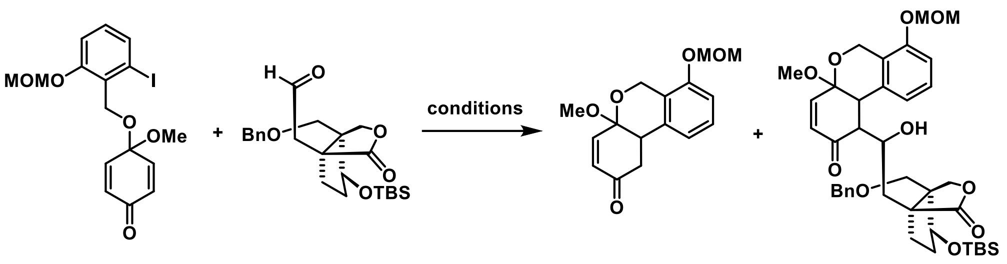

Entry	14	15	Reagents	Temp.	Result
**1**	1 equiv	-	*t*-BuLi (2.2 equiv), THF	–78 °C	Decomposition
**2**	1 equiv	-	*t*-BuLi (2.2 equiv), DMPU, THF	–78 °C	Decomposition
**3**	1 equiv	-	*t*-BuLi (2.2 equiv), HMPA, THF	–78 °C	Decomposition
**4**	1 equiv	-	*i*PrMgCl (1.1 equiv), THF	0 °C	Decomposition
**5**	1 equiv	-	AIBN (0.5 equiv), $HSnBu_3$ (1.2 equiv), toluene	50 °C	**S11** (70%)
**6**	1.6 equiv	1 equiv	AIBN (0.5 equiv), $HSnBu_3$ (1.2 equiv), toluene	50 °C	**S11** (70%), **13** not formed
**7**	1.6 equiv	1 equiv	AIBN (0.5 equiv), $HSnBu_3$ (1.2 equiv), BEt_3 (2.0 equiv), toluene	50 °C	Decomposition
**8**	1 equiv	-	BEt_3 (4.0 equiv), $HSnBu_3$ (1.2 equiv), air, toluene	–50 °C	**S11** (up to 90%)
**9**	1 equiv	-	BEt_3 (4.0 equiv), air, toluene	–50 °C	Decomposition
**10**	1 equiv	-	BEt_3 (4.0 equiv), air, benzene	23 °C	Decomposition
**11**	1 equiv	-	$HSnBu_3$ (1.2 equiv), air, toluene	–50 °C	No consumption of **14**
**12**	1.6 equiv	1 equiv	BEt_3 (4.0 equiv), $HSnBu_3$ (1.2 equiv), air, toluene	–50 °C	**13** (up to 81%), + **S11**

3.2. Synthesis and application of quinone monoacetal S20

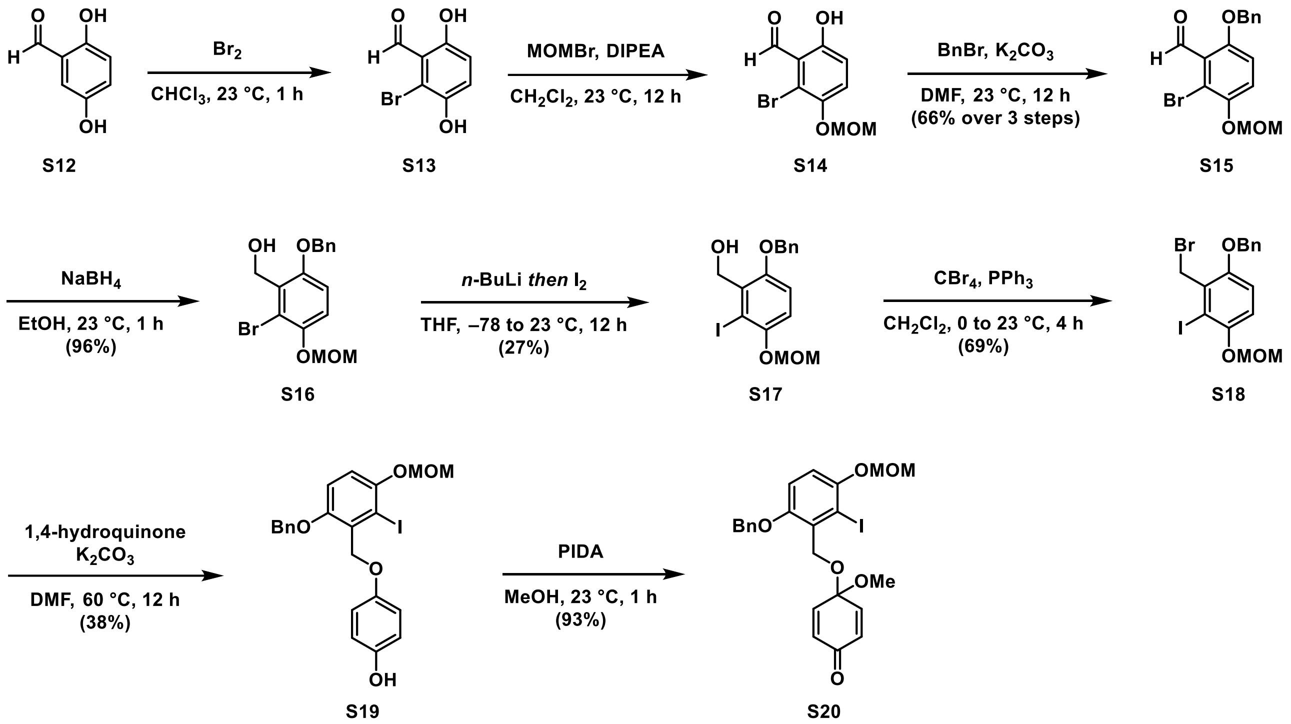

Scheme 2: Synthetic route towards quinone monoacetal **S20**.

Aryl bromide S13

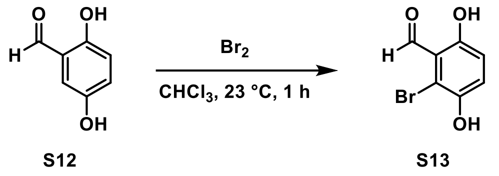

Bromine (7.83 mL, 152 mmol, 1.05 equiv) was added slowly to a solution of 2,5-dihydroxybenzaldehyde **S12** (20.0 g, 145 mmol, 1 equiv) in chloroform (600 mL) at 23 °C. After 1 h, aqueous saturated sodium thiosulfate solution (600 mL) was added to the reaction mixture. The layers were separated, and the aqueous phase was extracted with dichloromethane (2 × 300 mL). The combined organic phases were dried over magnesium sulfate and the dried solution was filtered. The filtrate was concentrated under reduced pressure to give crude aryl bromide **S13** (30.7 g) as a yellow solid, which was used in the next step without further purification.

<u>Analytical data for benzyl bromide **S13**:</u>

^{1}H NMR (400 MHz, $CDCl_3$): δ = 11.64 (s, 1H), 10.27 (s, 1H), 7.24 (d, *J* = 9.1 Hz, 1H), 6.92 (d, *J* = 9.1 Hz, 1H), 5.39 (s, 1H).

The obtained analytical data were in full agreement with those reported in the literature.[9]

MOM-ether S14

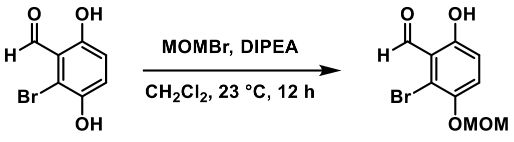

Bromomethyl methyl ether **S13** (11.5 mL, 141 mmol, 1.00 equiv) and *N*,*N*-diisopropylethylamine (27.0 mL, 156 mmol, 1.10 equiv) were added to a solution of benzyl bromide (assumed pure 30.7 g, 141 mmol, 1 equiv) in dichloromethane (600 mL) at 23 °C. After 12 h, water (600 mL) was added to the reaction mixture. The layers were separated, and the aqueous phase was extracted with dichloromethane (2 × 300 mL). The combined organic phases were dried over magnesium sulfate and the dried solution was filtered. The filtrate was concentrated under reduced pressure to give crude MOM-ether **S14** (32.2 g) as a yellow solid, which was used in the next step without further purification.

Benzyl ether S15

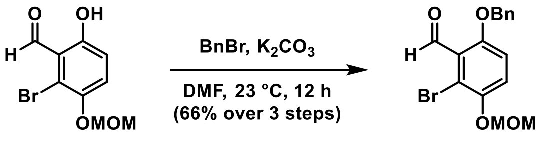

Benzyl bromide (17.6 mL, 148 mmol, 1.20 equiv) and potassium carbonate (34.1 g, 247 mmol, 2.00 equiv) were added to a solution of MOM-ether **S14** (assumed pure 32.2 g, 124 mmol, 1 equiv) in *N*,*N*-dimethylformamide (600 mL) at 23 °C. After 12 h, water (600 mL) was added to the reaction mixture. The layers were separated, and the aqueous phase was extracted with ethyl acetate (2 × 300 mL). The combined organic phases were washed with water (2 × 300 mL) and the washed layers were dried over magnesium sulfate and the dried solution was filtered. The filtrate was concentrated under reduced pressure and the residue was purified by flash column chromatography on silica gel (30% ethyl acetate in petroleum ether) to give benzyl ether **S15** (33.7 g, 96.4 mmol, 66% over 3 steps) as a yellow solid.

Analytical data for benzyl ether **S15**:

TLC (30% ethyl acetate in petroleum ether): R_f = 0.32 (UV, CAM).

^{1}H NMR (400 MHz, $CDCl_3$): δ = 10.46 (s, 1H), 7.42 – 7.32 (m, 6H), 6.95 (d, *J* = 9.2 Hz, 1H), 5.19 (s, 2H), 5.15 (s, 2H), 3.53 (s, 3H).

^{13}C NMR (101 MHz, $CDCl_3$): δ = 190.6, 157.4, 153.6, 136.1, 131.4, 128.9, 128.4, 127.3, 122.0, 115.7, 113.6, 96.2, 71.7, 56.7.

IR (ATR, neat): 2978, 2925, 2876, 1764, 1750, 1388, 1259, 1210, 1129, 1028, 869, 844, 753, 512 cm^{-1}.

HRMS (ESI) calc. for $C_{16}H_{15}BrO_4Na^+$ $[M+Na]^+$: 373.0046; found: 373.0028.

Benzylic alcohol S16

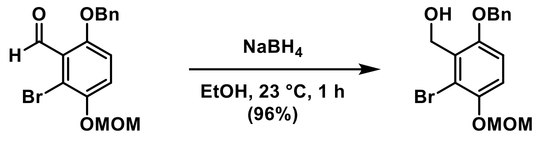

S15 **S16**

Sodium borohydride (4.04 g, 107 mmol, 1.50 equiv) was added to a solution of benzaldehyde **S15** (25.0 g, 71.2 mmol, 1 equiv) in ethanol (355 mL) at 23 °C. After 1 h, water (300 mL) was added to the reaction mixture and the reaction mixture was concentrated under reduced pressure. Water (300 mL) and dichloromethane (300 mL) was added to the residue and the layers were separated and the aqueous phase was extracted with dichloromethane (2 × 500 mL). The combined organic phases were dried over magnesium sulfate and the dried solution was filtered. The filtrate was concentrated under reduced pressure and the residue was purified by flash column chromatography on silica gel (40% ethyl acetate in petroleum ether) to give benzylic alcohol **S16** (24.3 g, 68.9 mmol, 96%) as a yellow solid.

Analytical data for benzylic alcohol **S16**:

TLC (40% ethyl acetate in petroleum ether): R_f = 0.25 (UV, CAM).

^{1}H NMR (400 MHz, $CDCl_3$): δ = 7.44 – 7.31 (m, 5H), 7.06 (d, *J* = 9.0 Hz, 1H), 6.87 (d, *J* = 9.0 Hz, 1H), 5.17 (s, 2H), 5.09 (s, 2H), 4.96 (d, *J* = 6.7 Hz, 2H), 3.52 (s, 3H), 2.43 (t, *J* = 6.9 Hz, 1H).

^{13}C NMR (101 MHz, $CDCl_3$): δ = 152.9, 148.5, 136.5, 130.5, 128.9, 128.4, 127.5, 116.8, 116.7, 112.2, 96.0, 71.4, 60.8, 56.5.

IR (ATR, neat): 3348, 2939, 2836, 1601, 1587, 1489, 1457, 1436, 1263, 1154, 1039, 783, 738 cm^{-1}.

HRMS (ESI) calc. for $C_{16}H_{17}BrO_4Na^+$ $[M+Na]^+$: 375.0202; found: 375.0192.

Benzyl iodide S17

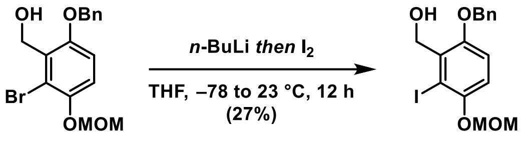

S16 S17

A solution of *n*-Butyllithium in hexanes (68.9 mL, 2.50 M in hexane, 172 mmol, 2.50 equiv) was added dropwise to a solution of aryl bromide **S16** (24.3 g, 68.9 mmol, 1 equiv) in tetrahydrofuran (340 mL) at –78 °C. After 1 h, a solution of iodine (52.4 g, 207 mmol, 3.00 equiv) in tetrahydrofuran (300 mL) was added slowly at –78 °C and the resulting solution was warmed to 23 °C. After 12 h, aqueous saturated sodium thiosulfate solution (500 mL) and dichloromethane (300 mL) were added to the reaction mixture. The layers were separated, and the aqueous phase was extracted with dichloromethane (2 × 300 mL). The combined organic phases were dried over magnesium sulfate and the dried solution was filtered. The filtrate was concentrated under reduced pressure and the residue was purified by flash column chromatography on silica gel (40% ethyl acetate in petroleum ether) to give benzyl iodide **S17** (7.46 g, 18.6 mmol, 27%) as a yellow oil.

Analytical data for benzyl iodide **S17**:

TLC (40% ethyl acetate in petroleum ether): R_f = 0.25 (UV, CAM).

^{1}H NMR (400 MHz, $CDCl_3$): δ = 7.44 – 7.31 (m, 5H), 6.99 (d, *J* = 9.0 Hz, 1H), 6.89 (d, *J* = 9.0 Hz, 1H), 5.17 (s, 2H), 5.09 (s, 2H), 4.97 (d, *J* = 7.0 Hz, 2H), 3.52 (s, 3H), 2.42 (t, *J* = 7.1 Hz, 1H).

^{13}C NMR (101 MHz, $CDCl_3$): δ = 152.4, 150.8, 136.6, 133.7, 128.9, 128.4, 127.5, 115.5, 113.4, 95.9, 95.8, 71.5, 65.6, 56.6.

IR (ATR, neat): 3486, 1589, 1574, 1468, 1442, 1329, 1289, 1244, 1194, 1173, 1110, 1015, 826, 748 cm^{-1}.

HRMS (ESI) calc. for $C_{16}H_{17}IO_4Na^+$ $[M+Na]^+$: 423.0064; found: 423.0050.

Benzyl bromide S18

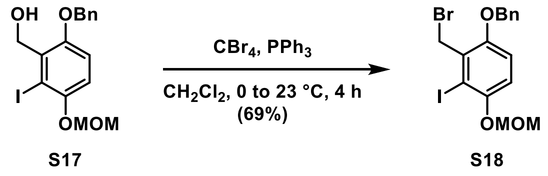

Carbon tetrabromide (1.82 g, 5.50 mmol, 1.10 equiv) and triphenylphosphine (1.57 g, 6.00 mmol, 1.20 equiv) were added to a solution of benzylic alcohol **S17** (2.00 g, 5.00 mmol, 1 equiv) in dichloromethane (50 mL) at 0 °C. After 30 min, the reaction mixture was allowed to warm to 23 °C. After 4 h, the reaction mixture was concentrated under reduced pressure and the residue was purified by flash column chromatography on silica gel (0 to 5% ethyl acetate in petroleum ether) to give benzyl bromide **S18** (1.61 g, 3.48 mmol, 69%) as a white solid.

Analytical data for benzyl bromide **S18**:

TLC (5% ethyl acetate in petroleum ether): R_f = 0.28 (UV, CAM).

^{1}H NMR (400 MHz, $CDCl_3$): δ = 7.48 (m, 2H), 7.43 – 7.37 (m, 2H), 7.37 – 7.33 (m, 1H), 7.00 (d, *J* = 9.0 Hz, 1H), 6.85 (d, *J* = 9.0 Hz, 1H), 5.17 (s, 2H), 5.13 (s, 2H), 4.87 (s, 2H), 3.52 (s, 3H).

^{13}C NMR (101 MHz, $CDCl_3$): δ = 152.2, 151.0, 136.7, 130.8, 128.8, 128.2, 127.4, 116.2, 113.2, 96.4, 96.0, 71.3, 56.6, 34.8.

IR (ATR, neat): 2974, 1567, 1475, 1442, 1277, 1223, 1203, 1045, 1025, 869, 817, 754 cm^{-1}.

HRMS (ESI) calc. for $C_{16}H_{16}BrIO_3Na$ $[M+Na]^+$: 484.9220; found: 484.9215.

Phenol S19

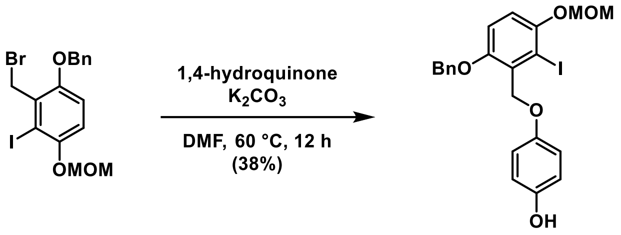

S18 S19

Potassium carbonate (7.46 mg, 54.0 µmol, 1.00 equiv) was added to a solution of benzyl bromide **S18** (25.0 mg, 54.0 µmol, 1 equiv) and 1,4-hydrochinone (11.9 mg, 108 µmol, 2.00 equiv) in *N*,*N*-dimethylformamide (1.00 mL) and the resulting mixture was warmed to 60 °C. After 12 h, water (5 mL) and ethyl acetate (5 mL) were added to the reaction mixture. The layers were separated, and the aqueous phase was extracted with ethyl acetate (2 × 5 mL). The combined organic phases were washed with 1 M aqueous lithium chloride solution (2 × 5 mL) and water (5 mL). The washed organic layers were dried over magnesium sulfate and the dried solution was filtered. The filtrate was concentrated under reduced pressure and the combined residues were purified by flash column chromatography on silica gel (30% ethyl acetate in petroleum ether) to give phenol **S19** (10.0 mg, 20.3 µmol, 38%) as a white solid.

Analytical data for phenol **S19**:

TLC (30% ethyl acetate in petroleum ether): R_f = 0.32 (UV, CAM).

^{1}H NMR (400 MHz, $CDCl_3$): δ = 7.39 – 7.27 (m, 5H), 7.04 (d, *J* = 9.0 Hz, 1H), 6.98 – 6.83 (m, 3H), 6.81 – 6.64 (m, 2H), 5.25 (s, 2H), 5.18 (s, 2H), 5.06 (s, 2H), 4.59 (s, 1H), 3.53 (s, 3H).

^{13}C NMR (101 MHz, $CDCl_3$): δ = 153.5, 153.0, 151.1, 149.9, 136.9, 130.1, 128.7, 128.1, 127.4, 116.8, 116.4, 116.0, 114.0, 97.5, 96.0, 71.8, 70.0, 56.6.

IR (ATR, neat): 3221, 2978, 1671, 1607, 1590, 1522, 1477, 1446, 1395, 1370, 1316, 1286, 1241, 1169, 1136, 1108, 871, 850, 771 cm^{-1}.

HRMS (ESI) calc. for $C_{22}H_{21}IO_5Na^+$ $[M+Na]^+$: 515.0326; found: 515.0316.

Quinone monoacetal S20

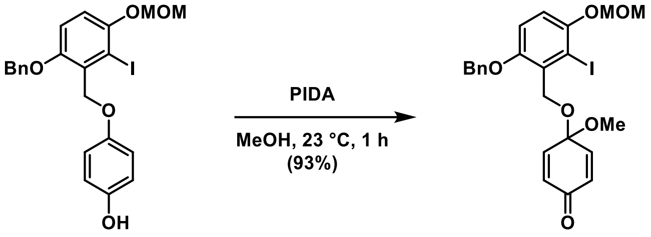

(Diacetoxyiodo)benzene (12.4 mg, 38.6 µmol, 1.05 equiv) was added to a solution of phenol **S19** (18.1 mg, 36.8 µmol, 1 equiv) in dry methanol (400 µmL). After 1 h, water (3 mL) and diethyl ether (3 mL) were added to the reaction mixture. The layers were separated, and the aqueous phase was extracted with diethyl ether (2 × 3 mL). The combined organic layers were dried over magnesium sulfate and the dried solution was filtered. The filtrate was concentrated under reduced pressure and the residue was purified by flash column chromatography on silica gel (30% ethyl acetate and 2% triethylamine in petroleum ether) to give quinone monoacetal **S20** (17.9 mg, 34.3 µmol, 93%) as a brown oil.

Analytical data for quinone monoacetal **S20**:

TLC (30% ethyl acetate in petroleum ether): R_f = 0.38 (UV, CAM).

^{1}H NMR (400 MHz, $CDCl_3$): δ = 7.44 – 7.30 (m, 5H), 7.03 (d, *J* = 9.0 Hz, 1H), 6.96 – 6.82 (m, 3H), 6.27 – 6.10 (m, 2H), 5.16 (s, 2H), 5.02 (s, 2H), 4.85 (s, 2H), 3.51 (s, 3H), 3.40 (s, 3H).

^{13}C NMR (101 MHz, $CDCl_3$): δ = 185.5, 153.0, 150.8, 143.9, 136.6, 129.9, 129.9, 128.7, 128.4, 127.9, 116.4, 113.1, 97.7, 96.0, 92.8, 71.5, 64.6, 56.5, 51.0.

IR (ATR, neat): 2921, 2356, 2323, 2174, 1657, 1642, 1591, 1464, 1450, 1367, 1308, 1251, 1189, 1151, 1066, 1027, 990, 901, 727 cm^{-1}.

HRMS (ESI) calc. for $C_{23}H_{23}IO_6Na^+$ $[M+Na]^+$: 545.0432; found: 545.0419.

Attempts of the the radical-addition/aldol reaction cascade employing quinone monoacetal S20

Table 5: Screened conditions.

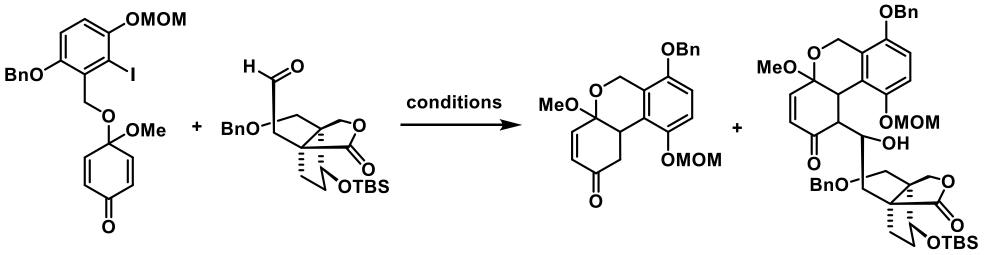

S20 **15** **S21** **S22**

Entry	35	15	Reagents	Temp.	Result
**1**	1.6 equiv	1 equiv	BEt_3 (4.0 equiv), $HSnBu_3$ (1.2 equiv), air, toluene	–78 °C	recovered **15**, + **S21** (not isolated), **S22** not formed
**2**	1.6 equiv	1 equiv	BEt_3 (4.0 equiv), $HSnBu_3$ (1.2 equiv), air, toluene	–50 °C	recovered **15**, + **S21** (40%), **S22** not formed
**3**	1.6 equiv	1 equiv	BEt_3 (4.0 equiv), $HSnBu_3$ (1.2 equiv), air, toluene	0 °C	recovered **15**, + **S21** (not isolated), **S22** not formed
**4**	1.6 equiv	1 equiv	BEt_3 (4.0 equiv), $HSnBu_3$ (1.2 equiv), air, toluene	23 °C	recovered **15** + decomposition, **S22** not formed

Possible transition state for the aldol addition

In this case, steric hindrance from the C4a substituent (OMOM group) prevents the formation of the boron-mediated six-membered transition state. This likely explains why the aldol reaction does not occur.

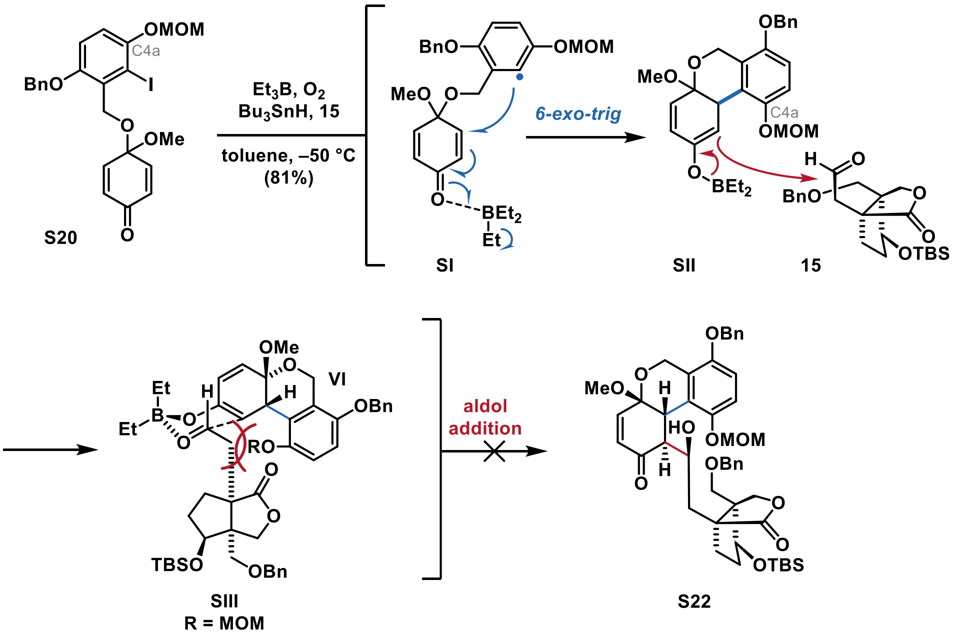

Scheme 3: Attempted aldol reaction via six-membered transition state SIII.

3.3. Synthesis and application of quinone monoacetal S28

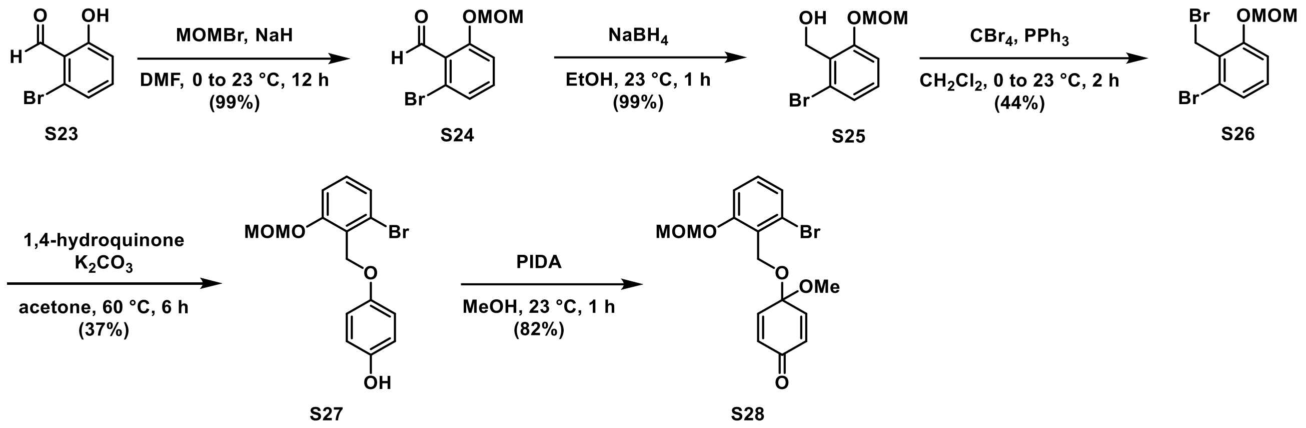

Scheme 4: Synthetic route towards quinone monoacetal **S28**.

MOM-ether S24

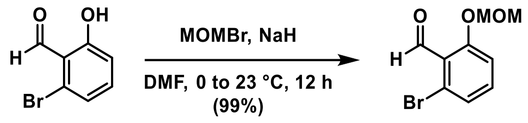

S23 S24

Sodium hydride (1.84 g, 76.6 mmol, 2.00 equiv, 60 wt% in mineral oil) was added to a solution of aldehyde **S23** (7.70 g, 38.3 mmol, 1 equiv) in *N*,*N*-dimethylformamide (380 mL) at 0 °C. After 30 min, bromomethyl methyl ether (4.67 mL, 57.5 mmol, 1.50 equiv) was added to the reaction mixture at 0 °C, which was then warmed to 23 °C. After 12 h, the reaction mixture was cooled to 0 °C and water (400 mL) and ethyl acetate (400 mL) were added to the reaction mixture. The layers were separated, and the aqueous phase was extracted with ethyl acetate (2 × 200 mL). The combined organic phases were washed with water (3 × 500 mL). The washed organic layers were dried over magnesium sulfate and the dried solution was filtered. The filtrate was concentrated under reduced pressure to give MOM-ether **S24** (9.39 g, 38.3 mmol, 99%) as a yellow oil.

Analytical data for MOM-ether **S24**:

TLC (20% ethyl acetate in petroleum ether): R_f = 0.43 (UV, CAM).

^{1}H NMR (400 MHz, $CDCl_3$): δ = 10.36 (s, 1H), 7.25 – 7.21 (m, 2H), 7.15 – 7.09 (m, 1H), 5.20 (s, 2H), 3.44 (s, 3H).

^{13}C NMR (101 MHz, $CDCl_3$): δ = 190.3, 159.7, 134.8, 127.8, 124.6, 124.4, 114.9, 95.2, 56.7.

IR (ATR, neat): 2957, 2925, 2853, 1699, 1655, 1585, 1566, 1453, 1399, 1307, 1282, 1253, 1306, 1282, 1201, 1156, 1086, 990, 960, 923, 780 cm^{-1}.

HRMS (ESI) calc. for $C_9H_9BrO_3Na^+$ $[M+Na]^+$: 266.9627; found: 266.9622.

Benzylic alcohol S25

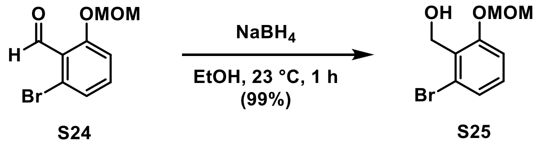

Sodium borohydride (4.35 g, 115 mmol, 3.00 equiv) was added to a solution of benzaldehyde **S24** (9.39 g, 38.3 mmol, 1 equiv) in ethanol (380 mL) at 23 °C. After 1 h, water (300 mL) was added to the reaction mixture and the reaction mixture was concentrated under reduced pressure. Water (300 mL) and dichloromethane (300 mL) were added to the residue, the layers were separated and the aqueous phase was extracted with dichloromethane (2 × 500 mL). The combined organic phases were dried over magnesium sulfate and the dried solution was filtered. The filtrate was concentrated under reduced pressure to give benzylic alcohol **S25** (9.46 g, 38.3 mmol, 99%) as a yellow solid.

Analytical data for benzylic alcohol **S25**:

TLC (30% ethyl acetate in petroleum ether): R_f = 0.28 (UV, CAM).

^{1}H NMR (400 MHz, $CDCl_3$): δ = 7.24 (dd, *J* = 7.3, 1.9 Hz, 1H), 7.14 – 7.06 (m, 2H), 5.23 (s, 2H), 4.89 (s, 2H), 3.50 (s, 3H), 2.36 (s, 1H).

^{13}C NMR (101 MHz, $CDCl_3$): δ = 156.7, 130.2, 129.7, 126.5, 125.3, 114.1, 95.2, 60.3, 56.5.

IR (ATR, neat): 2957, 2925, 2896, 2852, 1589, 1571, 1453, 1439, 1404, 1310, 1253, 1203, 1155, 1137, 1081, 984, 922, 833, 774, 733 cm^{-1}.

HRMS (ESI) calc. for $C_9H_{11}BrO_3Na^+$ $[M+Na]^+$: 268.9784; found: 268.9778.

Benzyl bromide S26

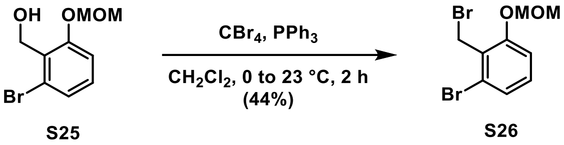

Carbon tetrabromide (15.2 g, 46.0 mmol, 1.20 equiv) and triphenylphosphine (14.1 g, 53.6 mmol, 1.40 equiv) were added to a solution of benzylic alcohol **S25** (9.46 g, 38.3 mmol, 1 equiv) in dichloromethane (380 mL) at 0 °C. After 30 min, the reaction mixture was allowed to warm to 23 °C. After 1.5 h, the reaction mixture was concentrated under reduced pressure and the residue was purified by flash column chromatography on silica gel (0 to 5% ethyl acetate in petroleum ether) to give benzyl bromide **S26** (5.26 g, 17.0 mmol, 44%) as a colorless oil.

Analytical data for benzyl bromide **S26**:

TLC (5% ethyl acetate in petroleum ether): R_f = 0.48 (UV, CAM).

^{1}H NMR (400 MHz, $CDCl_3$): δ = 7.22 (dd, *J* = 7.9, 1.3 Hz, 1H), 7.11 (t, *J* = 8.1 Hz, 1H), 7.06 (dd, *J* = 8.4, 1.3 Hz, 1H), 5.27 (s, 2H), 4.75 (s, 2H), 3.52 (s, 3H).

^{13}C NMR (101 MHz, $CDCl_3$): δ = 156.2, 130.6, 126.8, 126.2, 125.8, 113.3, 94.5, 56.5, 28.4.

IR (ATR, neat): 2957, 2899, 2826, 1588, 1569, 1456, 1402, 1308, 1254, 1224, 1205, 1188, 1165, 1146, 1082, 999, 961, 921, 882, 826, 774, 736 cm^{-1}.

HRMS (ESI) calc. for $C_9H_{11}Br_2O_2$ [M–Br]$^+$: 228.9859; found: 228.9853.

Phenol S27

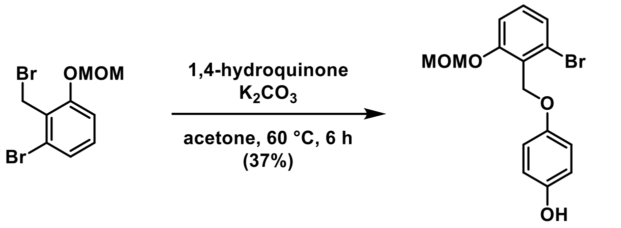

S26 S27

Potassium carbonate (2.35 g, 17.0 mmol, 1.00 equiv) was added to a solution of benzyl bromide **S26** (5.26 g, 17.0 mmol, 1 equiv) and 1,4-hydrochinone (13.1 g, 119 mmol, 7.00 equiv) in acetone (170 mL) and the resulting mixture was warmed to 60 °C. The reaction mixture was concentrated under reduced pressure after 6 h and water (100 mL) and ethyl acetate (100 mL) were added to the residue. The layers were separated, and the aqueous phase was extracted with ethyl acetate (2 × 100 mL). The combined organic phases were dried over magnesium sulfate and the dried solution was filtered. The filtrate was concentrated under reduced pressure and the combined residues were purified by flash column chromatography on silica gel (30% ethyl acetate in petroleum ether) to give phenol **S27** (2.14 g, 6.31 mmol, 37%) as a yellow oil.

Analytical data for phenol **S27**:

TLC (30% ethyl acetate in petroleum ether): R_f = 0.35 (UV, CAM).

^{1}H NMR (400 MHz, $CDCl_3$): δ = 7.28 (dd, *J* = 7.9, 1.3 Hz, 1H), 7.16 (t, *J* = 8.1 Hz, 1H), 7.10 (dd, *J* = 8.4, 1.3 Hz, 1H), 6.95 – 6.89 (m, 2H), 6.79 – 6.73 (m, 2H), 5.19 (s, 2H), 5.17 (s, 2H), 4.76 (s, 1H), 3.46 (s, 3H).

^{13}C NMR (101 MHz, $CDCl_3$): δ = 157.1, 153.4, 150.0, 130.9, 127.0, 126.5, 125.9, 116.6, 116.1, 114.0, 95.0, 65.6, 56.4.

IR (ATR, neat): 3385, 2954, 2827, 1591, 1573, 1506, 1451, 1404, 1381, 1309, 1253, 1204, 1157, 1143, 1101, 1081, 997, 923, 874, 769 721 cm^{-1}.

HRMS (ESI) calc. for $C_{15}H_{16}BrO_4^+$ $[M+H]^+$: 339.0226; found: 339.0219.

Quinone monoacetal S27

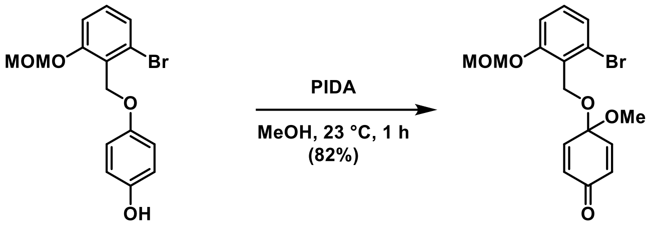

S27 S28

(Diacetoxyiodo)benzene (910 mg, 2.82 mmol, 1.03 equiv) was added to a solution of phenol **S27** (930 mg, 2.74 mmol, 1 equiv) in dry methanol (15 mL). After 1 h, water (10 mL) and diethyl ether (10 mL) were added to the reaction mixture. The layers were separated, and the aqueous phase was extracted with diethyl ether (2 × 5 mL). The combined organic layers were dried over magnesium sulfate and the dried solution was filtered. The filtrate was concentrated under reduced pressure and the residue was purified by flash column chromatography on silica gel (20% ethyl acetate and 2% triethylamine in petroleum ether) to give quinone monoacetal **S28** (830 mg, 2.25 mmol, 82%) as a brown oil.

Analytical data for quinone monoacetal **S28**:

TLC (30% ethyl acetate in petroleum ether): R_f = 0.40 (UV, CAM).

^{1}H NMR (400 MHz, $CDCl_3$): δ = 7.24 (dd, *J* = 7.9, 1.2 Hz, 1H), 7.13 (t, *J* = 8.1 Hz, 1H), 7.06 (dd, *J* = 8.3, 1.2 Hz, 1H), 7.01 – 6.95 (m, 2H), 6.34 – 6.25 (m, 2H), 5.18 (s, 2H), 4.81 (s, 2H), 3.48 (s, 3H), 3.45 (s, 3H).

^{13}C NMR (101 MHz, $CDCl_3$): δ = 185.5, 157.0, 143.8, 130.8, 129.9, 126.8, 126.4, 126.0, 113.8, 94.9, 92.8, 59.9, 56.4, 50.9.

IR (ATR, neat): 2940, 2830, 1687, 1673, 1638, 1572, 1459, 1384, 1309, 1255, 1205, 1177, 1158, 1144, 1101, 1084, 1024, 995, 922, 862, 847, 780 cm^{-1}.

HRMS (ESI) calc. for $C_{16}H_{18}BrO_5$ $[M+H]^+$: 369.0332; found: 369.0325.

Attempt of the the radical-addition/aldol reaction cascade employing quinone monoacetal S28

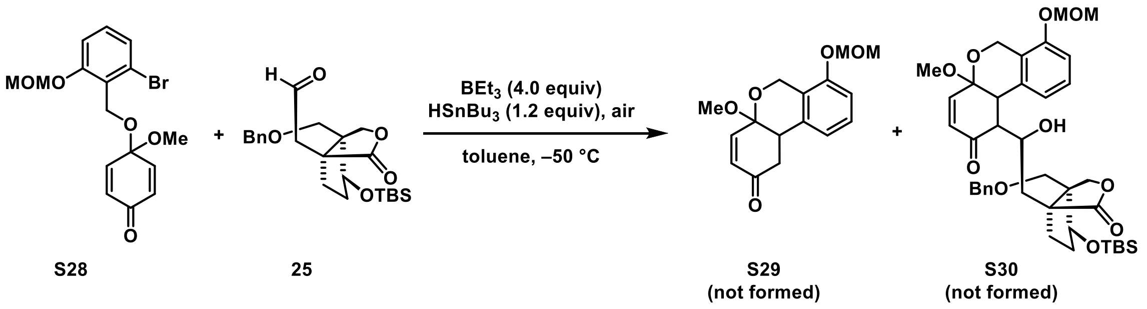

Scheme 5: Attempted radical-addition/aldol reaction sequence.

3.4. Synthesis and application of model substrate S36

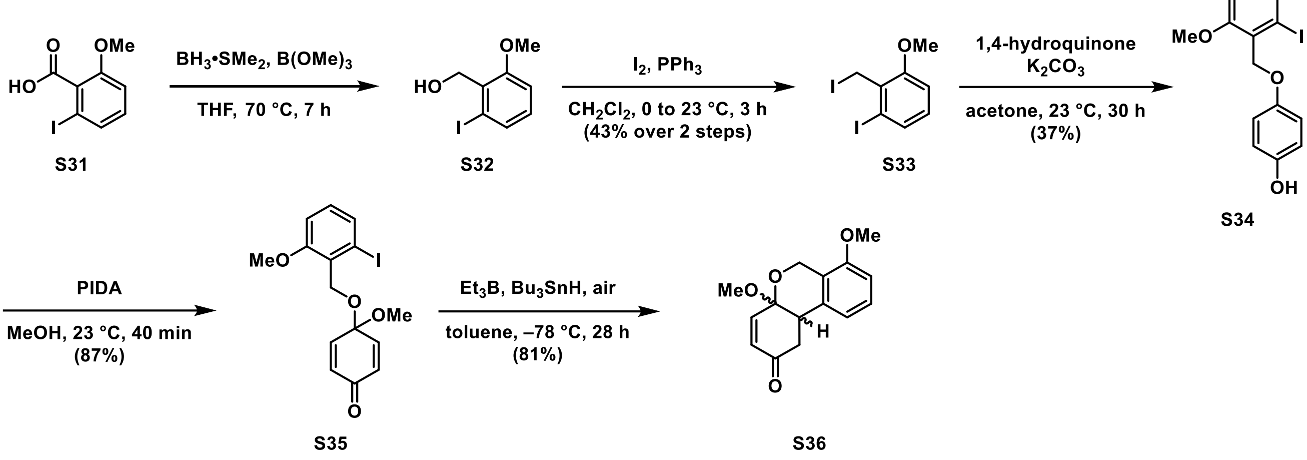

Scheme 6: Synthetic route towards tricycle **S36**.

Benzylic alcohol S32

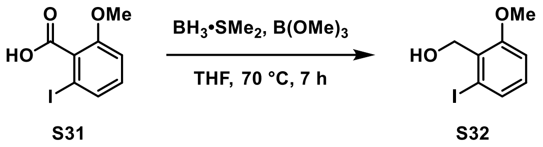

Borane dimethyl sulfide (5.43 mL, 62.8 mmol, 1.50 equiv) and trimethyl borate (7.13 mL, 62.8 mmol, 1.50 equiv) were added to a solution of benzoic acid **S31** (11.6 g, 41.9 mmol, 1 equiv) in tetrahydrofuran (71 mL) at 23 °C, which was then warmed to 70 °C. After 7 h, methanol (100 mL) was added slowly to the reaction mixture and the reaction mixture was concentrated under reduced pressure to give crude benzylic alcohol **S32** (11.1 g, 41.9 mmol) as a pale yellow solid, which was used in the next step without further purification.

Analytical data for benzylic alcohol **S32**:

^{1}H NMR (400 MHz, $CDCl_3$) δ = 7.47 (dd, *J* = 7.9, 1.0 Hz, 1H), 6.99 (t, *J* = 8.1 Hz, 1H), 6.90 (dd, *J* = 8.3, 1.1 Hz, 1H), 4.89 (s, 2H), 3.88 (s, 3H), OH not detected due to exchange events.

Benzyl iodide S33

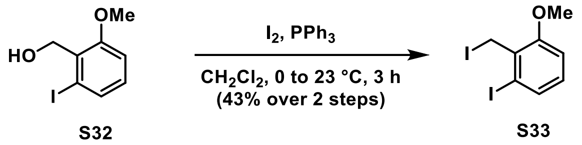

Triphenylphosphine (12.1 g, 46.1 mmol, 1.10 equiv) and iodine (10.6 g, 41.9 mmol, 1.00 equiv) were added to a solution of benzylic alcohol **S32** (11.1 g, 41.9 mmol, 1 equiv) in dichloromethane (200 mL) at 0 °C, which was then warmed to 23 °C. After 3 h, aqueous saturated sodium thiosulfate solution (100 mL) was added to the reaction mixture. The layers were separated, and the aqueous phase was extracted with dichloromethane (2 × 70 mL). The combined organic phases were dried over magnesium sulfate and the dried solution was filtered. The filtrate was concentrated under reduced pressure and the residue was purified by flash column chromatography on silica gel (0 to 2% ethyl acetate in petroleum ether) to give benzyl bromide **S33** (6.83 g, 18.3 mmol, 43% over 2 steps) as a white solid.

Analytical data for quinone benzyl iodide **S33**:

TLC (5% ethyl acetate in petroleum ether): R_f = 0.40 (UV, CAM).

^{1}H NMR (400 MHz, $CDCl_3$): δ = 7.41 (dd, *J* = 7.9, 1.0 Hz, 1H), 6.94 (t, *J* = 8.1 Hz, 1H), 6.81 (dd, *J* = 8.3, 1.0 Hz, 1H), 4.65 (s, 2H), 3.90 (s, 3H).

^{13}C NMR (101 MHz, $CDCl_3$): δ = 157.6, 131.9, 130.5, 130.0, 111.0, 101.4, 56.1, 8.0.

IR (ATR, neat): 2955, 2933, 2831, 1581, 1564, 1458, 1429, 1415, 1297, 1261, 1210, 1197, 1151, 1032, 845, 818, 772, 733 cm^{-1}.

HRMS (ESI) calc. for $C_8H_9I_2O$ $[M+H]^+$: 374.8737; found: mass not found.

Phenol S34

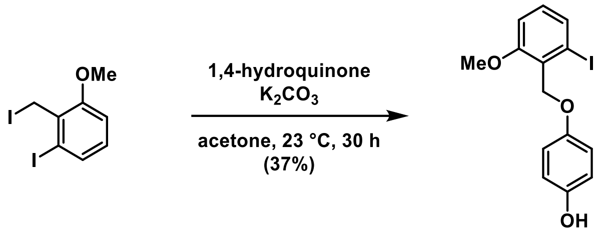

S33 S34

Potassium carbonate (111 mg, 0.802 mmol, 1.00 equiv) was added to a solution of benzyl iodide **S33** (300 mg, 0.802 mmol, 1 equiv) and 1,4-hydrochinone (177 mg, 1.60 mmol, 2.00 equiv) in acetone (5.00 mL). After 30 h, water (20 mL) and ethyl acetate (20 mL) were added to the reaction mixture. The layers were separated, and the aqueous phase was extracted with ethyl acetate (2 × 20 mL). The combined organic layers were dried over magnesium sulfate and the dried solution was filtered. The filtrate was concentrated under reduced pressure and the residue was purified by flash column chromatography on silica gel (3% ethyl acetate in dichloromethane) to give phenol **S34** (107 mg, 0.300 mmol, 37%) as a brown solid.

Analytical data for phenol **S34**:

TLC (20% ethyl acetate in petroleum ether): R_f = 0.26 (UV, CAM).

^{1}H NMR (400 MHz, $CDCl_3$): δ = 7.50 (dd, *J* = 7.9, 1.0 Hz, 1H), 7.02 (t, *J* = 8.1 Hz, 1H), 6.97 – 6.87 (m, 3H), 6.81 – 6.74 (m, 2H), 5.12 (s, 2H), 4.41 (s, 1H), 3.82 (s, 3H).

^{13}C NMR (101 MHz, $CDCl_3$): δ = 158.5, 153.4, 149.8, 131.9, 131.3, 127.8, 116.5, 115.9, 111.1, 103.0, 69.3, 56.0.

IR (ATR, neat): 3376, 1567, 1508, 1459, 1432, 1264, 1225, 1030, 827, 768 cm^{-1}.

HRMS (ESI) calc. for $C_{14}H_{14}IO_3$ $[M+H]^+$: 356.9982; found: 356.9976.

Quinone monoacetal S35

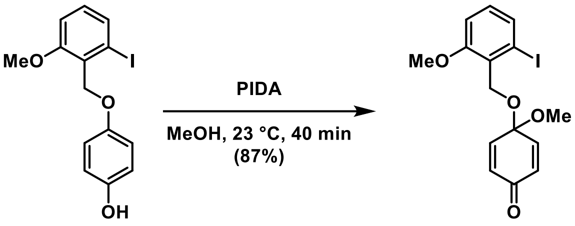

S34 S35

(Diacetoxyiodo)benzene (3.19 g, 9.89 mmol, 1.03 equiv) was added in small portions to a solution of phenol **S34**(3.42 g, 9.60 mmol, 1 equiv) in dry methanol (48 mL). After 40 min, water (50 mL) and diethyl ether (50 mL) were added to the reaction mixture. The layers were separated, and the aqueous phase was extracted with diethyl ether (2 × 50 mL). The combined organic layers were dried over magnesium sulfate and the dried solution was filtered. The filtrate was concentrated under reduced pressure and the residue was purified by flash column chromatography on silica gel (50% diethyl ether and 1% triethylamine in cyclohexane) to give quinone monoacetal **S35** (3.24 g, 8.39 mmol, 87%) as a yellow oil.

Analytical data for quinone monoacetal **S35**:

TLC (25% ethyl acetate in cyclohexane): R_f = 0.40 (UV, CAM).

^{1}H NMR (400 MHz, $CDCl_3$): δ = 7.45 (dd, *J* = 7.9, 1.0 Hz, 1H), 7.07 – 6.92 (m, 3H), 6.85 (dd, *J* = 8.3, 1.0 Hz, 1H), 6.42 – 6.24 (m, 2H), 4.76 (s, 2H), 3.81 (s, 3H), 3.49 (s, 3H).

^{13}C NMR (101 MHz, $CDCl_3$): δ = 185.5, 158.5, 143.9, 131.9, 131.3, 129.9, 128.3, 111.1, 103.1, 92.8, 64.1, 56.0, 51.0.

IR (ATR, neat): 2940, 2836, 1687, 1673, 1637, 1585, 1568, 1460, 1433, 1384, 1304, 1265, 1177, 1147, 1101, 1085, 1028, 968, 914 cm^{-1}.

HRMS (ESI) calc. for $C_{15}H_{16}IO_4$ $[M+H]^+$: 387.0088; found: 387.0085.

Tricycle S36

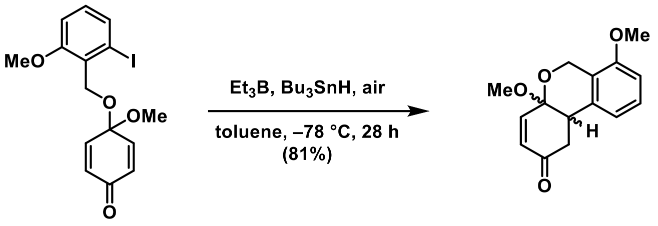

S35 S36

Tributyltin hydride (191 µL, 684 µmol, 1.20 equiv) was added to a solution of quinone monoacetal **S35** (220 mg, 0.570 mmol, 1 equiv) in toluene (18.0 mL) at 23 °C. Triethylborane (0.798 mL, 1.00 M in tetrahydrofuran, 798 µmol, 1.40 equiv) was then added at –78 °C, followed by the addition of air (2.00 mL), which was bubbled through the solution. After 28 h, water (20 mL) and ethyl acetate (20 mL) were added to the reaction mixture. The layers were separated, and the aqueous phase was extracted with ethyl acetate (2 × 20 mL). The combined organic phases were dried over magnesium sulfate and the dried solution was filtered. The filtrate was concentrated under reduced pressure and the residue was purified by flash column chromatography on silica gel (10% ethyl acetate and 2% triethylamine in petroleum ether) to give tricycle **S36** (120 mg, 461 µmol, 81%) as a pale yellow oil.

Analytical data for tricycle **S36**:

TLC (10% ethyl acetate in petroleum ether): R_f = 0.32 (UV, CAM).

^{1}H NMR (400 MHz, $CDCl_3$): δ = 7.15 (dd, *J* = 8.3, 7.5 Hz, 1H), 6.96 (d, *J* = 10.4 Hz, 1H), 6.70 – 6.55 (m, 2H), 6.07 (dd, *J* = 10.4, 0.9 Hz, 1H), 4.89 (d, *J* = 16.0 Hz, 1H), 4.61 (dt, *J* = 15.9, 1.1 Hz, 1H), 3.76 (s, 3H), 3.41 (s, 3H), 3.23 – 3.16 (m, 1H), 2.69 – 2.54 (m, 2H).

^{13}C NMR (101 MHz, $CDCl_3$): δ = 198.2, 154.9, 142.7, 135.0, 130.8, 127.9, 120.3, 120.1, 108.0, 94.7, 60.6, 55.2, 49.4, 44.5, 41.0.

IR (ATR, neat): 2941, 2838, 1685, 1632, 1594, 1473, 1440, 1391, 1374, 1315, 1259, 1206, 1132, 1105, 1081, 1068, 1033, 992, 964, 920, 857 cm^{-1}.

HRMS (ESI) calc. for $C_{15}H_{17}O_4^+$ $[M+H]^+$: 261.1121; found: 261.1114.

Attempts of an aldol reaction employing tricycle S36 and aldehyde 15

Table 6: Screened conditions.

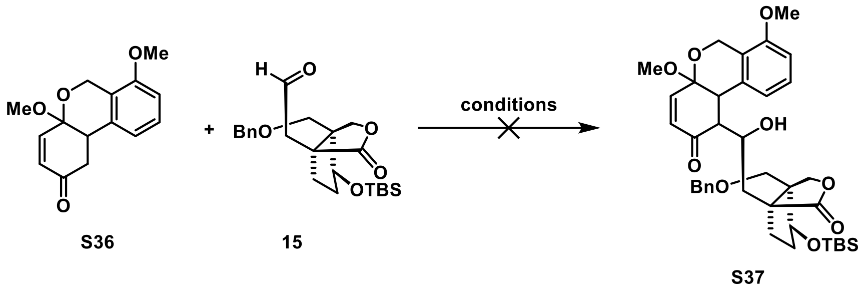

Entry	Reagents	Temp.	Result
**1**	LDA, THF	–78 to 23 °C	no consumption of **S36** and **15**
**2**	KHMDS, THF	–78 to 23 °C	no consumption of **S36** and **15**
**3**	LHMDS, THF	–78 to 23 °C	no consumption of **S36** and **15**
**4**	DIPEA, *n*-Bu_2BOTf	–78 to 23 °C	no consumption of **S36** and **15**
**5**	NEt_3, Cy_2BOTf	–78 to 23 °C	no consumption of **S36** and **15**

4. References

(1) Morrill, C.; Péter, Á.; Amalina, I.; Pye, E.; Crisenza, G. E. M.; Kaltsoyannis, N.; Procter, D. J. Diastereoselective Radical 1,4-Ester Migration: Radical Cyclizations of Acyclic Esters with SmI_2. *J. Am. Chem. Soc.* **2022**, *144* (30), 13946–13952. https://doi.org/10.1021/jacs.2c05972.

(2) Nagasawa, S.; Sasano, Y.; Iwabuchi, Y. Synthesis of 1,3-Cycloalkadienes from Cycloalkenes: Unprecedented Reactivity of Oxoammonium Salts. *Angew Chem Int Ed* **2016**, *55* (42), 13189–13194. https://doi.org/10.1002/anie.201607752.

(3) Kohara, K.; Trowbridge, A.; Smith, M. A.; Gaunt, M. J. Thiol-Mediated α-Amino Radical Formation via Visible-Light-Activated Ion-Pair Charge-Transfer Complexes. *J. Am. Chem. Soc.* **2021**, *143* (46), 19268–19274. https://doi.org/10.1021/jacs.1c09445.

(4) Wollnitzke, P.; Essig, S.; Gölz, J. P.; Von Schwarzenberg, K.; Menche, D. Total Synthesis of Ajudazol A by a Modular Oxazole Diversification Strategy. *Org. Lett.* **2020**, *22* (16), 6344–6348. https://doi.org/10.1021/acs.orglett.0c02188.

(5) Chen, S.; Wei, F.; Cheng, X.; Luo, Y.; Meng, F.; Zhang, Y.; Huang, W.; Lv, J.; Pan, H.; Wu, Q.; Zhao, G. Regioselective Deacetylation of Peracetylated Deoxy- *C* -Glycopyranosides by Boron Trichloride (BCl_3). *J. Org. Chem.* **2024**, *89* (7), 4802–4817. https://doi.org/10.1021/acs.joc.4c00026.

(6) Riaz, M. T.; Pohorilets, I.; Hernandez, J. J.; Rios, J.; Totah, N. I. Preparation of 2-(Trimethylsilyl)Methyl-2-Propen-1-Ol Derivatives by Cobalt Catalyzed Sp2-Sp3 Coupling. *Tetrahedron Letters* **2018**, *59* (29), 2809–2812. https://doi.org/10.1016/j.tetlet.2018.06.018.

(7) Huang, C.; Xiong, J.; Guan, H.-D.; Wang, C.-H.; Lei, X.; Hu, J.-F. Discovery, Synthesis, Biological Evaluation and Molecular Docking Study of (R)-5-Methylmellein and Its Analogs as Selective Monoamine Oxidase A Inhibitors. *Bioorganic & Medicinal Chemistry* **2019**, *27* (10), 2027–2040. https://doi.org/10.1016/j.bmc.2019.03.060.

(8) Villar, F.; Equey, O.; Renaud, P. Desymmetrization of 1,4-Dien-3-Ols and Related Compounds via Ueno−Stork Radical Cyclizations. *Org. Lett.* **2000**, *2* (8), 1061–1064. https://doi.org/10.1021/ol005613v.

(9) Hu, Y.; Li, C.; Kulkarni, B. A.; Strobel, G.; Lobkovsky, E.; Torczynski, R. M.; Porco, J. A. Exploring Chemical Diversity of Epoxyquinoid Natural Products: Synthesis and Biological Activity of (−)-Jesterone and Related Molecules. *Org. Lett.* **2001**, *3* (11), 1649–1652. https://doi.org/10.1021/ol0159367.

5. ^{1}H and ^{13}C NMR Spectra

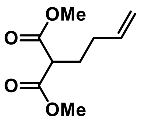

S1
^{1}H NMR, $CDCl_3$
300 MHz

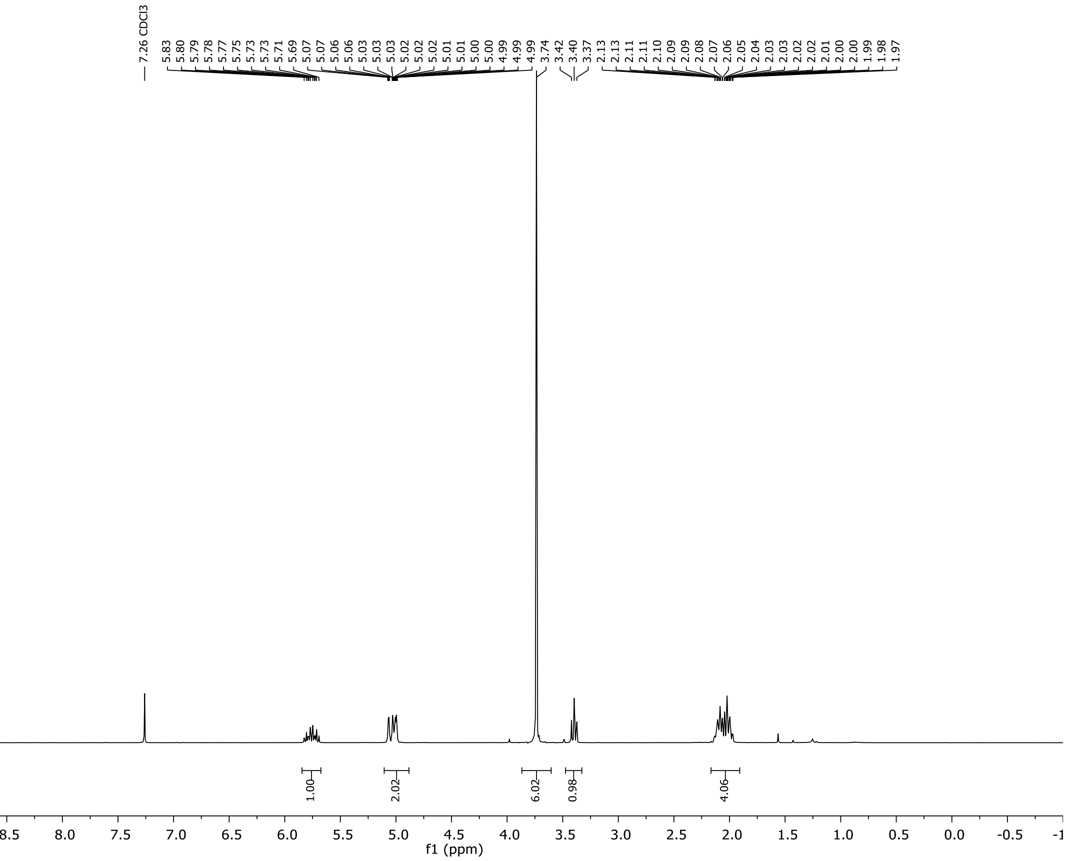

S1
^{13}C NMR, $CDCl_3$
75 MHz

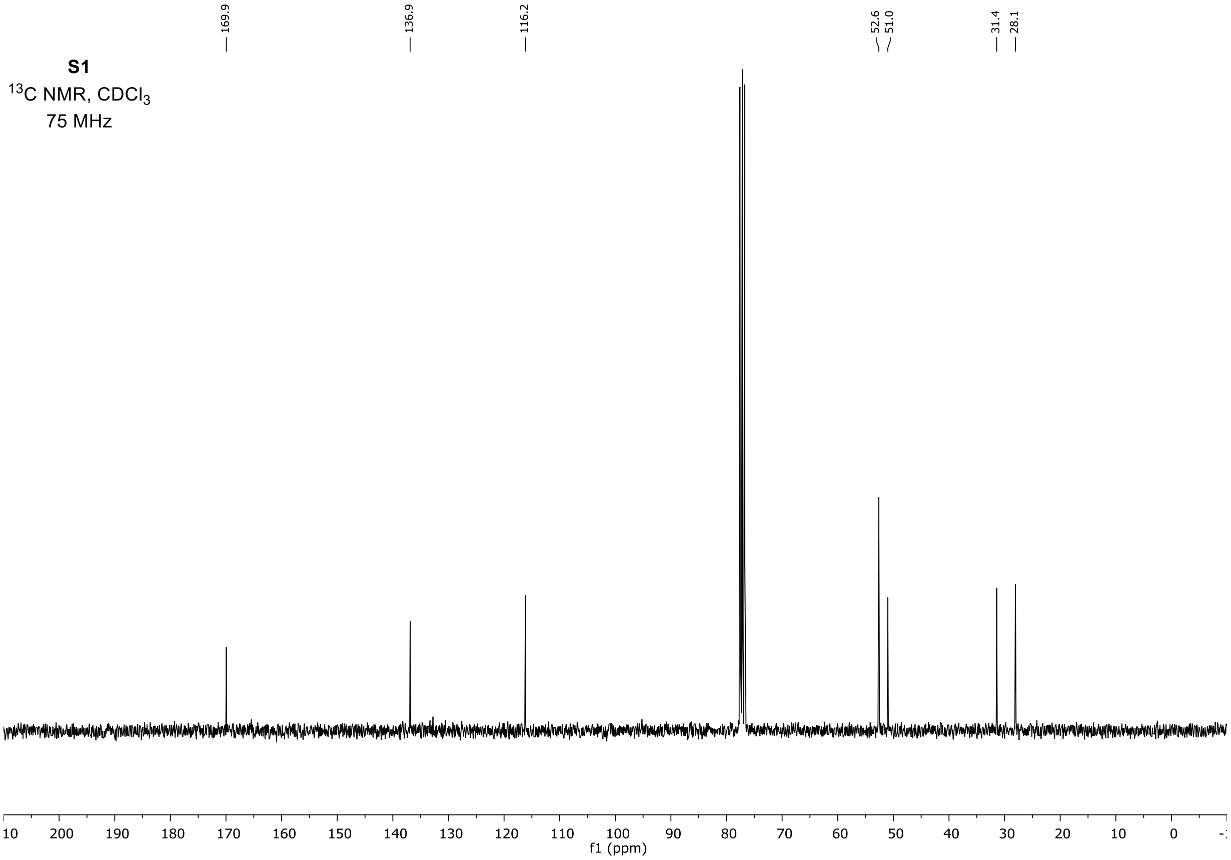

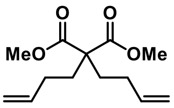

S2
^{1}H NMR, $CDCl_3$
400 MHz

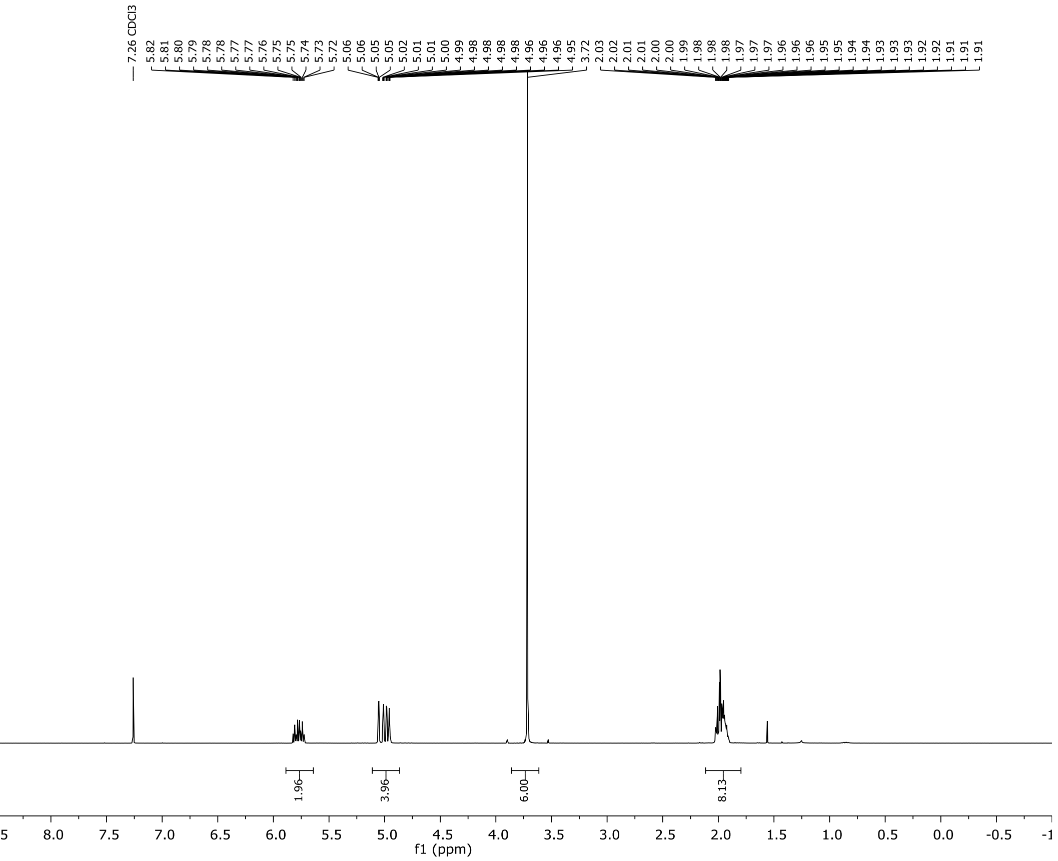

S2
^{13}C NMR, $CDCl_3$
101 MHz

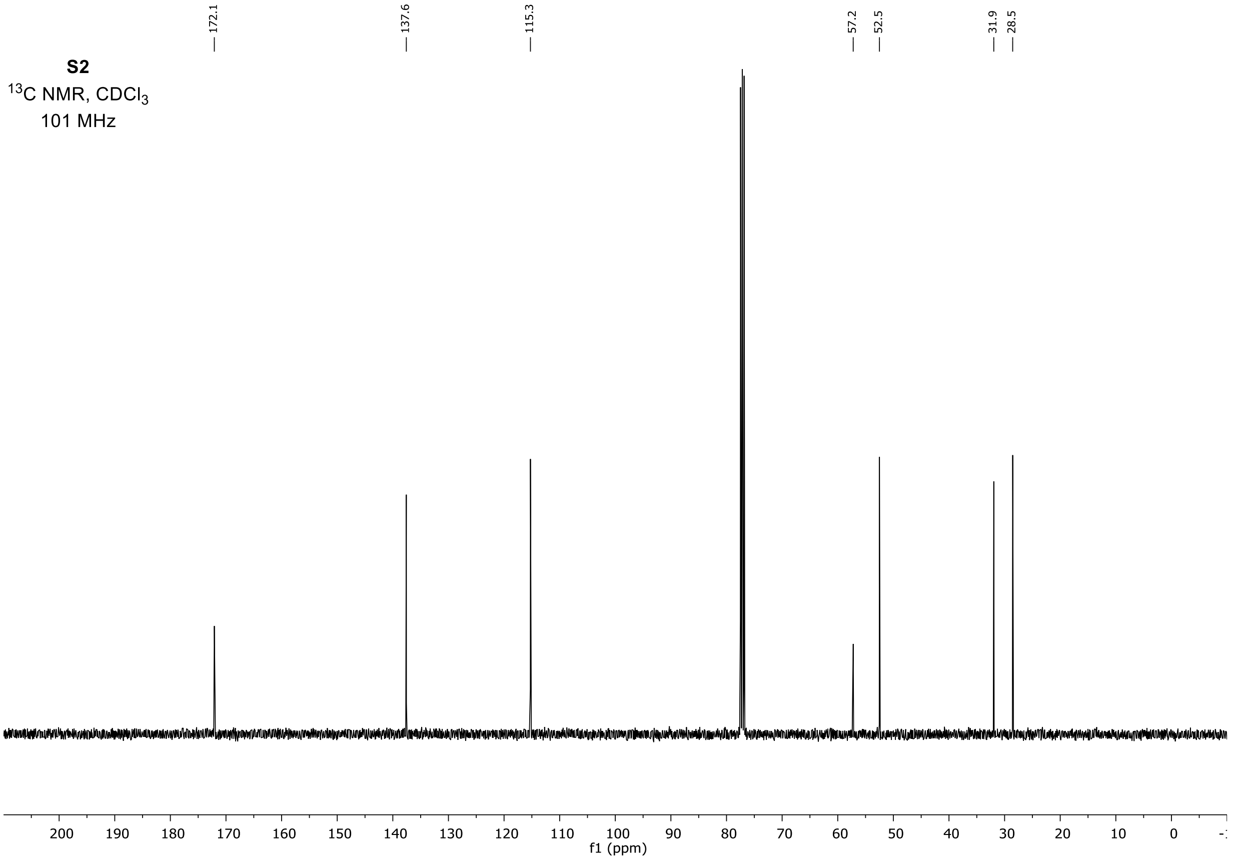

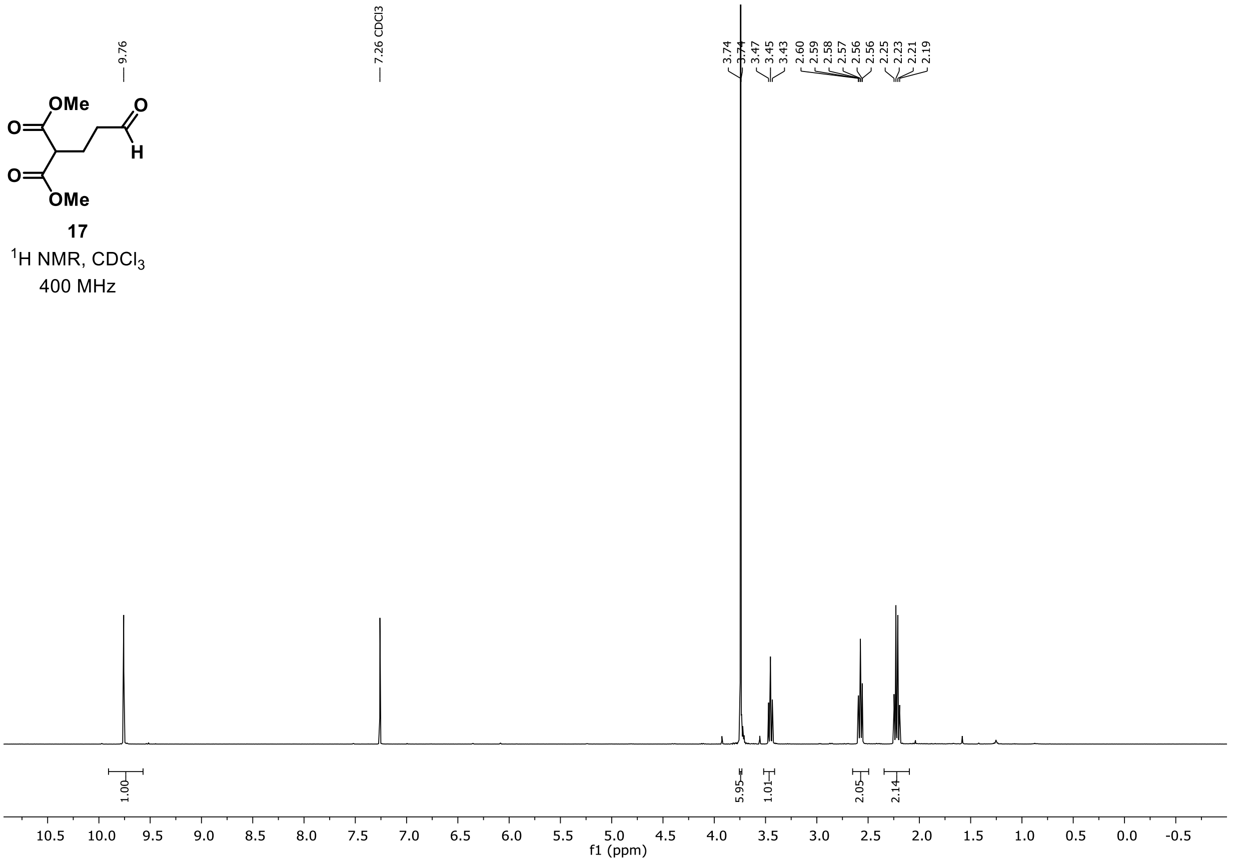
OMe
O
O
H
O
OMe
17
^{1}H NMR, $CDCl_3$
400 MHz
9.76
7.26 CDCl3
3.74
3.74
3.47
3.45
3.43
2.60
2.59
2.58
2.57
2.56
2.56
2.25
2.23
2.21
2.19
1.00
5.95
1.01
2.05
2.14
f1 (ppm)

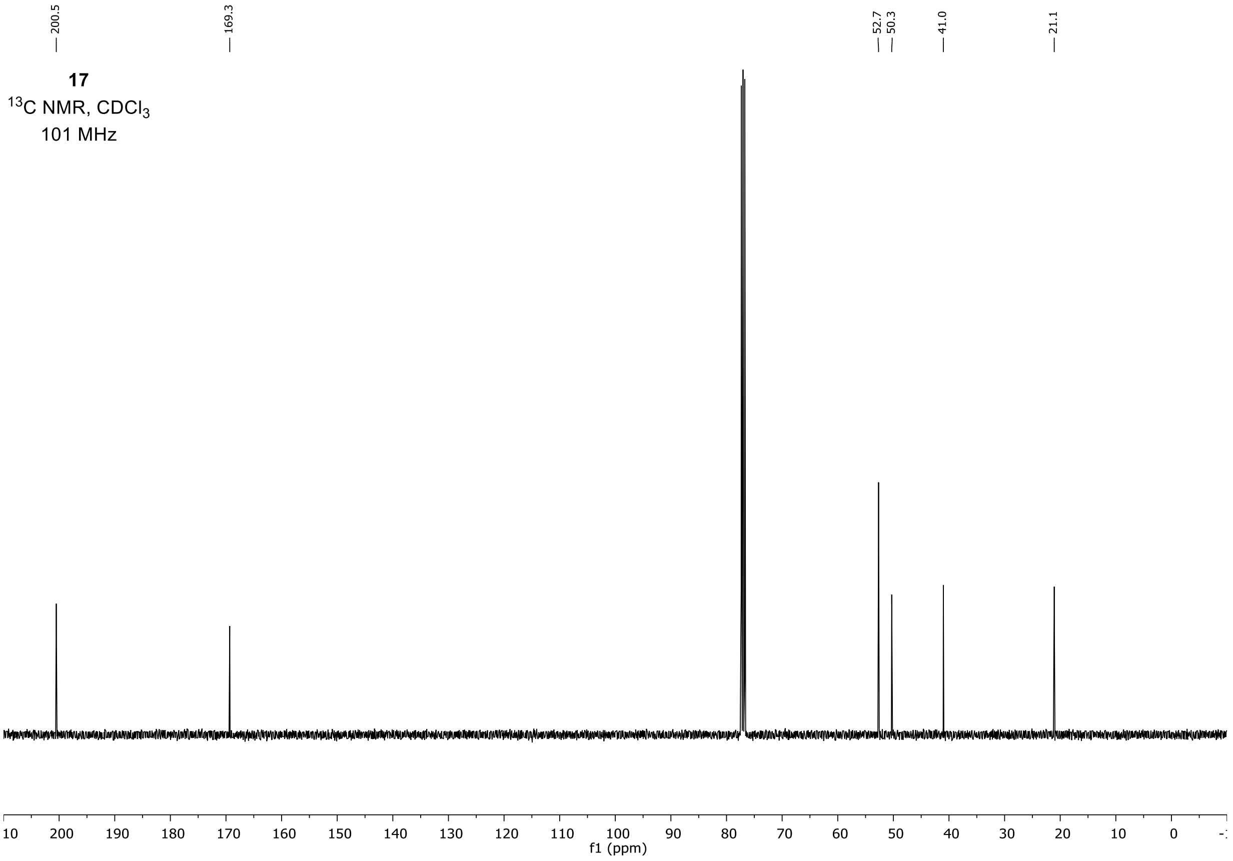
200.5
169.3
52.7
50.3
41.0
21.1
17
^{13}C NMR, $CDCl_3$
101 MHz
f1 (ppm)

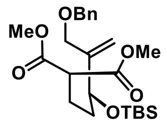

16
^{1}H NMR, $CDCl_3$
400 MHz

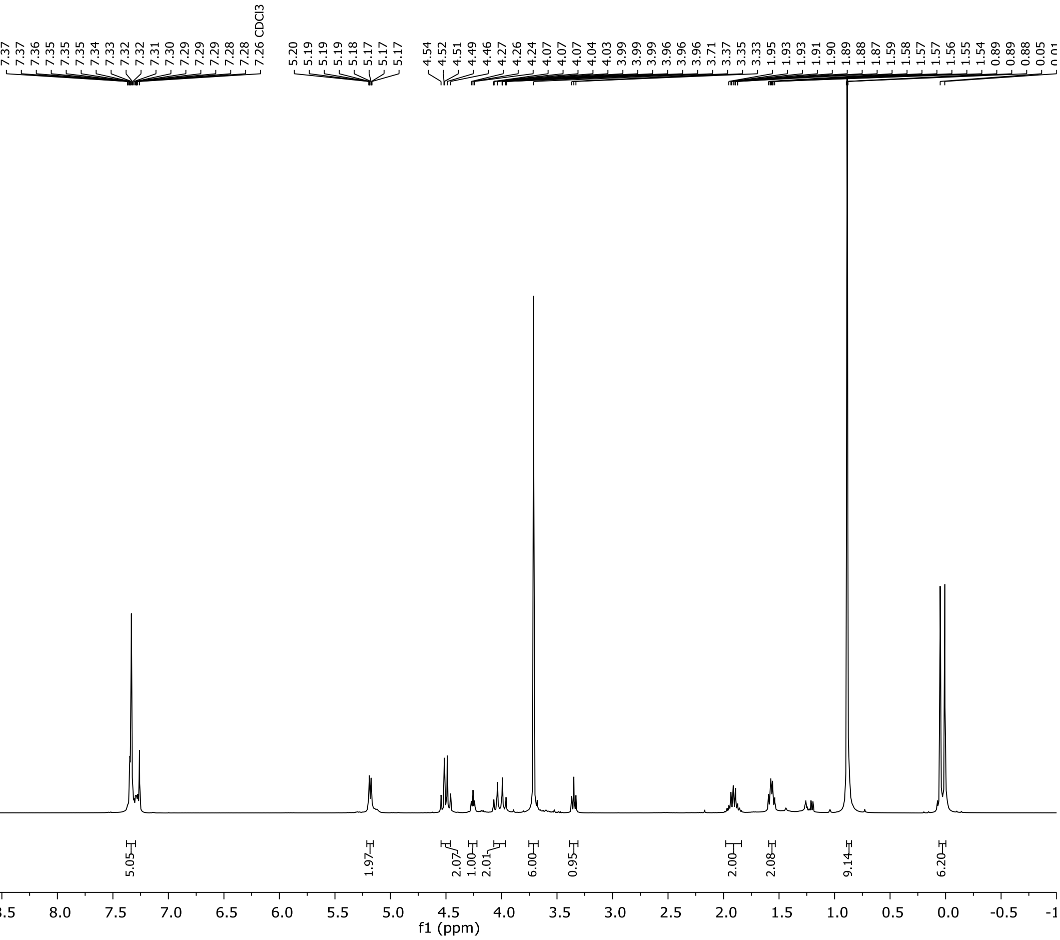

16
^{13}C NMR, $CDCl_3$
101 MHz

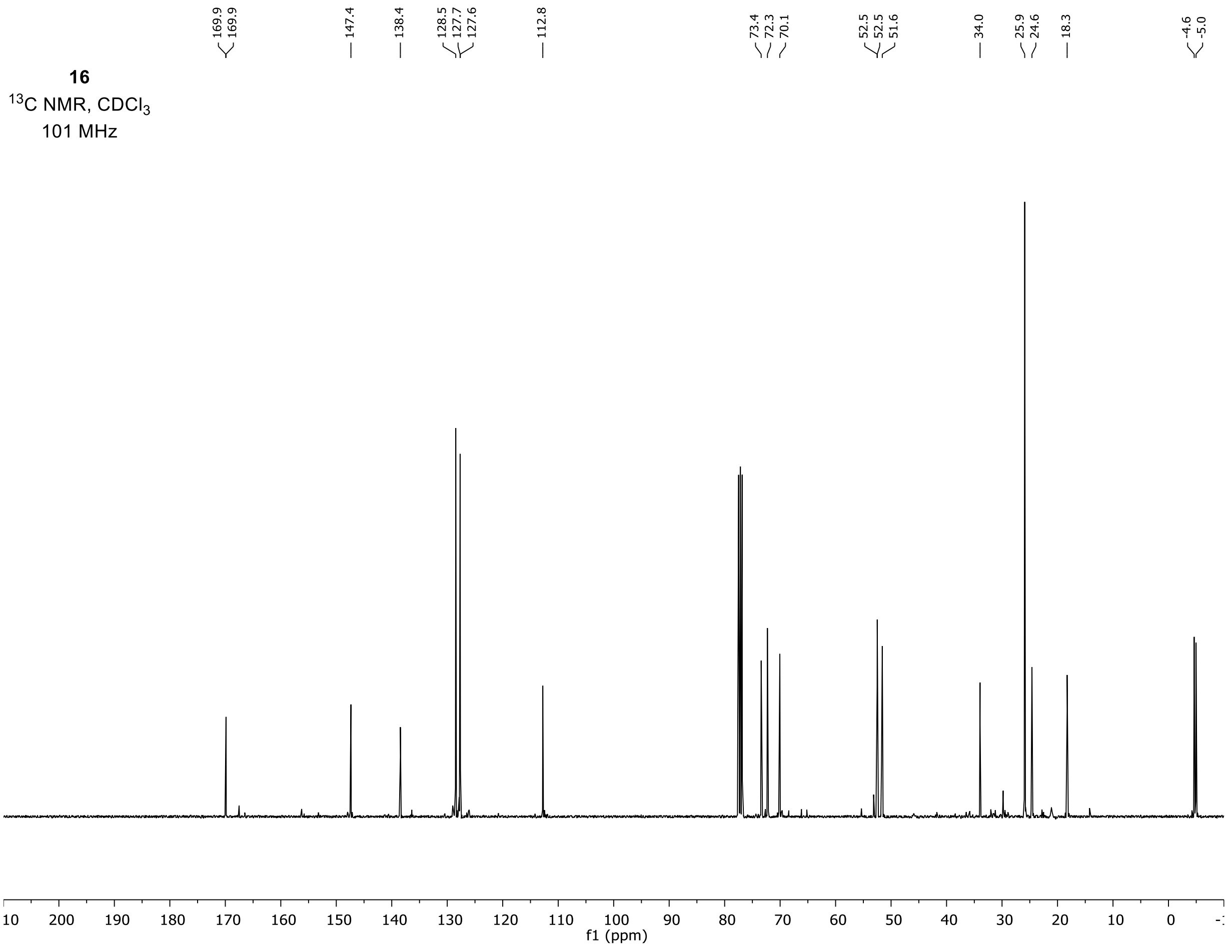

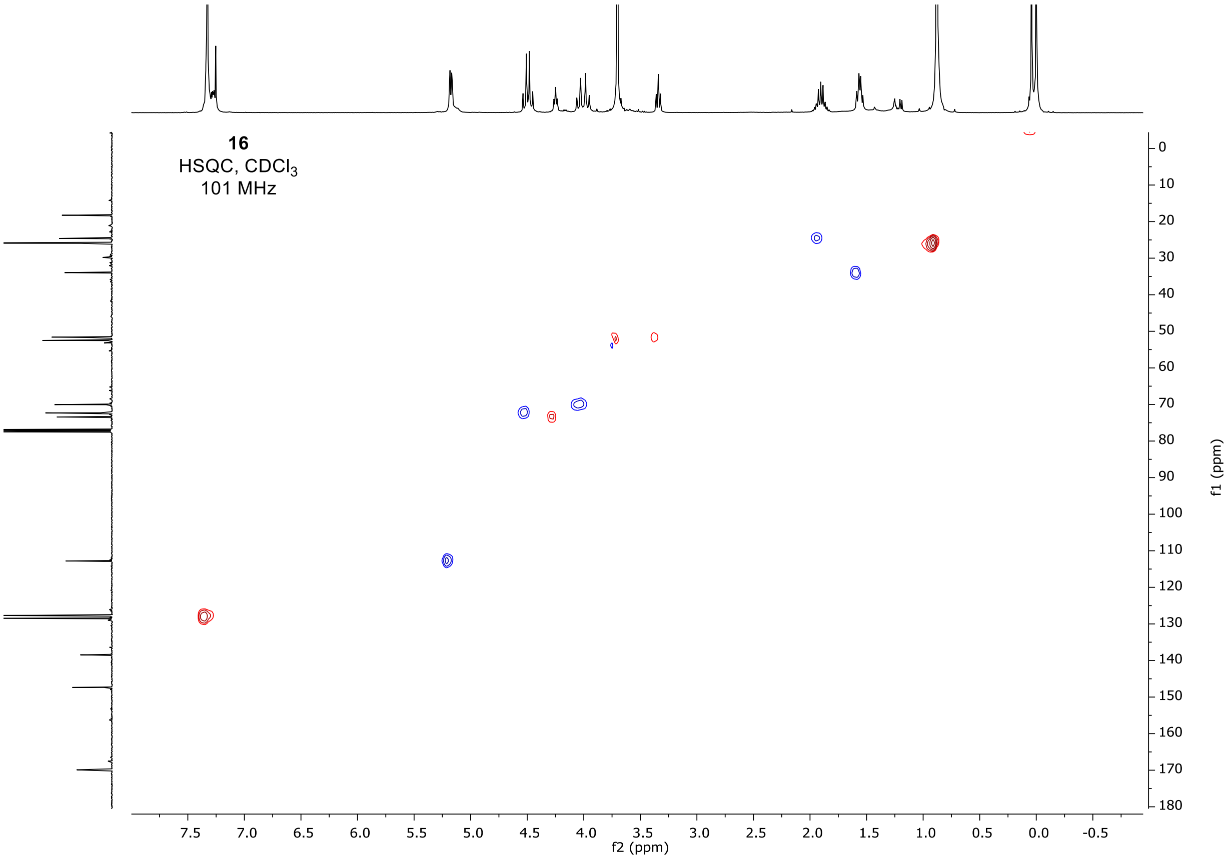
16
HSQC, CDCl3
101 MHz
f2 (ppm)
f1 (ppm)

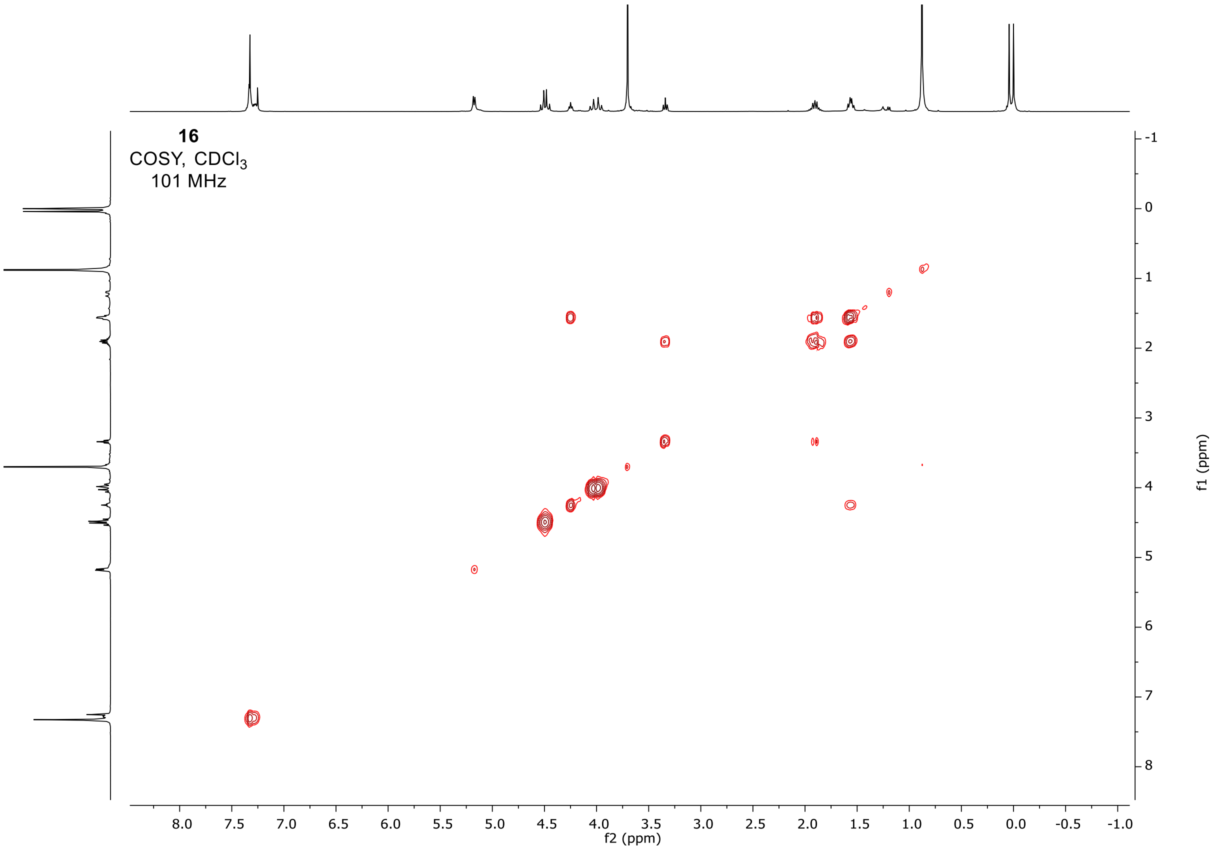
16
COSY, CDCl3
101 MHz
f2 (ppm)
f1 (ppm)

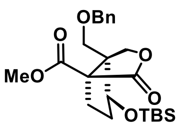

19
^{1}H NMR, $CDCl_3$
600 MHz

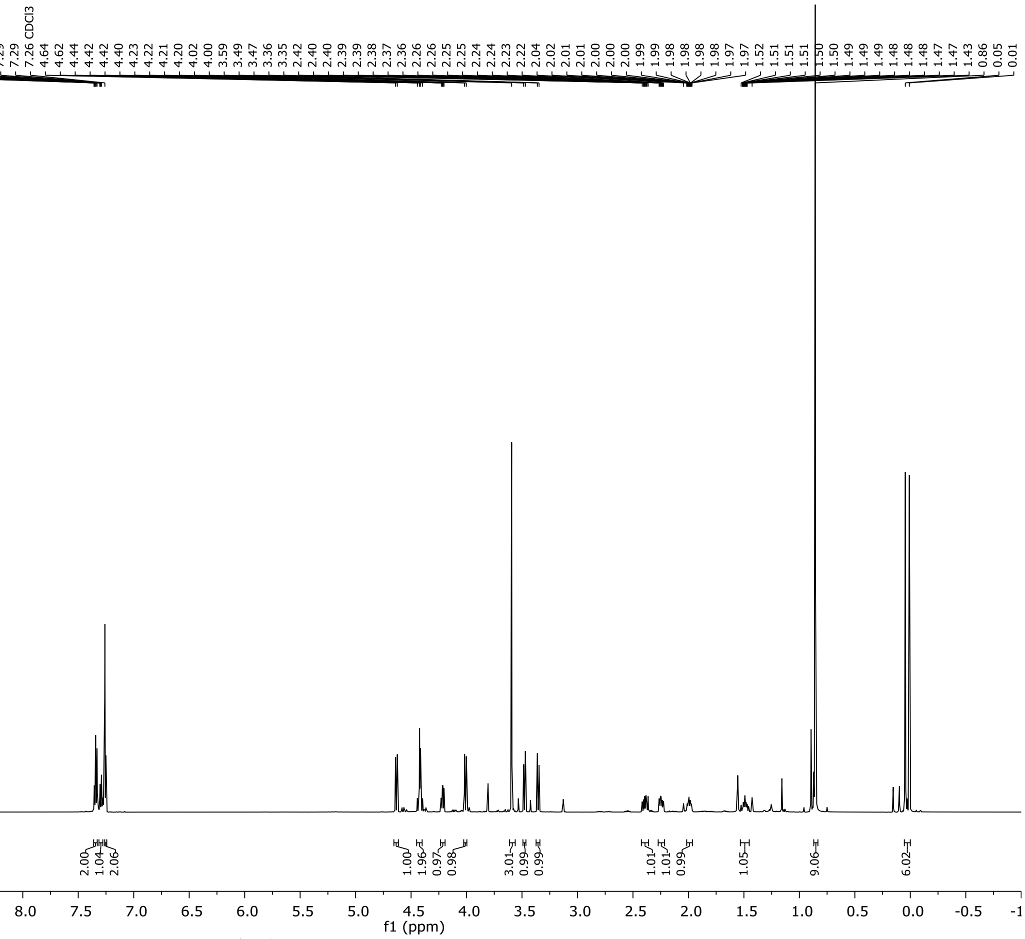

19
^{13}C NMR, $CDCl_3$
151 MHz

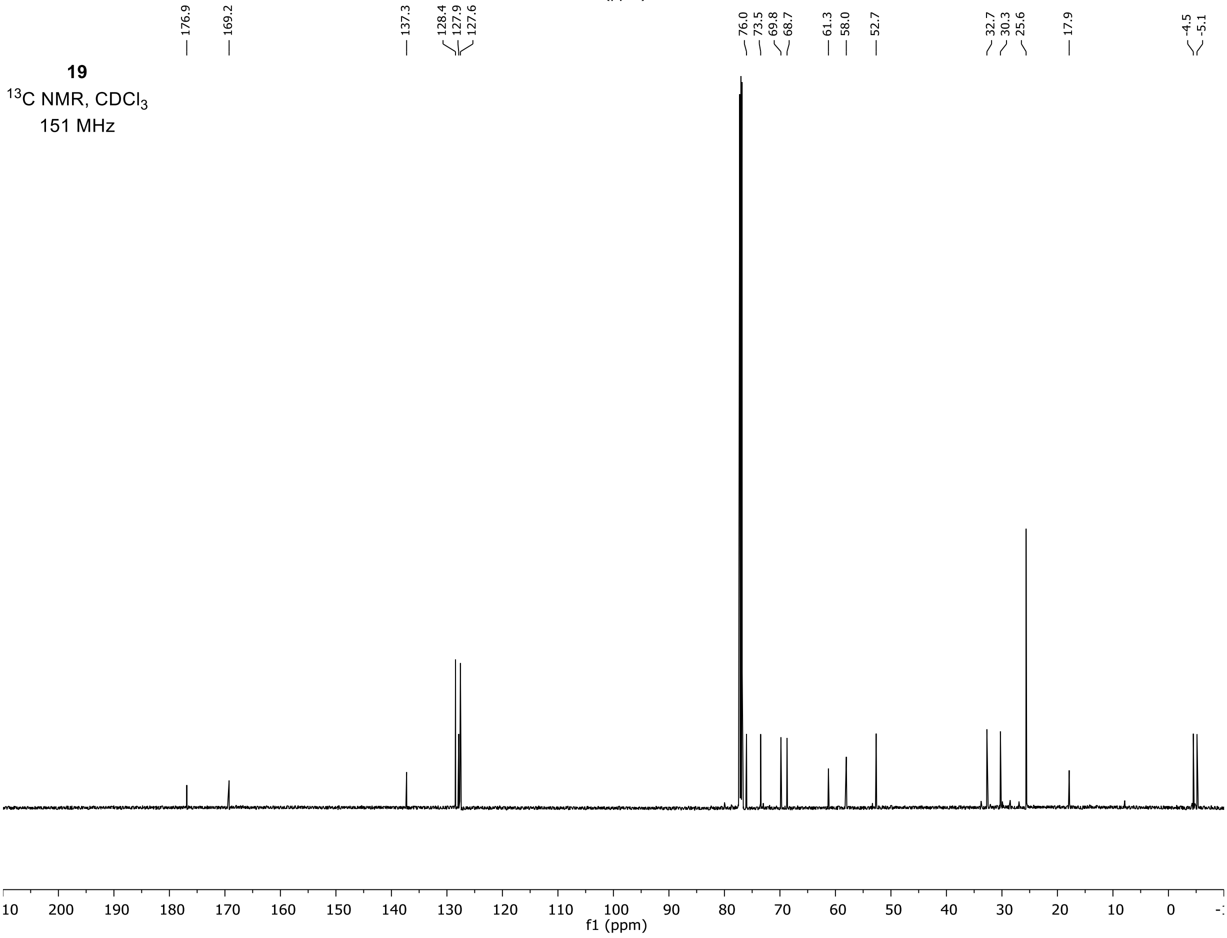

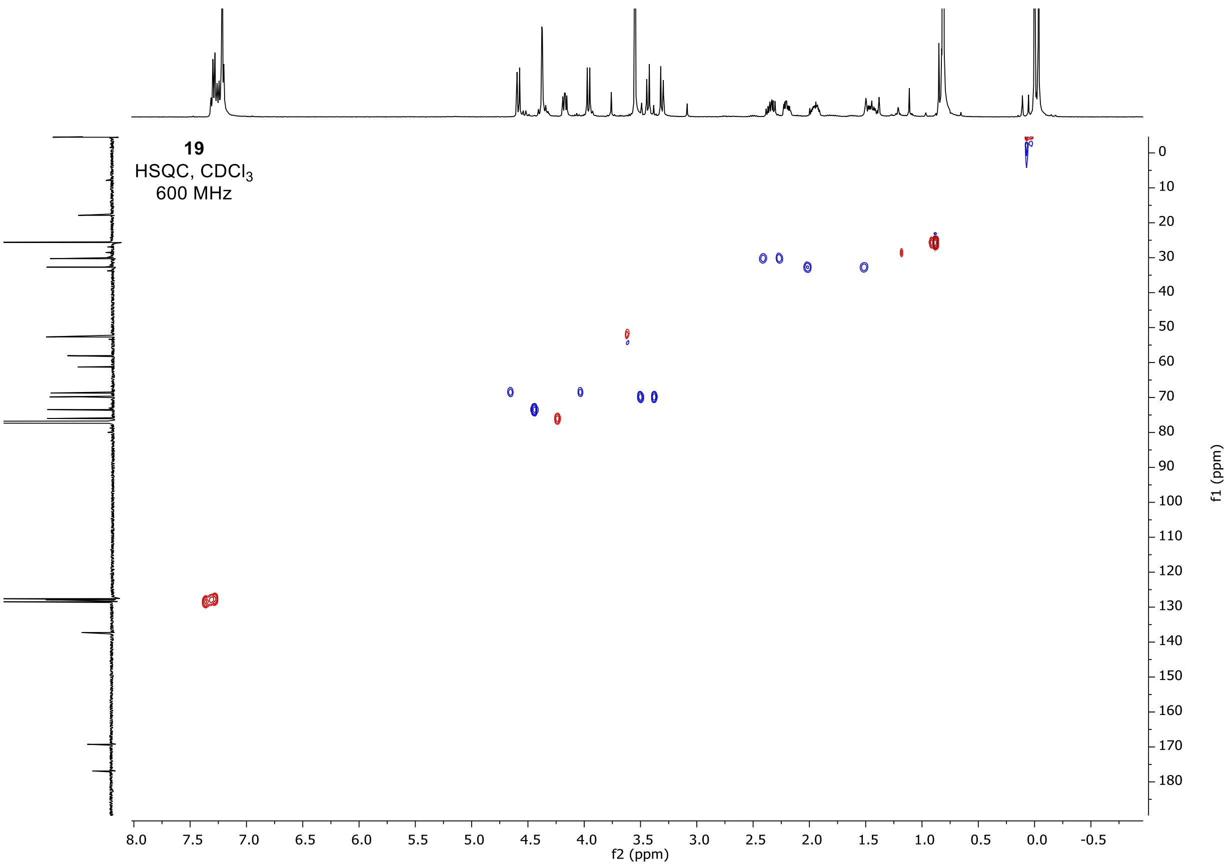
19
HSQC, CDCl3
600 MHz
f2 (ppm)
f1 (ppm)

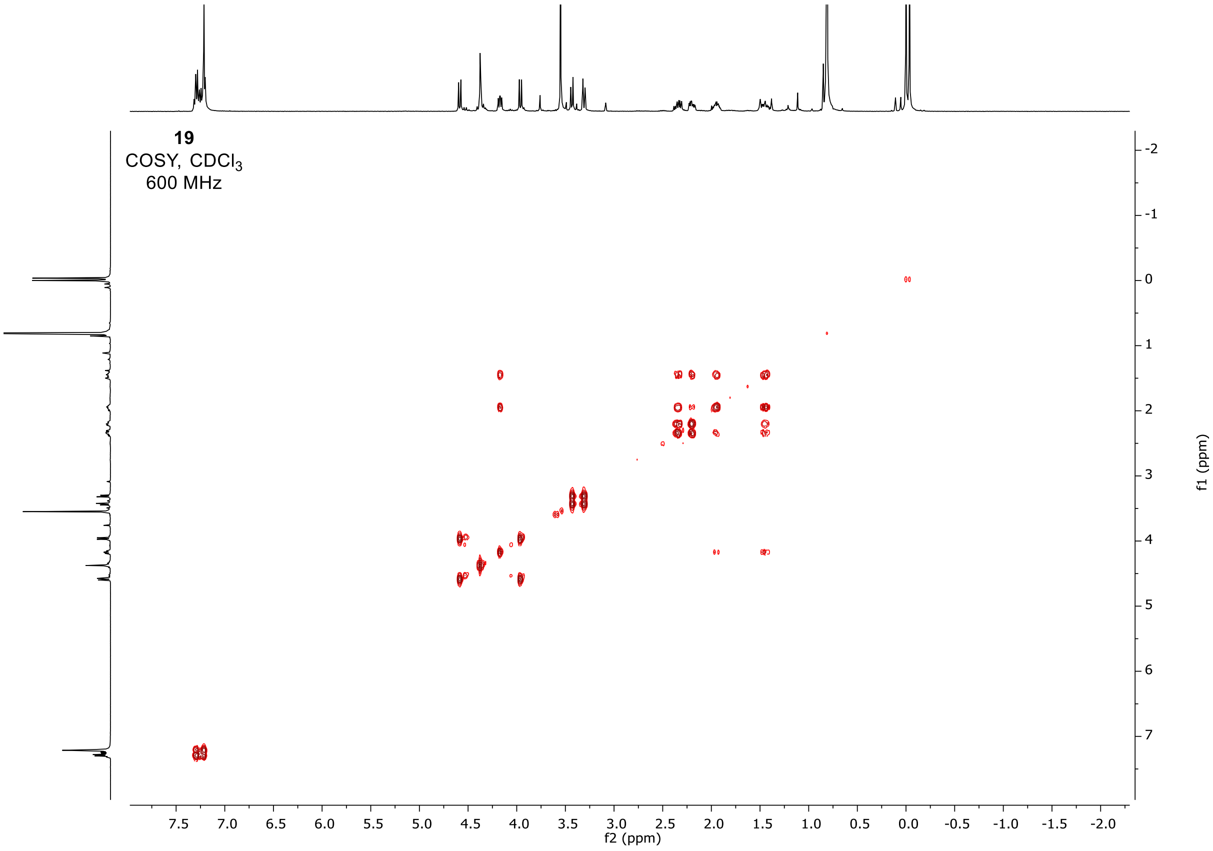
19
COSY, CDCl3
600 MHz
f2 (ppm)
f1 (ppm)

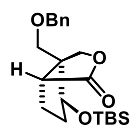

20
^{1}H NMR, $CDCl_3$
400 MHz

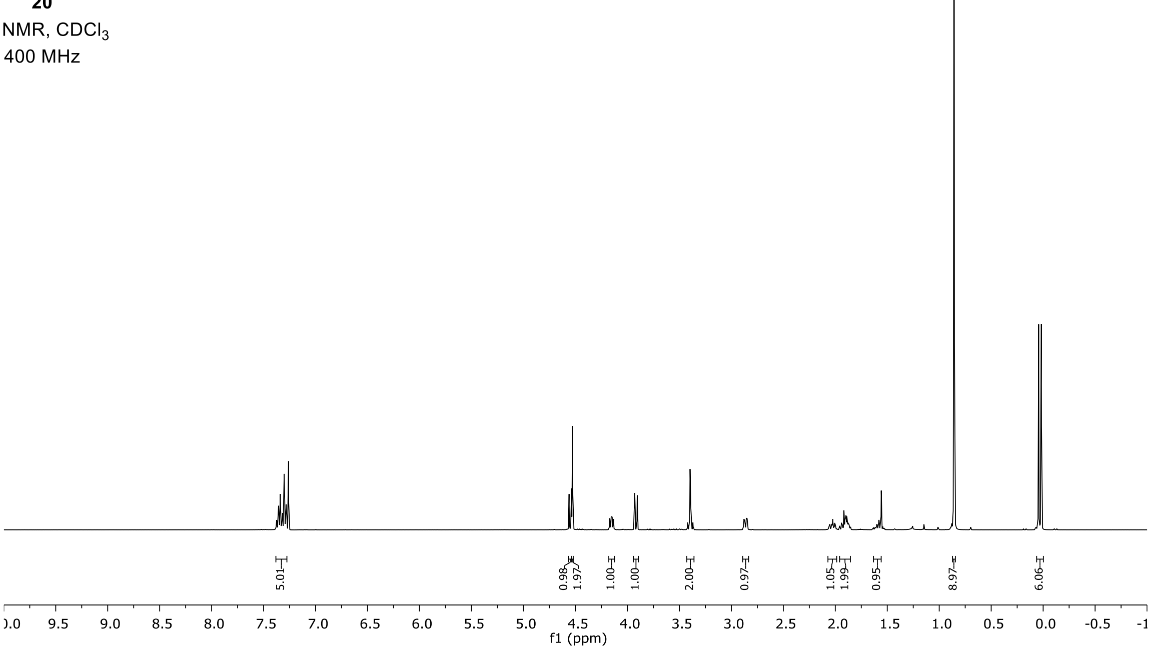

20
^{13}C NMR, $CDCl_3$
101 MHz

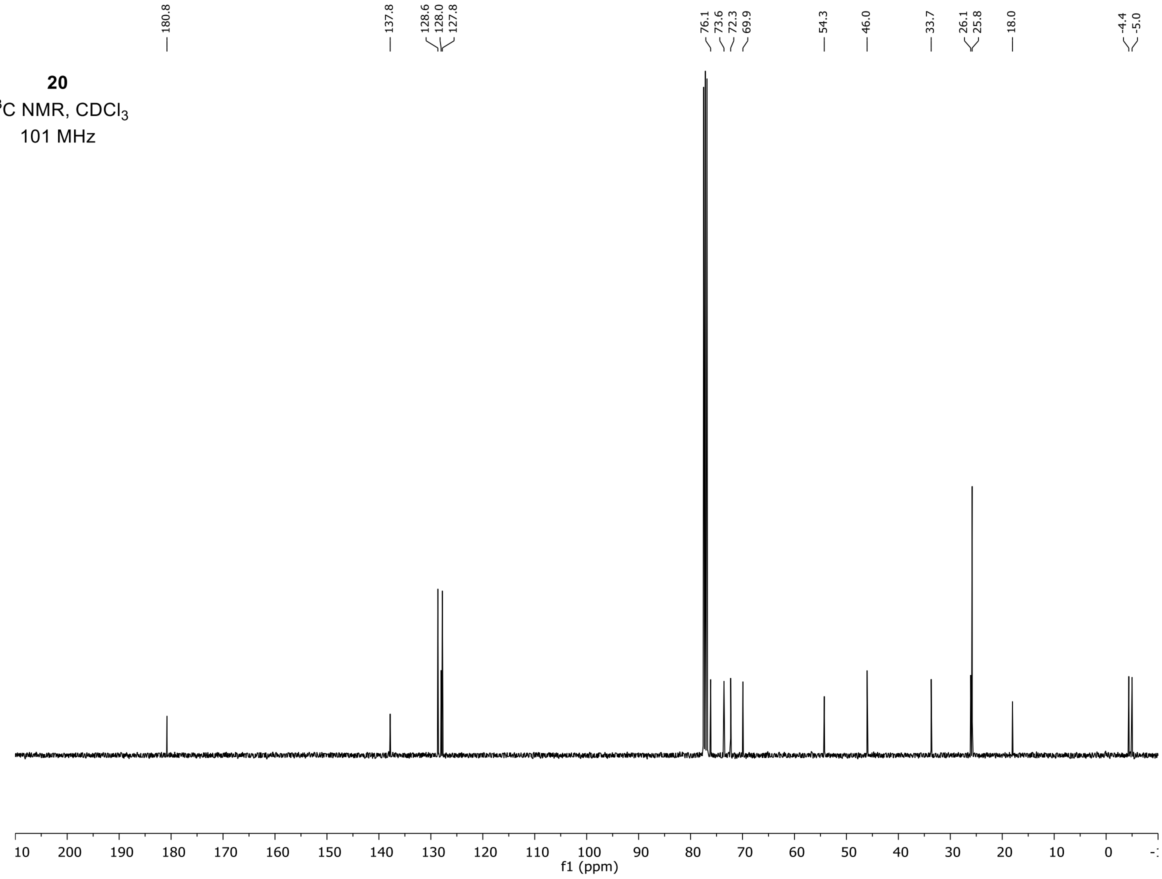

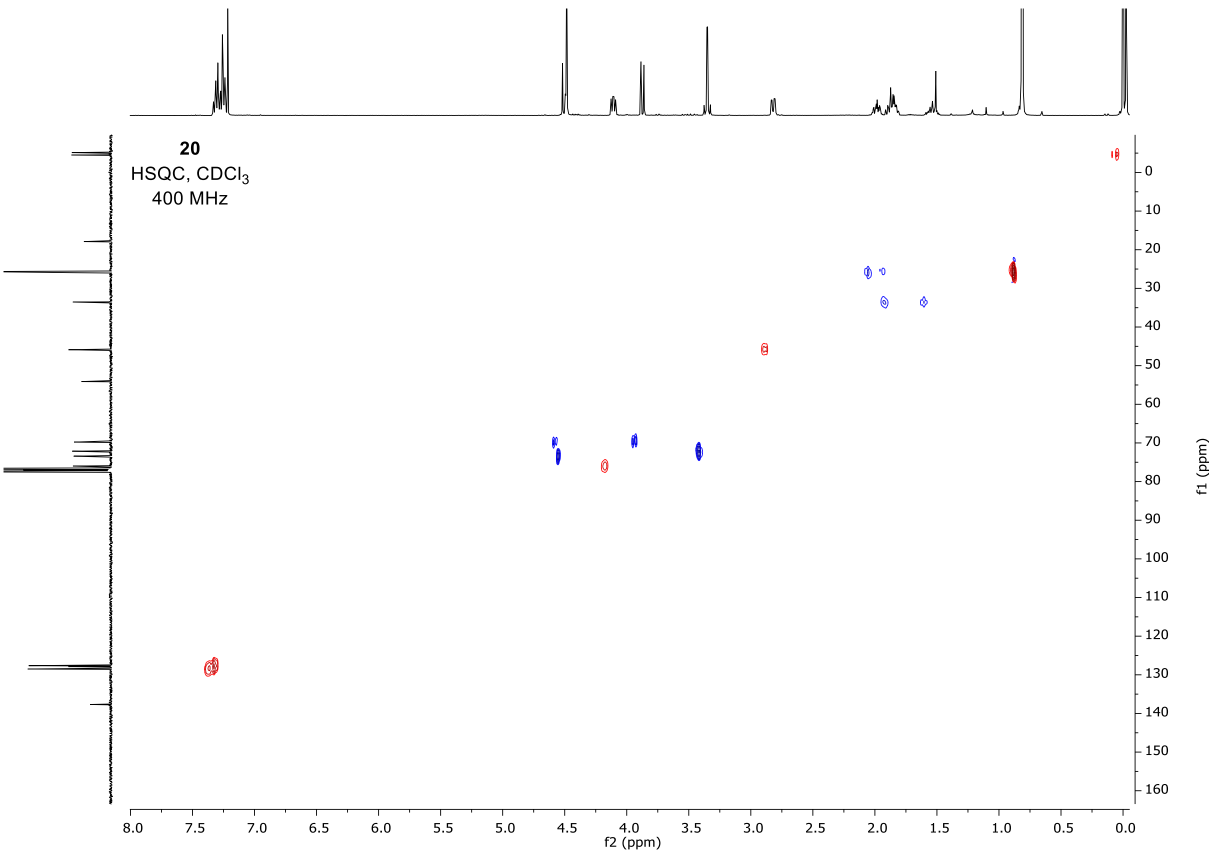
20
HSQC, $CDCl_3$
400 MHz
f2 (ppm)
f1 (ppm)

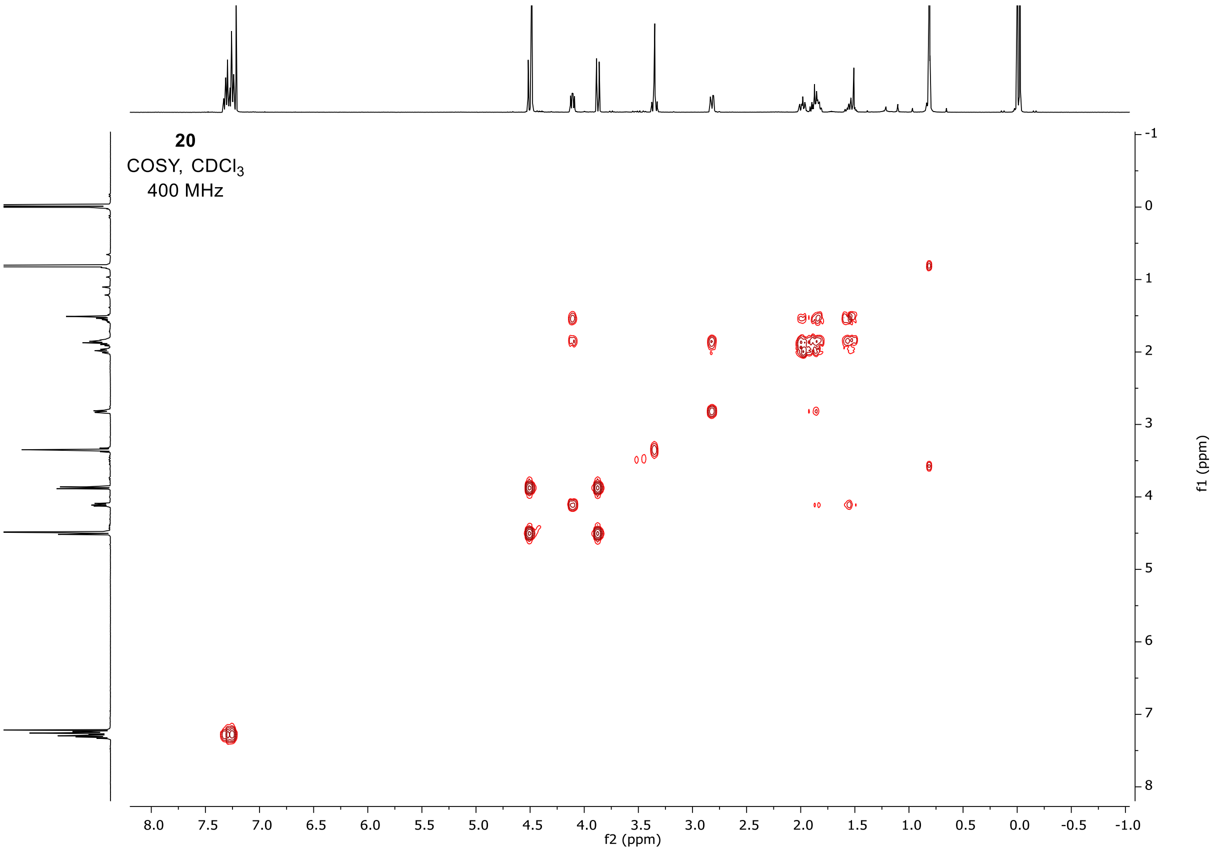
20
COSY, $CDCl_3$
400 MHz
f2 (ppm)
f1 (ppm)

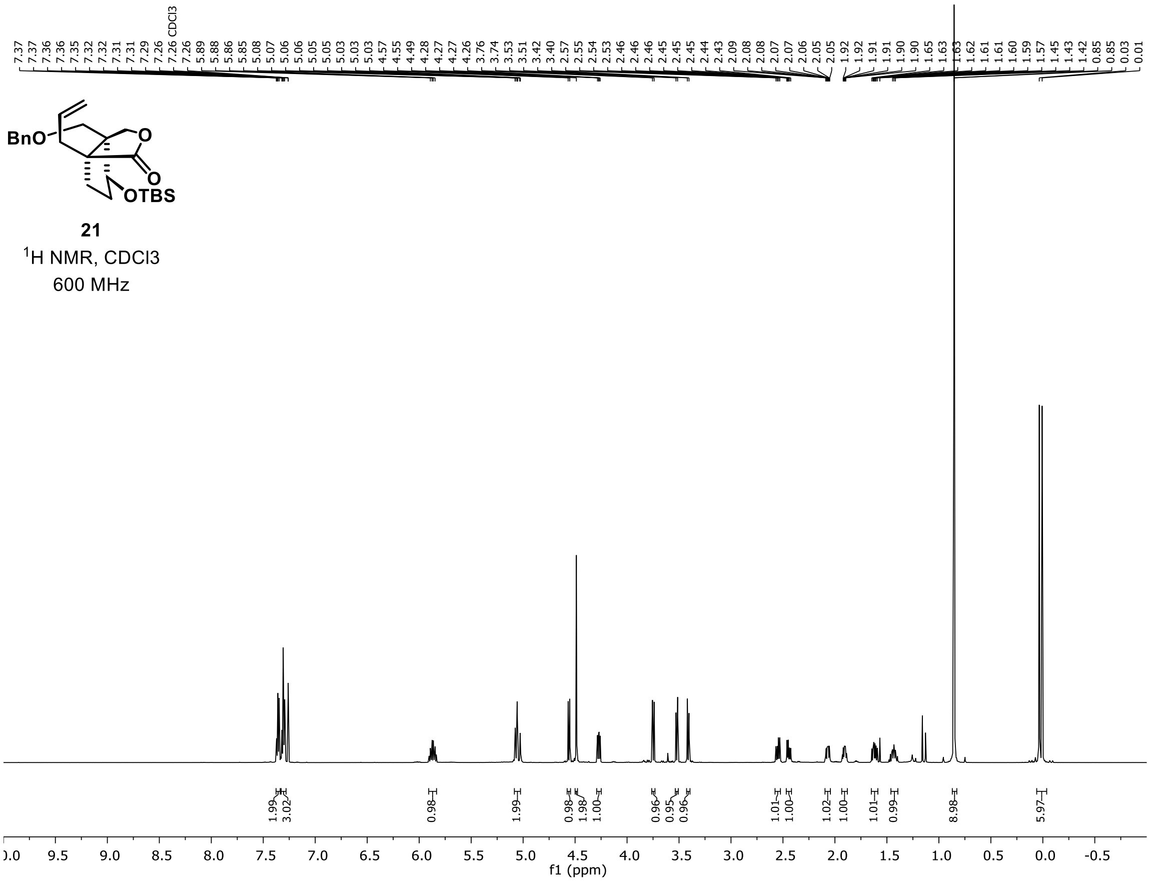
BnO
OTBS
O
O
21
¹H NMR, CDCl3
600 MHz
f1 (ppm)

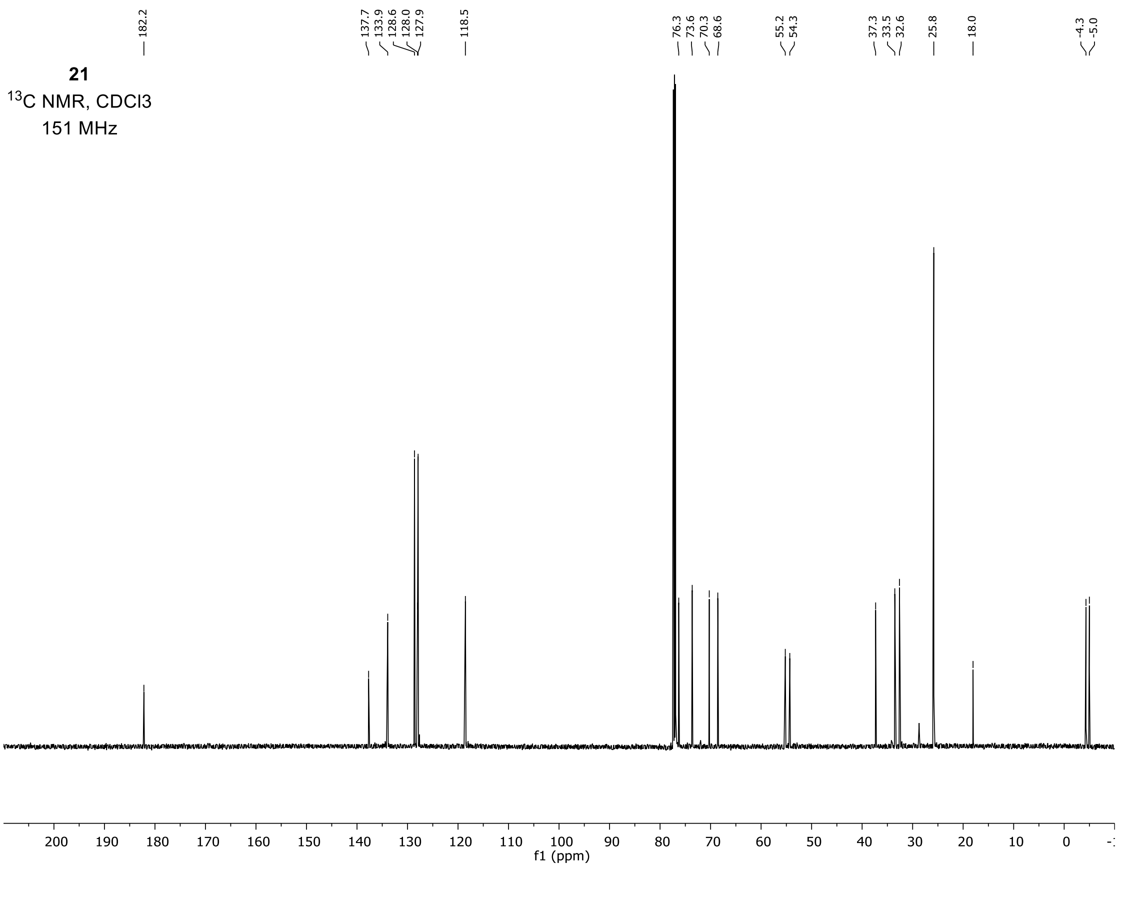
21
¹³C NMR, CDCl3
151 MHz
f1 (ppm)

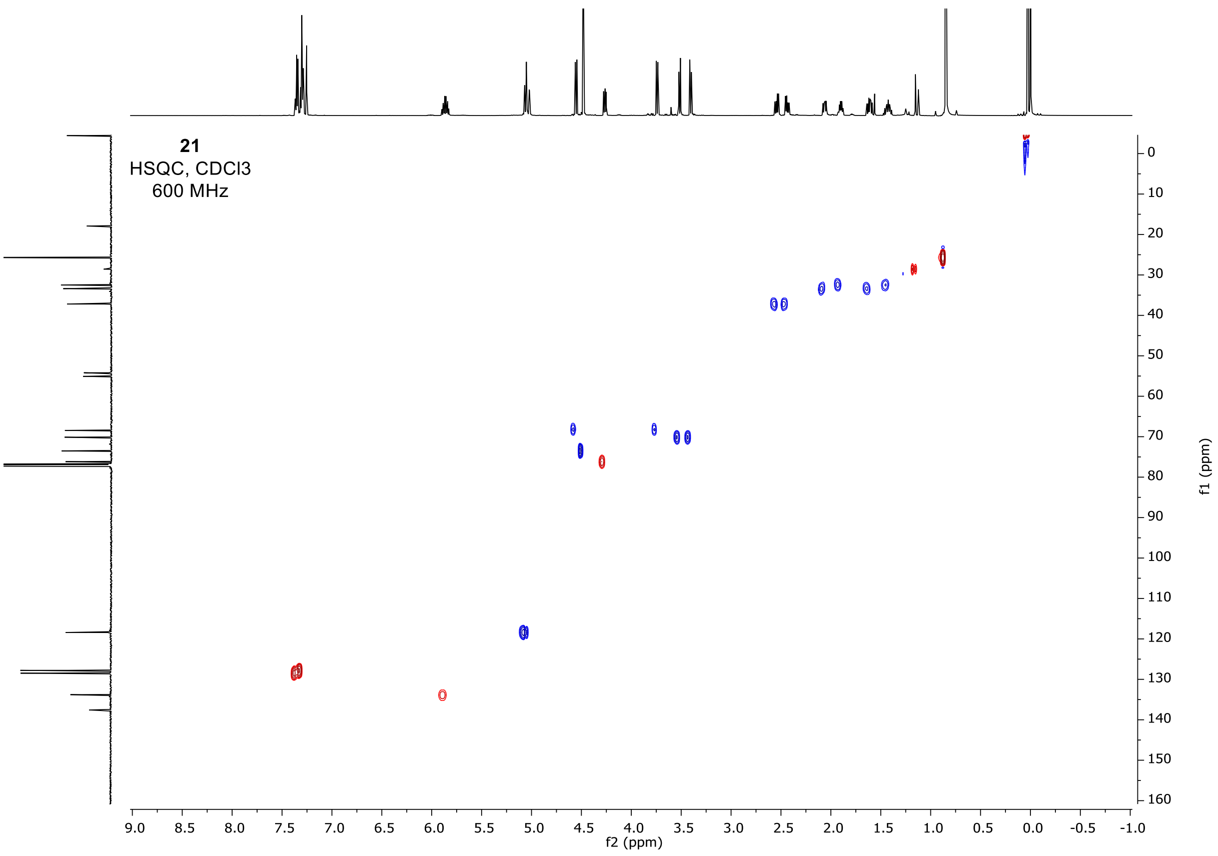
21
HSQC, CDCl3
600 MHz
f2 (ppm)
f1 (ppm)

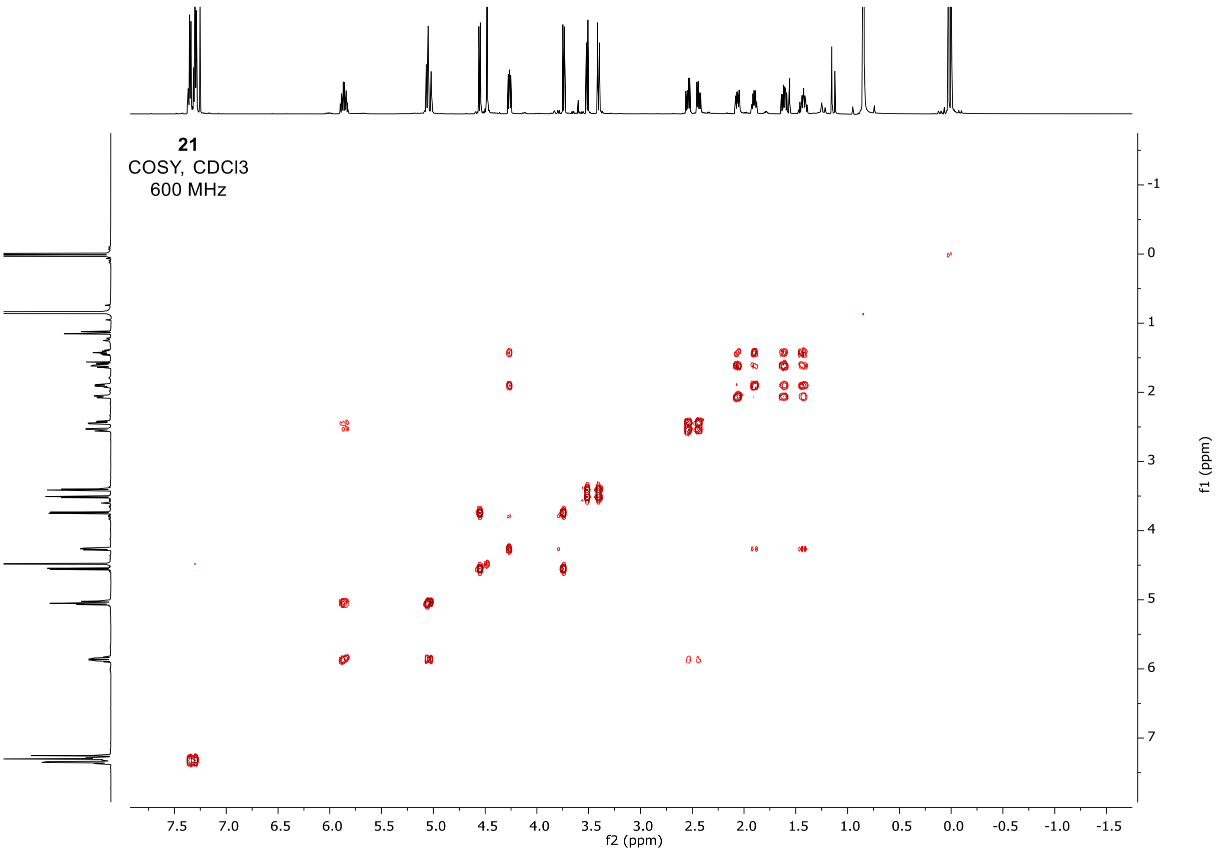
21
COSY, CDCl3
600 MHz
f2 (ppm)
f1 (ppm)

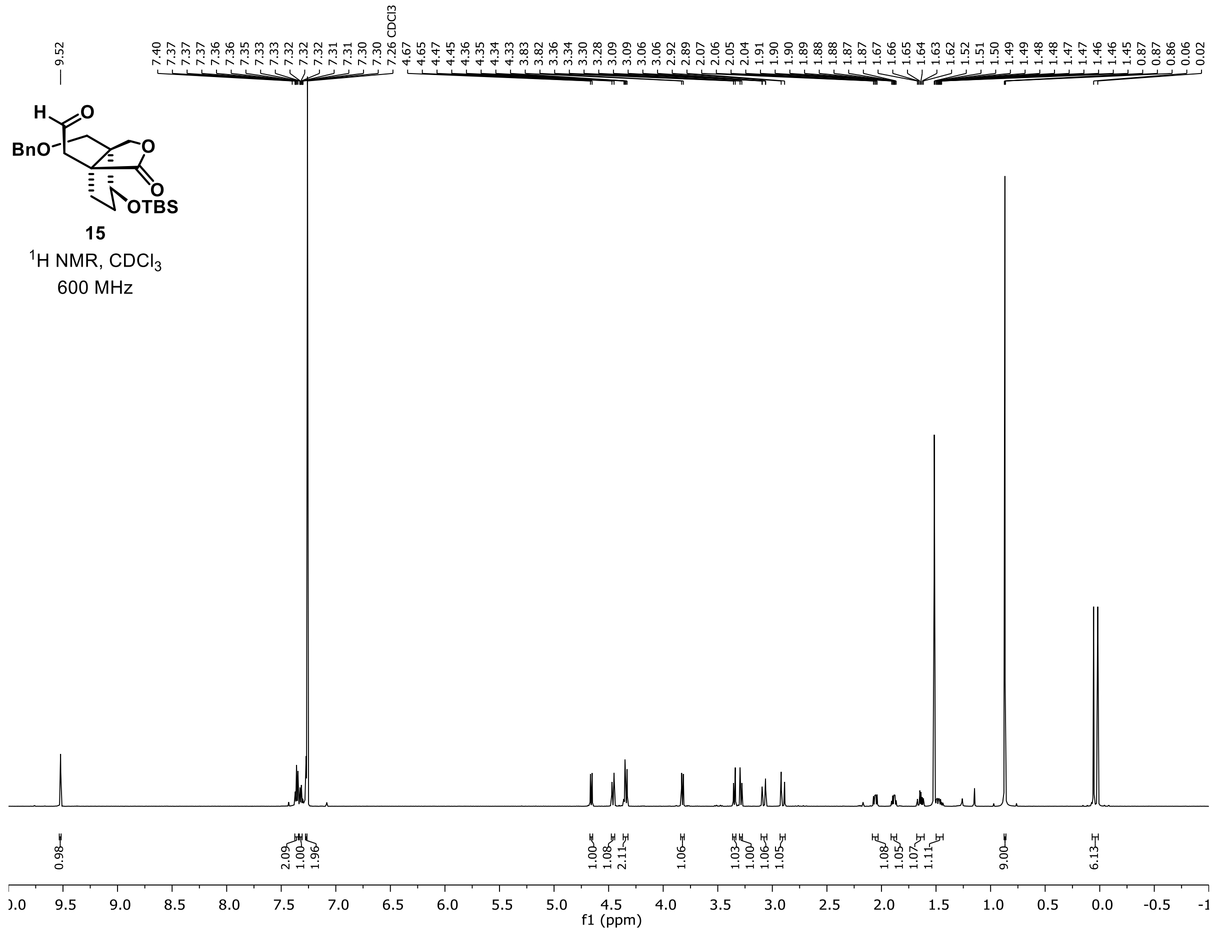

9.52
7.40 7.37 7.37 7.37 7.36 7.36 7.35 7.33 7.33 7.32 7.32 7.32 7.31 7.31 7.30 7.30
7.26 CDCl3
4.67 4.65 4.47 4.45 4.36 4.35 4.34 4.33 3.83 3.82 3.36 3.34 3.30 3.28 3.09 3.09 3.06 3.06 2.92 2.89 2.07 2.06 2.05 2.04 1.91 1.90 1.89 1.88 1.88 1.87 1.87 1.67 1.66 1.65 1.64 1.63 1.62 1.52 1.51 1.50 1.49 1.49 1.48 1.48 1.47 1.47 1.46 1.46 1.45 0.87 0.86 0.06 0.02
H
O
BnO
O
O
OTBS
15
^{1}H NMR, $CDCl_3$
600 MHz
0.98
2.09 1.00 1.96
1.00 1.08 2.11
1.06
1.03 1.00 1.06 1.05
1.08 1.05 1.07 1.11
9.00
6.13
f1 (ppm)

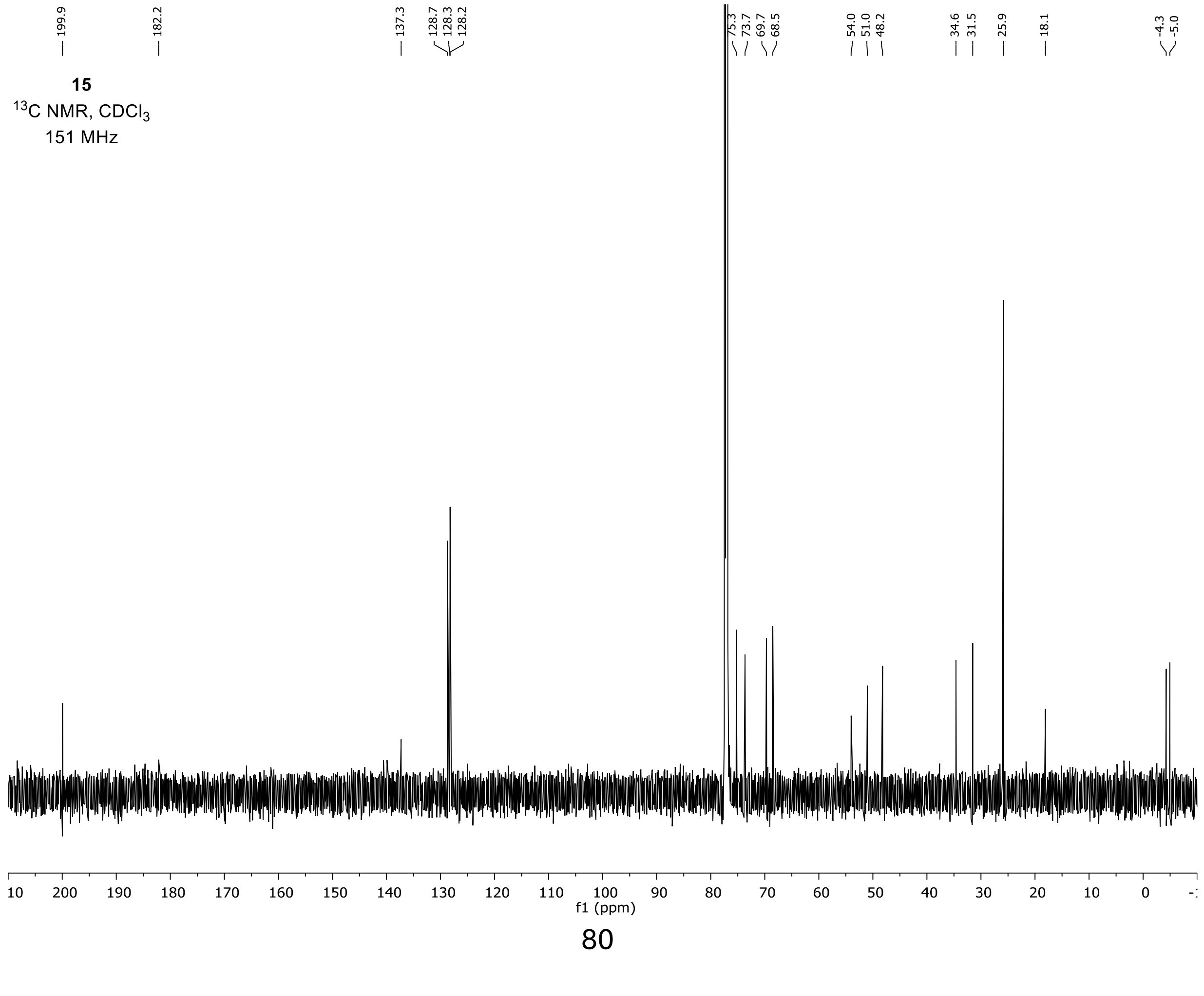

199.9
182.2
137.3
128.7 128.3 128.2
75.3 73.7 69.7 68.5
54.0 51.0 48.2
34.6 31.5
25.9
18.1
-4.3 -5.0
15
^{13}C NMR, $CDCl_3$
151 MHz
f1 (ppm)

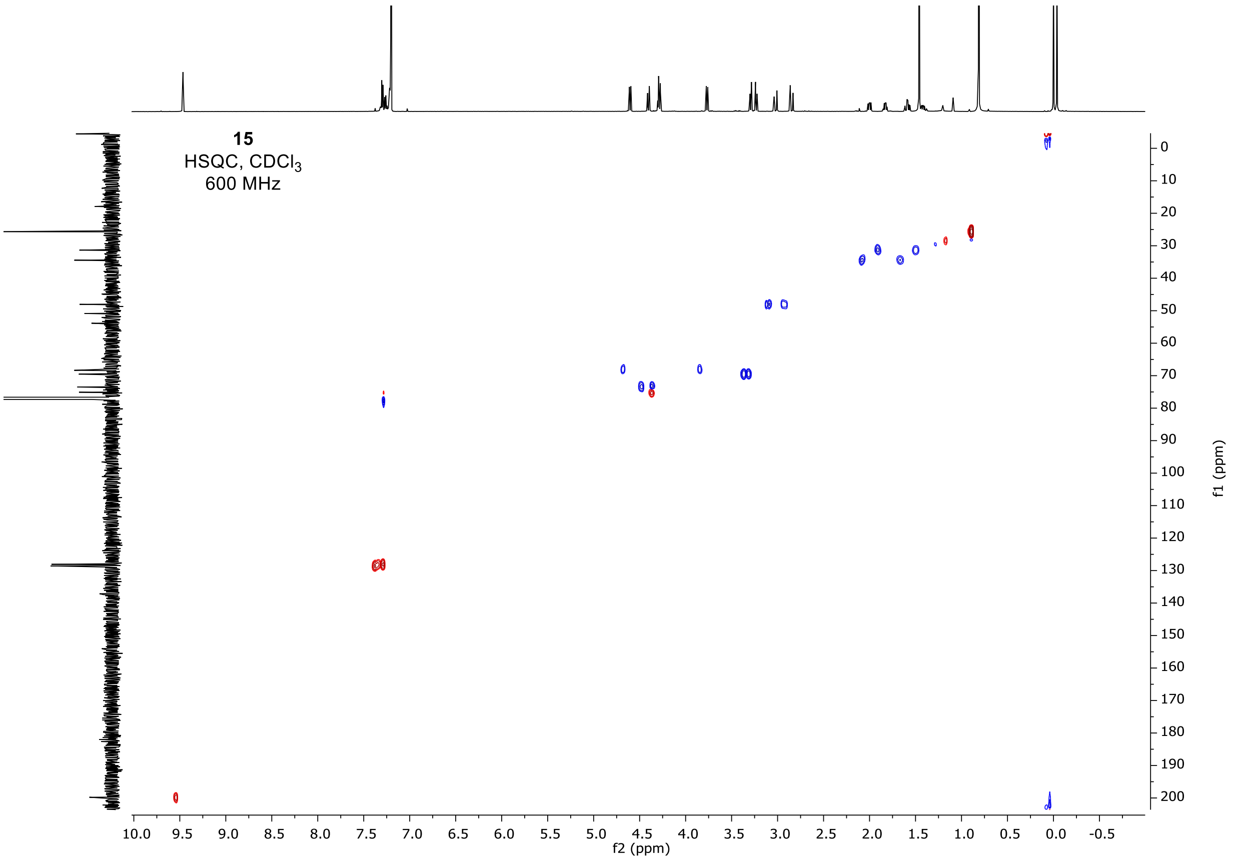
15
HSQC, CDCl3
600 MHz
f2 (ppm)
f1 (ppm)

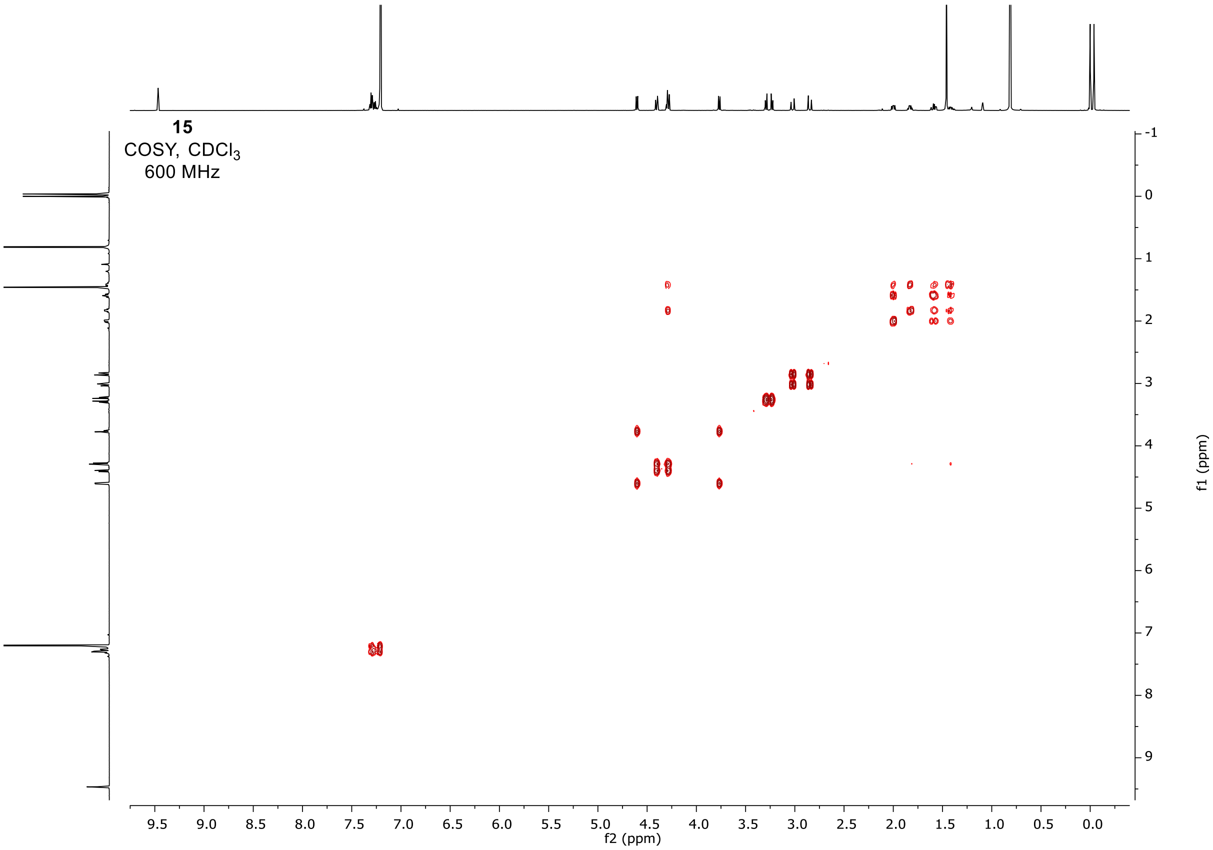
15
COSY, CDCl3
600 MHz
f2 (ppm)
f1 (ppm)

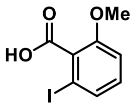

S7
^{1}H NMR, $CDCl_3$
400 MHz

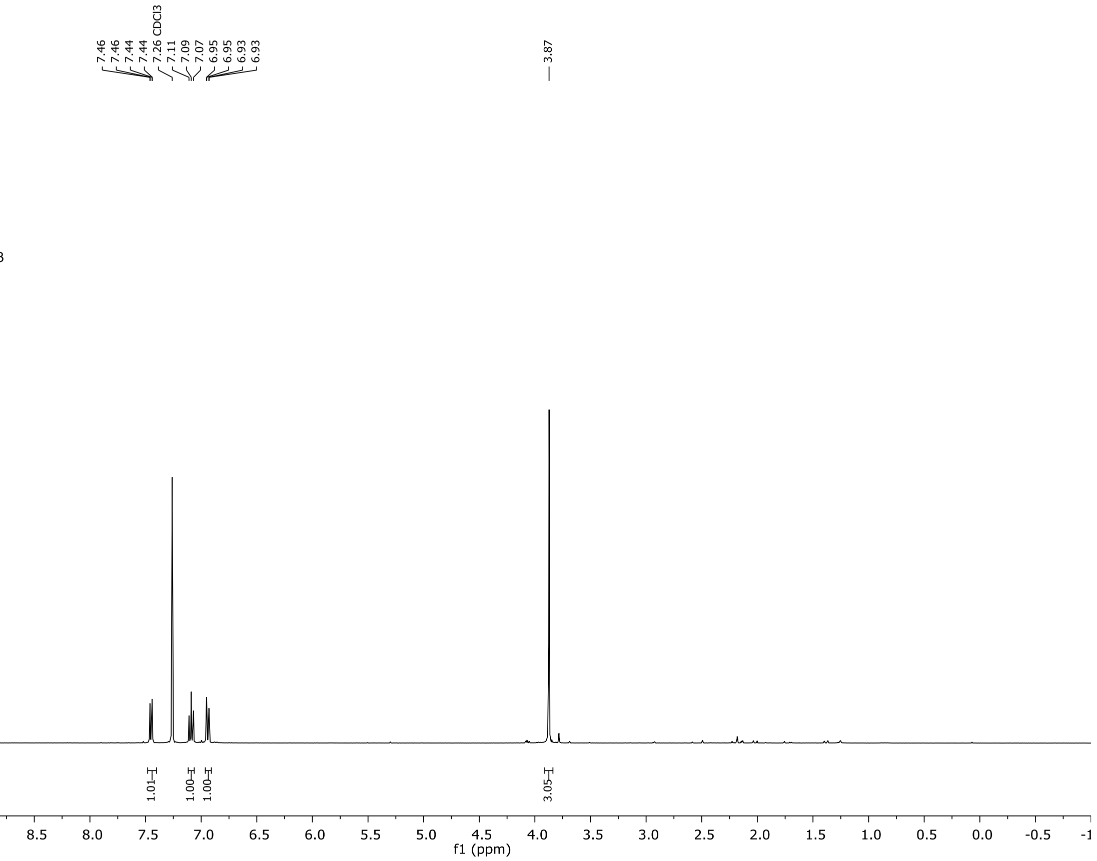

S7
^{13}C NMR, $CDCl_3$
101 MHz

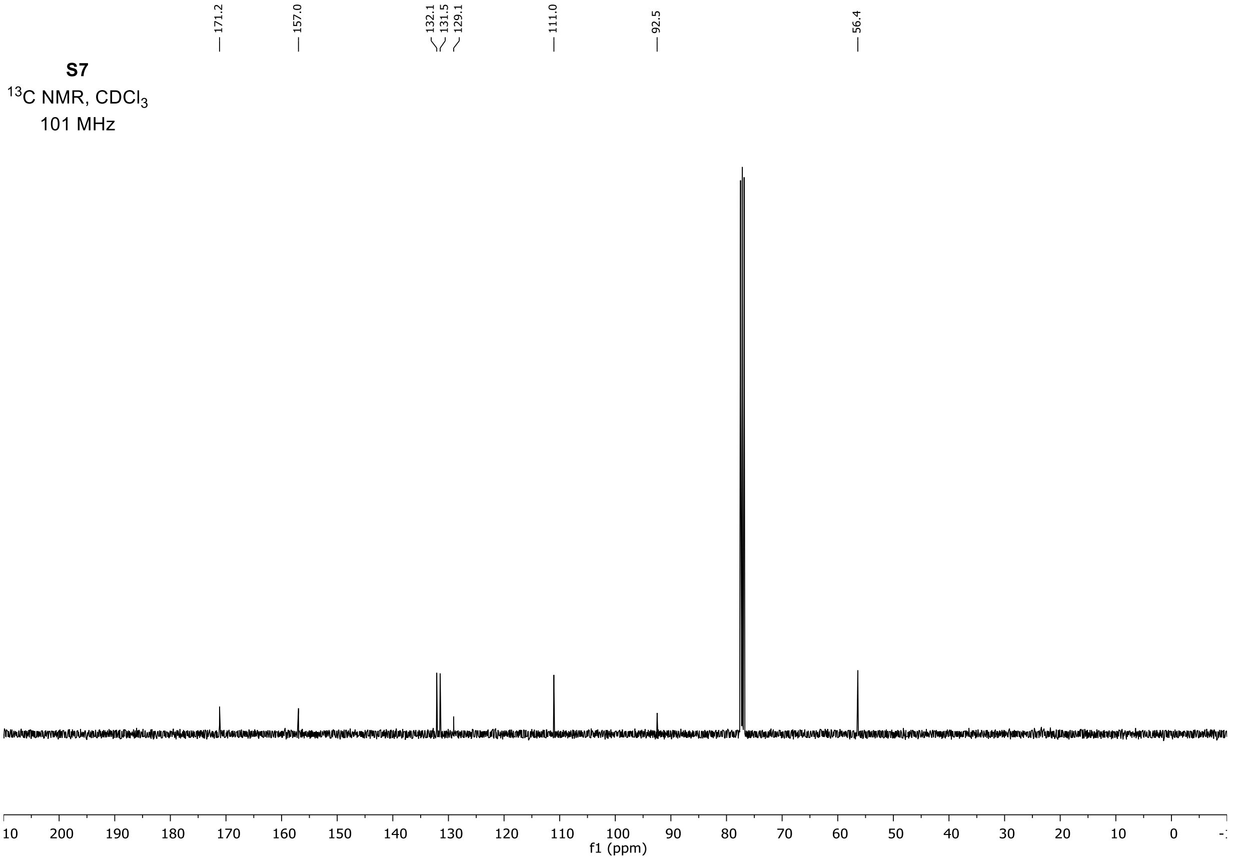

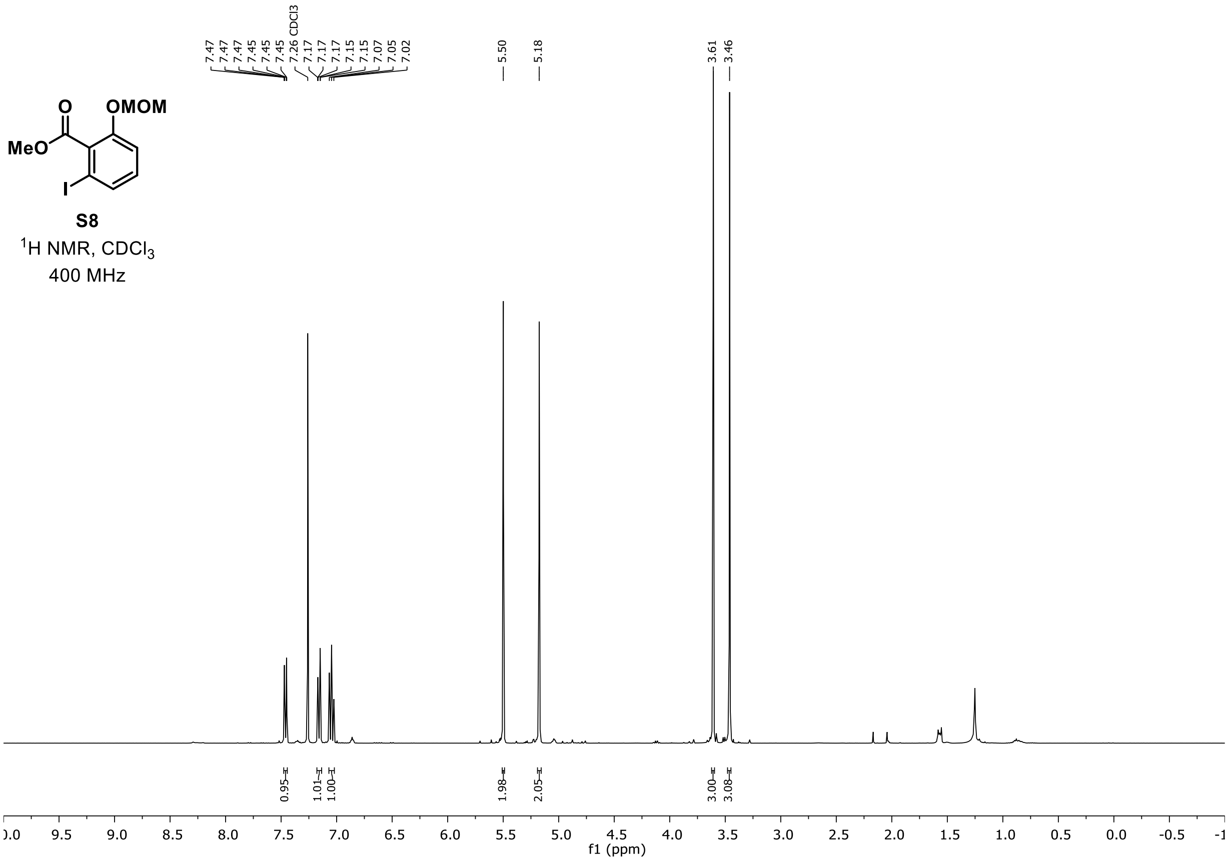
OMOM
MeO
O
I
S8
^{1}H NMR, $CDCl_3$
400 MHz
7.47
7.47
7.47
7.45
7.45
7.26 CDCl3
7.17
7.17
7.17
7.15
7.15
7.07
7.05
7.02
5.50
5.18
3.61
3.46
0.95
1.01
1.00
1.98
2.05
3.00
3.08
f1 (ppm)

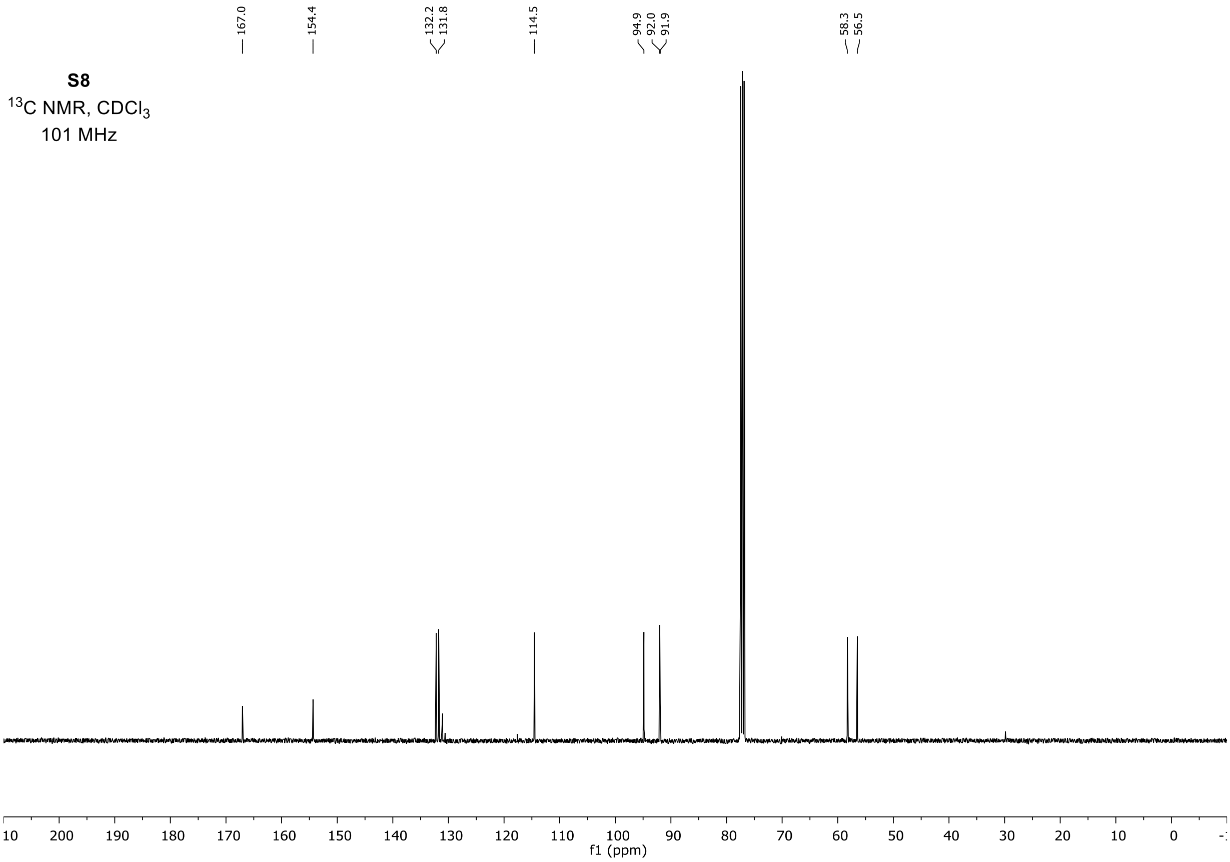
S8
^{13}C NMR, $CDCl_3$
101 MHz
167.0
154.4
132.2
131.8
114.5
94.9
92.0
91.9
58.3
56.5
f1 (ppm)

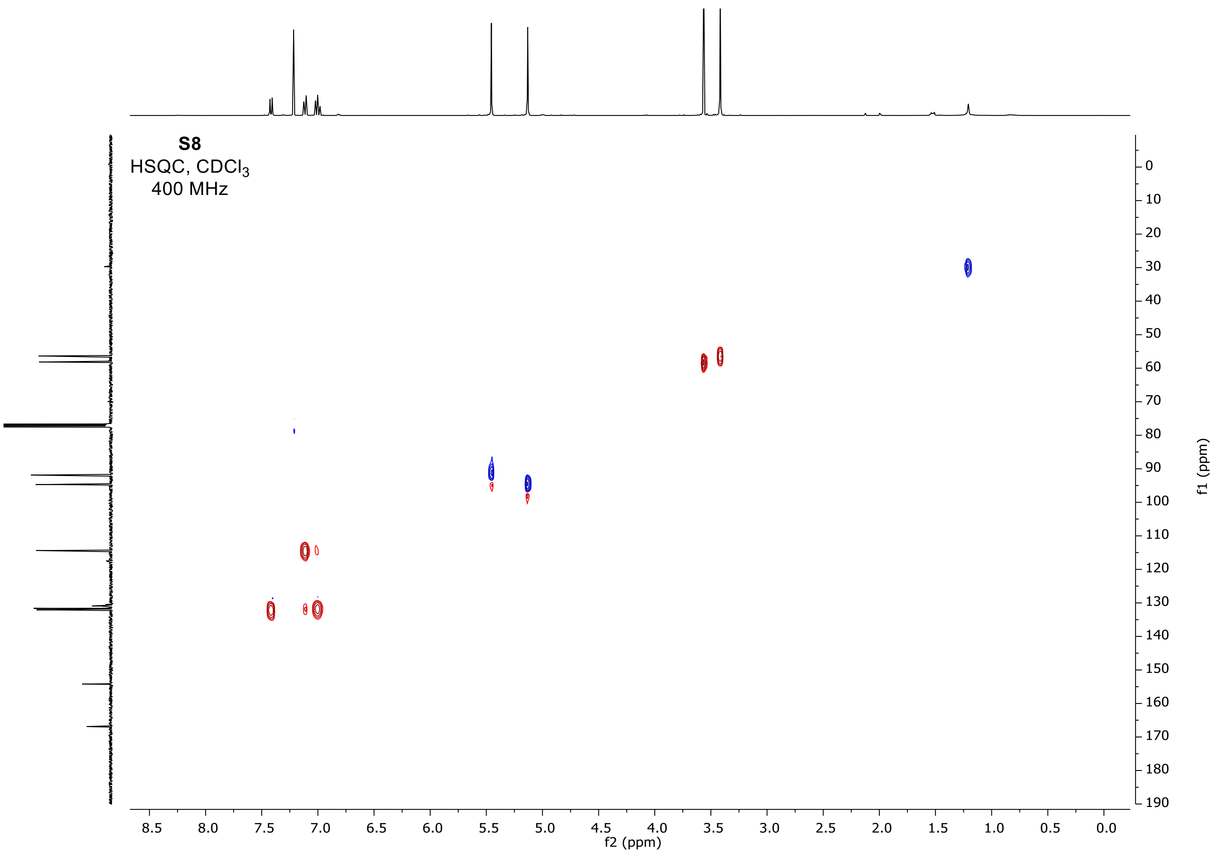
S8
HSQC, CDCl3
400 MHz
f2 (ppm)
f1 (ppm)

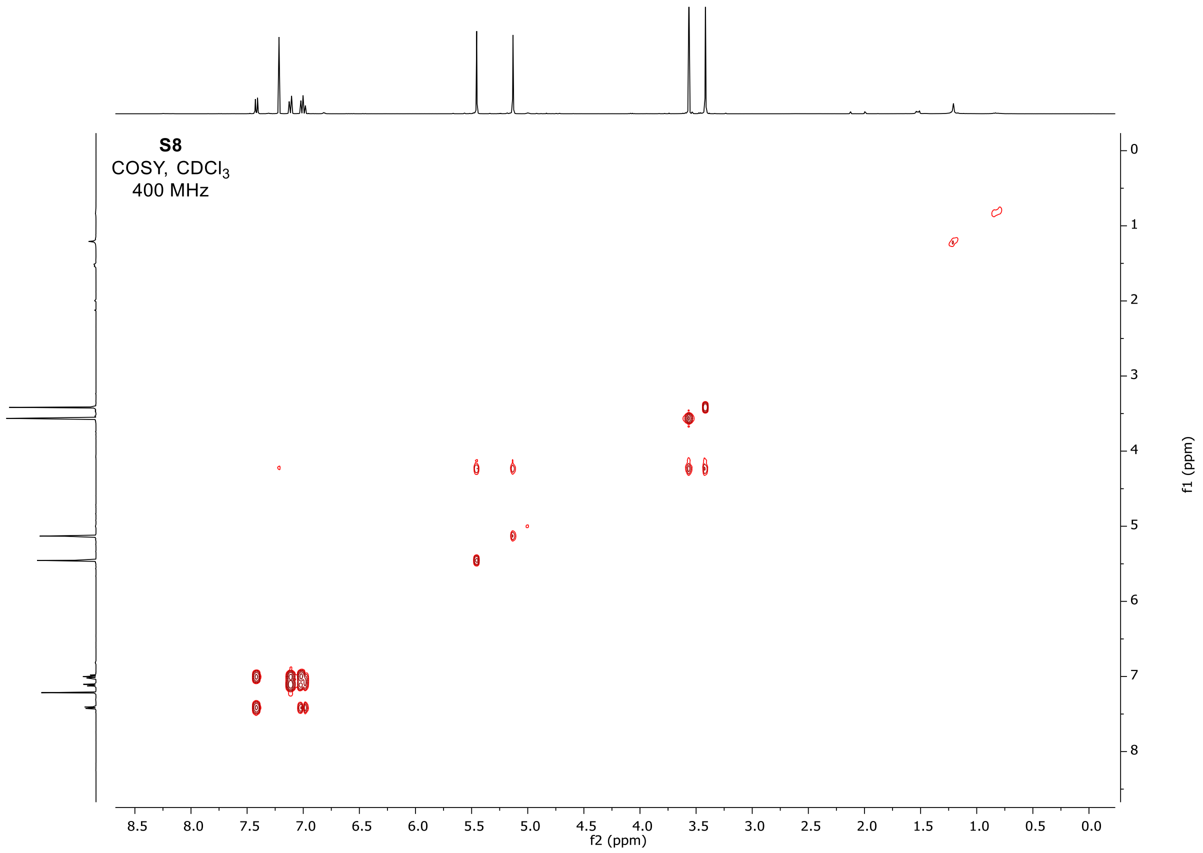
S8
COSY, CDCl3
400 MHz
f2 (ppm)
f1 (ppm)

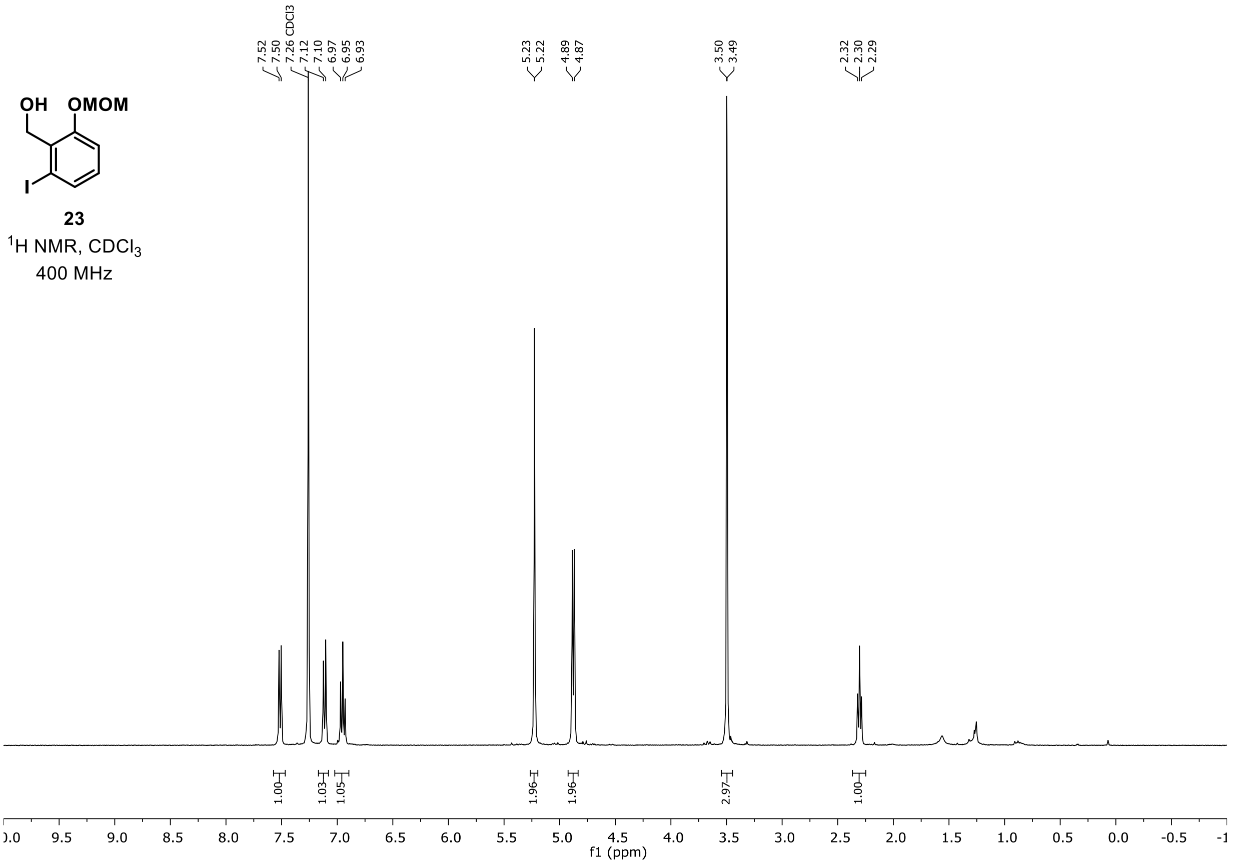

23
^{1}H NMR, $CDCl_3$
400 MHz

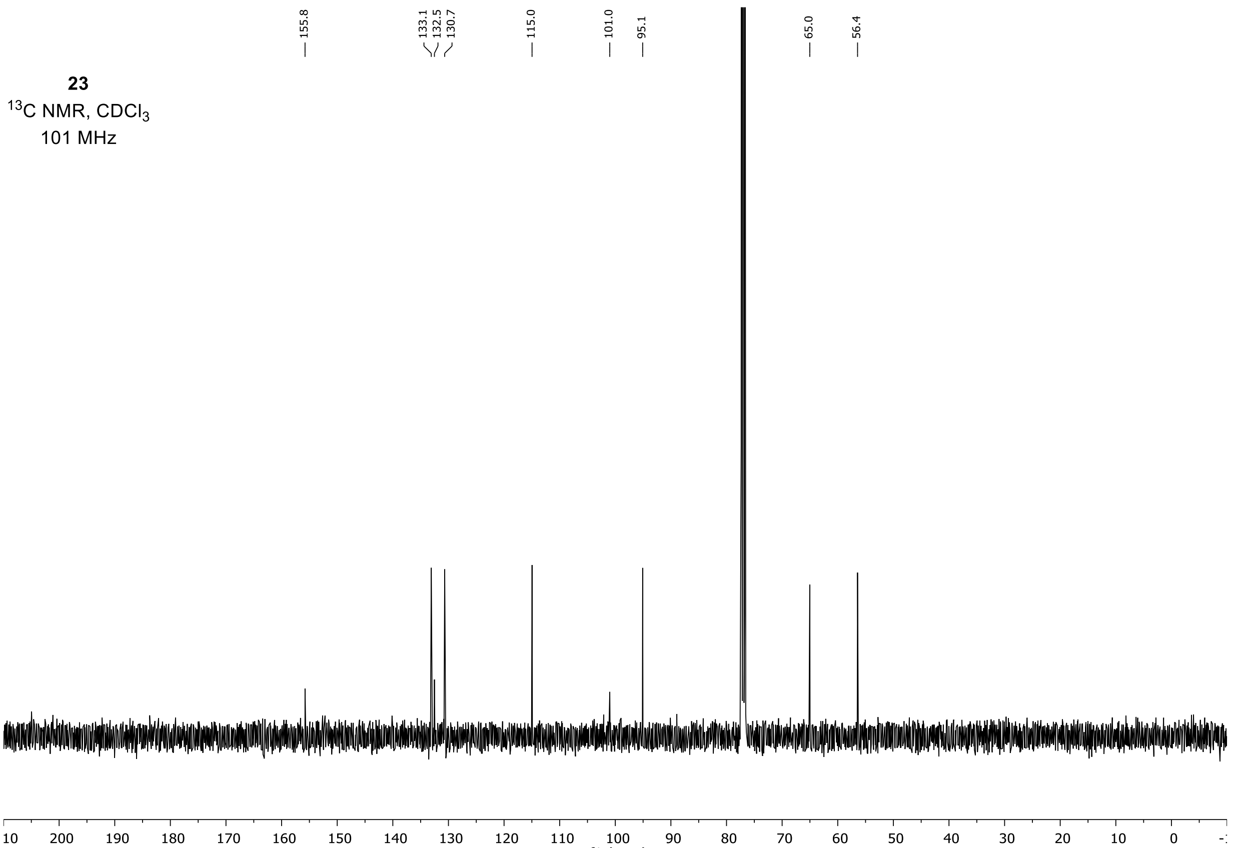

23
^{13}C NMR, $CDCl_3$
101 MHz

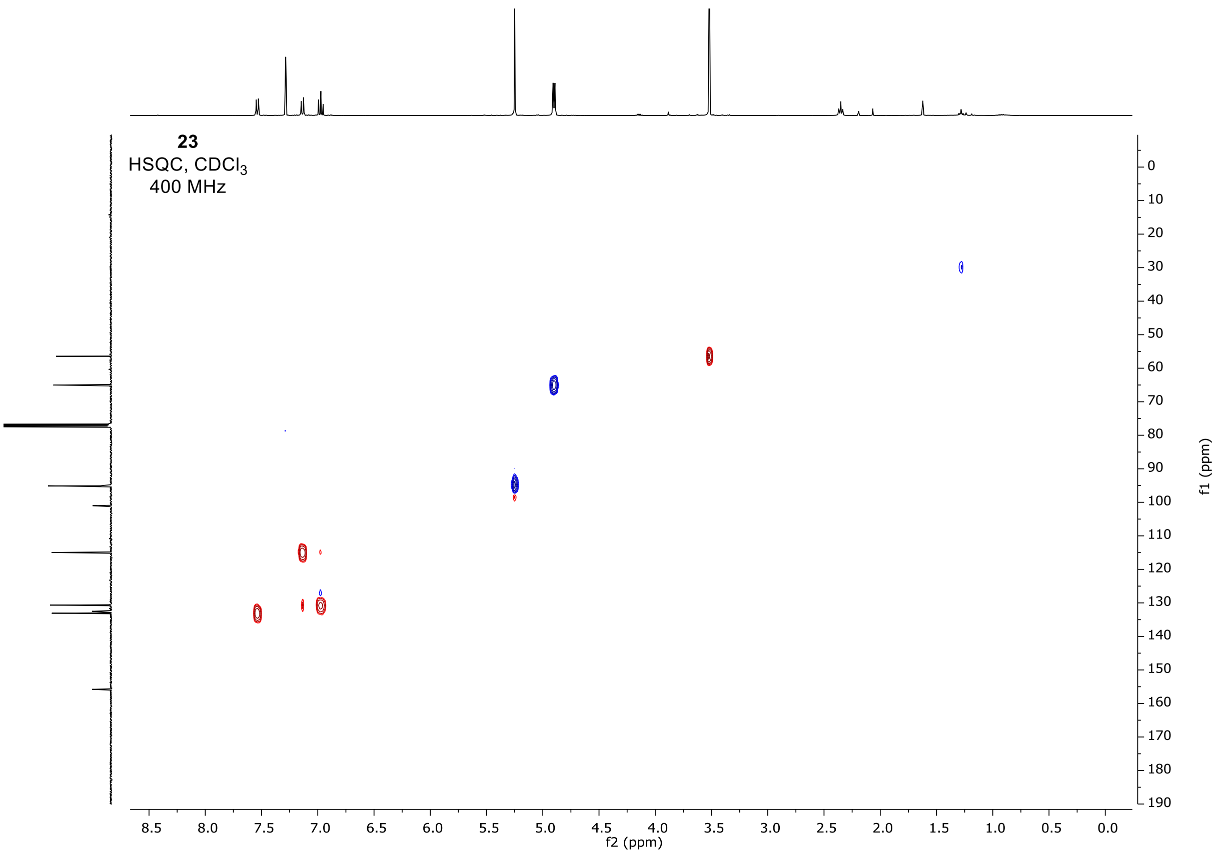
23
HSQC, CDCl3
400 MHz
f2 (ppm)
f1 (ppm)

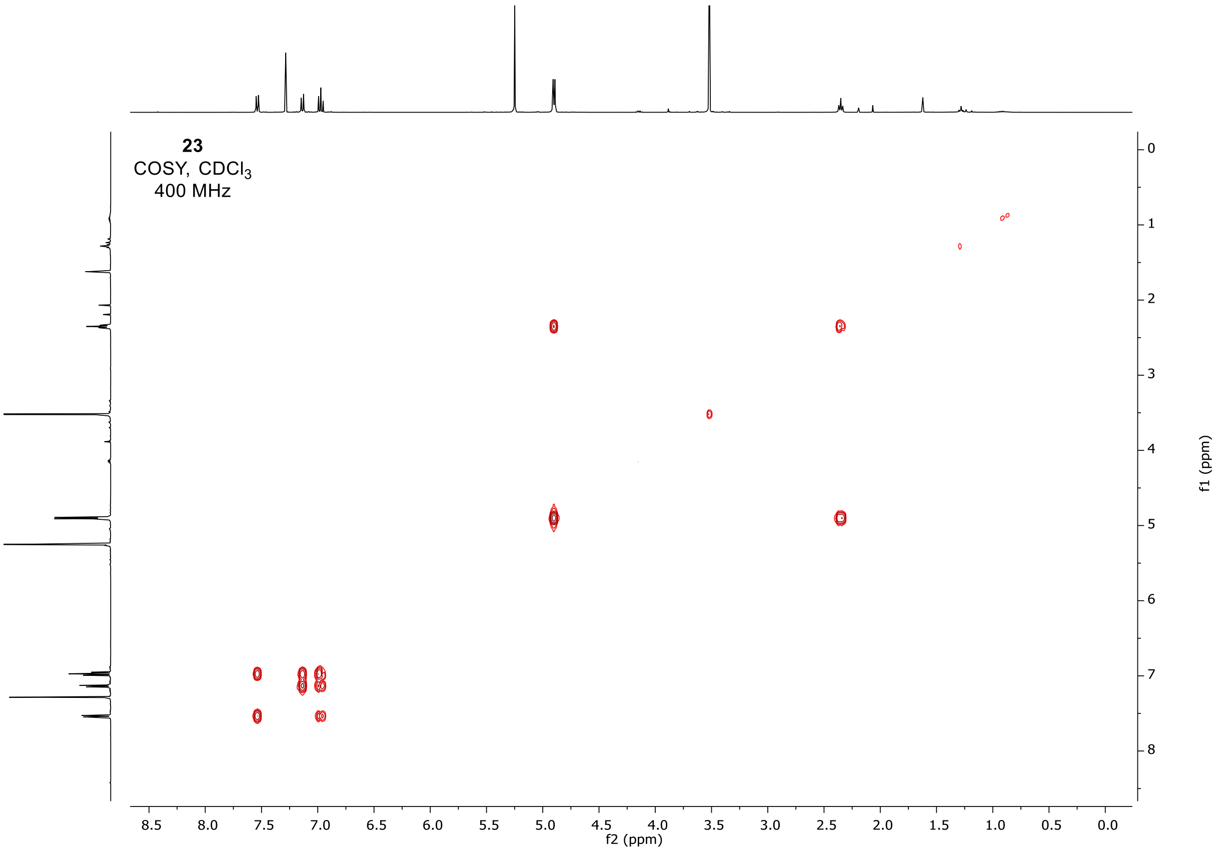
23
COSY, CDCl3
400 MHz
f2 (ppm)
f1 (ppm)

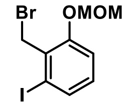

24

^{1}H NMR, $CDCl_3$

400 MHz

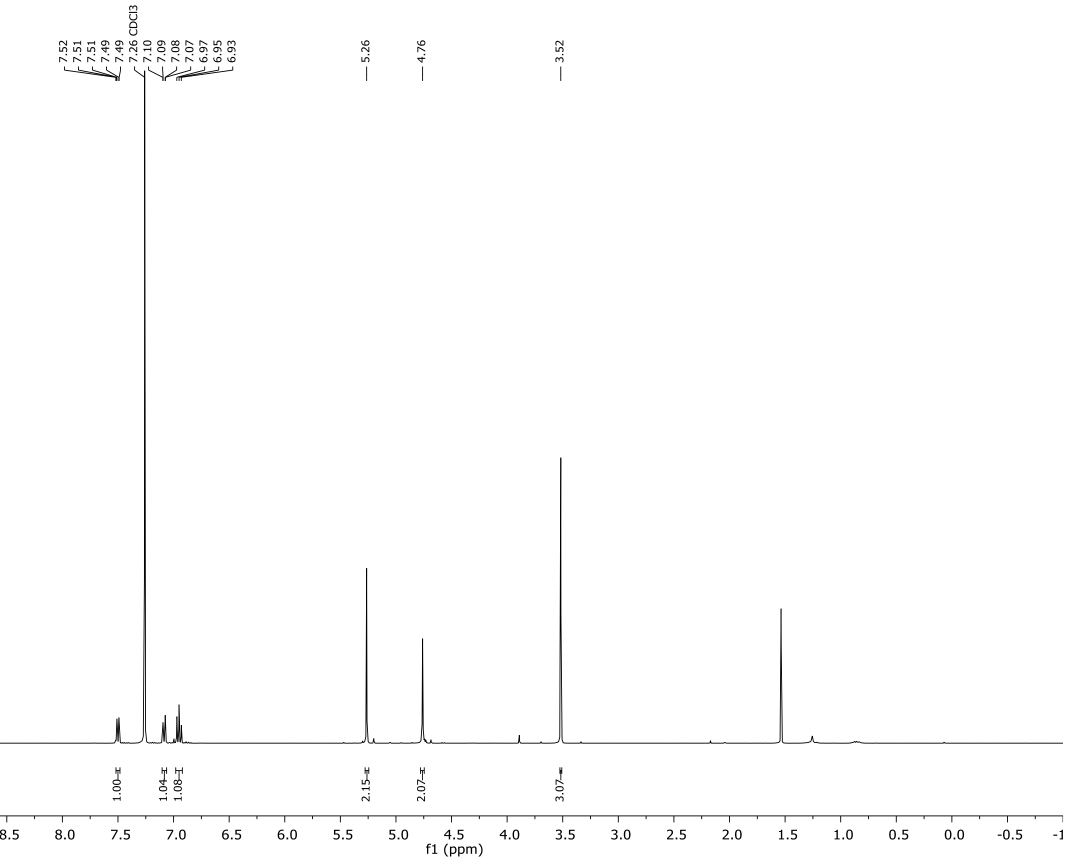

24

^{13}C NMR, $CDCl_3$

101 MHz

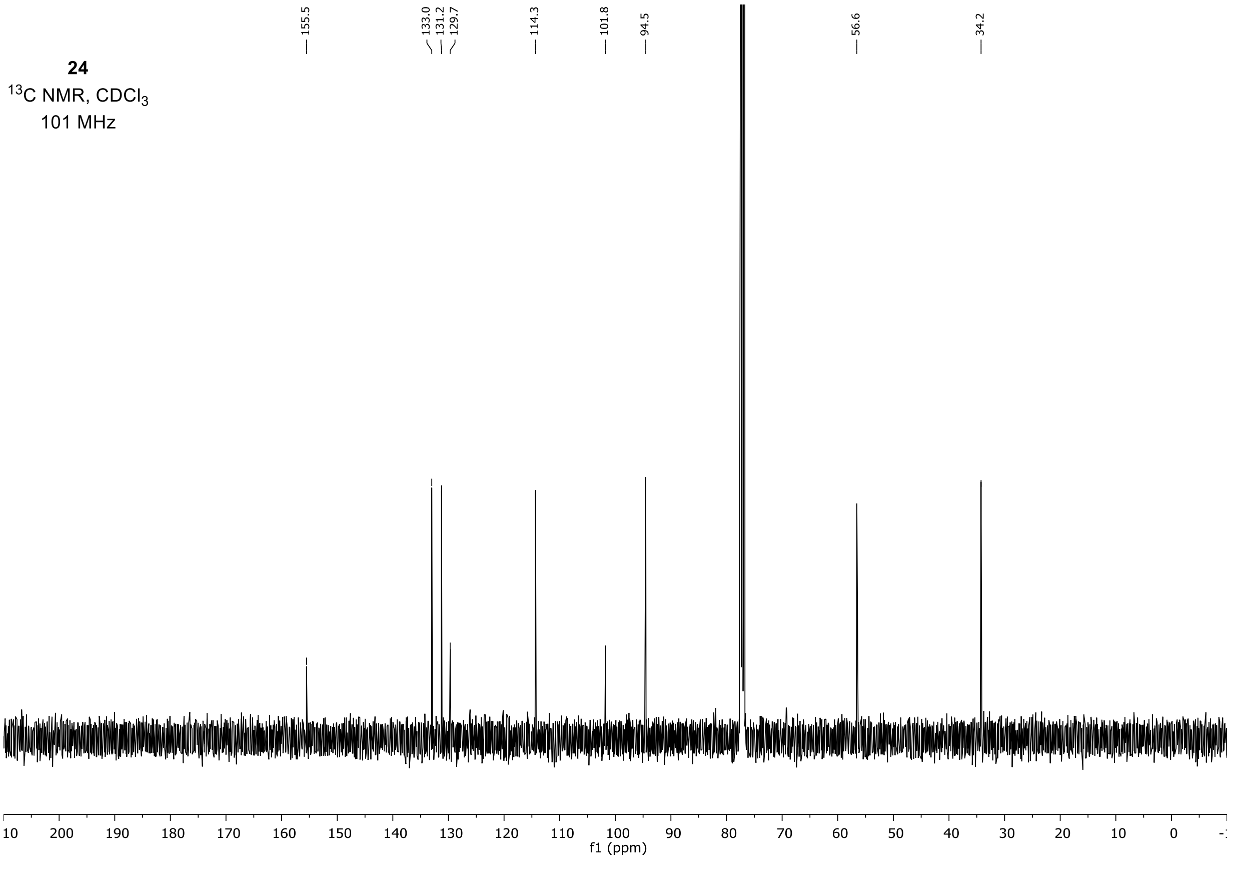

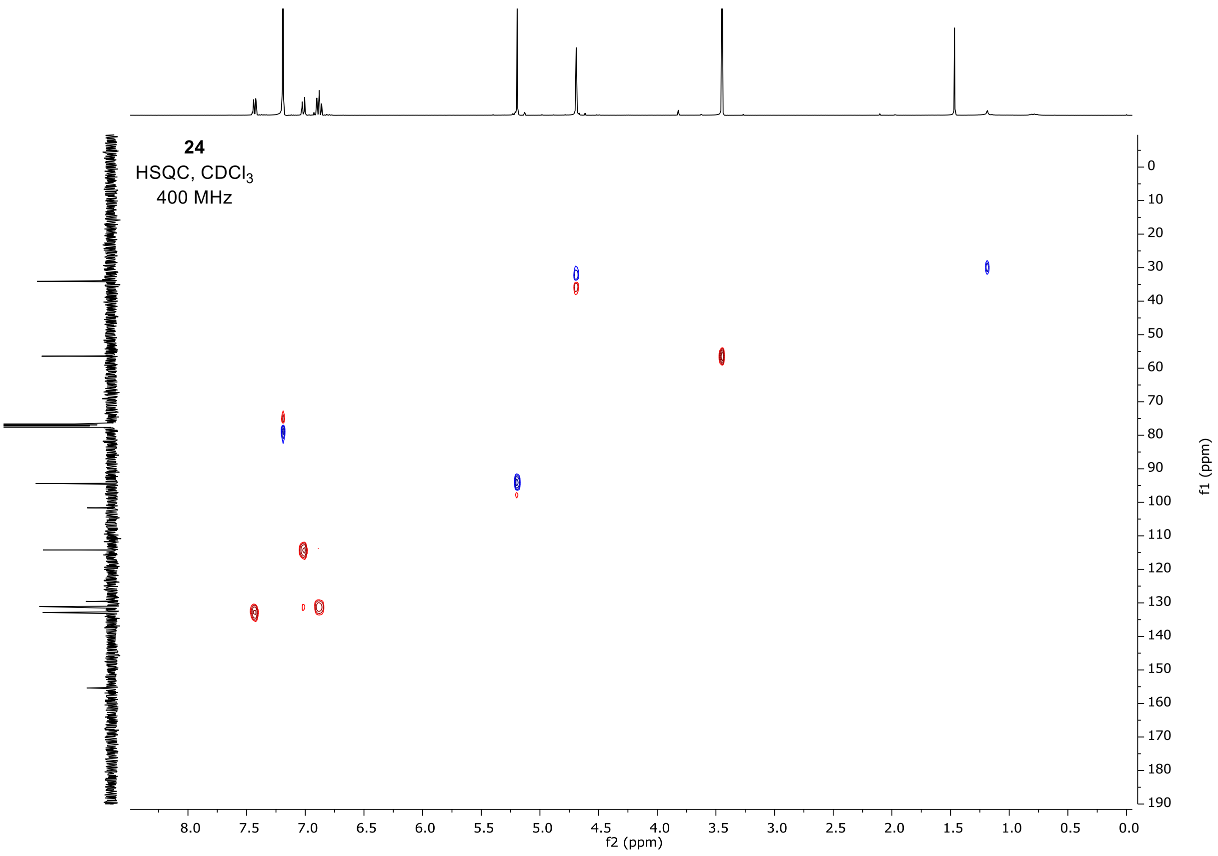
24
HSQC, CDCl3
400 MHz
f2 (ppm)
f1 (ppm)

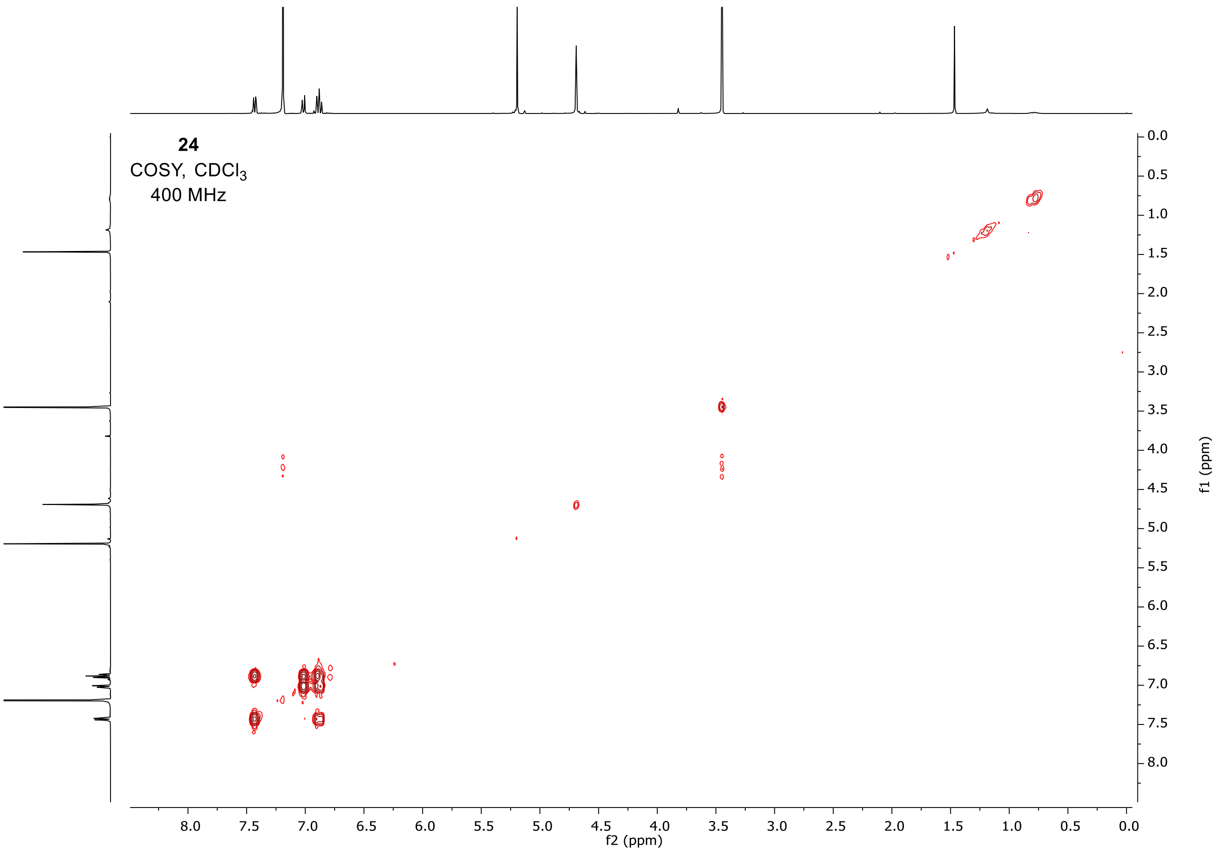
24
COSY, CDCl3
400 MHz
f2 (ppm)
f1 (ppm)

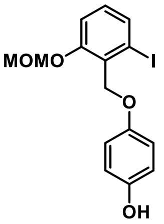

25
^{1}H NMR, $CDCl_3$
400 MHz

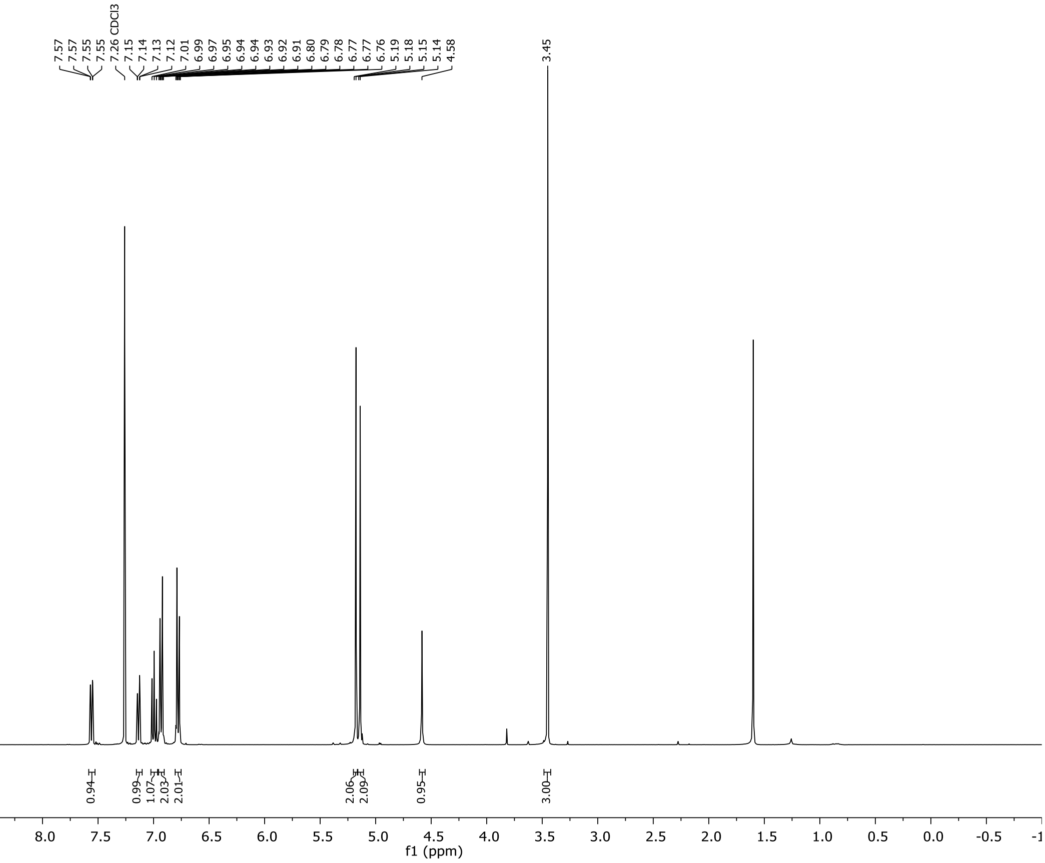

25
^{13}C NMR, $CDCl_3$
101 MHz

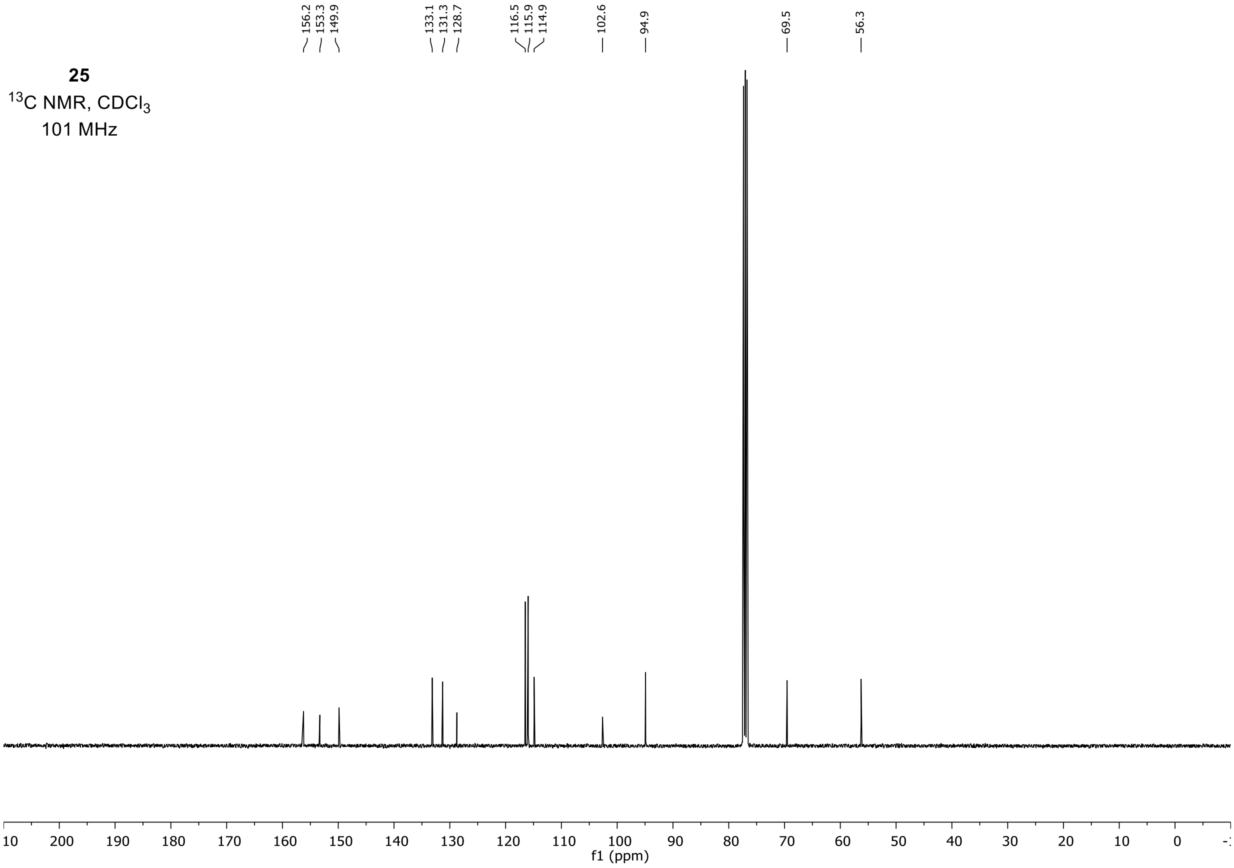

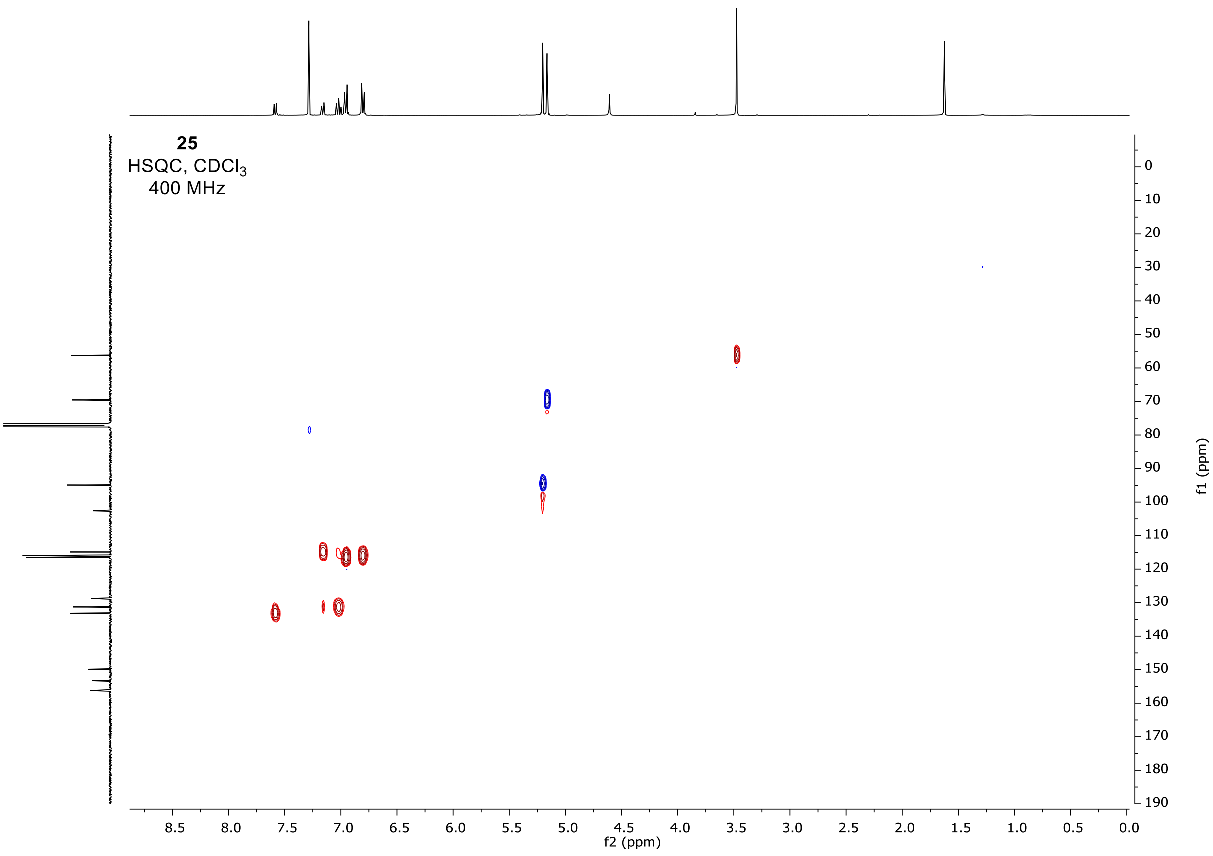
25
HSQC, CDCl3
400 MHz
f2 (ppm)
f1 (ppm)

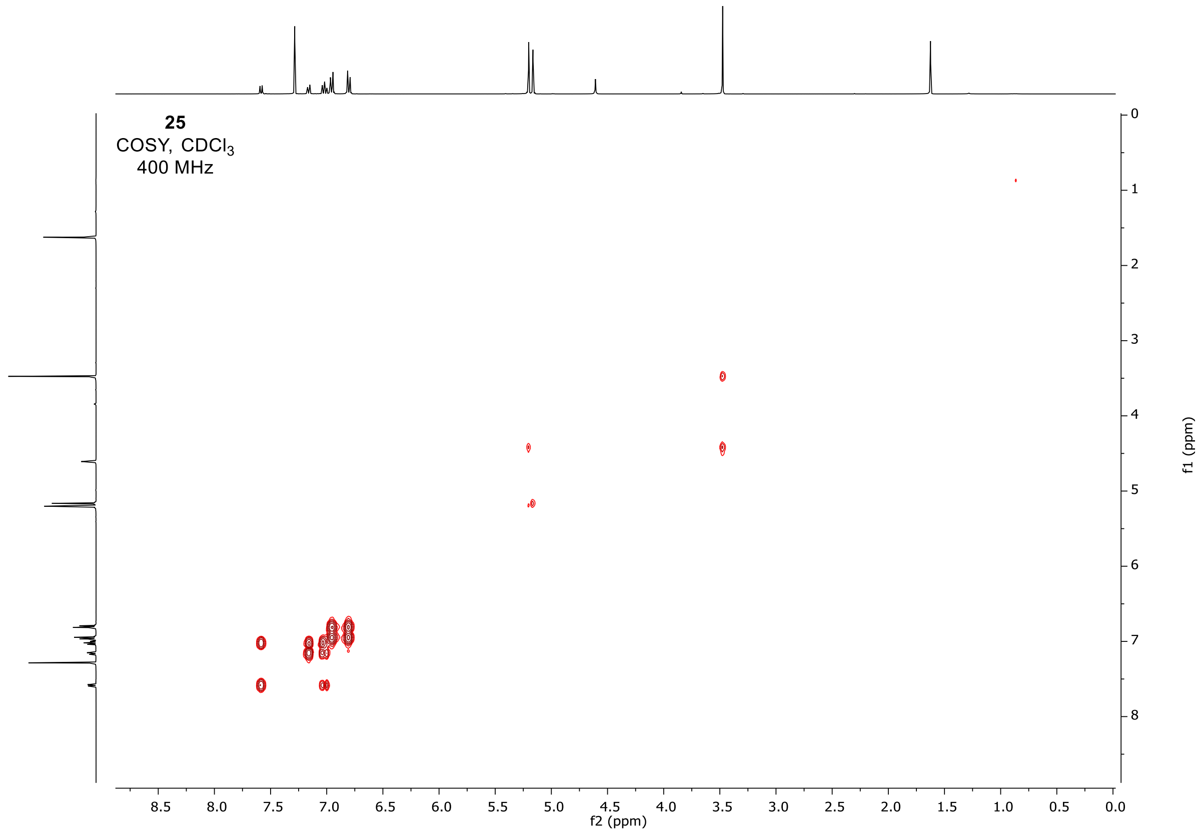
25
COSY, CDCl3
400 MHz
f2 (ppm)
f1 (ppm)

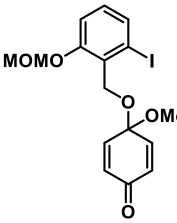

14
^{1}H NMR, $CDCl_3$
400 MHz

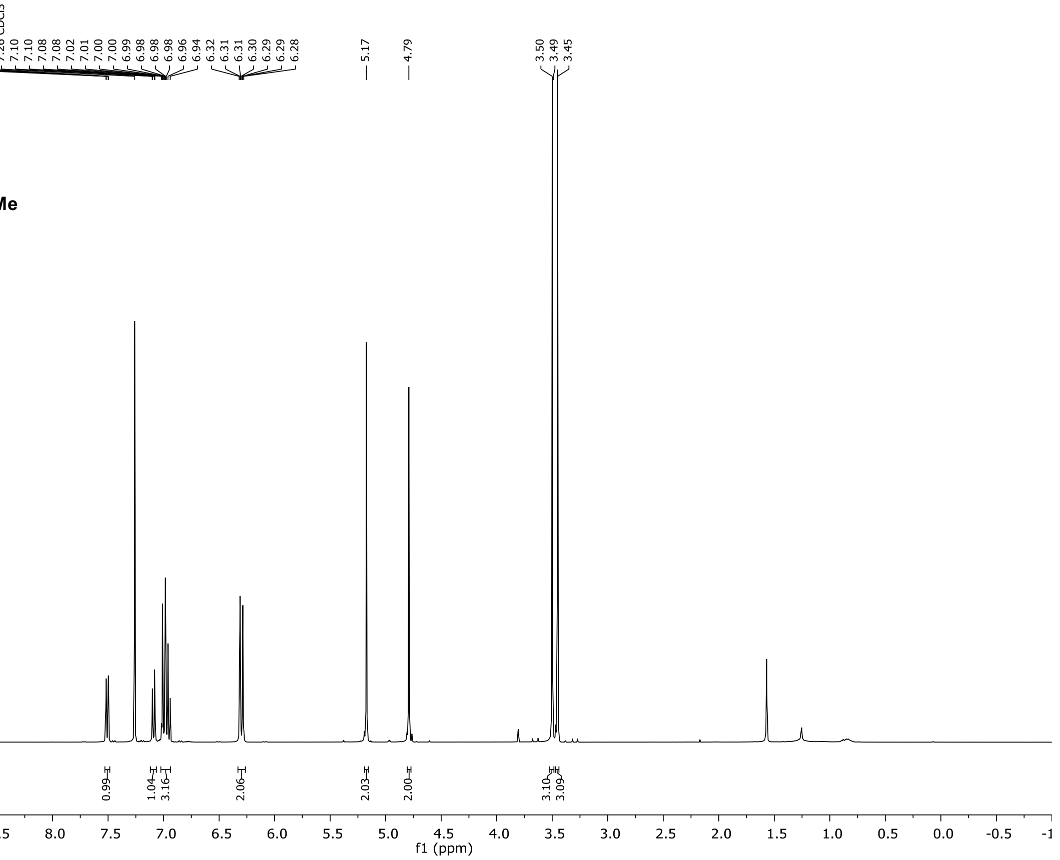

14
^{13}C NMR, $CDCl_3$
101 MHz

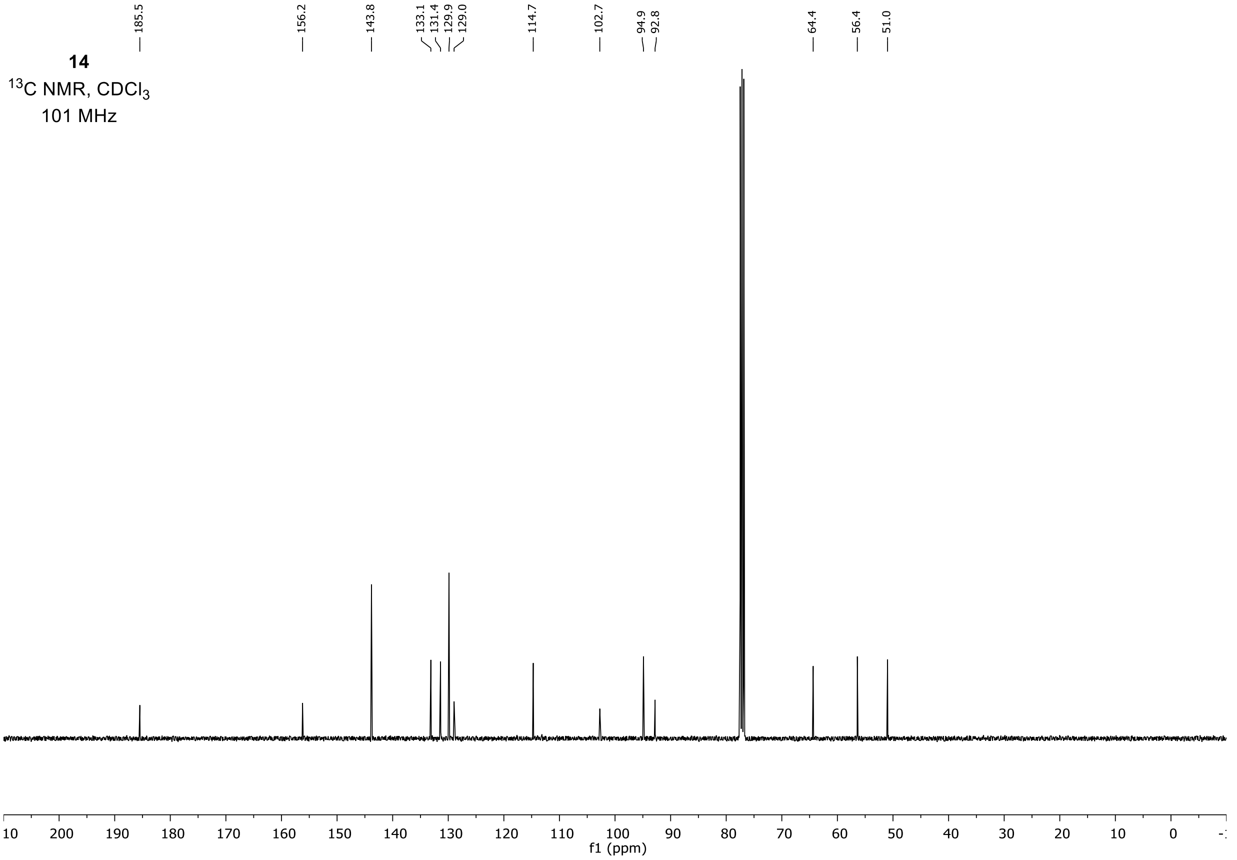

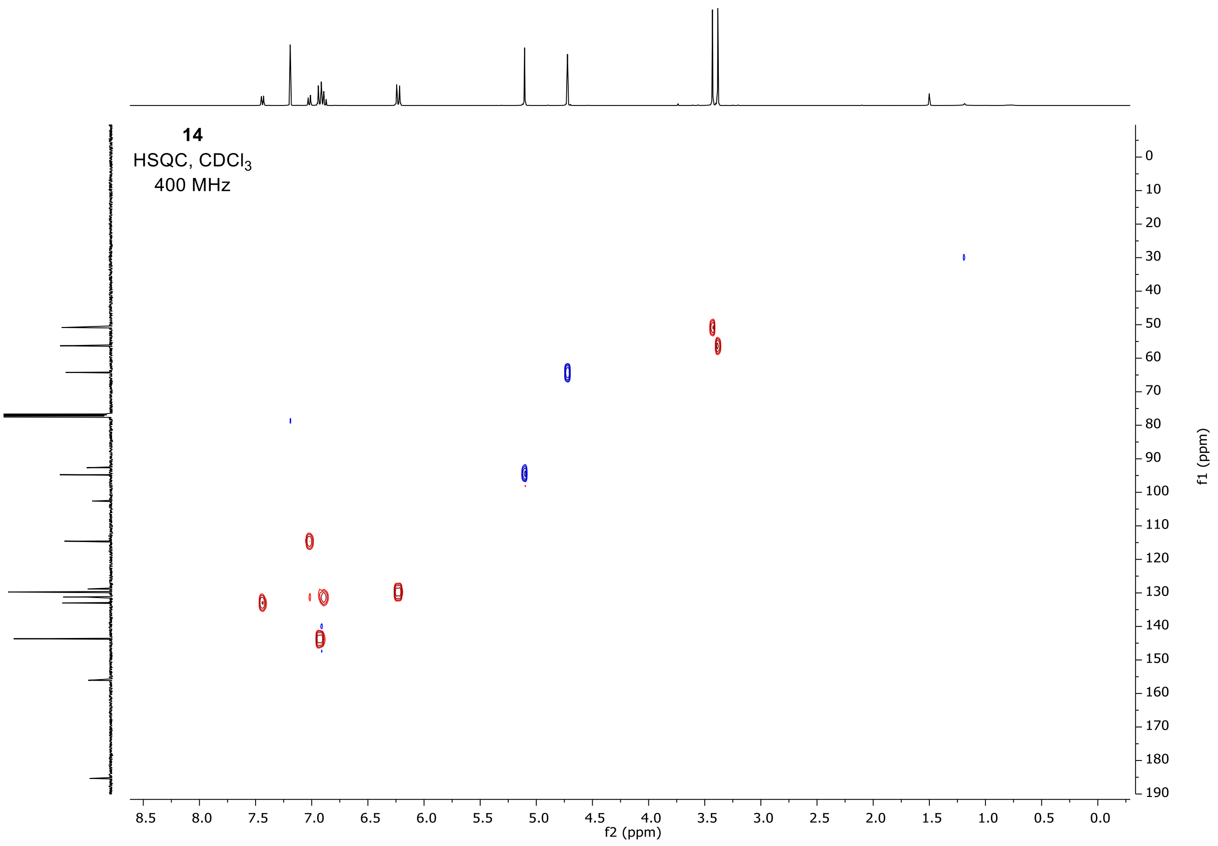
14
HSQC, $CDCl_3$
400 MHz
f2 (ppm)
f1 (ppm)

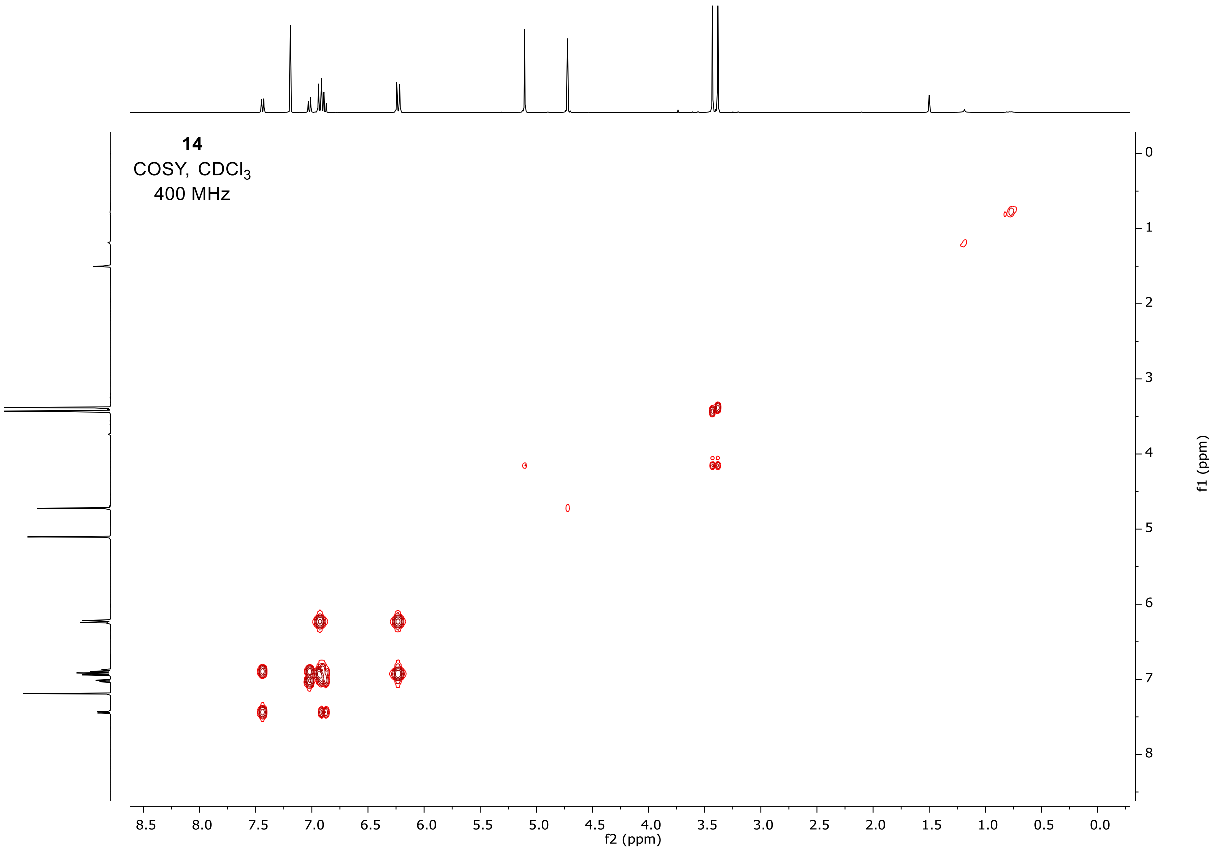
14
COSY, $CDCl_3$
400 MHz
f2 (ppm)
f1 (ppm)

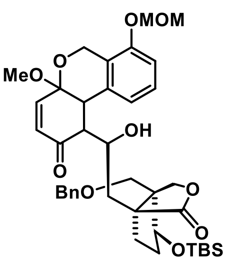

13

^{1}H NMR, $CDCl_3$

400 MHz

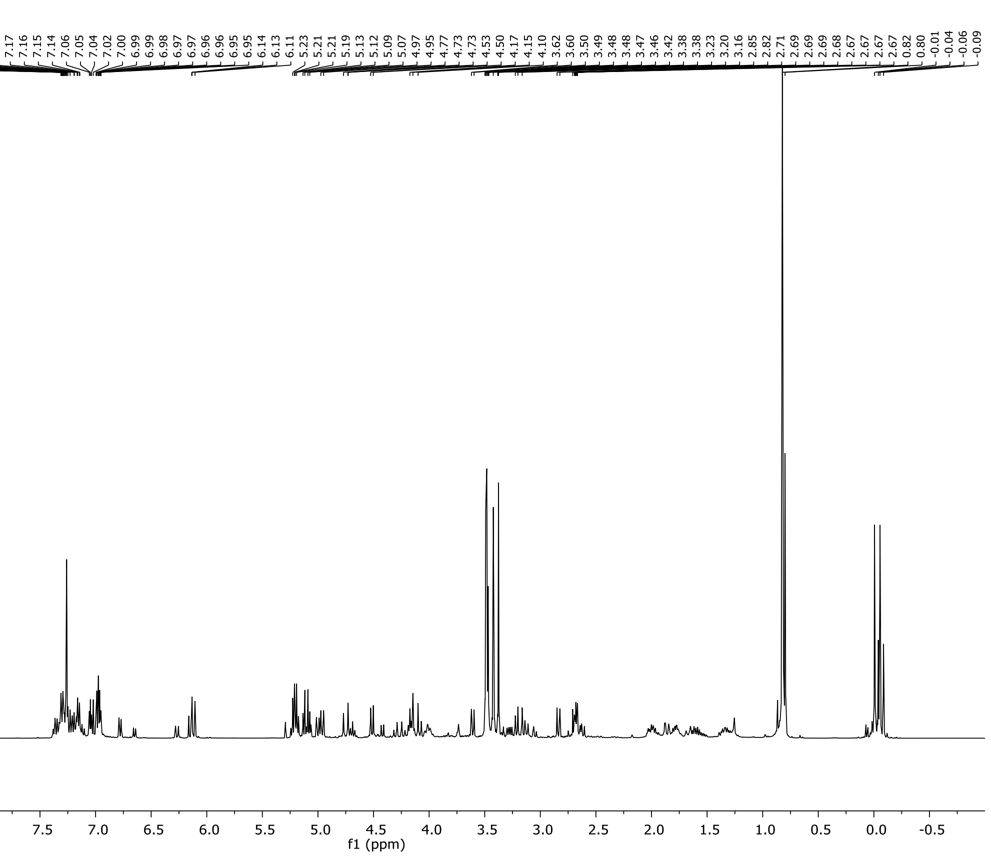

13

^{13}C NMR, $CDCl_3$

101 MHz

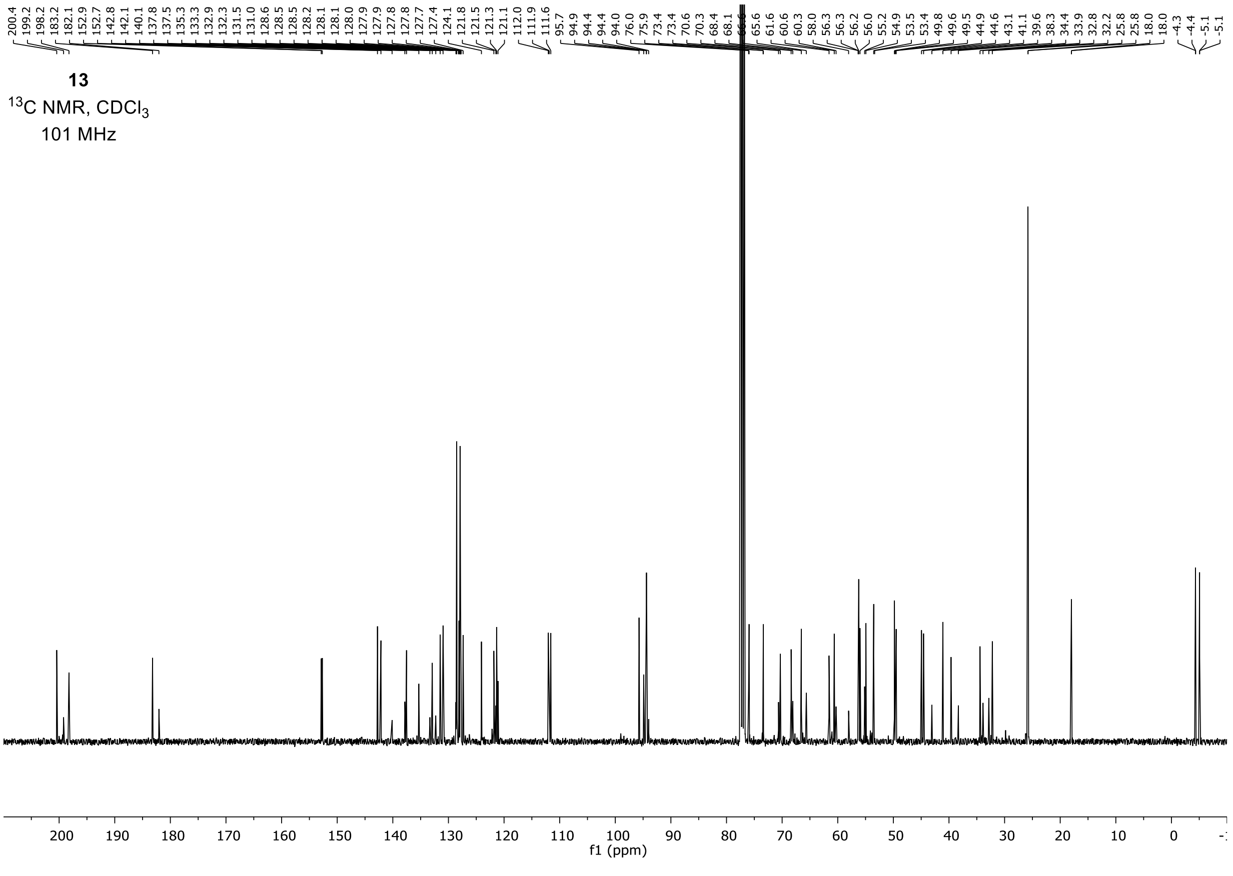

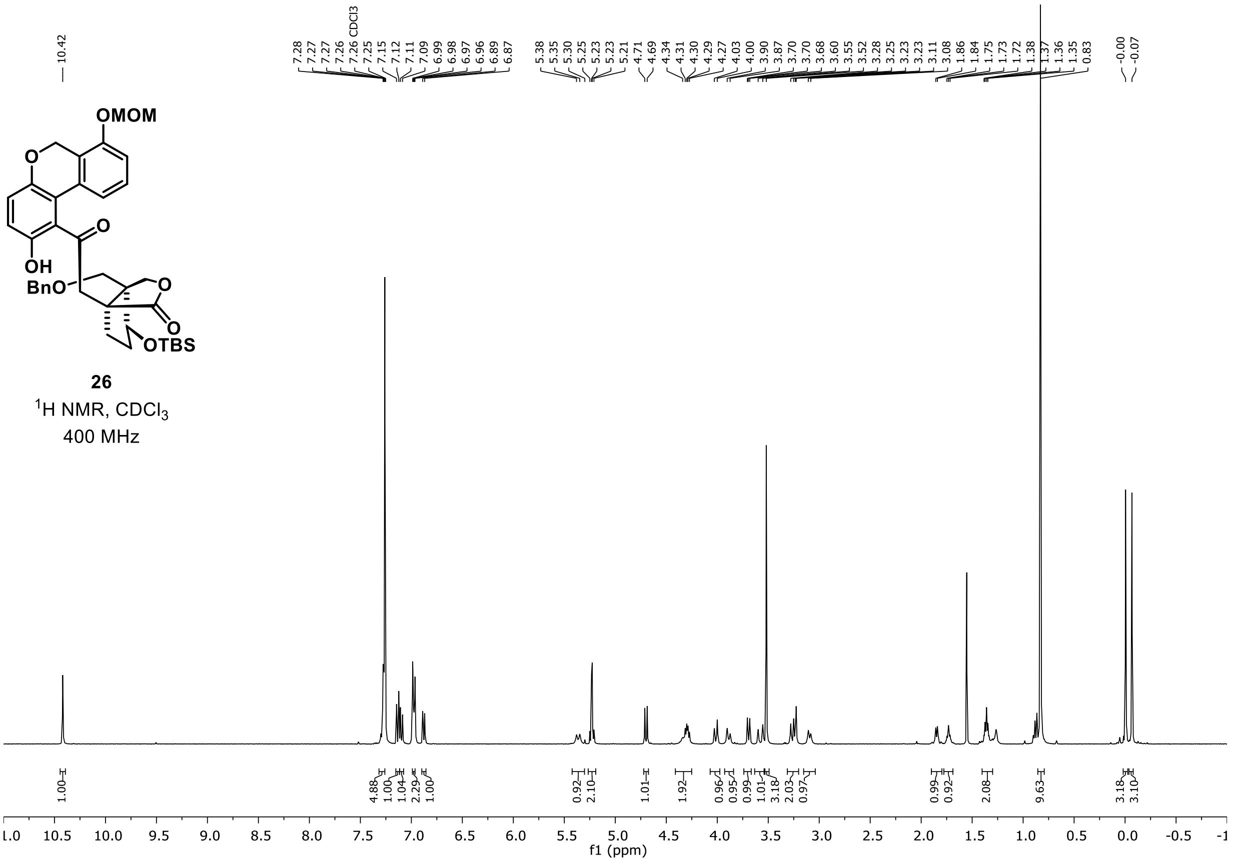

OMOM
O
O
OH
BnO
O
O
OTBS
26
^{1}H NMR, CDCl$_3$
400 MHz
10.42
7.28 7.27 7.27 7.26 7.26 CDCl3 7.25 7.15 7.12 7.11 7.09 6.99 6.98 6.97 6.96 6.89 6.87
5.38 5.35 5.30 5.25 5.23 5.23 5.21 4.71 4.69 4.34 4.31 4.30 4.29 4.27 4.03 4.00 3.90 3.87 3.70 3.70 3.68 3.60 3.55 3.52 3.28 3.25 3.23 3.23 3.11 3.08 1.86 1.84 1.75 1.73 1.72 1.38 1.37 1.36 1.35 0.83
-0.00 -0.07
1.00 4.88 1.00 1.04 2.29 1.00 0.92 2.10 1.01 1.92 0.96 0.95 0.99 1.01 3.18 2.03 0.97 0.99 0.92 2.08 9.63 3.18 3.10
f1 (ppm)

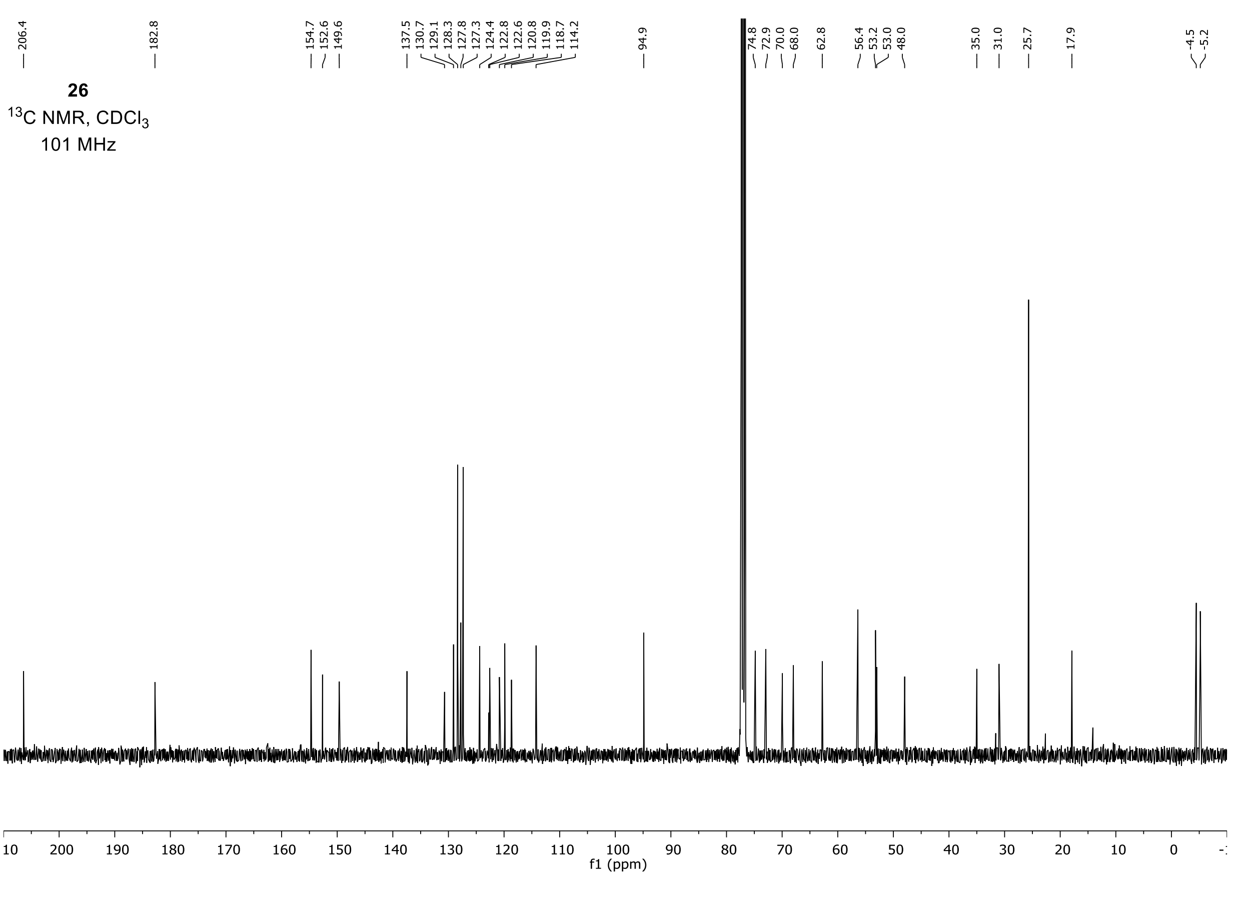

26
^{13}C NMR, CDCl$_3$
101 MHz
206.4 182.8 154.7 152.6 149.6 137.5 130.7 129.1 128.3 127.8 127.3 124.4 122.8 122.6 120.8 119.9 118.7 114.2 94.9 74.8 72.9 70.0 68.0 62.8 56.4 53.2 53.0 48.0 35.0 31.0 25.7 17.9 -4.5 -5.2
f1 (ppm)

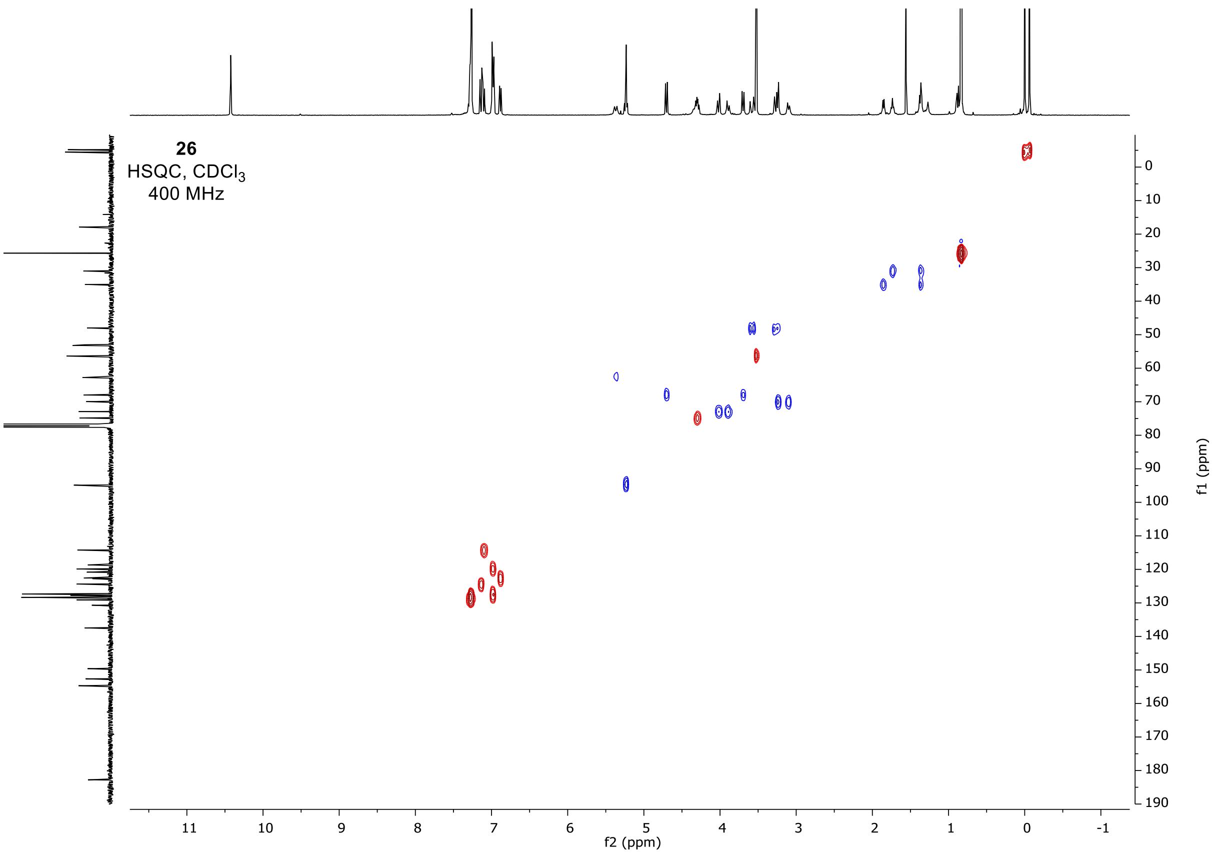
26
HSQC, CDCl3
400 MHz
f2 (ppm)
f1 (ppm)

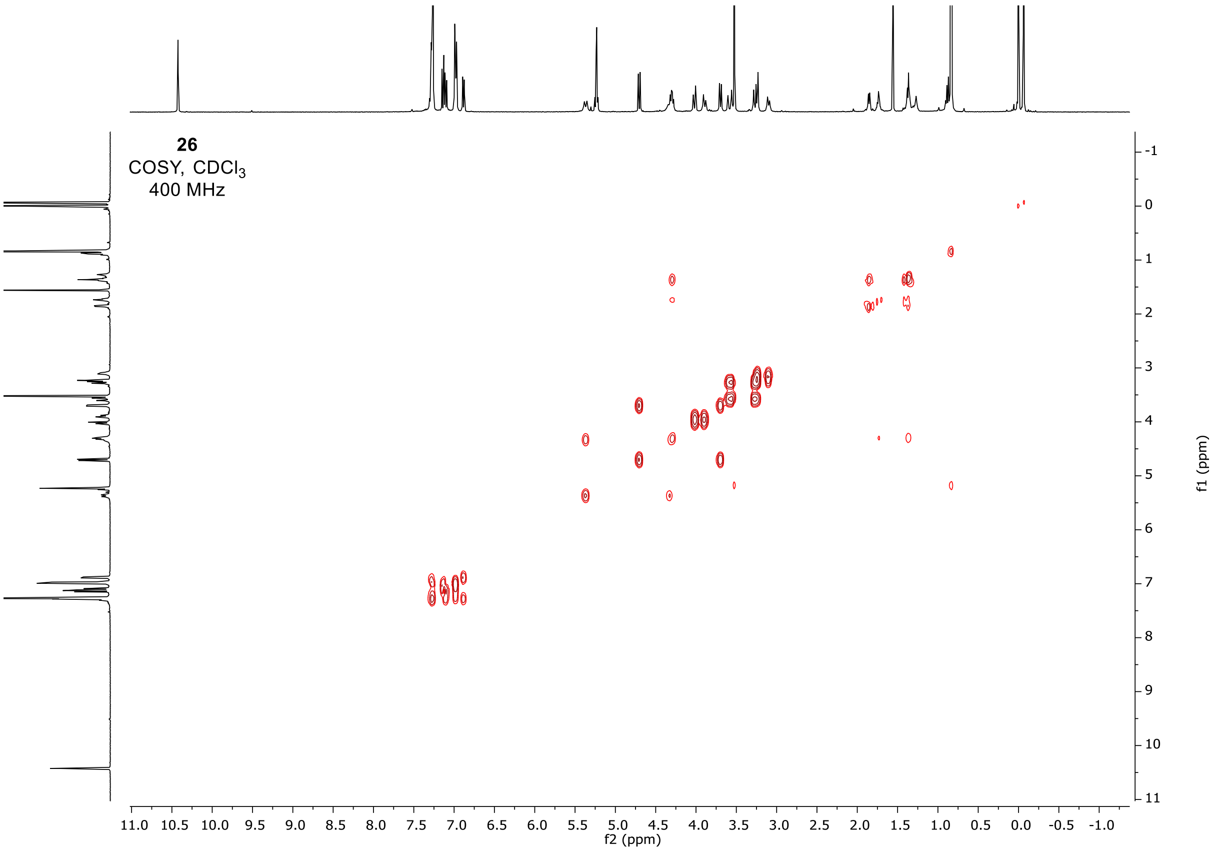
26
COSY, CDCl3
400 MHz
f2 (ppm)
f1 (ppm)

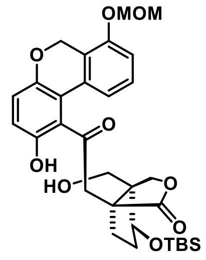

27
^{1}H NMR, $CDCl_3$
400 MHz

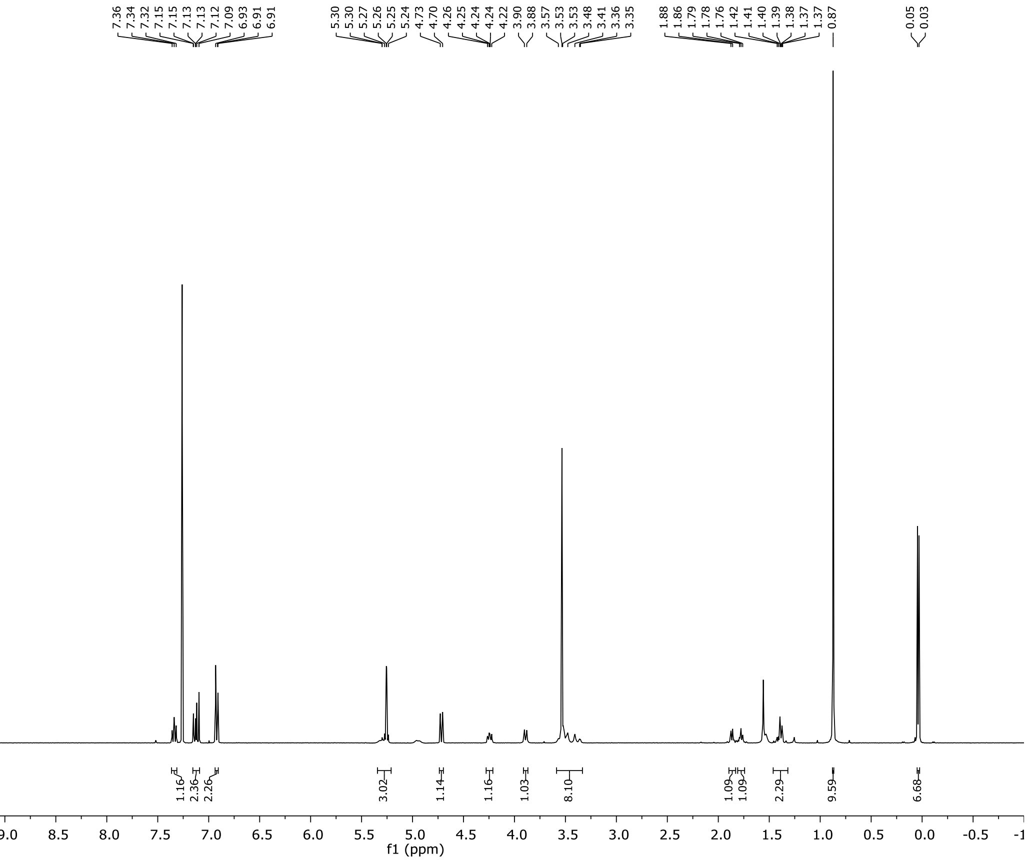

27
^{13}C NMR, $CDCl_3$
101 MHz

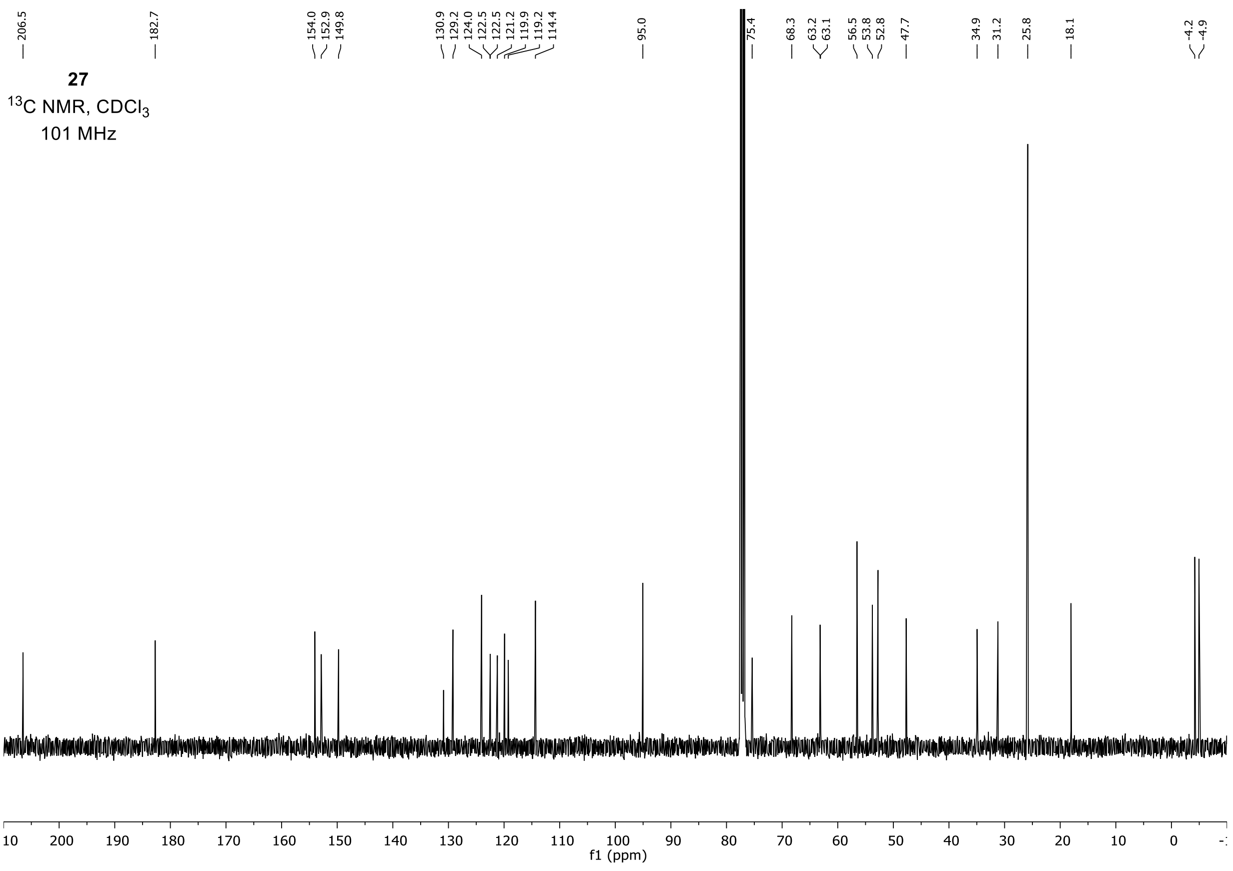

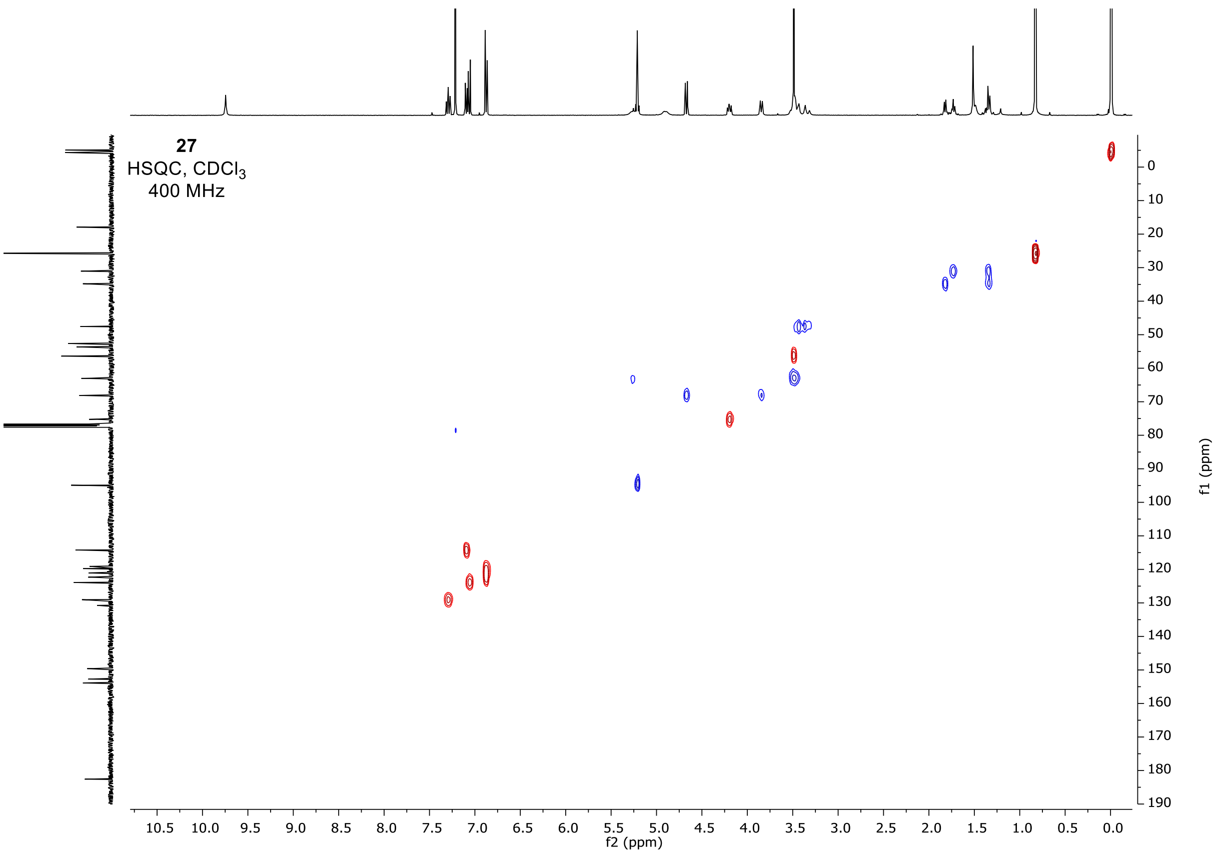
27
HSQC, CDCl3
400 MHz
f2 (ppm)
f1 (ppm)

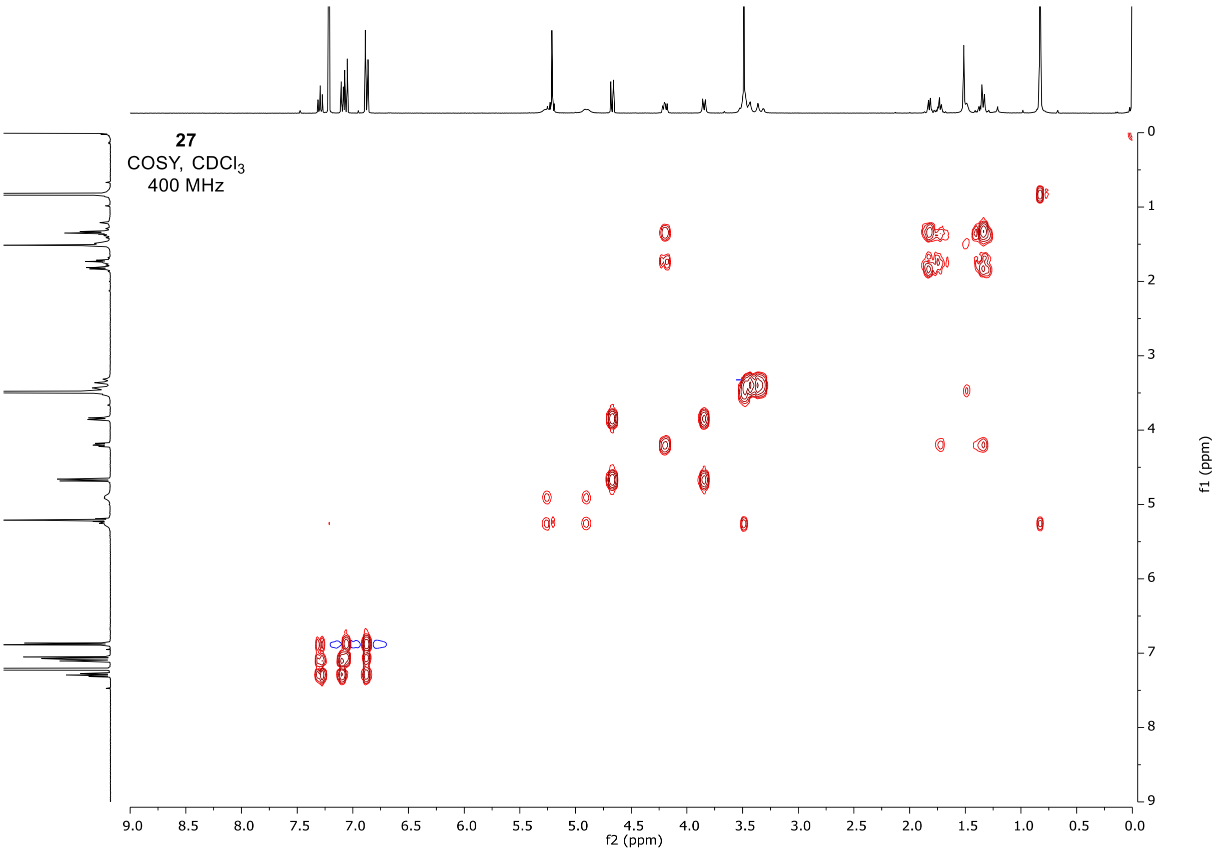
27
COSY, CDCl3
400 MHz
f2 (ppm)
f1 (ppm)

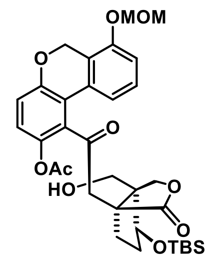

S10

^{1}H NMR, $CDCl_3$

400 MHz

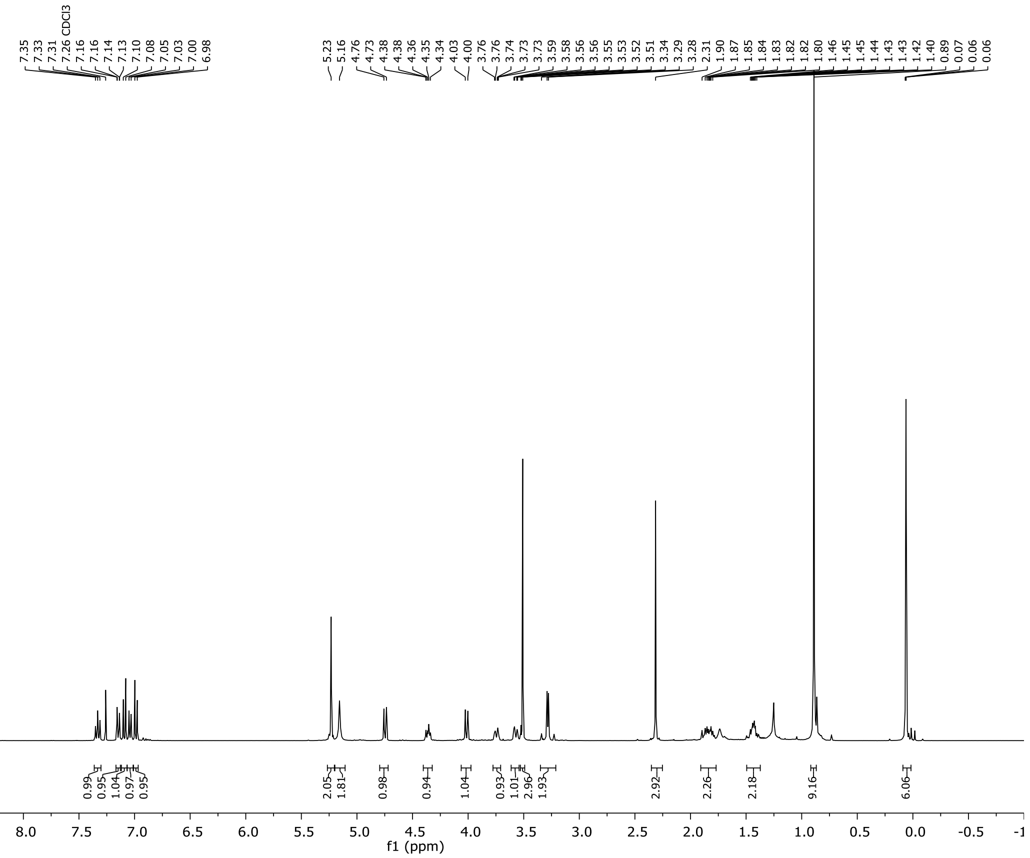

S10

^{13}C NMR, $CDCl_3$

101 MHz

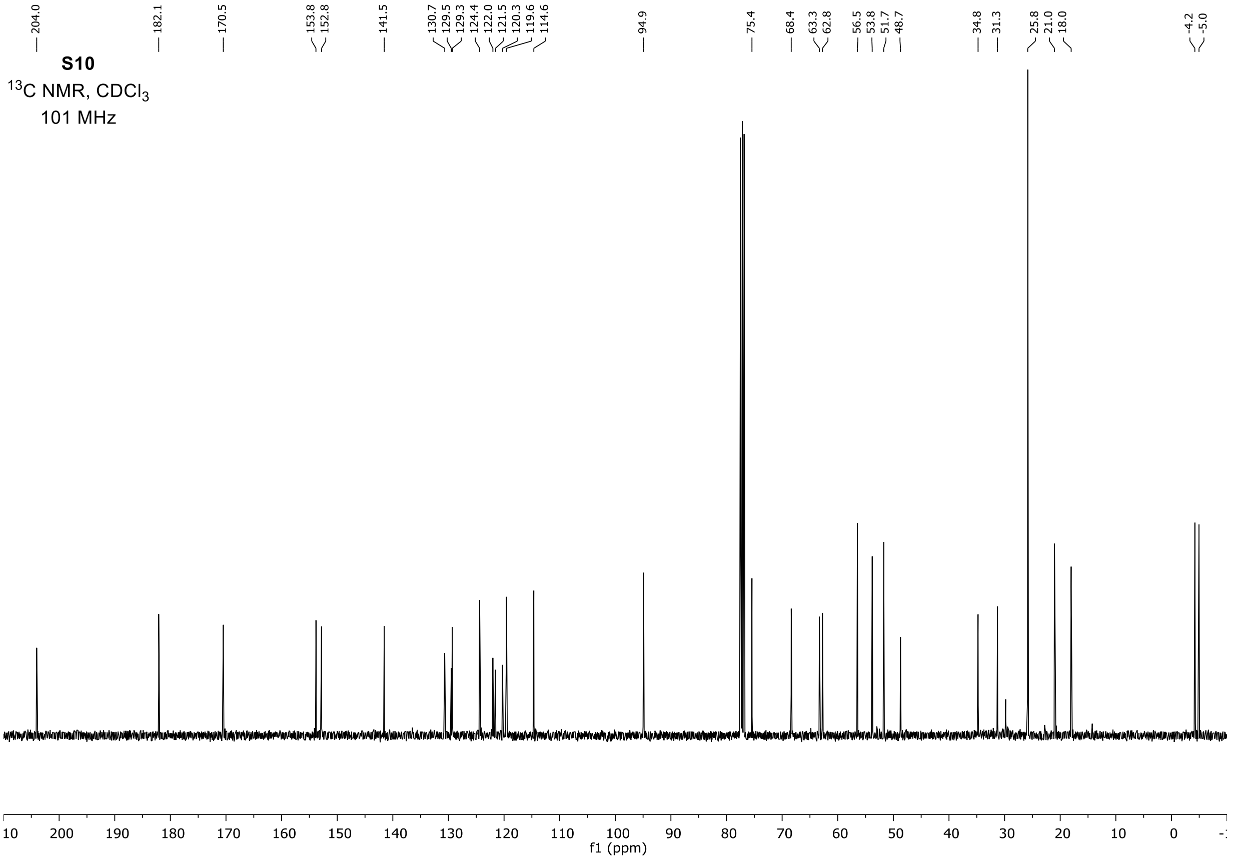

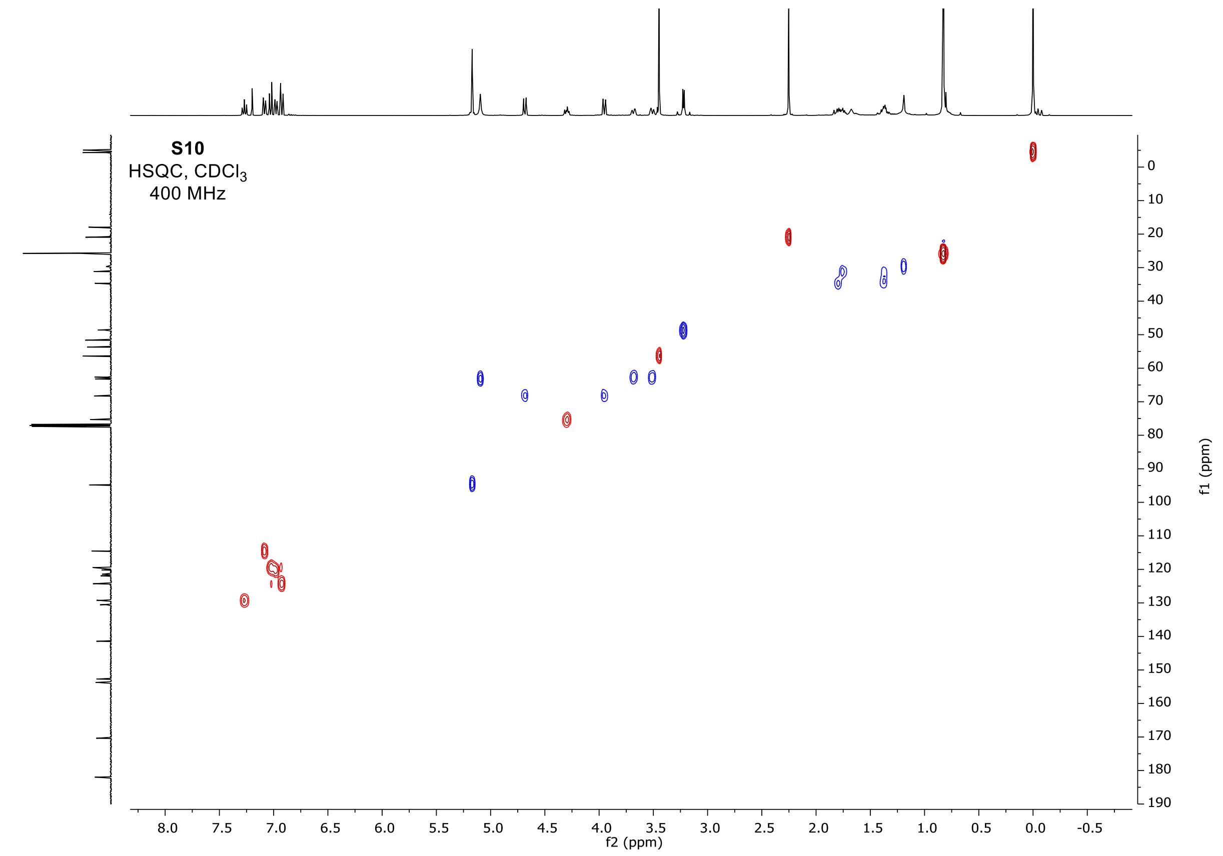
S10
HSQC, CDCl3
400 MHz
f2 (ppm)
f1 (ppm)

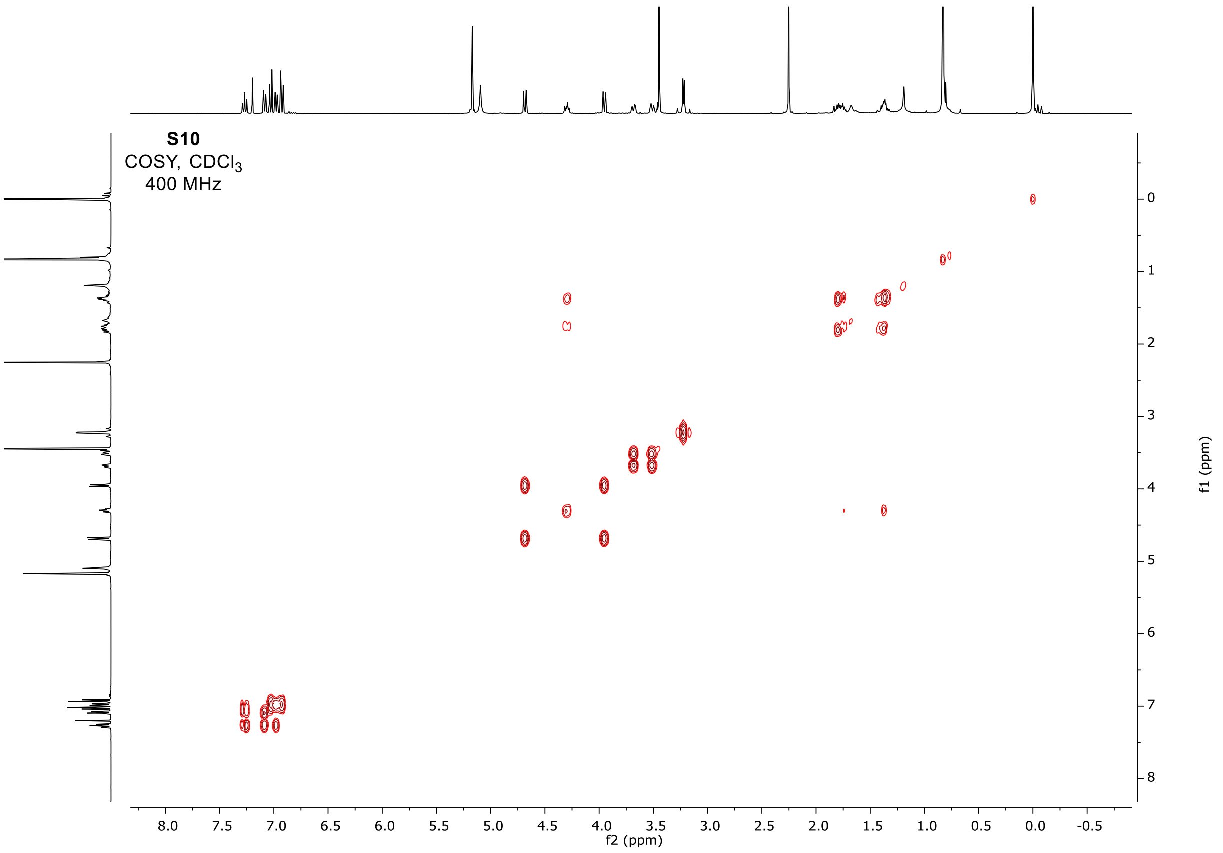
S10
COSY, CDCl3
400 MHz
f2 (ppm)
f1 (ppm)

28

^{1}H NMR, $CDCl_3$

400 Mhz

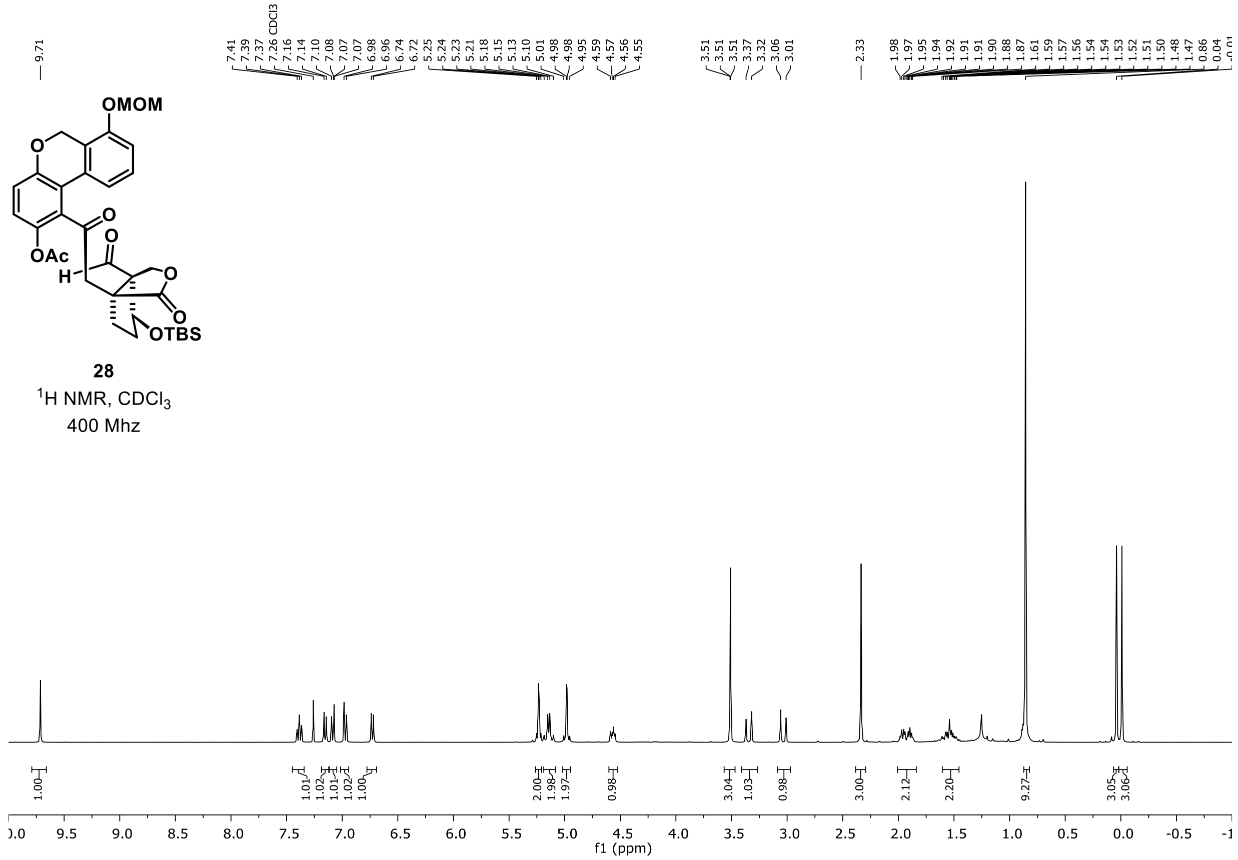

28

^{13}C NMR, $CDCl_3$

101 Mhz

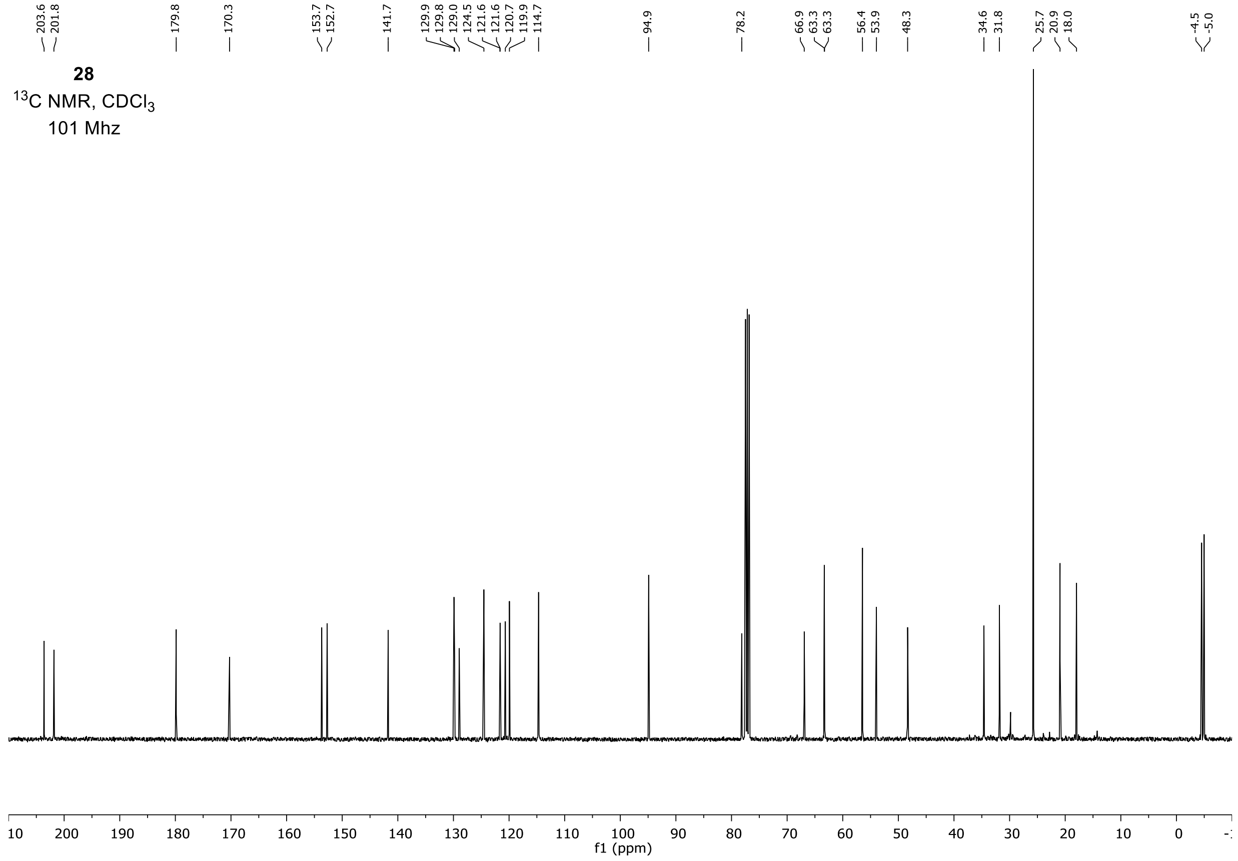

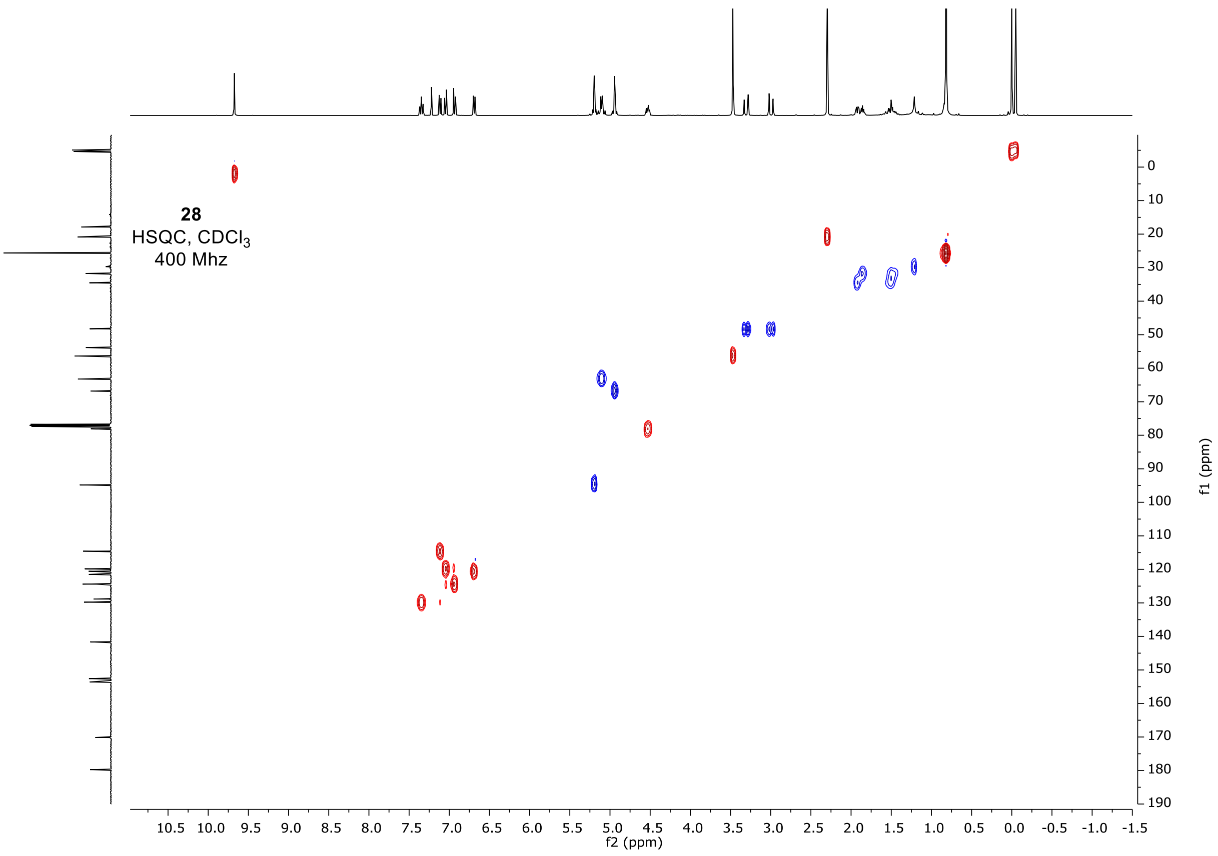
28
HSQC, CDCl3
400 Mhz
f2 (ppm)
f1 (ppm)

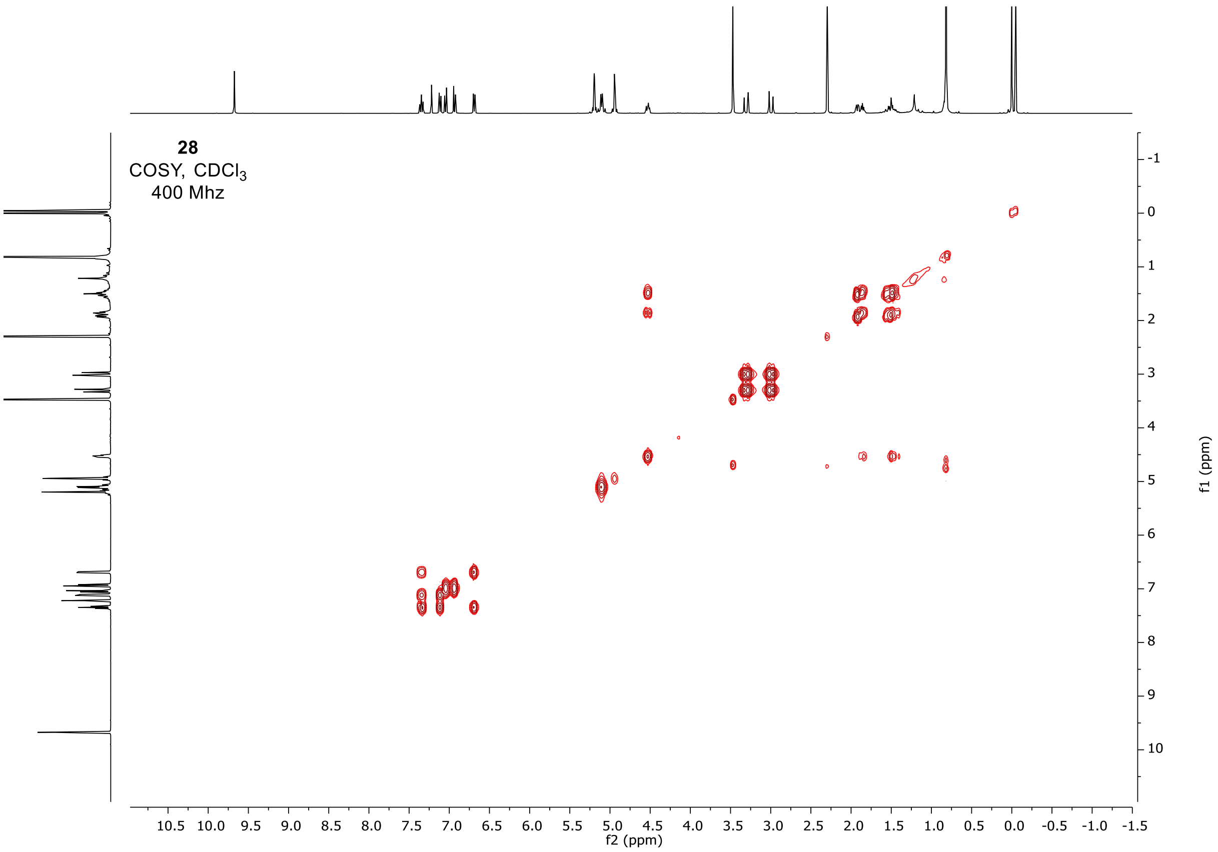
28
COSY, CDCl3
400 Mhz
f2 (ppm)
f1 (ppm)

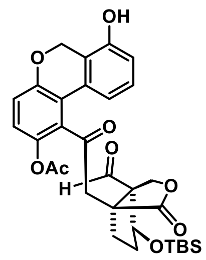

S11
^{1}H NMR, $CDCl_3$
400 MHz

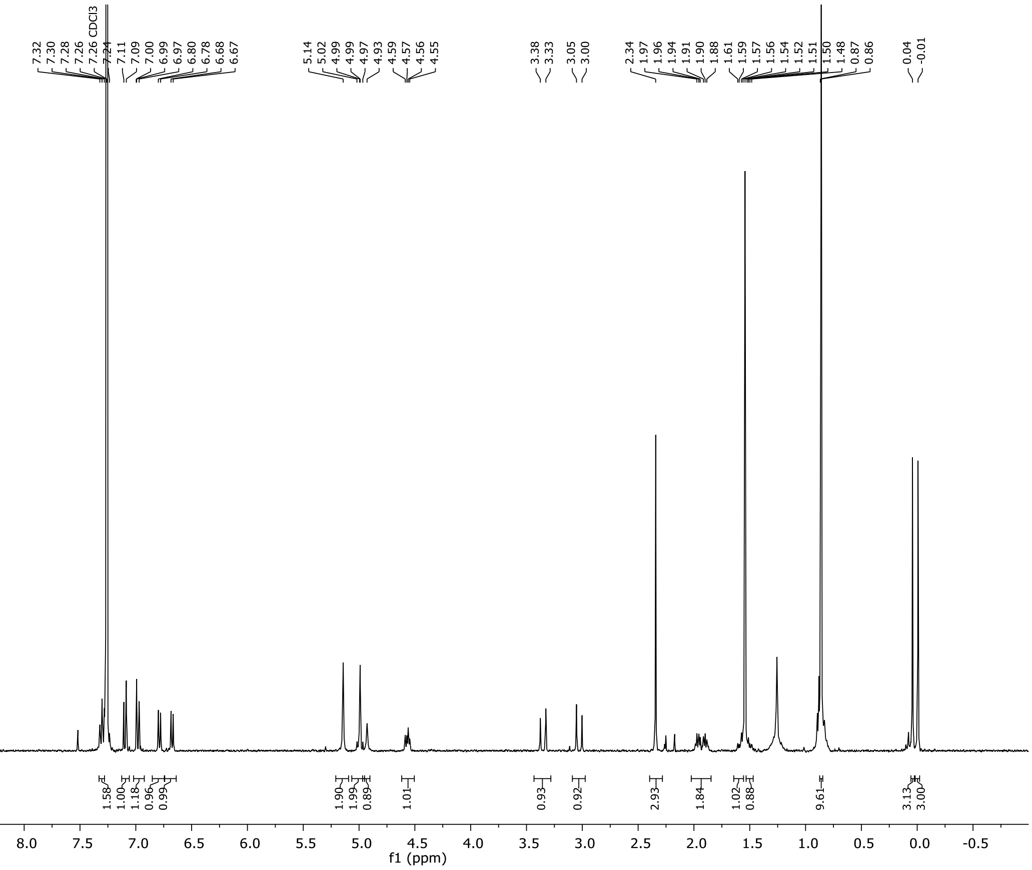

S11
^{13}C NMR, $CDCl_3$
101 MHz

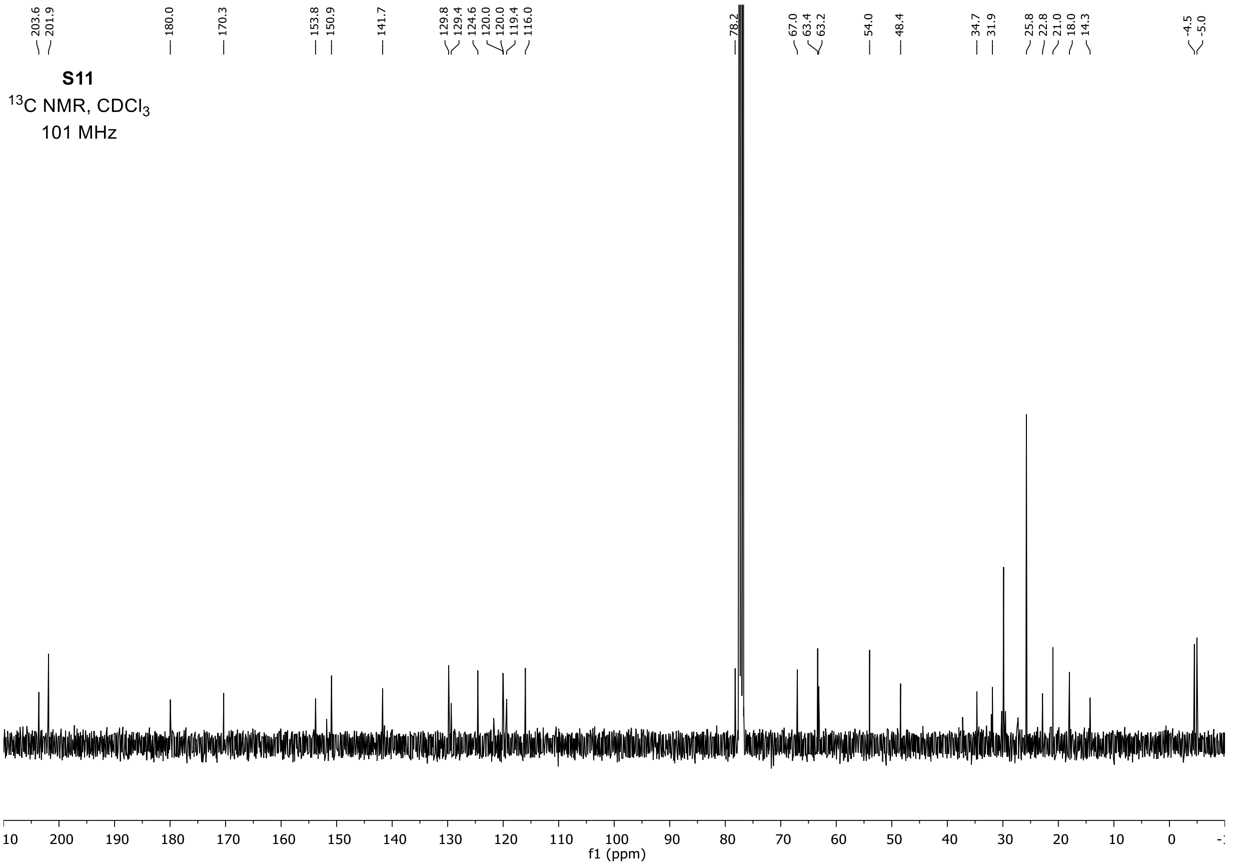

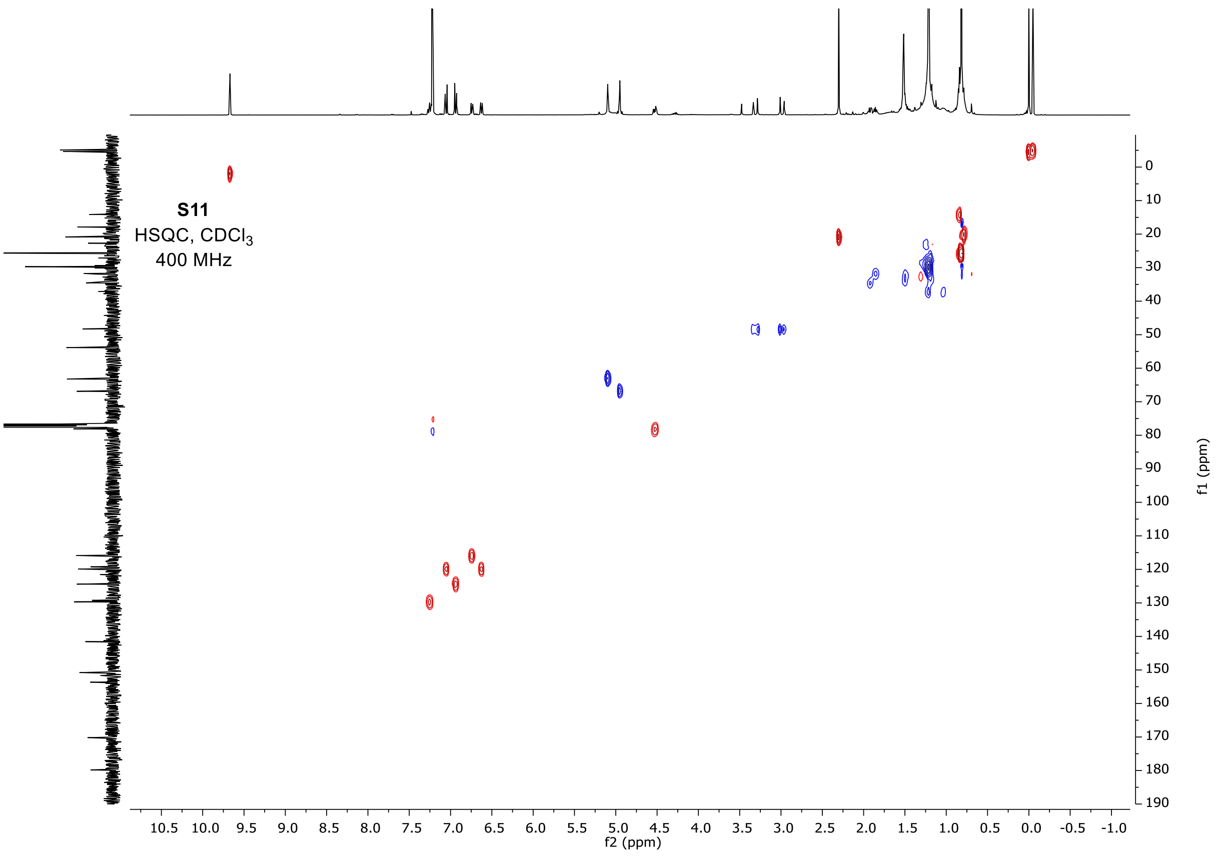
S11
HSQC, CDCl3
400 MHz
f2 (ppm)
f1 (ppm)

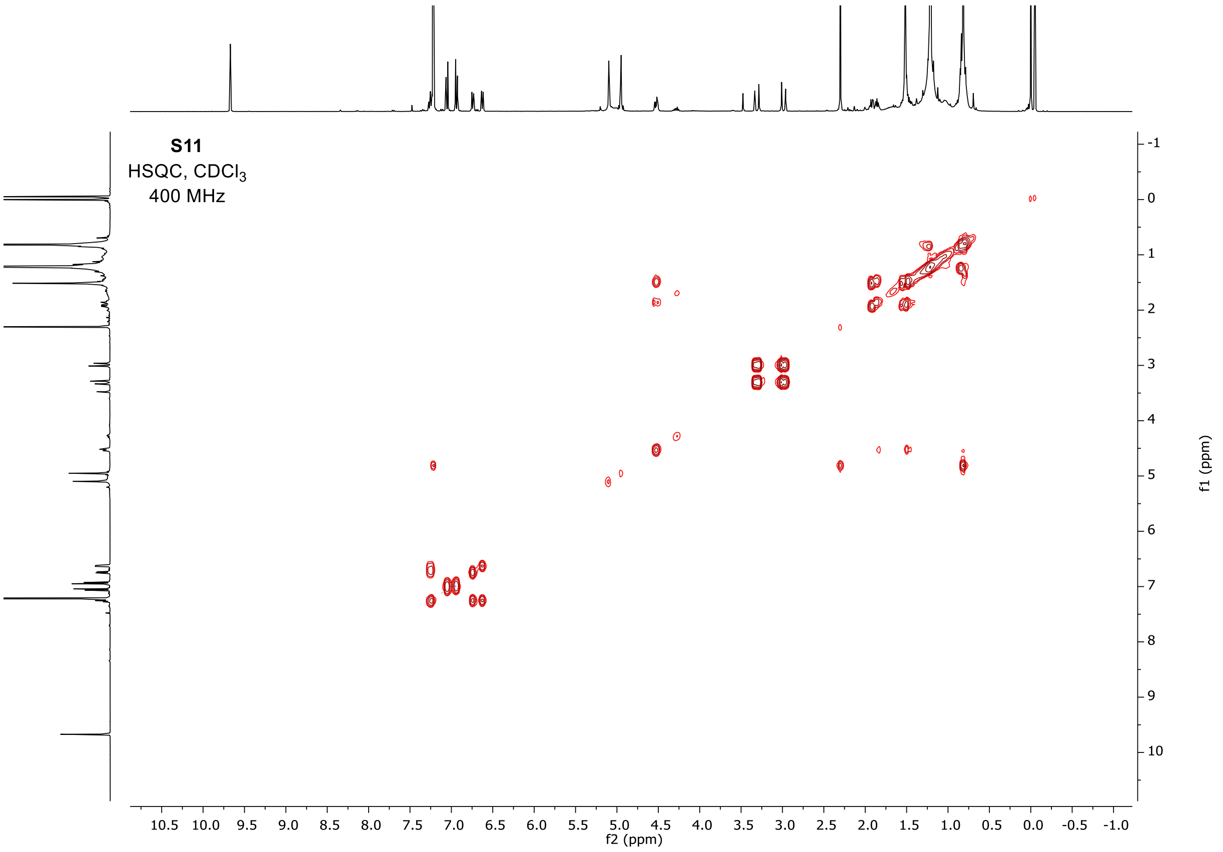
S11
HSQC, CDCl3
400 MHz
f2 (ppm)
f1 (ppm)

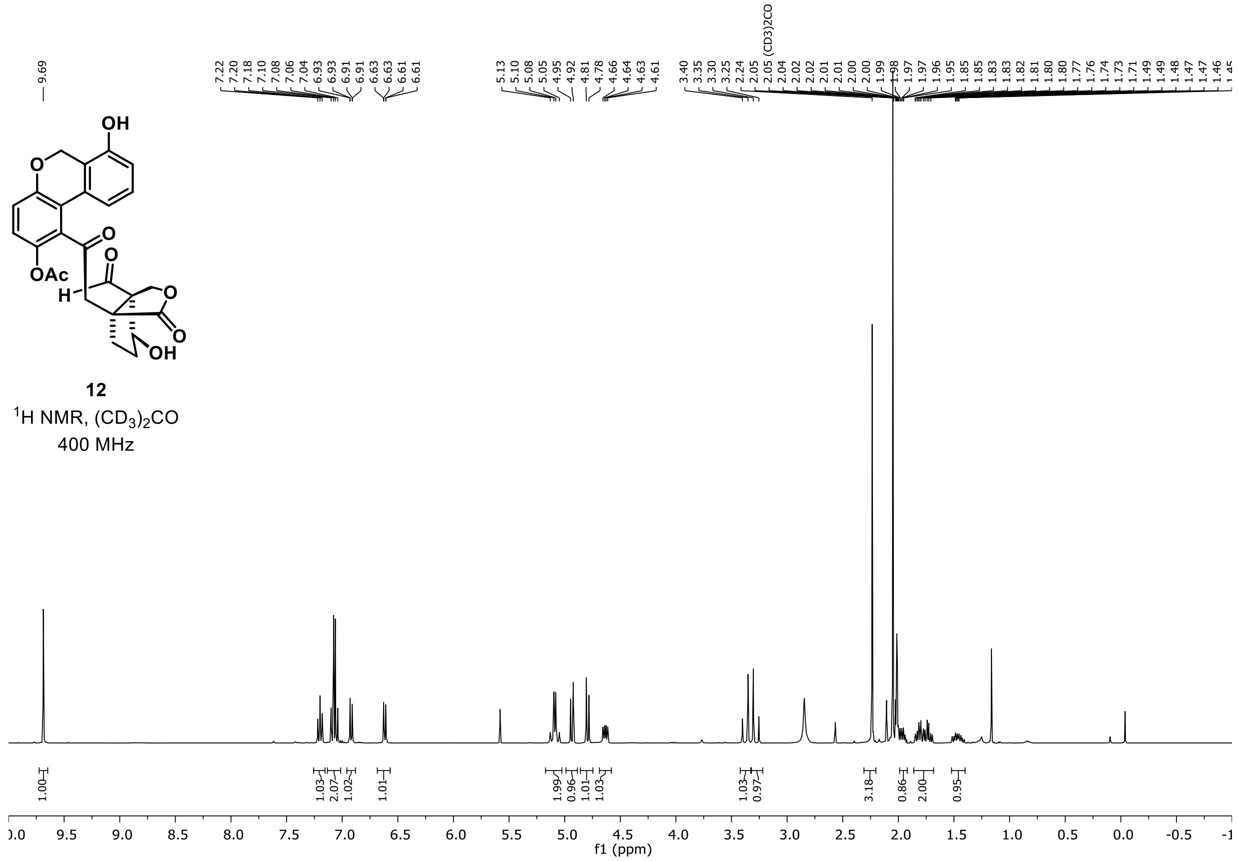
OH
O
OAc
H
O
O
O
O
OH
12
1H NMR, (CD3)2CO
400 MHz
(CD3)2CO
f1 (ppm)

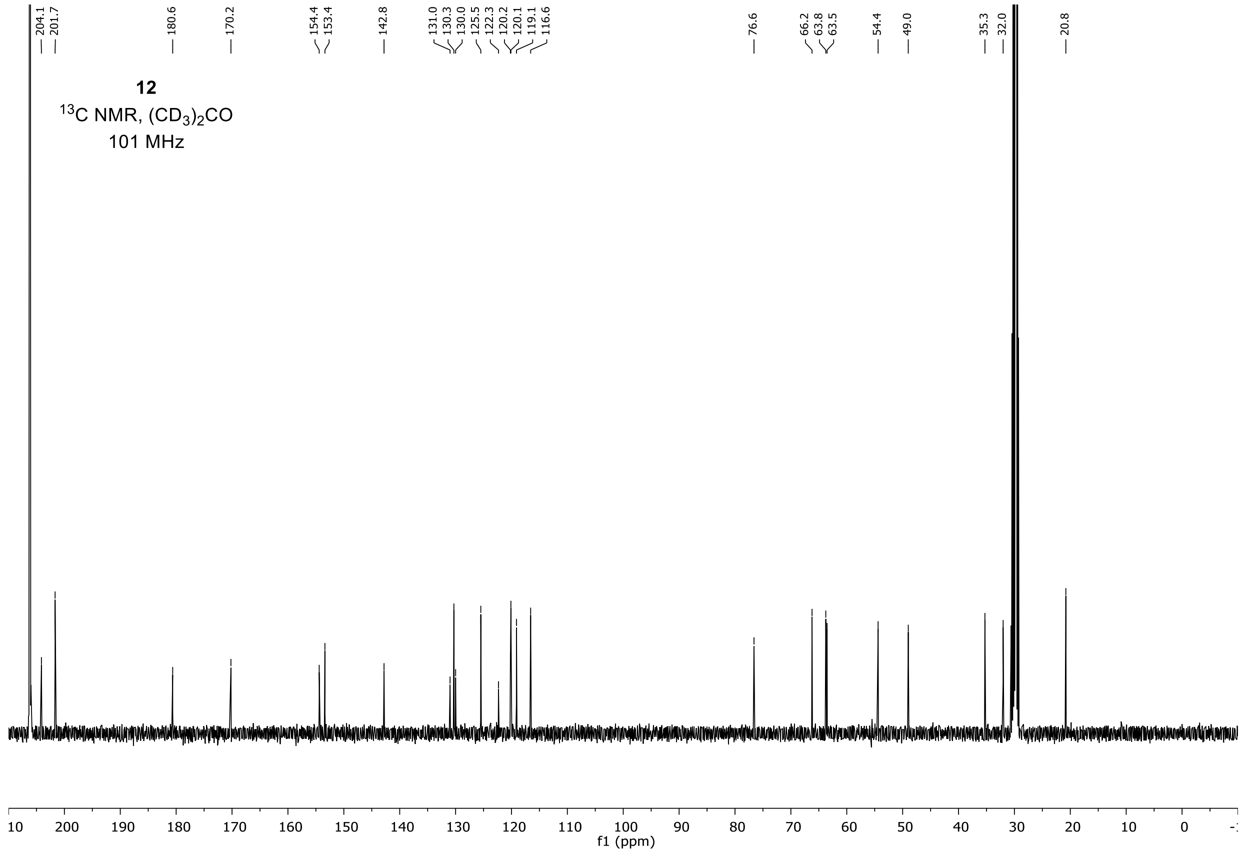
12
13C NMR, (CD3)2CO
101 MHz
f1 (ppm)

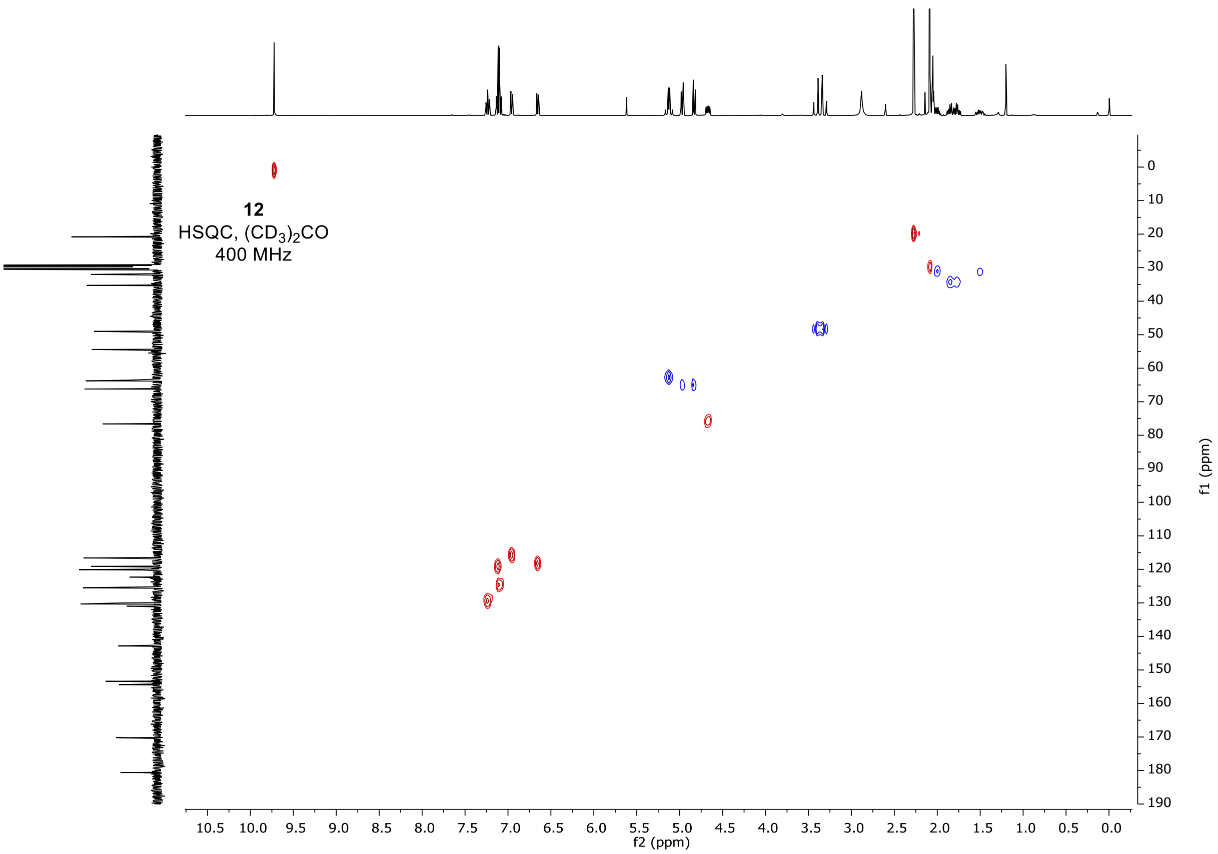
12
HSQC, $(CD_3)_2CO$
400 MHz
f2 (ppm)
f1 (ppm)

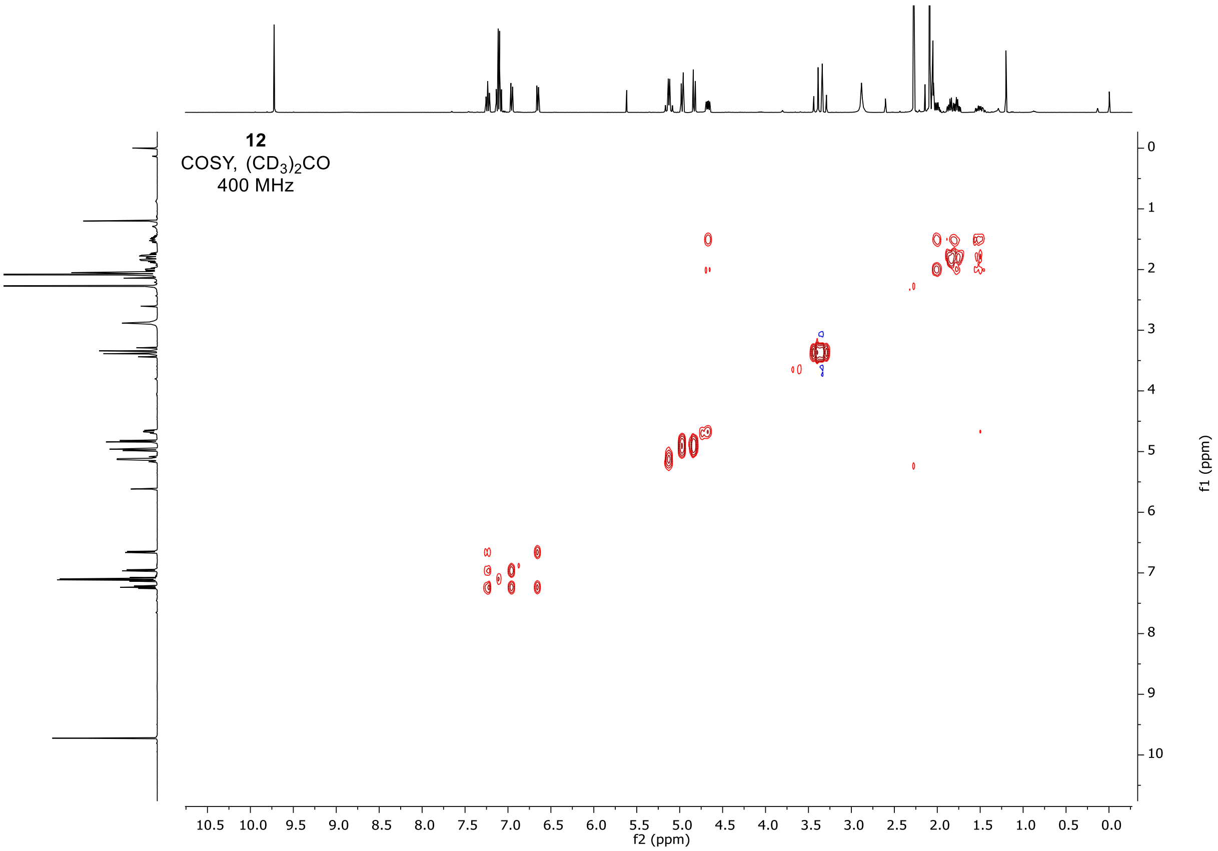
12
COSY, $(CD_3)_2CO$
400 MHz
f2 (ppm)
f1 (ppm)

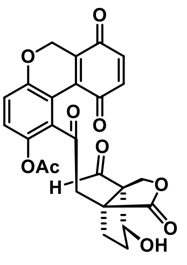

29
^{1}H NMR, $CDCl_3$
400 MHz

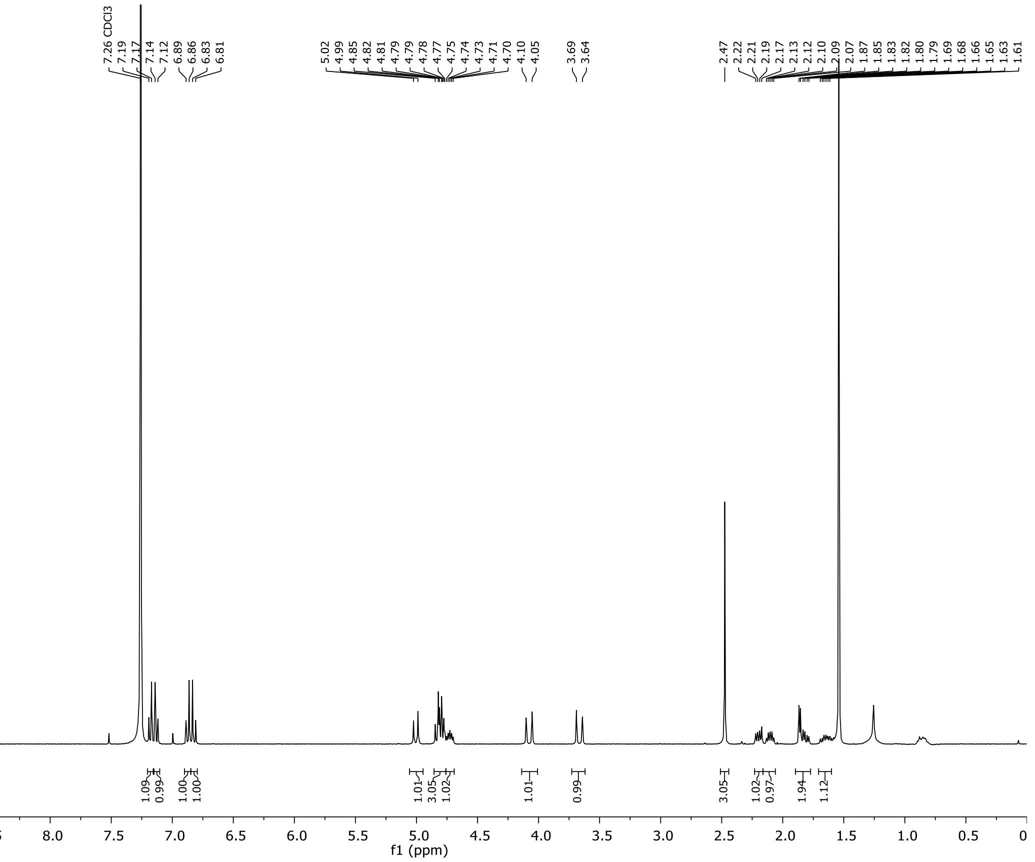

29
^{13}C NMR, $CDCl_3$
101 MHz

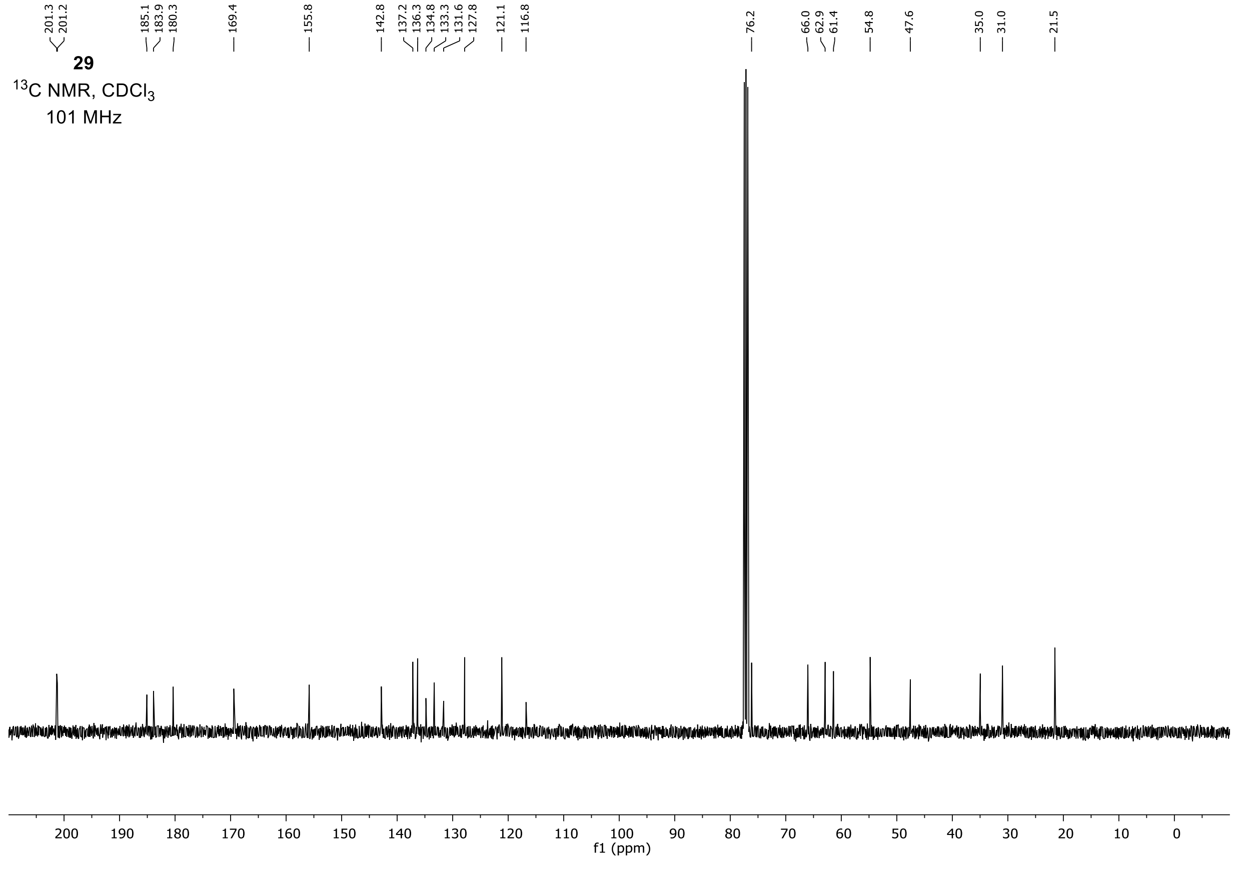

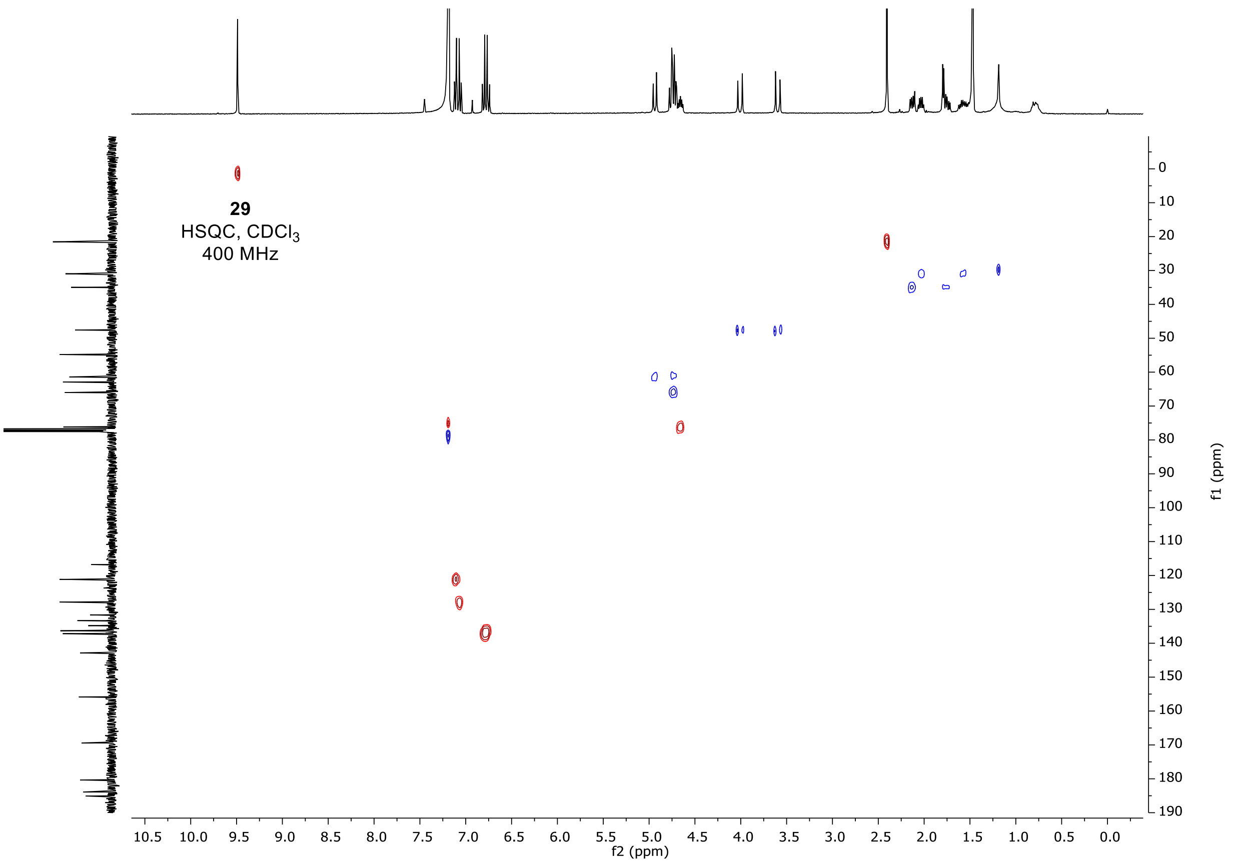
29
HSQC, CDCl3
400 MHz
f2 (ppm)
f1 (ppm)

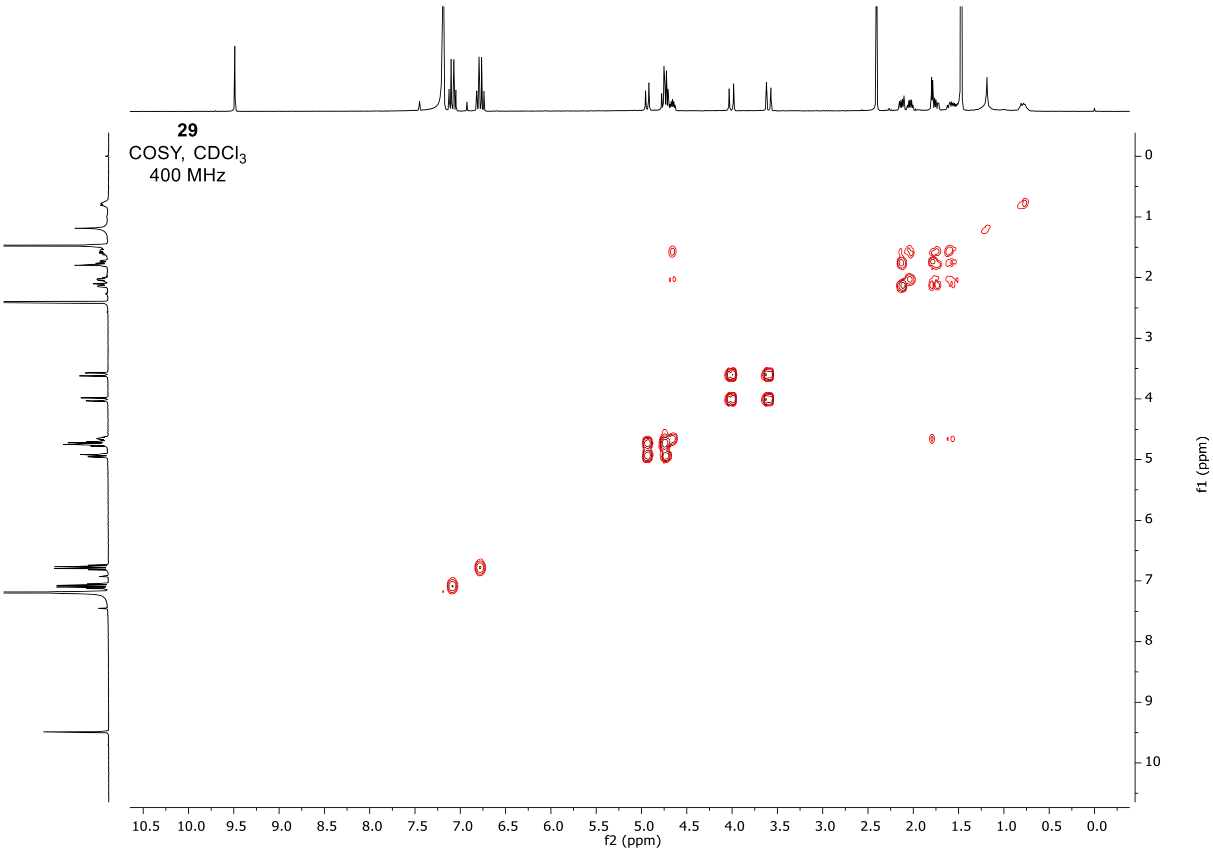
29
COSY, CDCl3
400 MHz
f2 (ppm)
f1 (ppm)

11
^{1}H NMR, $(CD_3)_2CO$
400 MHz

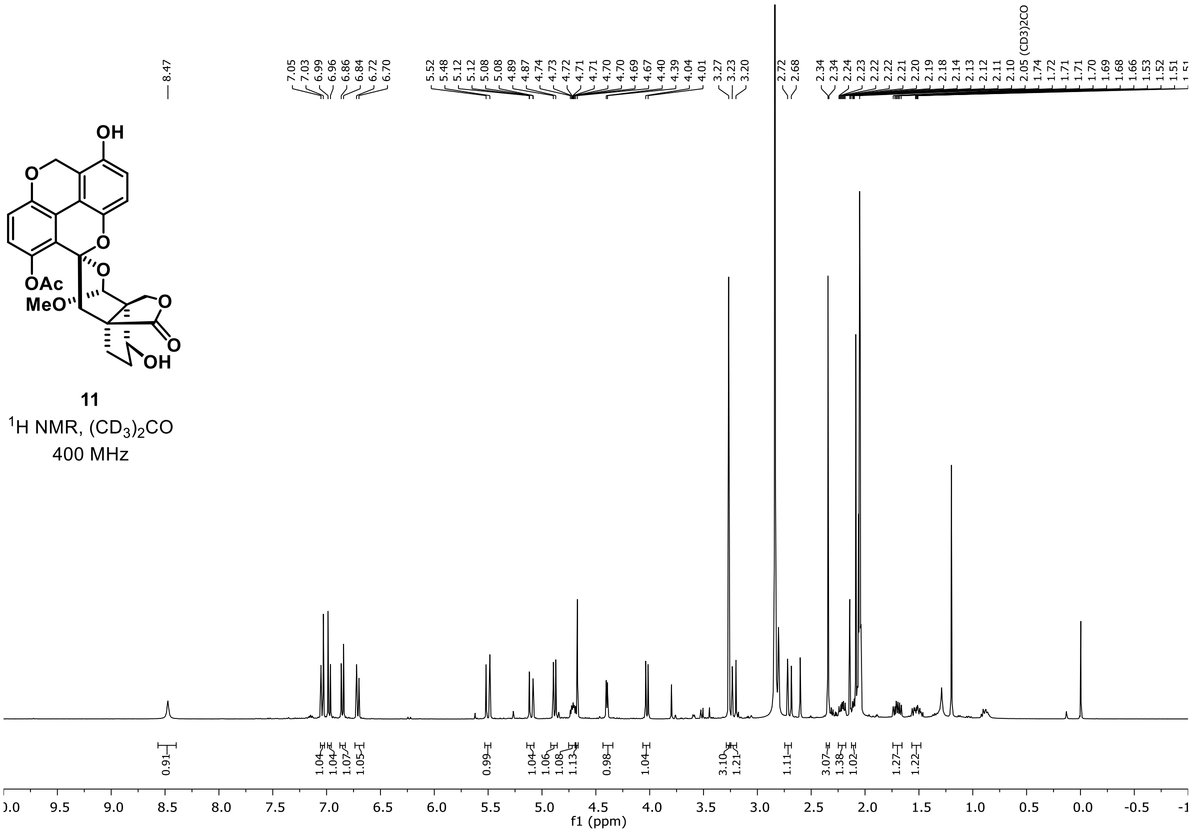

11
^{13}C NMR, $(CD_3)_2CO$
101 MHz

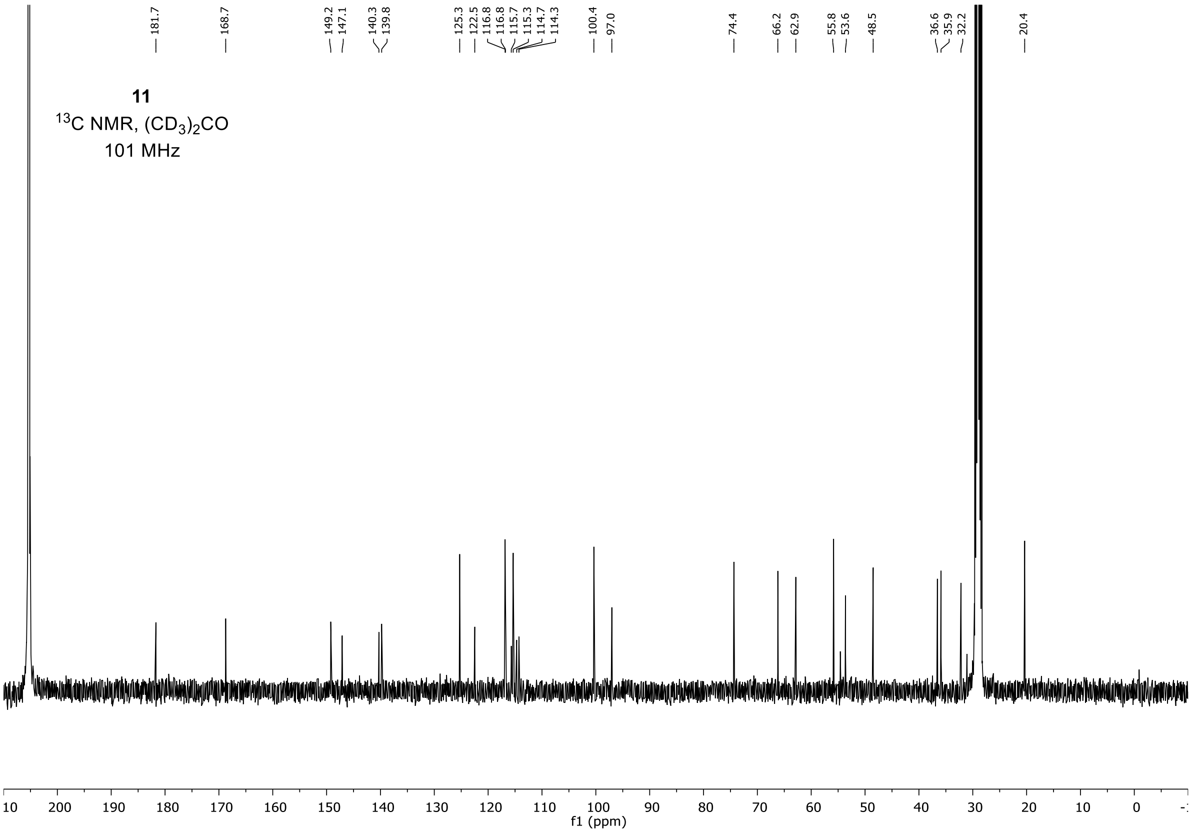

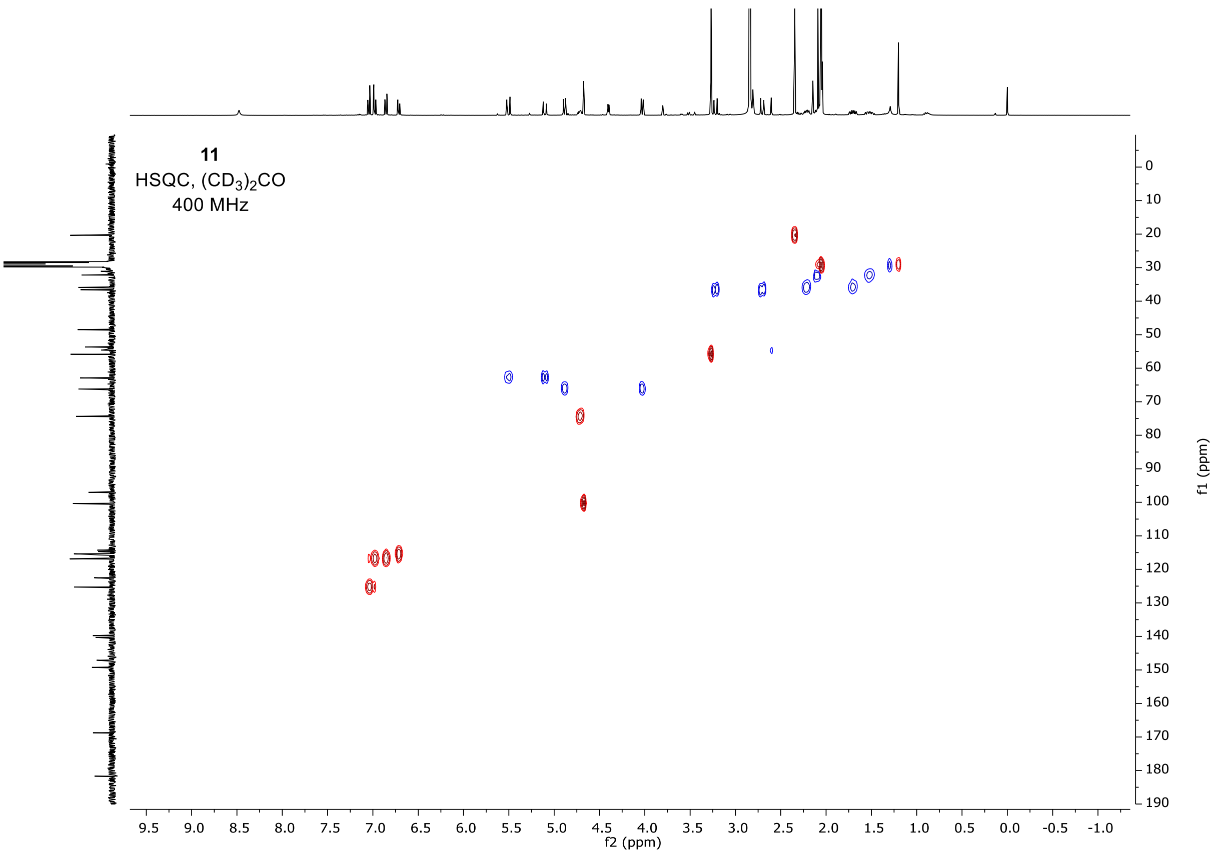
11
HSQC, $(CD_3)_2CO$
400 MHz
f2 (ppm)
f1 (ppm)

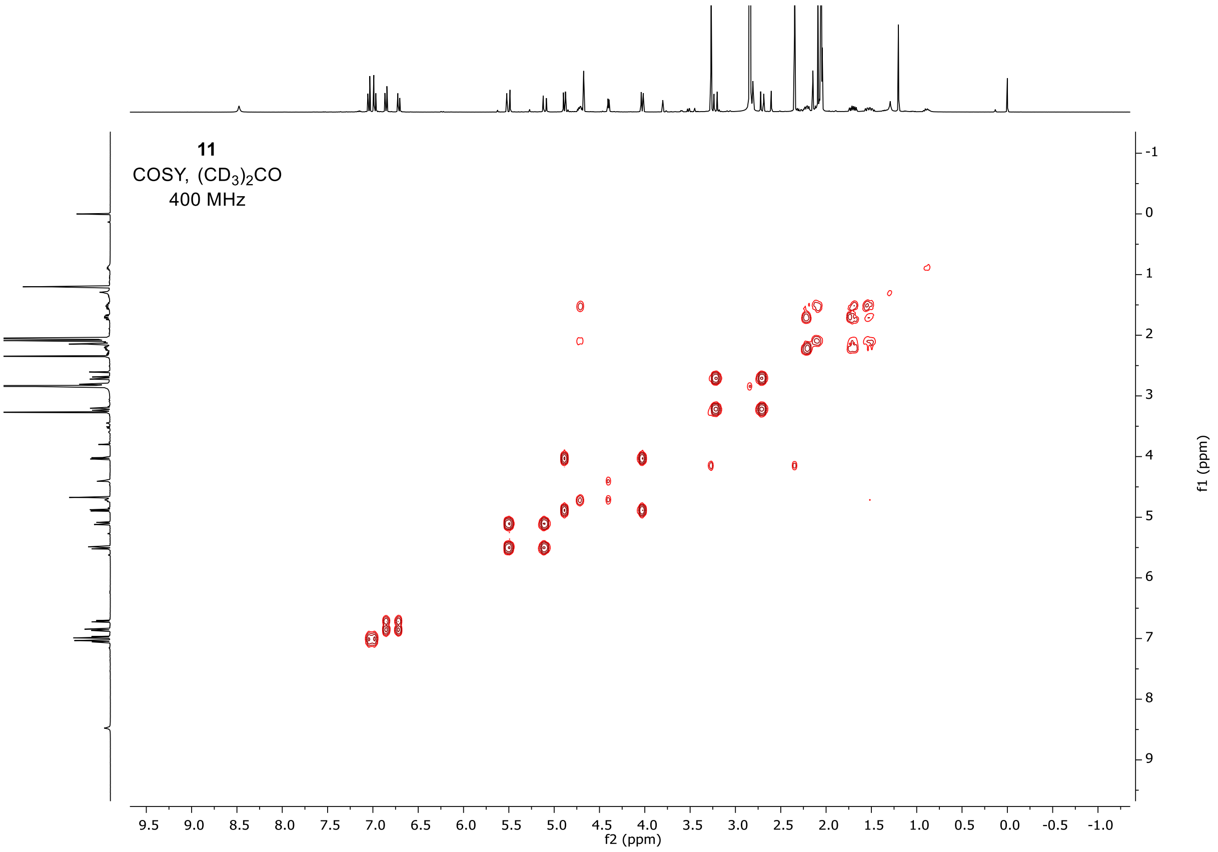
11
COSY, $(CD_3)_2CO$
400 MHz
f2 (ppm)
f1 (ppm)

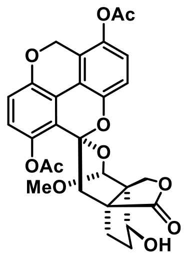

30
^{1}H NMR, $CDCl_3$
400 MHz

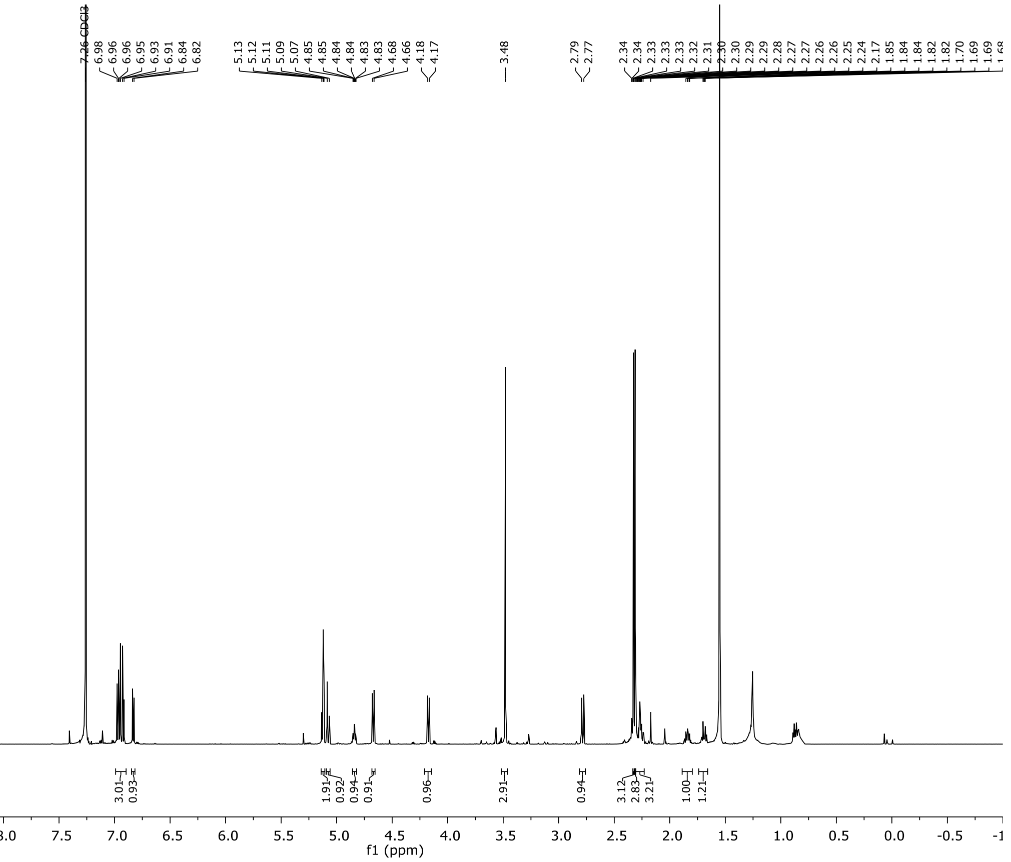

30
^{13}C NMR, $CDCl_3$
101 MHz

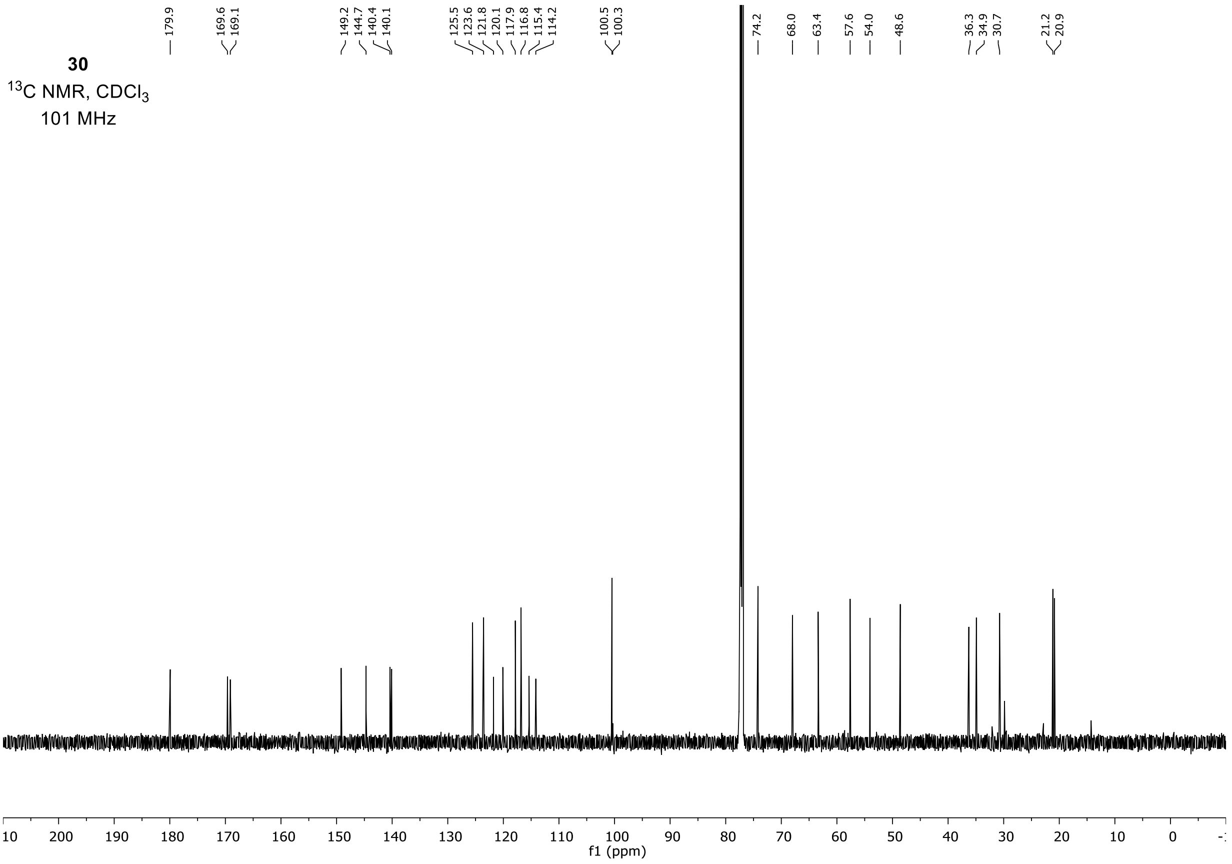

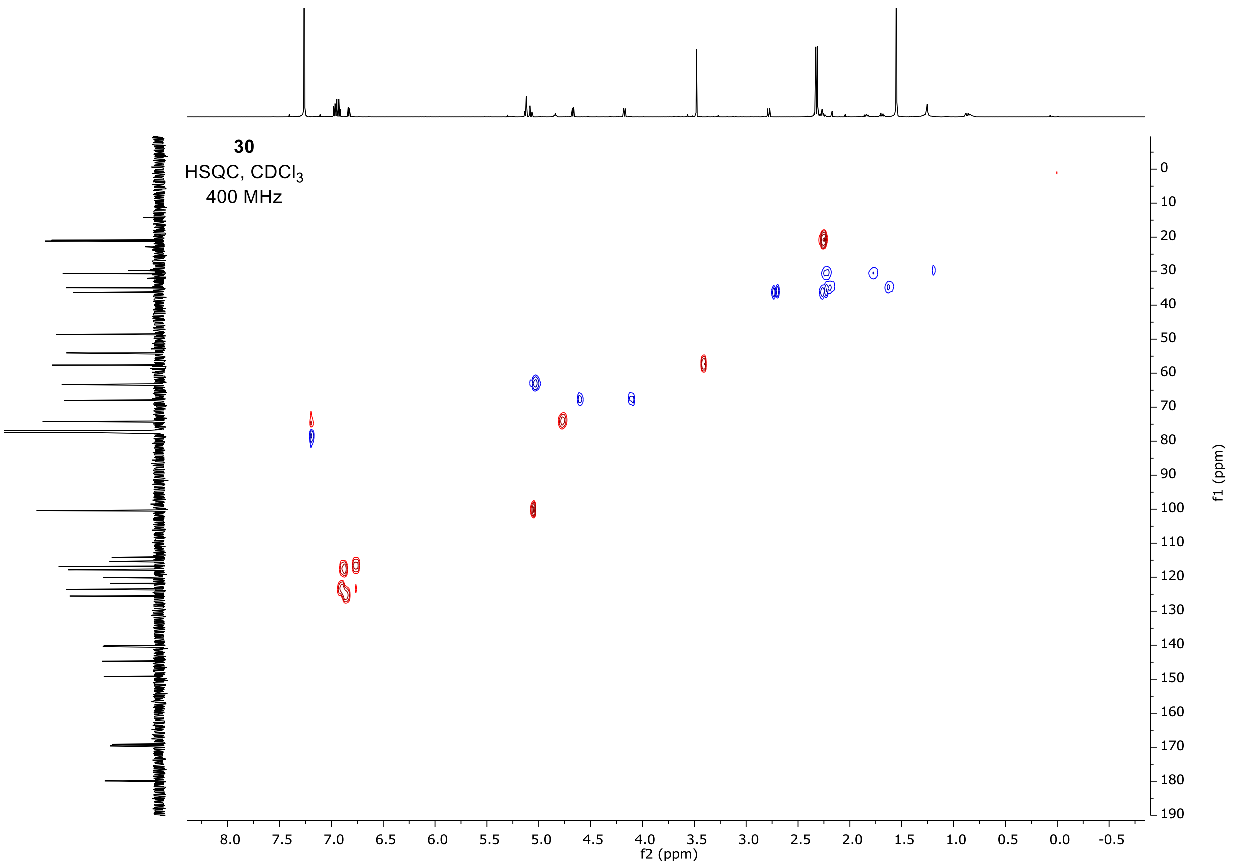
30
HSQC, CDCl3
400 MHz
f2 (ppm)
f1 (ppm)

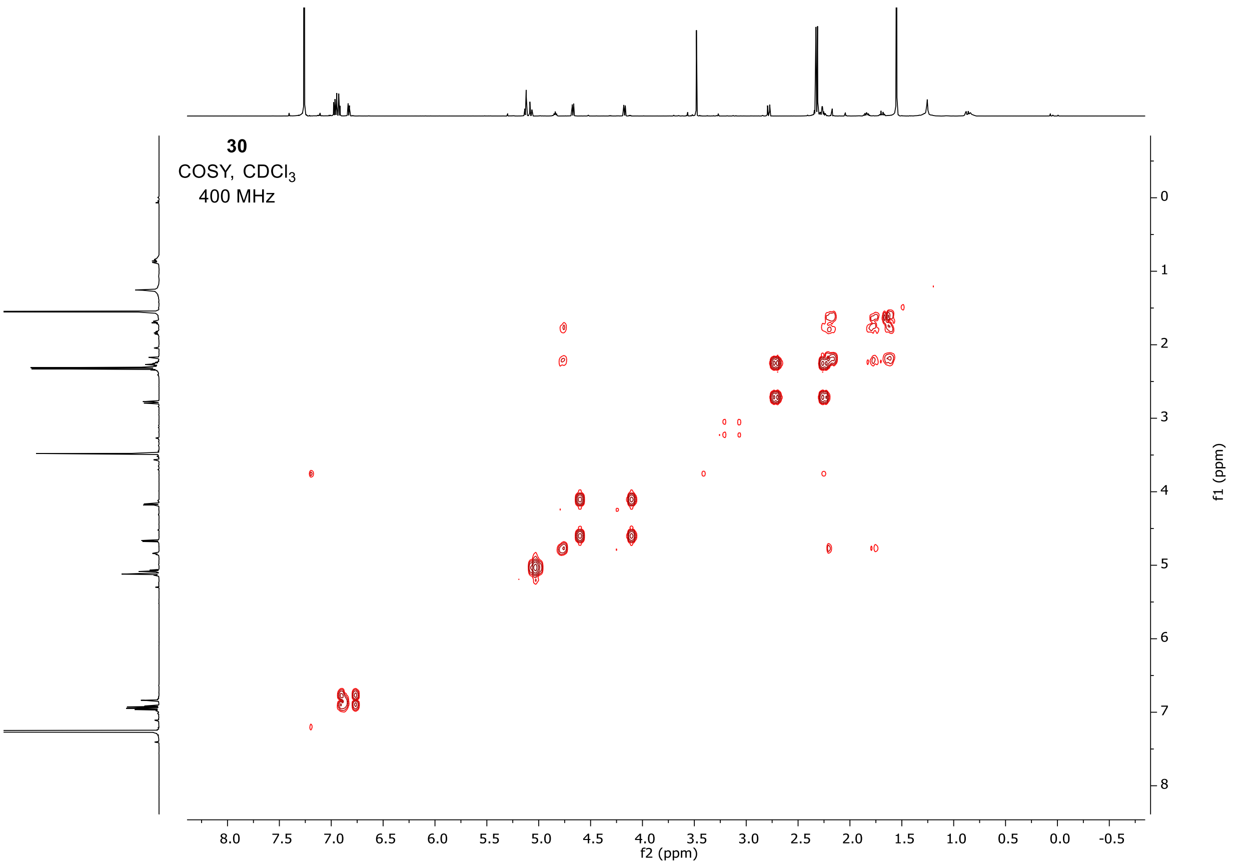
30
COSY, CDCl3
400 MHz
f2 (ppm)
f1 (ppm)

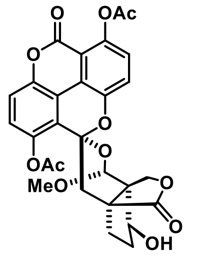

31
^{1}H NMR, $CDCl_3$
700 MHz

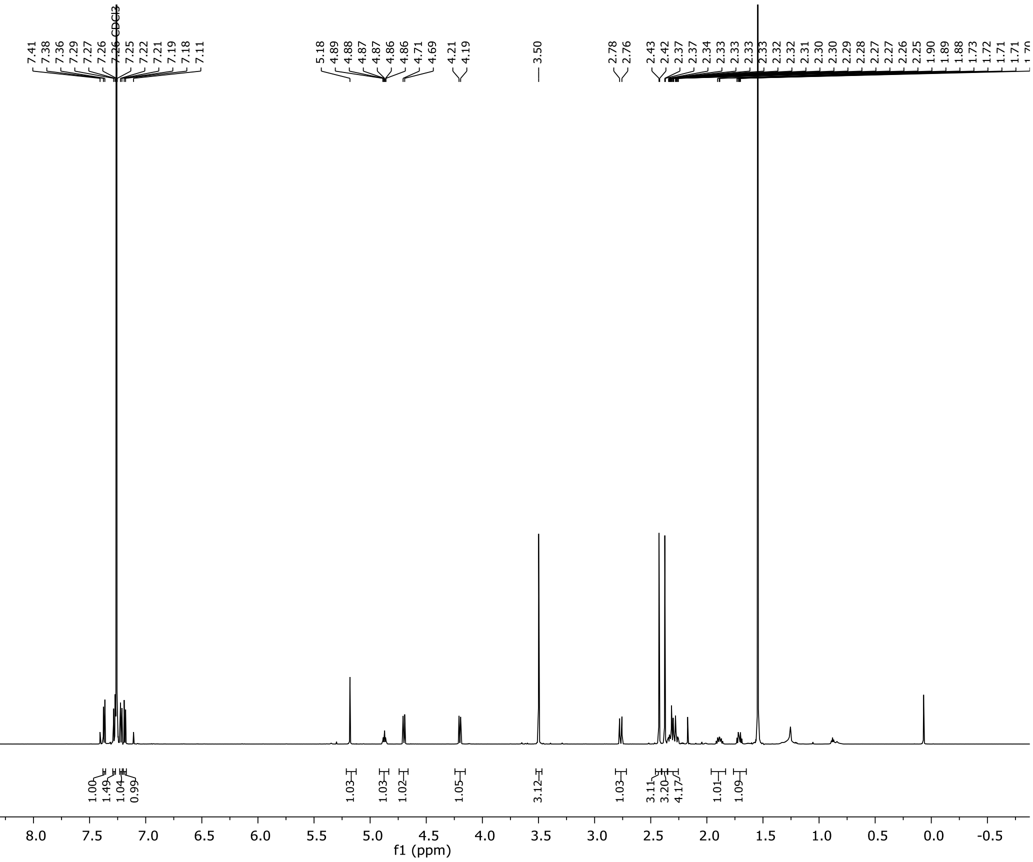

31
^{13}C NMR, $CDCl_3$
176 MHz

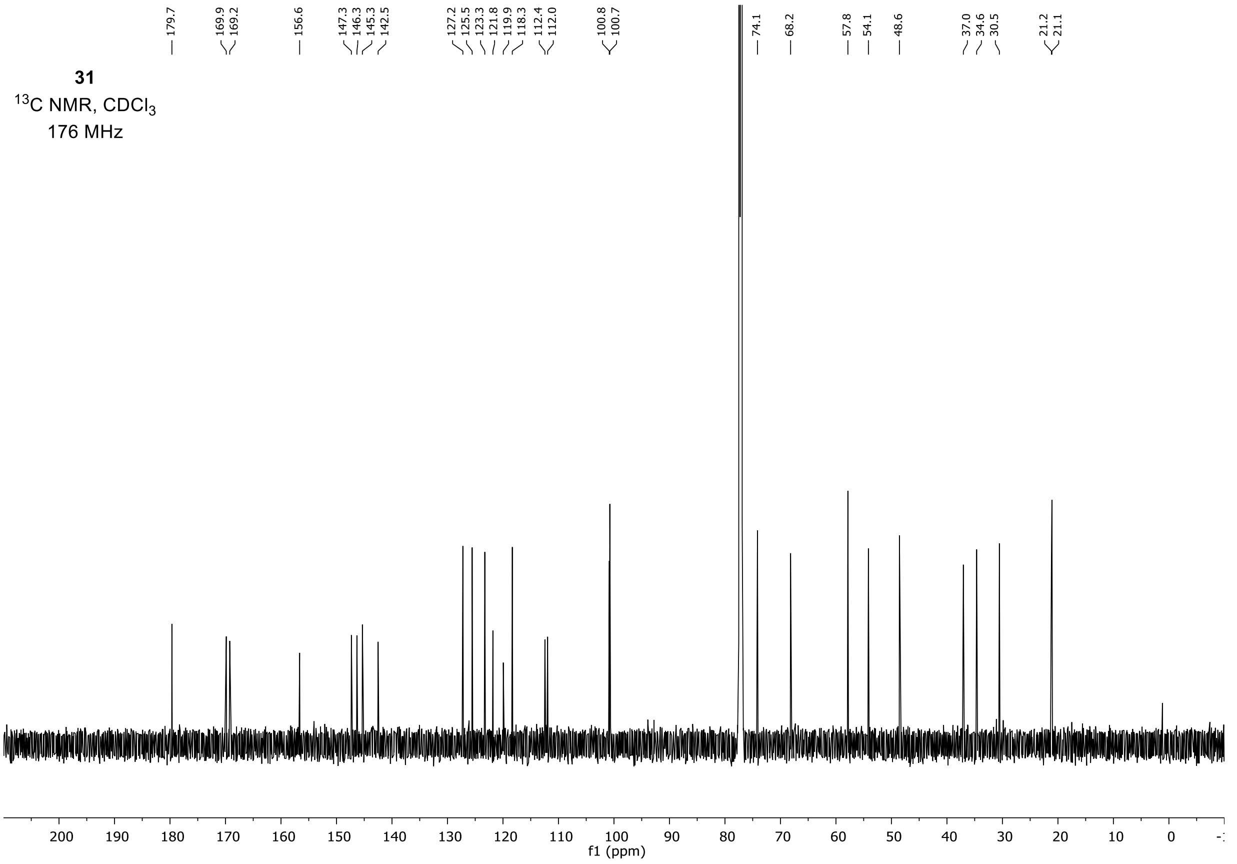

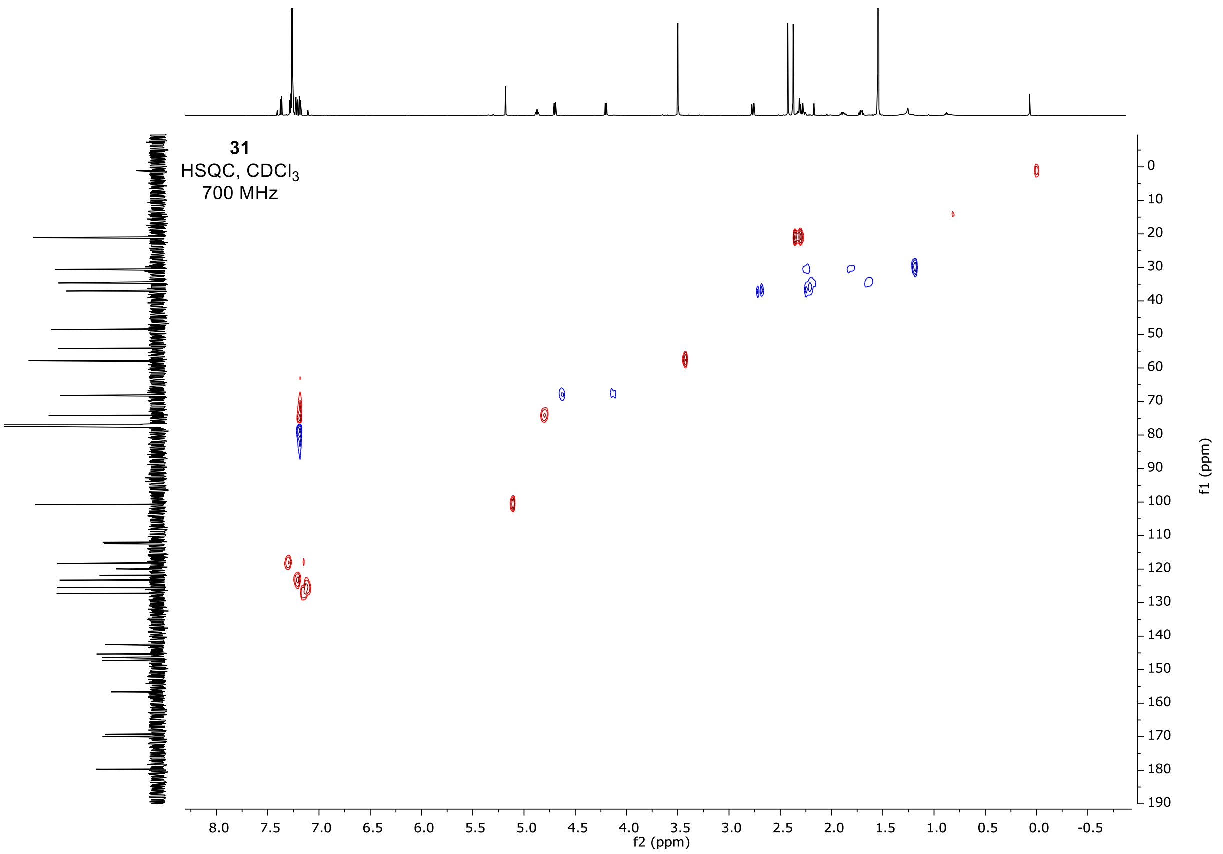

31
HSQC, $CDCl_3$
700 MHz
f1 (ppm)
f2 (ppm)

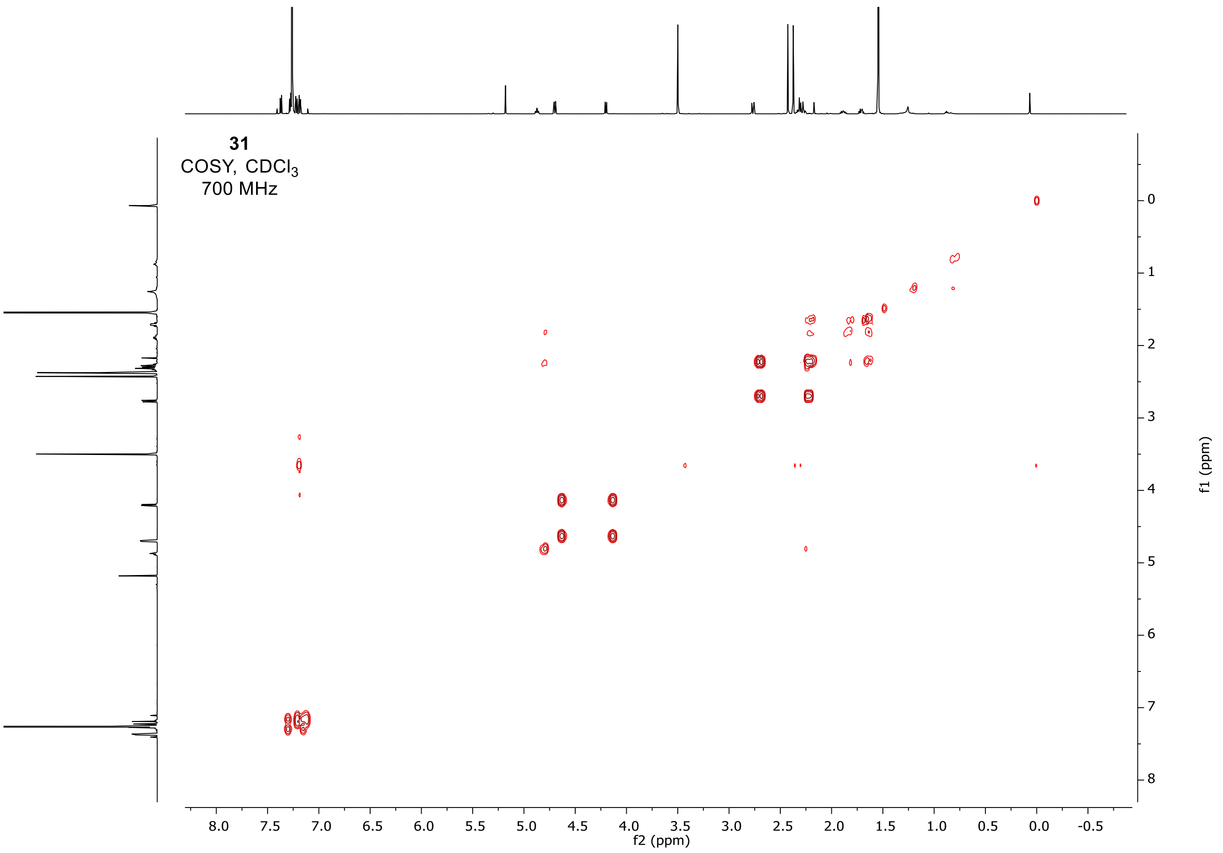

31
COSY, $CDCl_3$
700 MHz
f1 (ppm)
f2 (ppm)

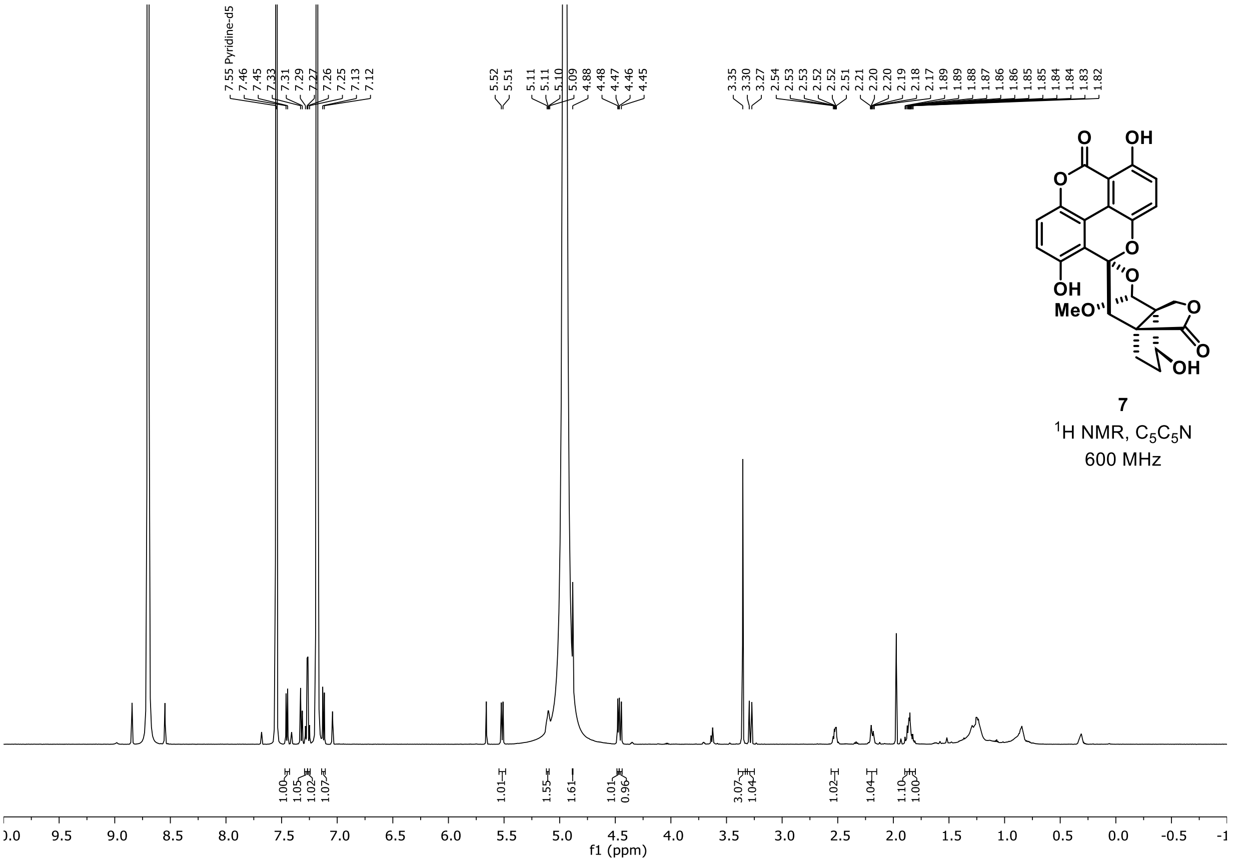
7
^{1}H NMR, C_5C_5N
600 MHz
f1 (ppm)

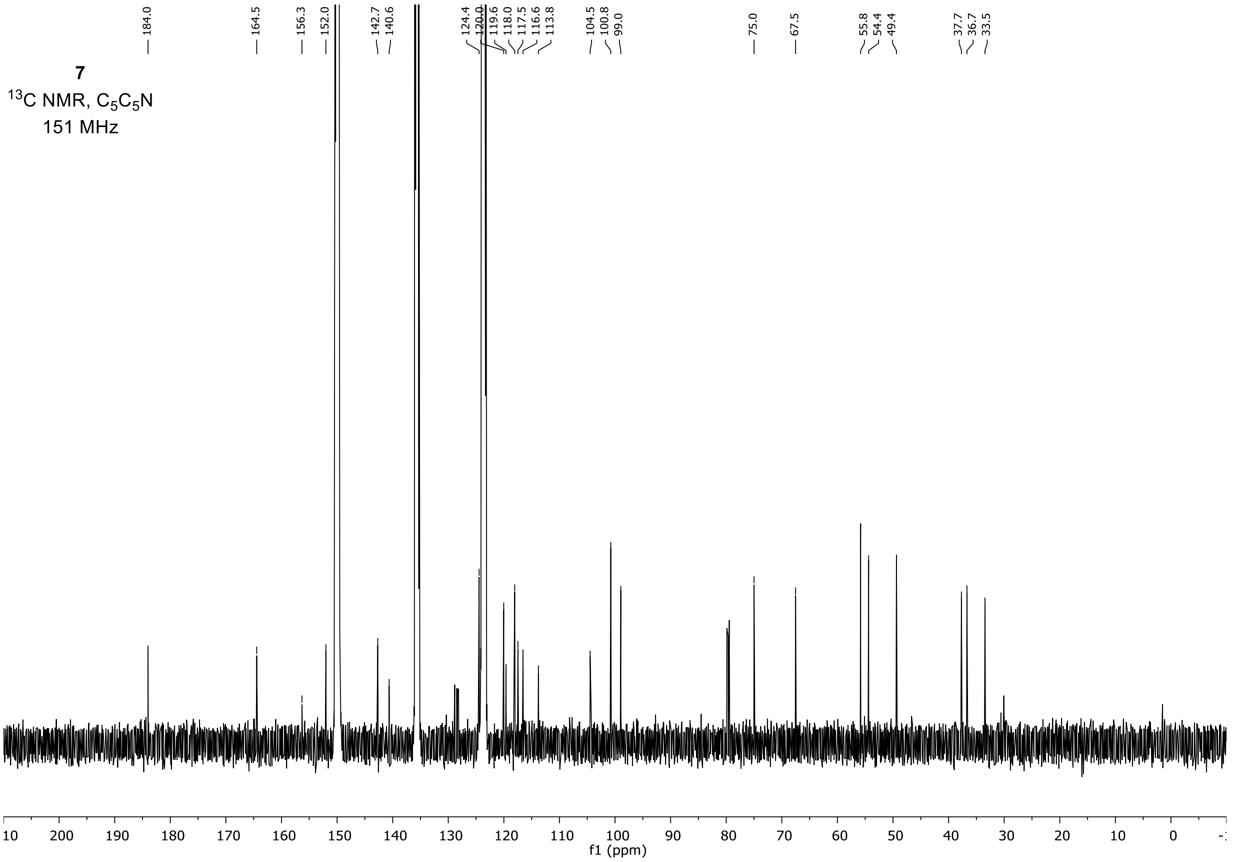
7
^{13}C NMR, C_5C_5N
151 MHz
f1 (ppm)

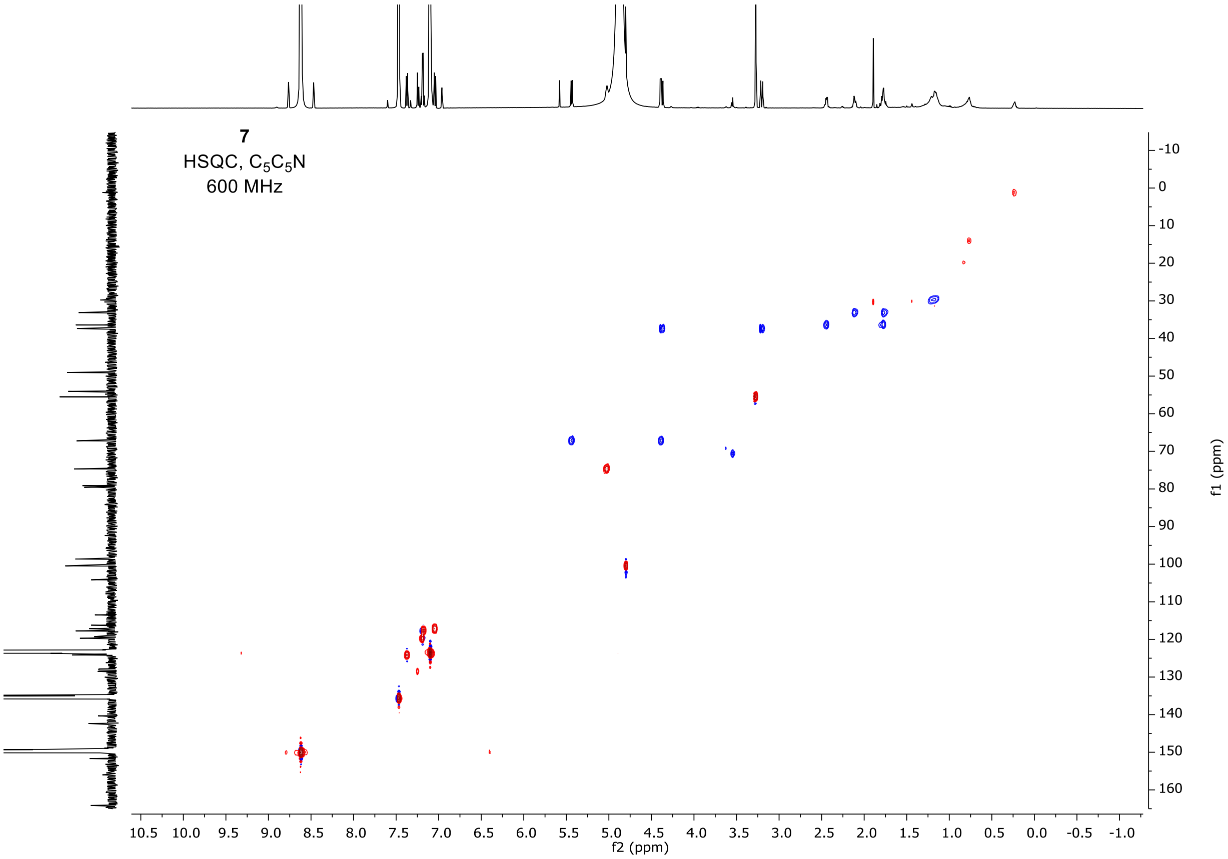

7
HSQC, C5C5N
600 MHz
f2 (ppm)
f1 (ppm)

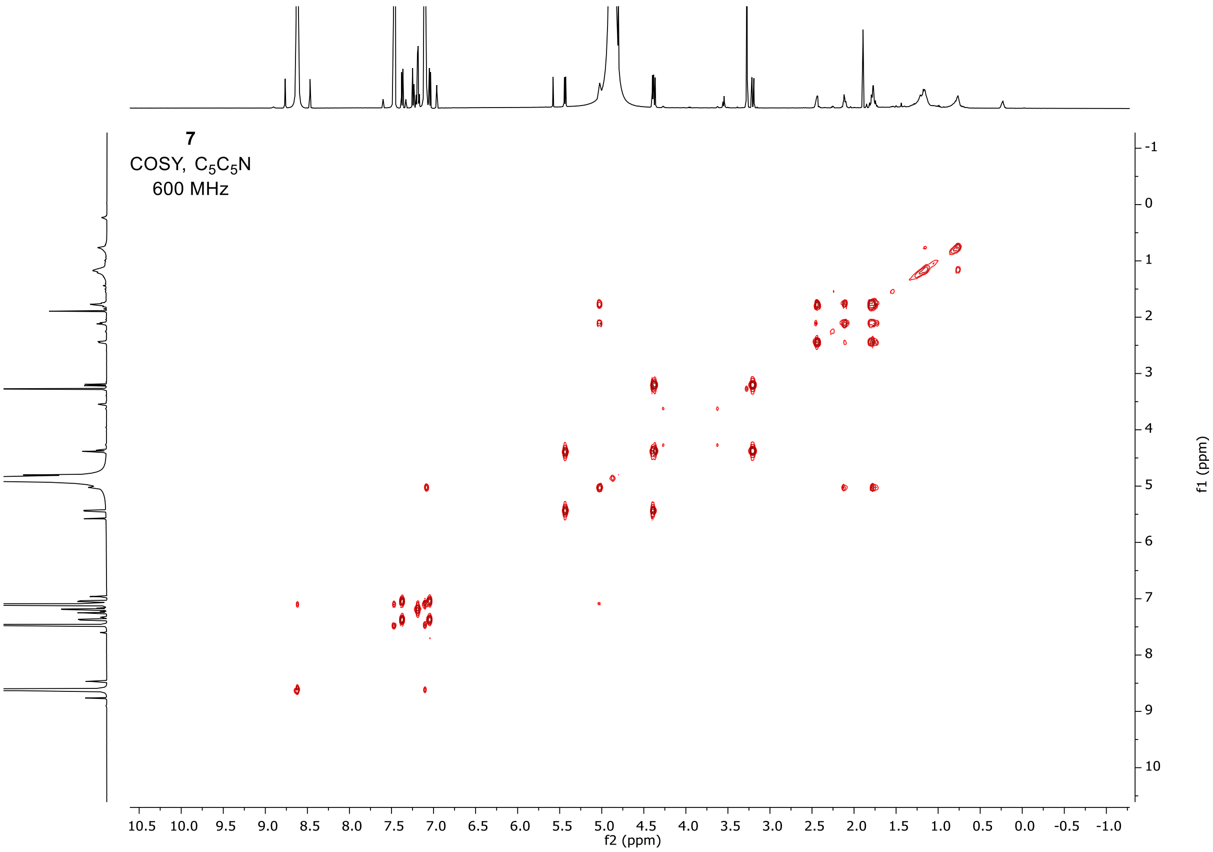

7
COSY, C5C5N
600 MHz
f2 (ppm)
f1 (ppm)

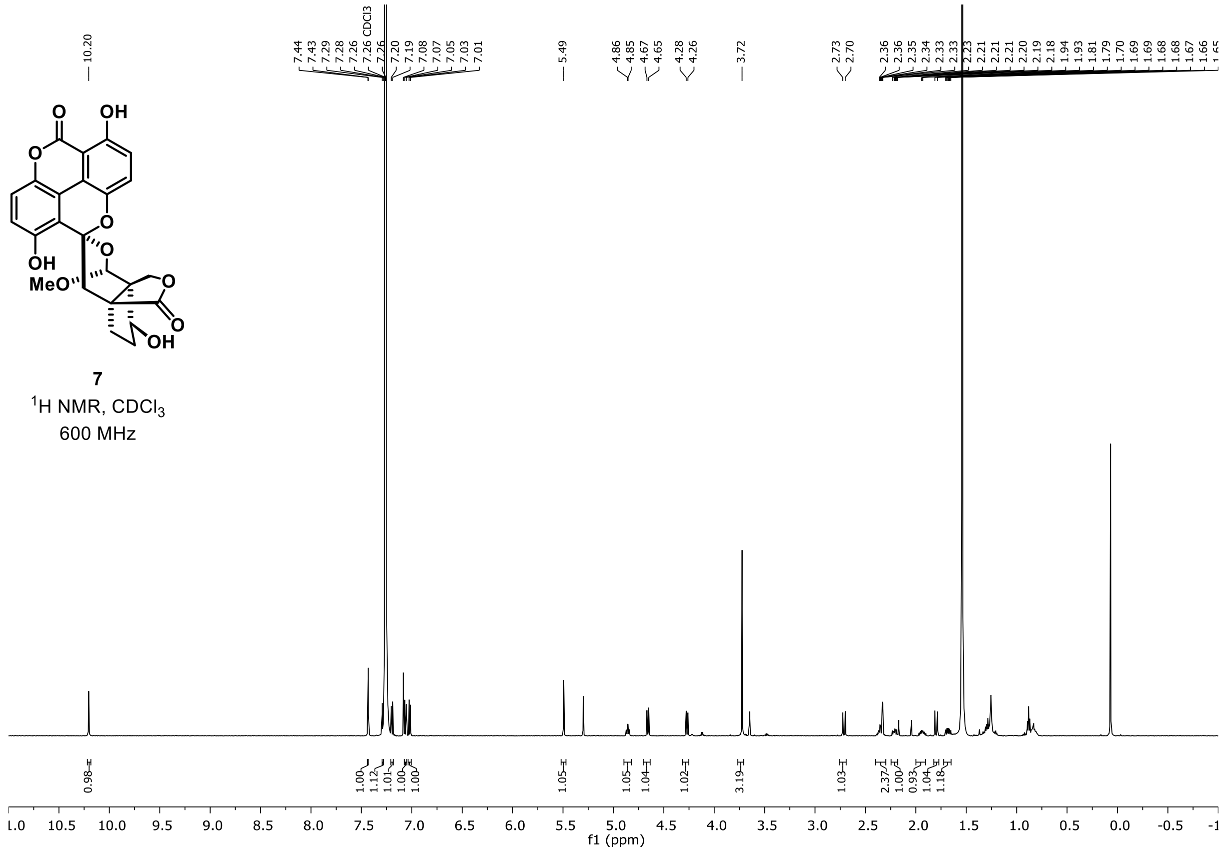

7
^{1}H NMR, $CDCl_3$
600 MHz

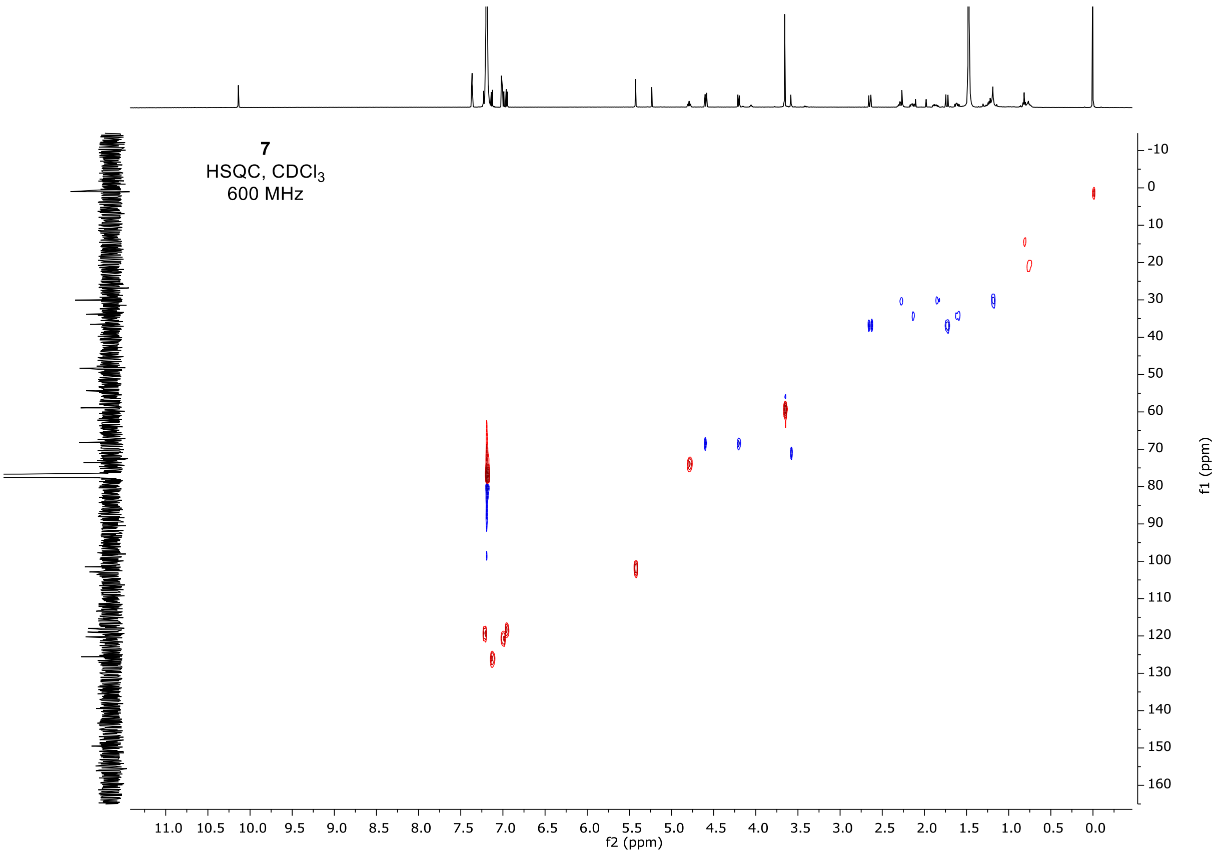

7
HSQC, $CDCl_3$
600 MHz

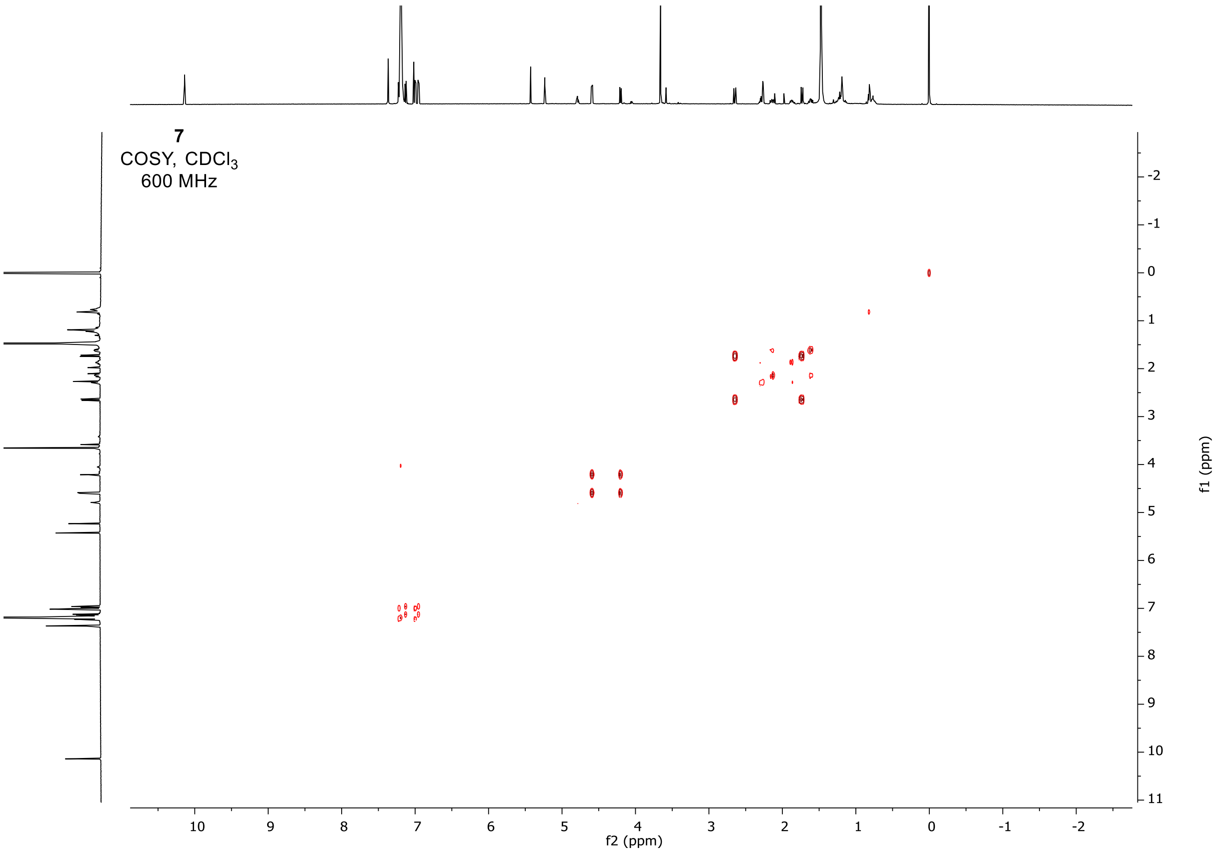

7
COSY, CDCl3
600 MHz
f2 (ppm)
f1 (ppm)

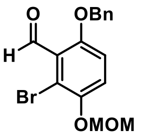

S15
^{1}H NMR, $CDCl_3$
400 MHz

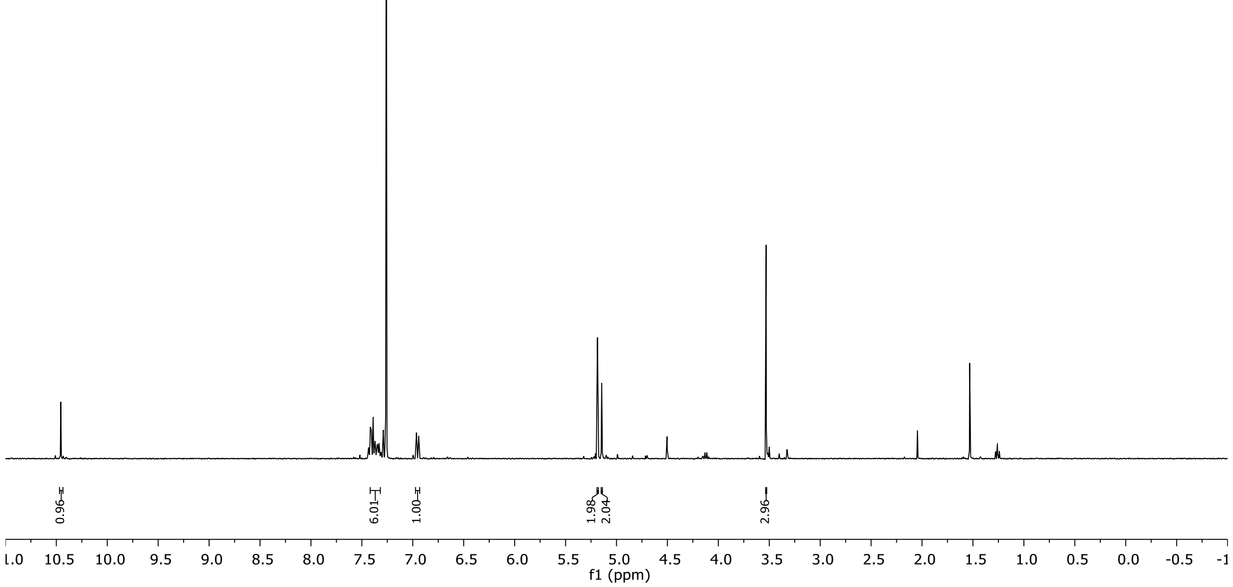

S15
^{13}C NMR, $CDCl_3$
101 MHz

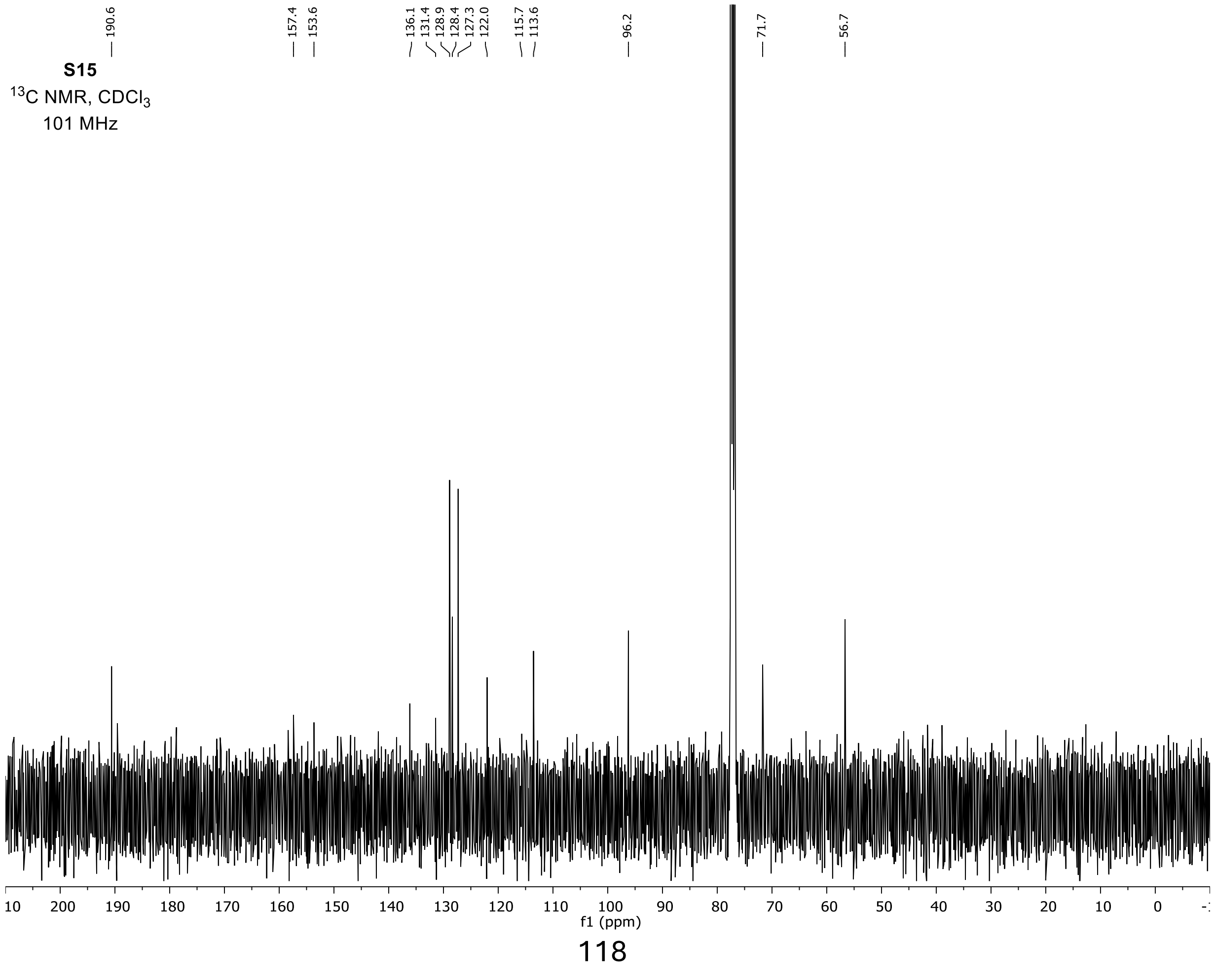

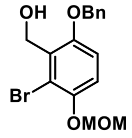

S16
^{1}H NMR, $CDCl_3$
400 MHz

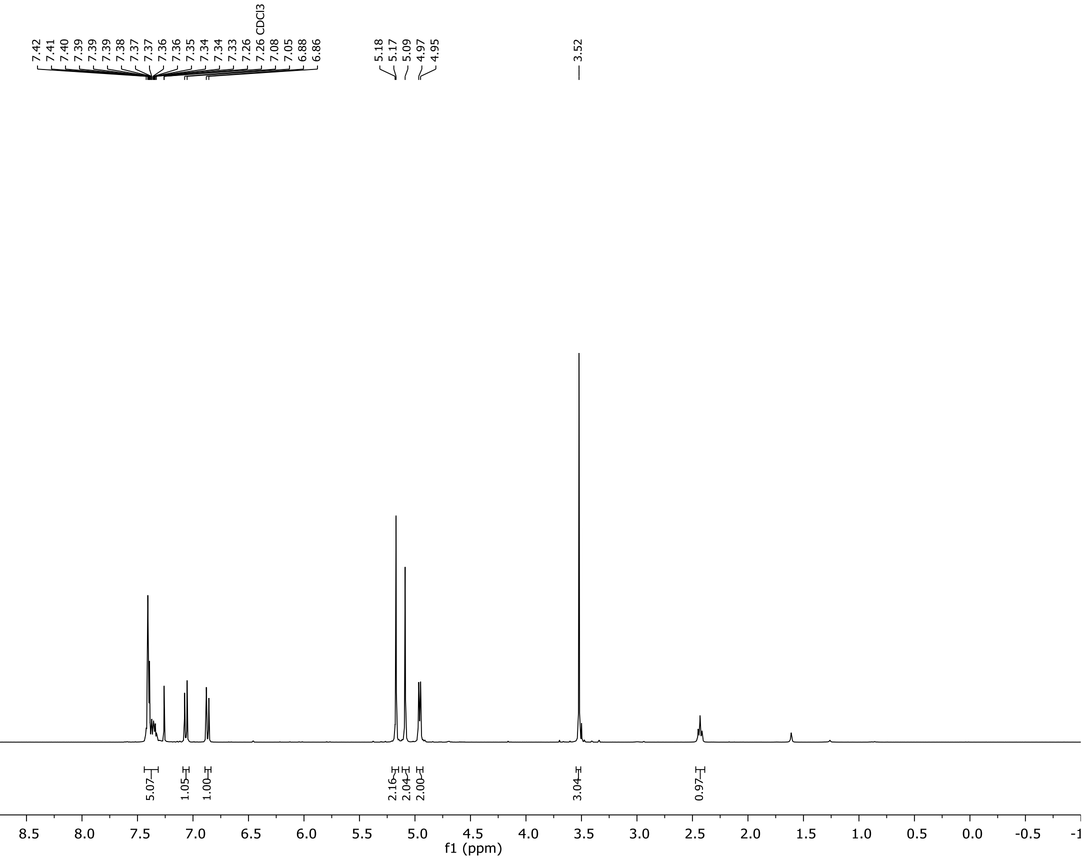

S16
^{13}C NMR, $CDCl_3$
101 MHz

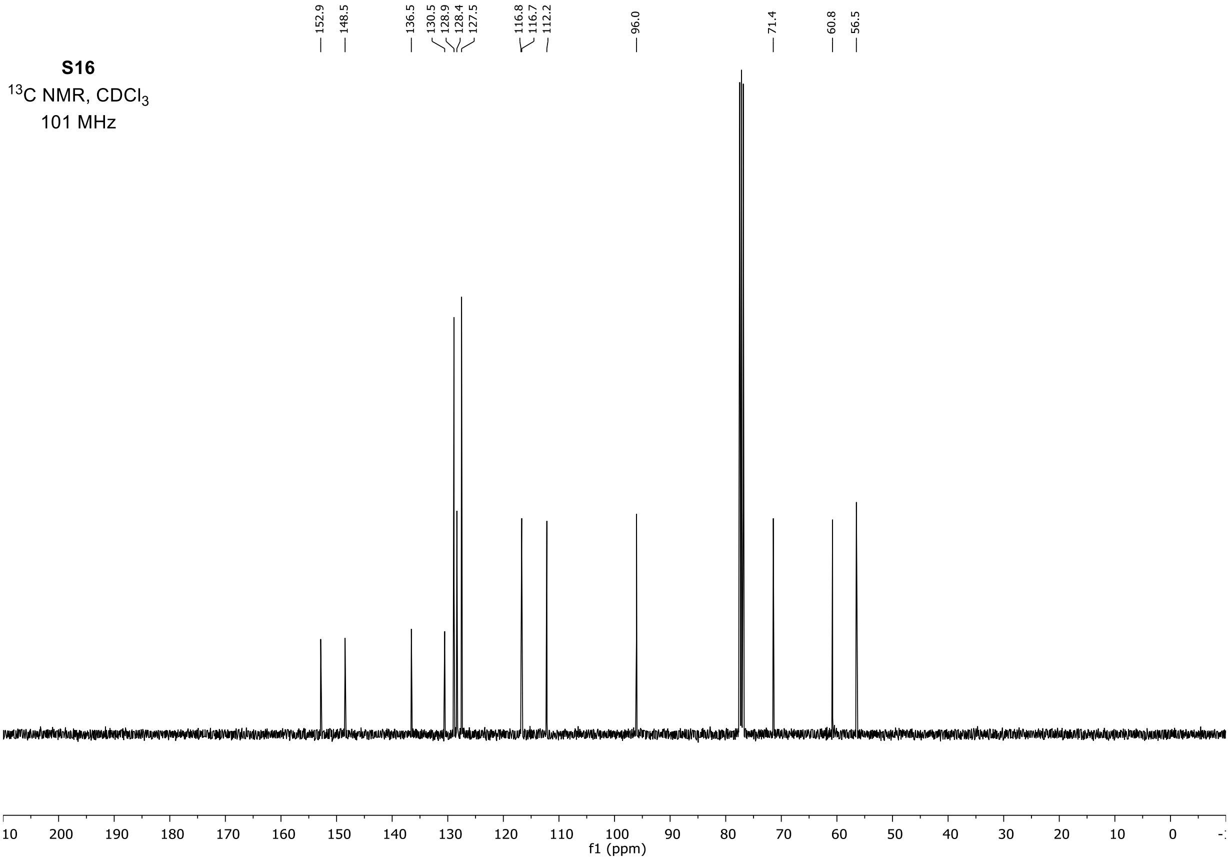

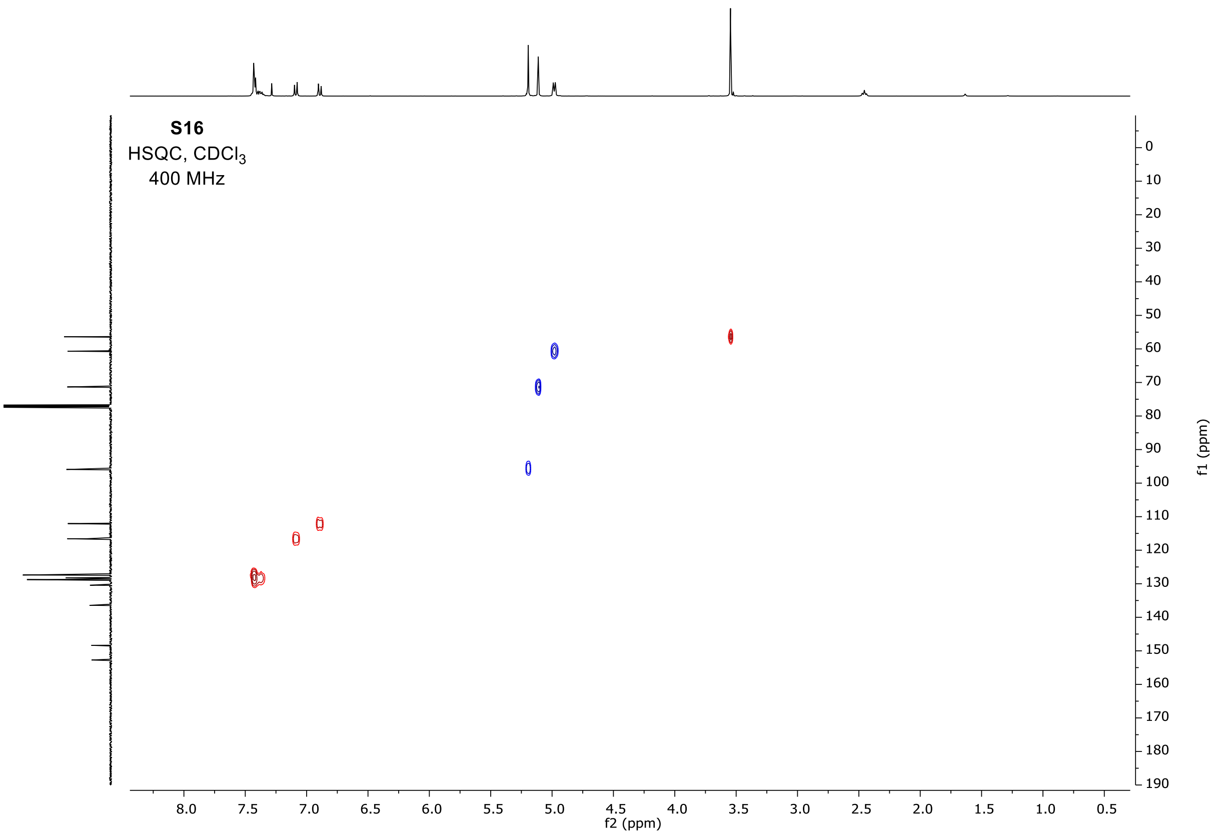
S16
HSQC, CDCl3
400 MHz
f1 (ppm)
f2 (ppm)
0
10
20
30
40
50
60
70
80
90
100
110
120
130
140
150
160
170
180
190
8.0
7.5
7.0
6.5
6.0
5.5
5.0
4.5
4.0
3.5
3.0
2.5
2.0
1.5
1.0
0.5

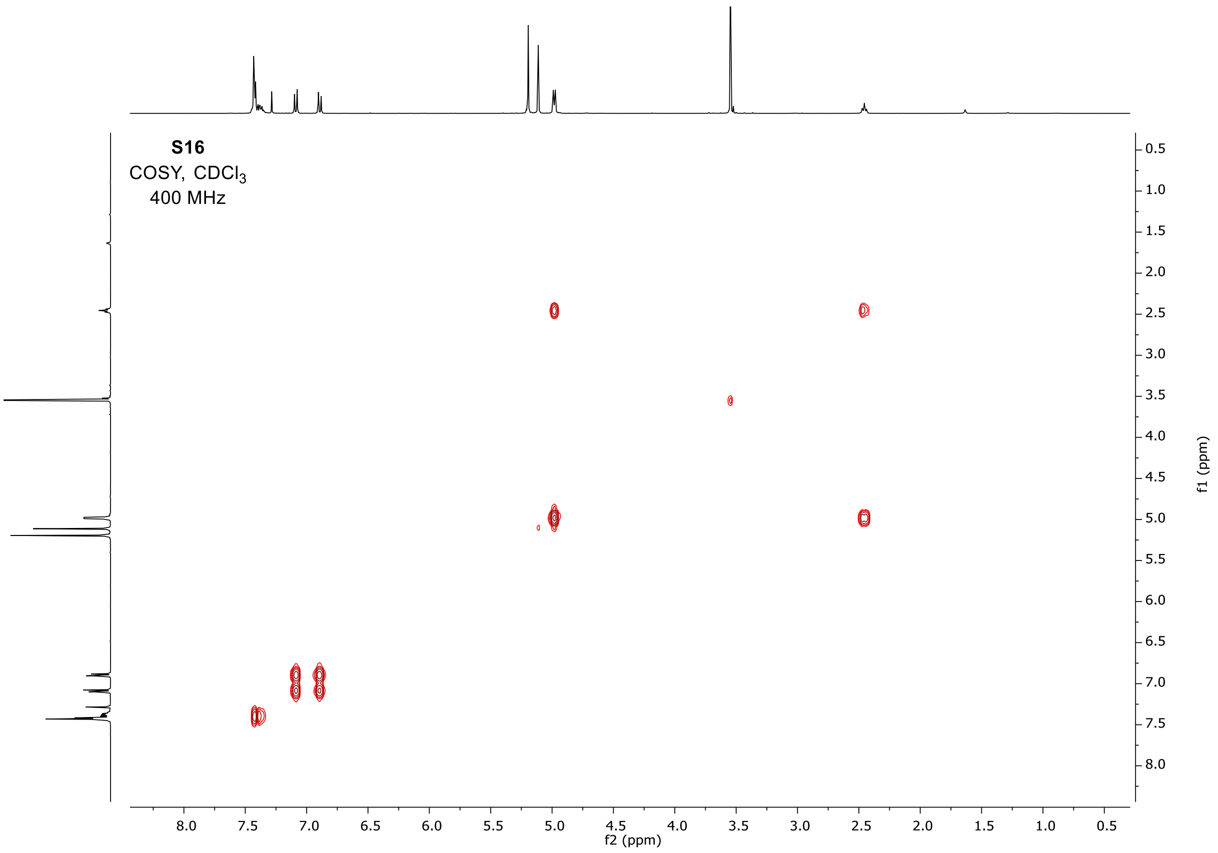
S16
COSY, CDCl3
400 MHz
f1 (ppm)
f2 (ppm)
0.5
1.0
1.5
2.0
2.5
3.0
3.5
4.0
4.5
5.0
5.5
6.0
6.5
7.0
7.5
8.0
8.0
7.5
7.0
6.5
6.0
5.5
5.0
4.5
4.0
3.5
3.0
2.5
2.0
1.5
1.0
0.5

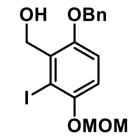

S17
^{1}H NMR, $CDCl_3$
400 MHz

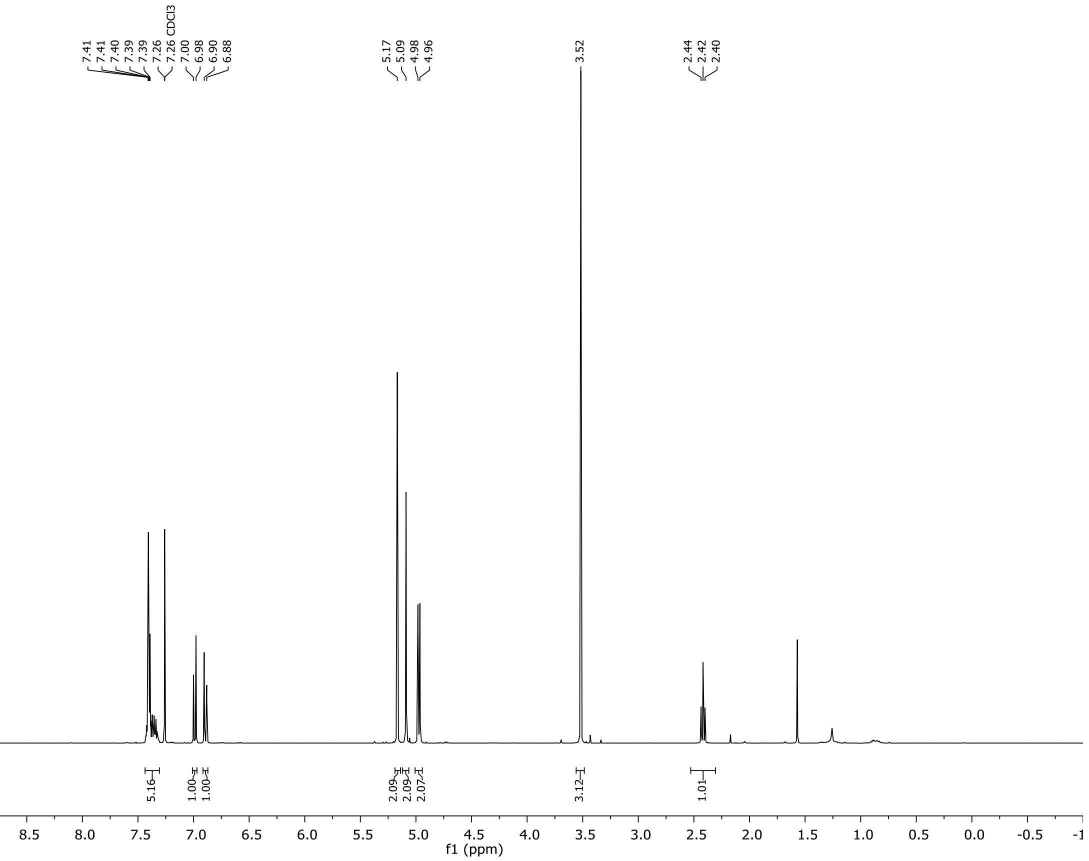

S17
^{13}C NMR, $CDCl_3$
101 MHz

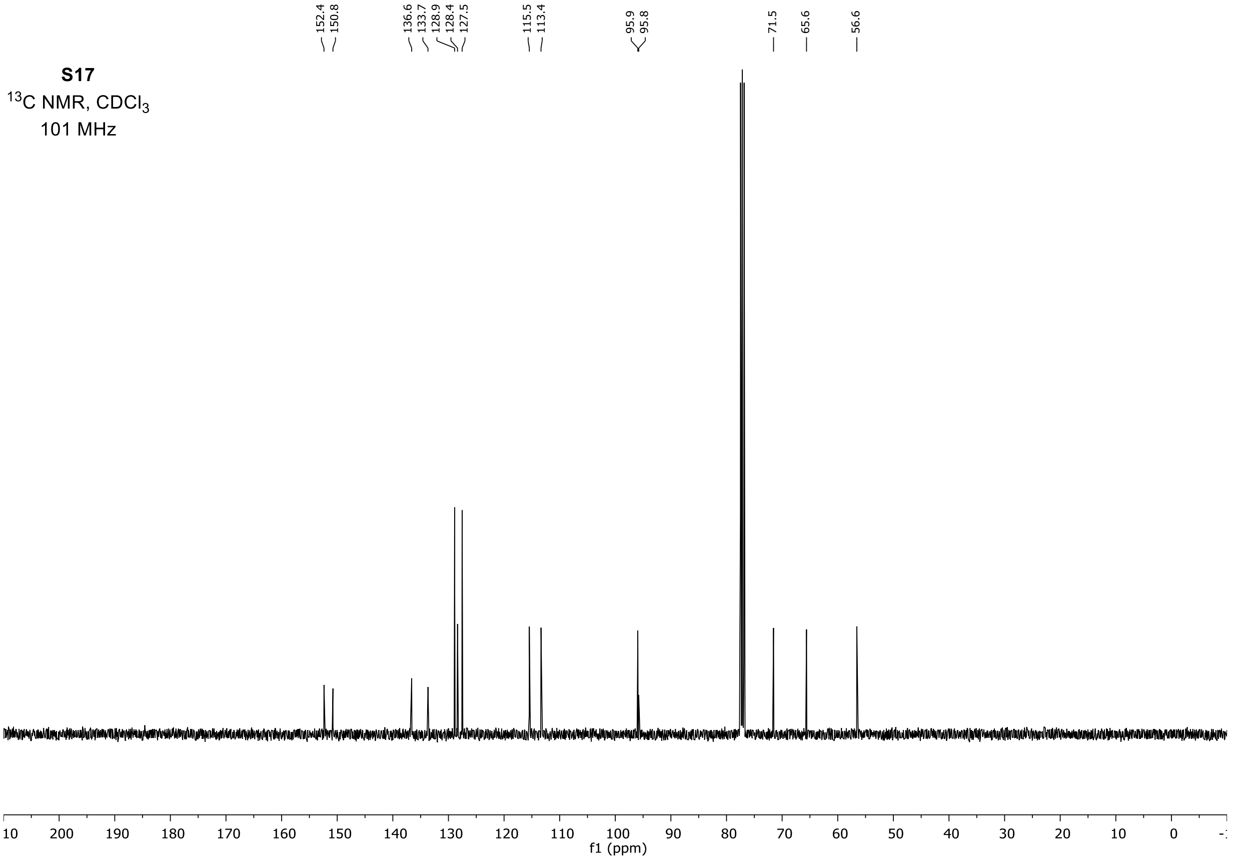

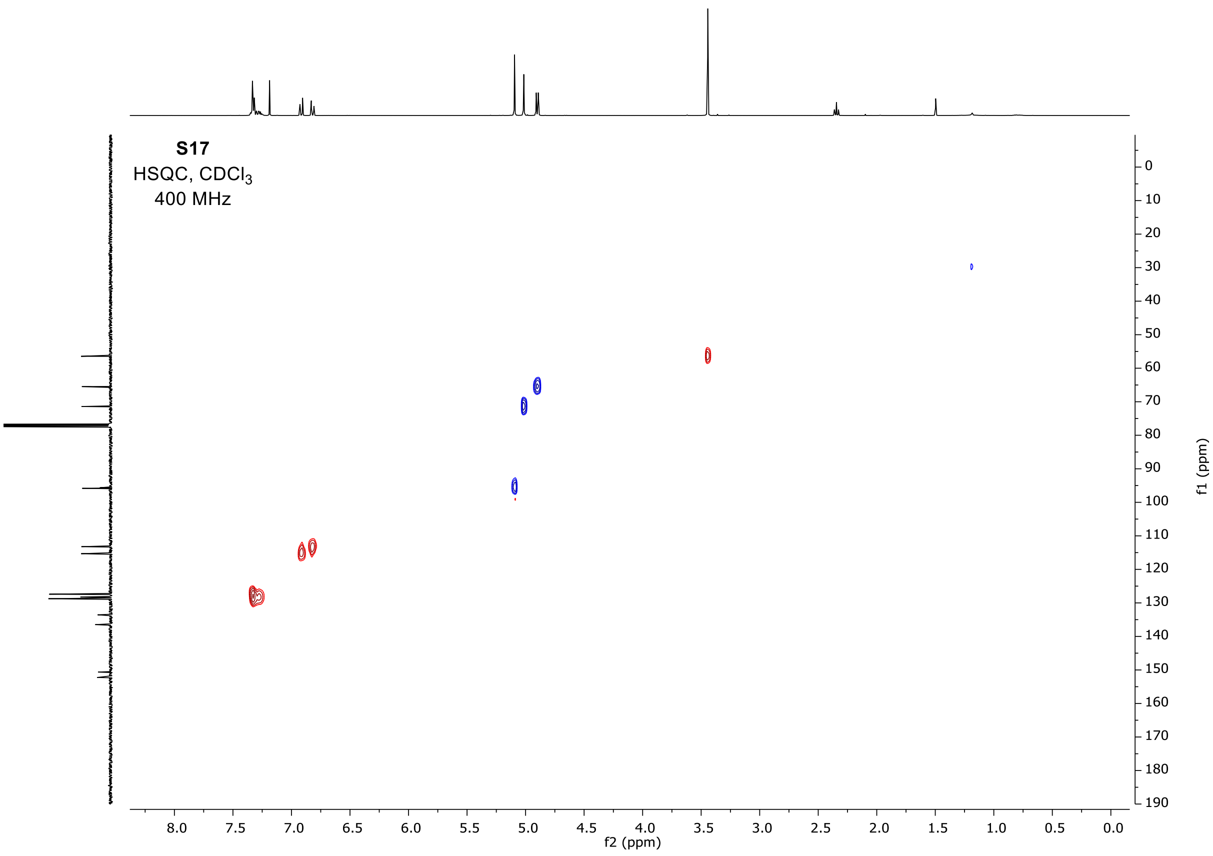
S17
HSQC, CDCl3
400 MHz
f1 (ppm)
f2 (ppm)

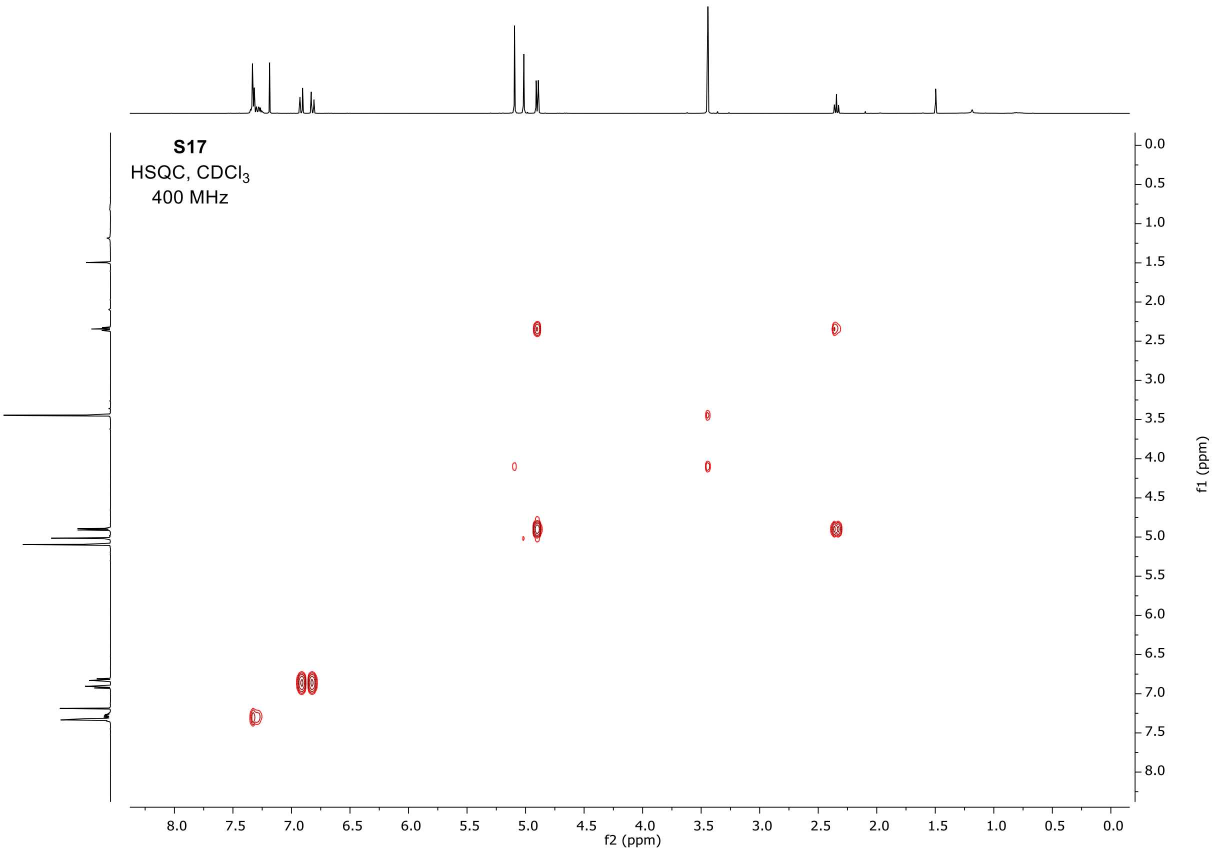
S17
HSQC, CDCl3
400 MHz
f1 (ppm)
f2 (ppm)

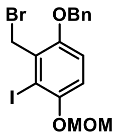

S18

^{1}H NMR, $CDCl_3$

400 MHz

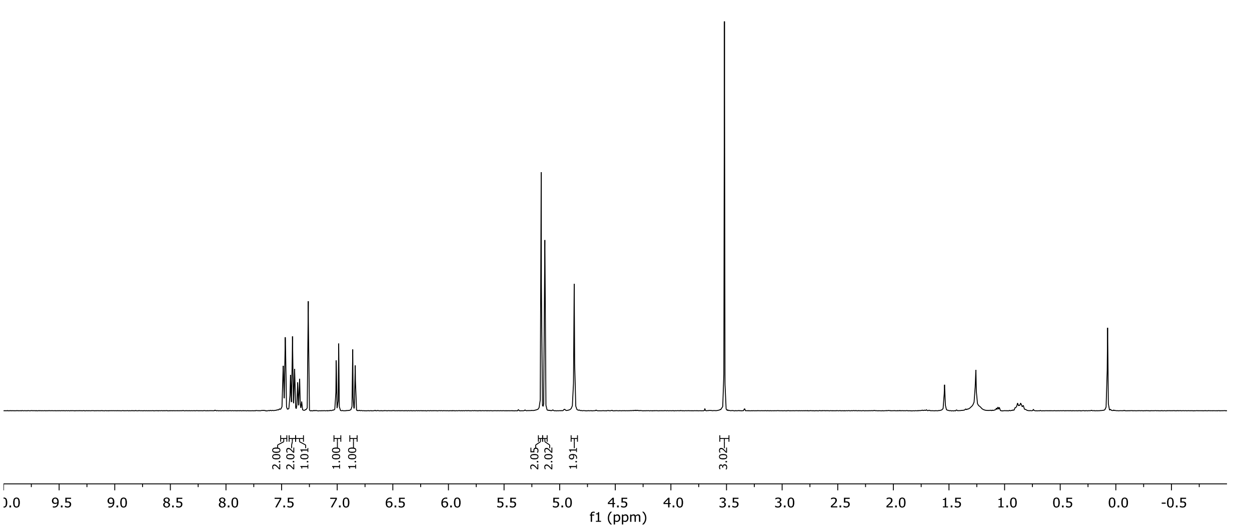

S18

^{13}C NMR, $CDCl_3$

101 MHz

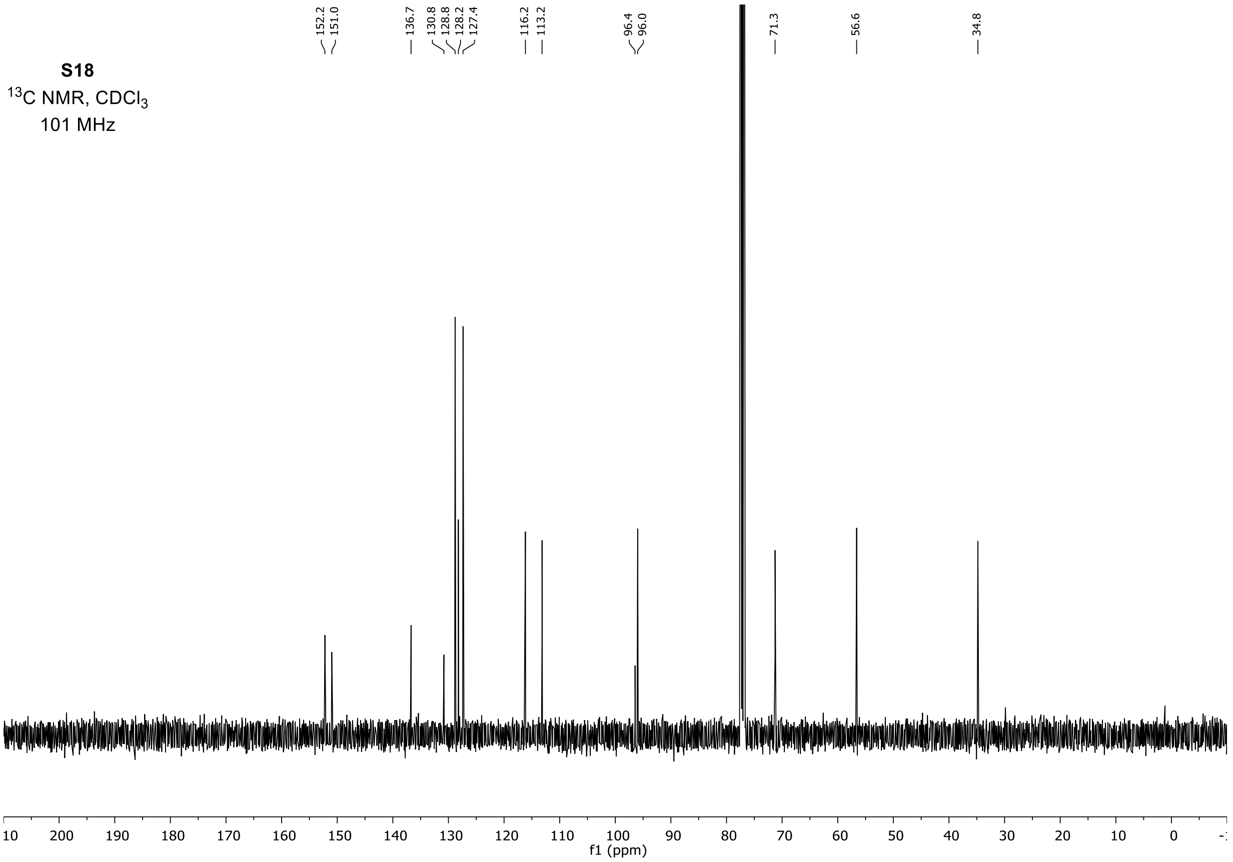

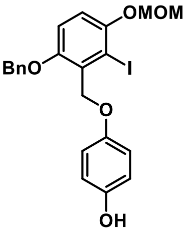

S19
^{1}H NMR, $CDCl_3$
400 MHz

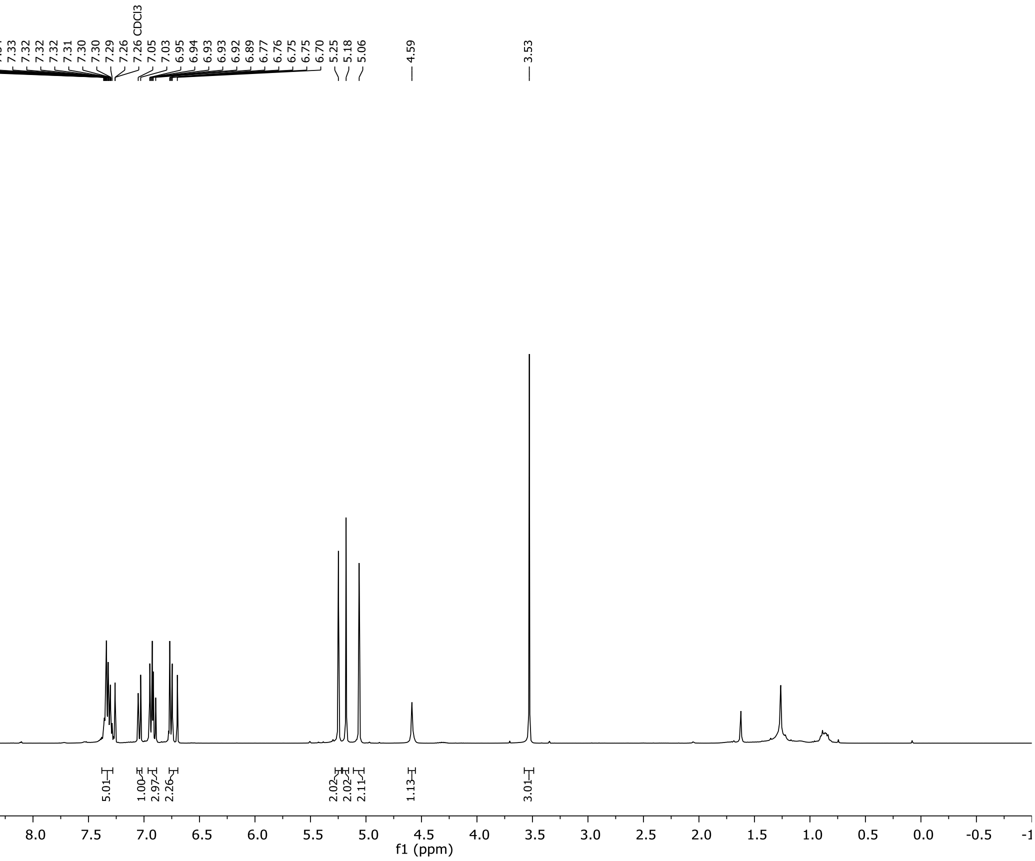

S19
^{13}C NMR, $CDCl_3$
101 MHz

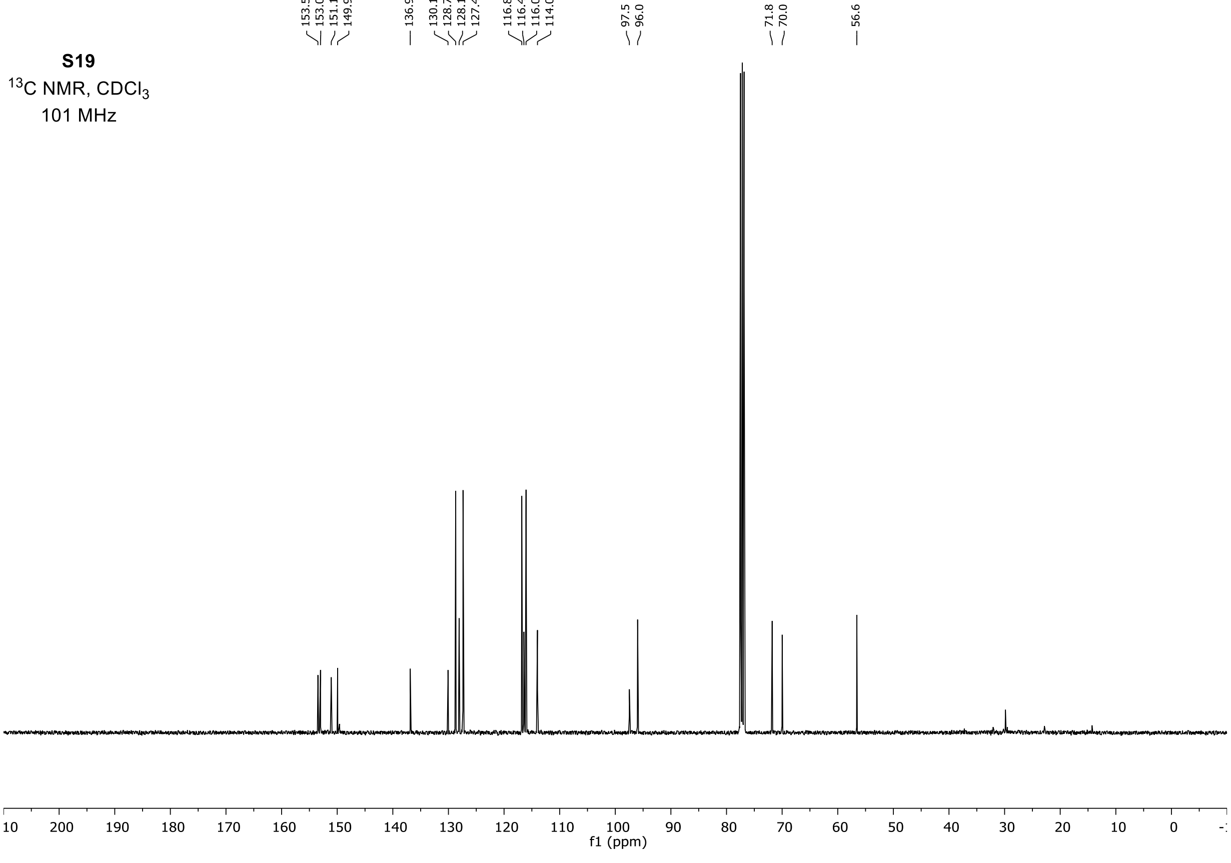

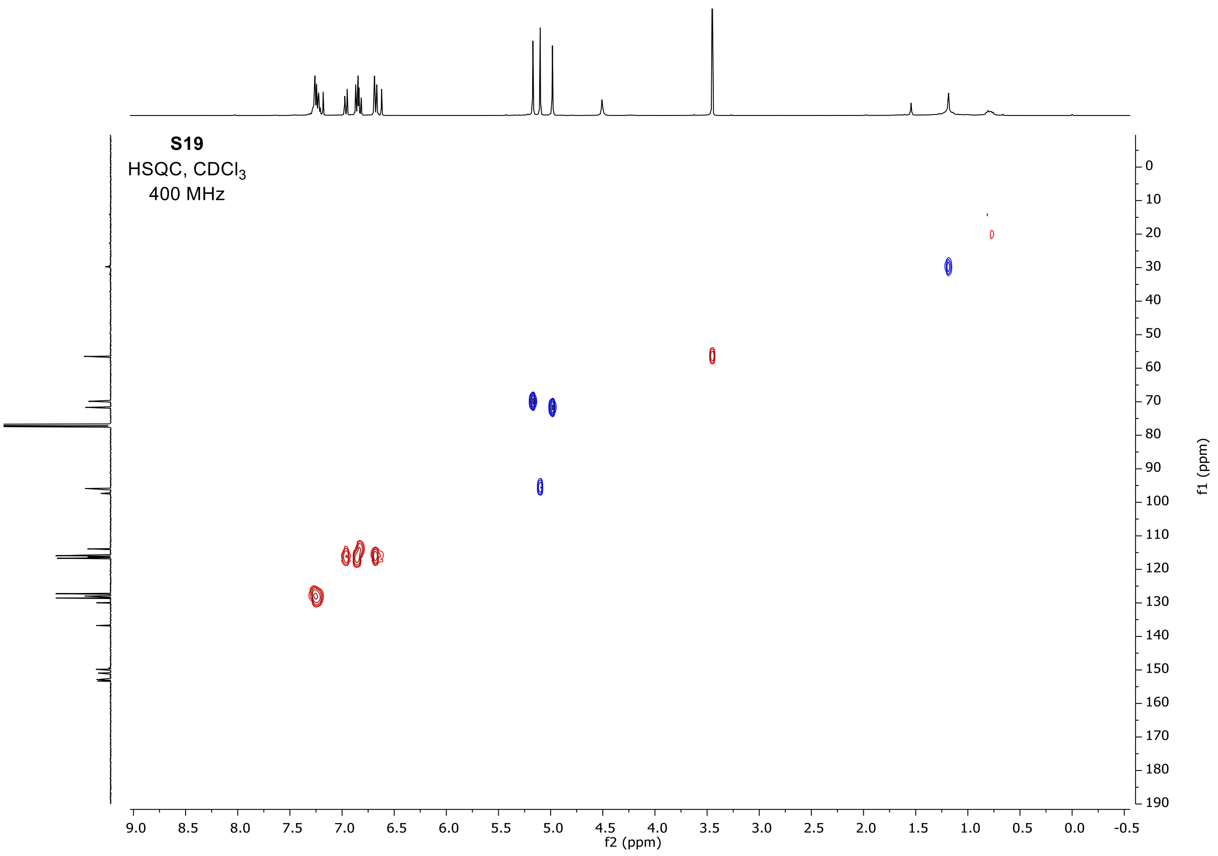
S19
HSQC, CDCl3
400 MHz
f2 (ppm)
f1 (ppm)

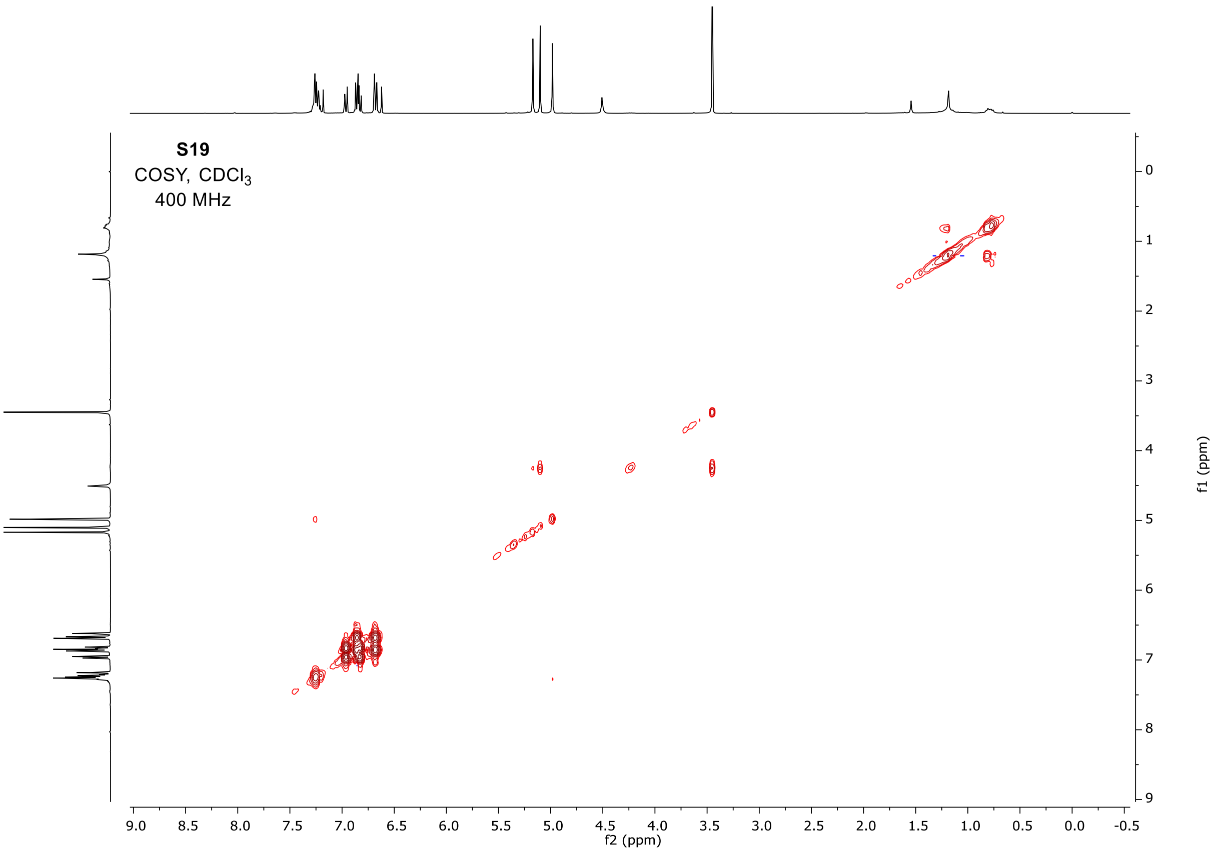
S19
COSY, CDCl3
400 MHz
f2 (ppm)
f1 (ppm)

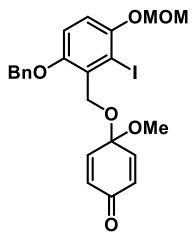

S20
^{1}H NMR, CDC$_3$
400 MHz

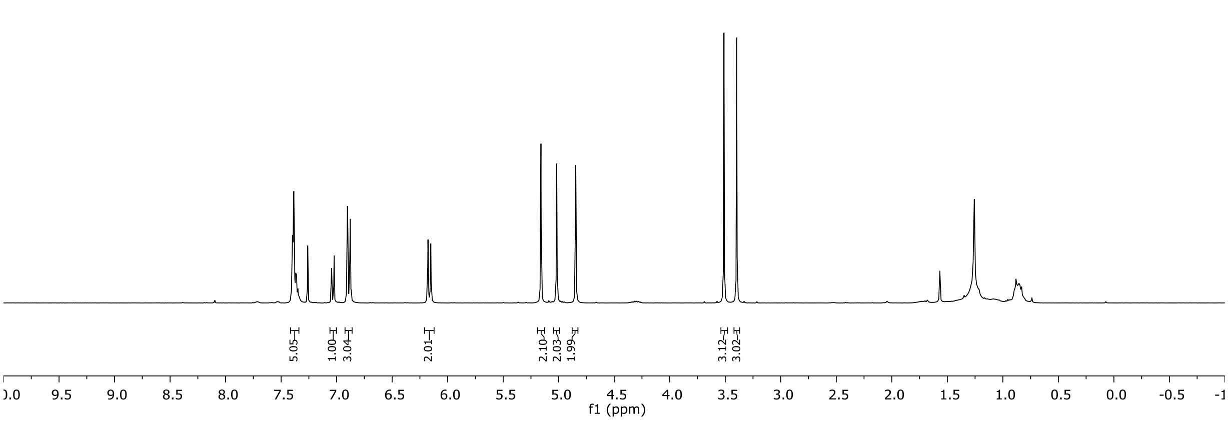

S20
^{13}C NMR, CDC$_3$
101 MHz

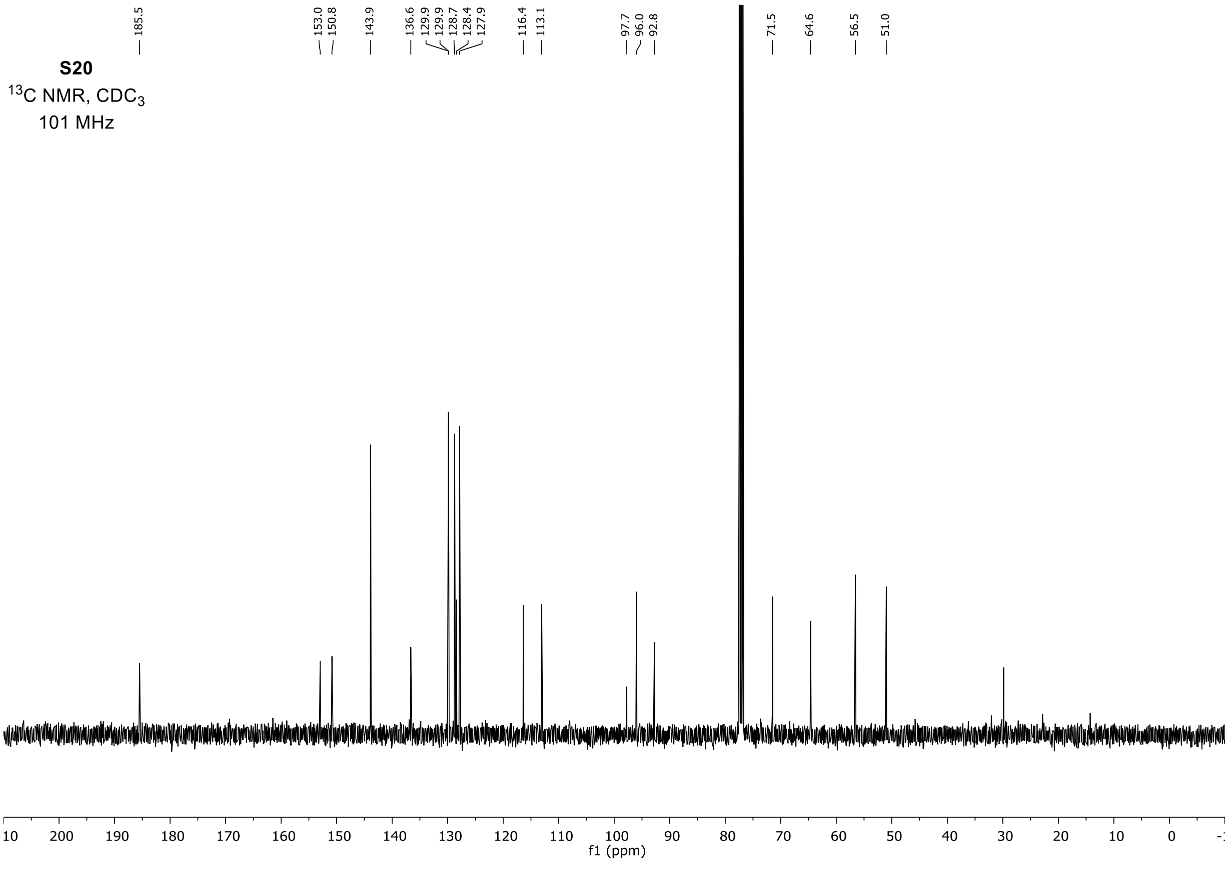

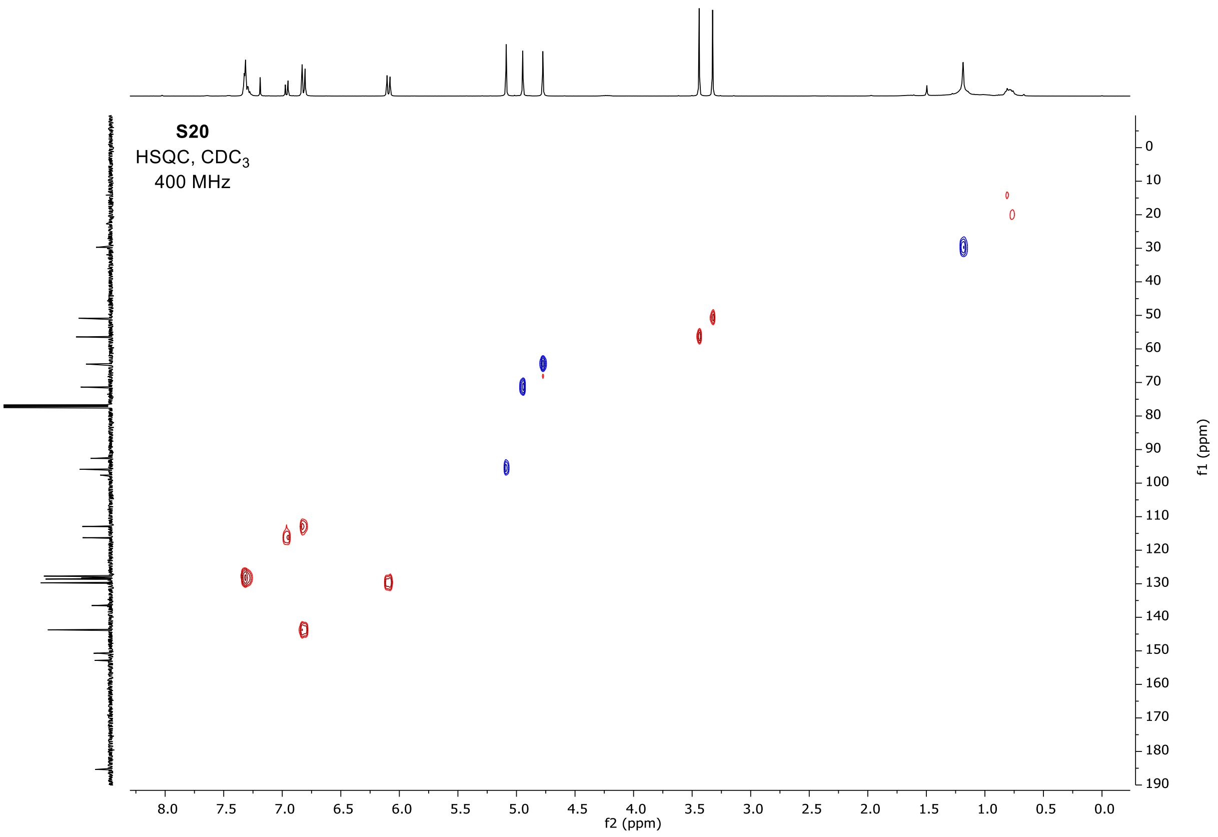
S20
HSQC, CDC3
400 MHz
f2 (ppm)
f1 (ppm)

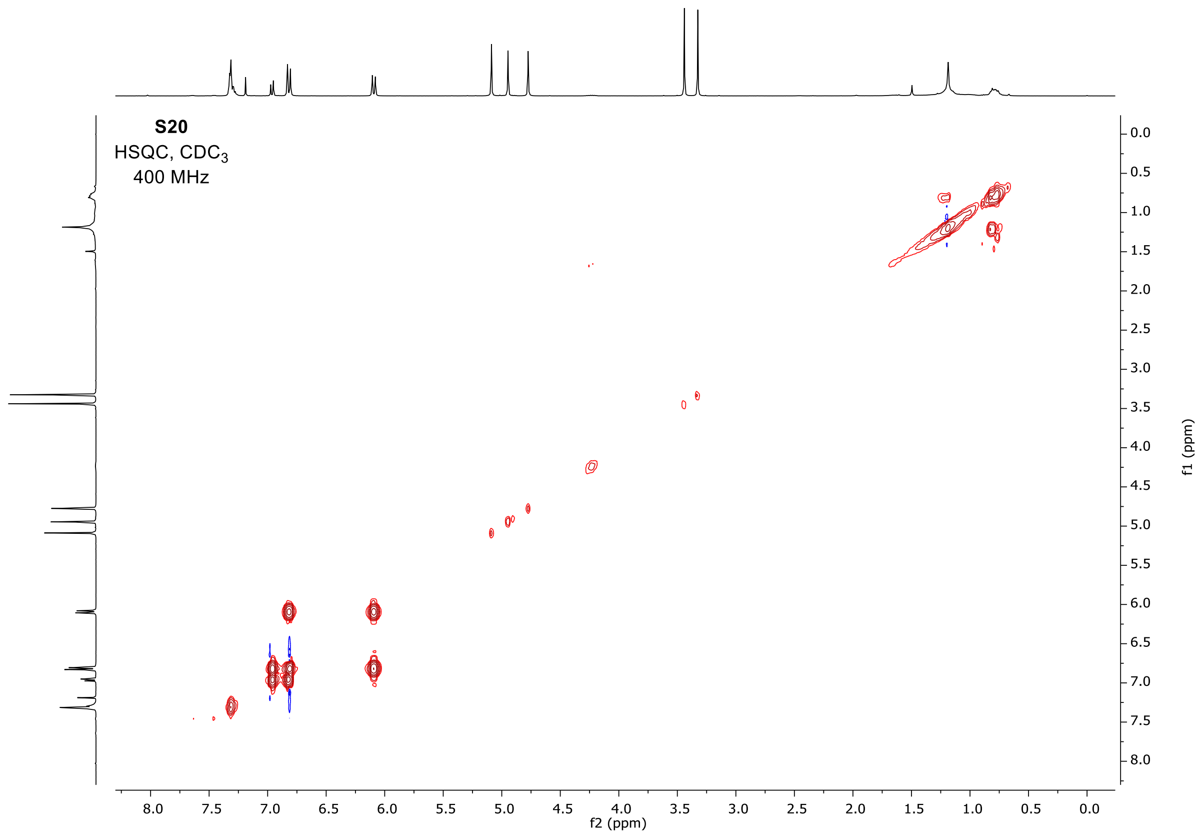
S20
HSQC, CDC3
400 MHz
f2 (ppm)
f1 (ppm)

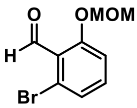

S24
^{1}H NMR, $CDCl_3$
400 MHz

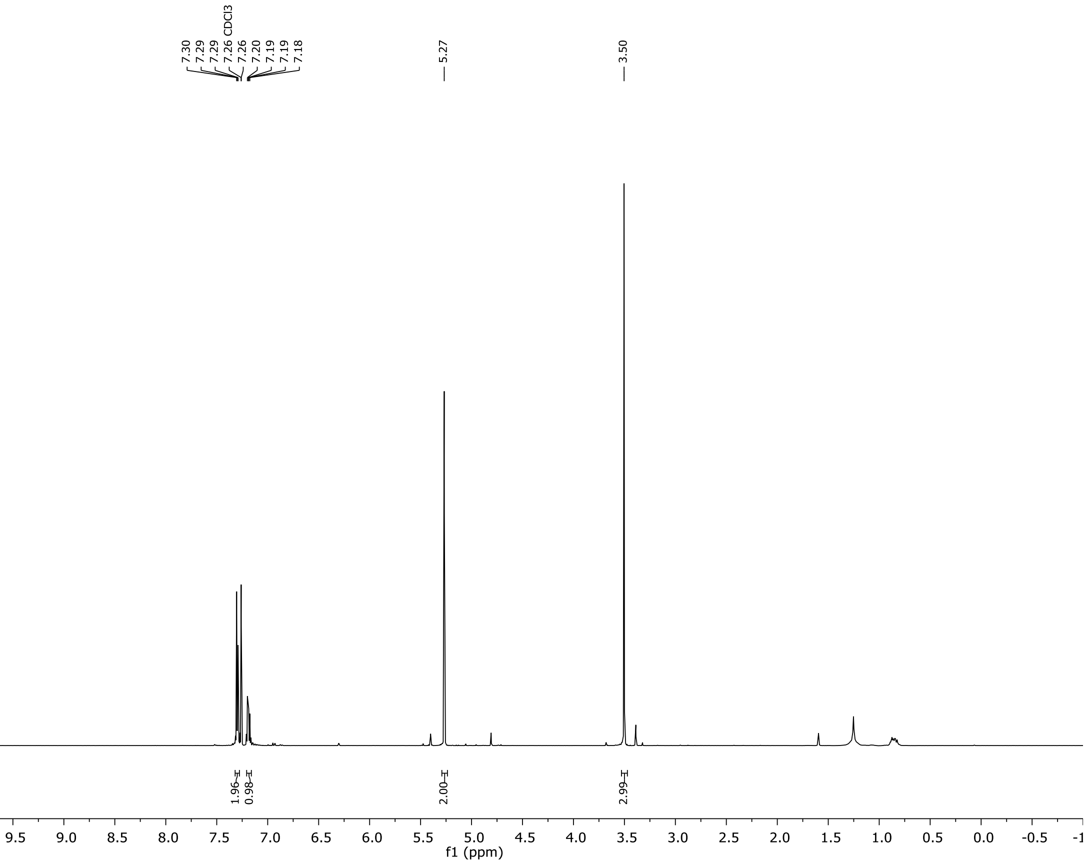

S24
^{13}C NMR, $CDCl_3$
101 MHz

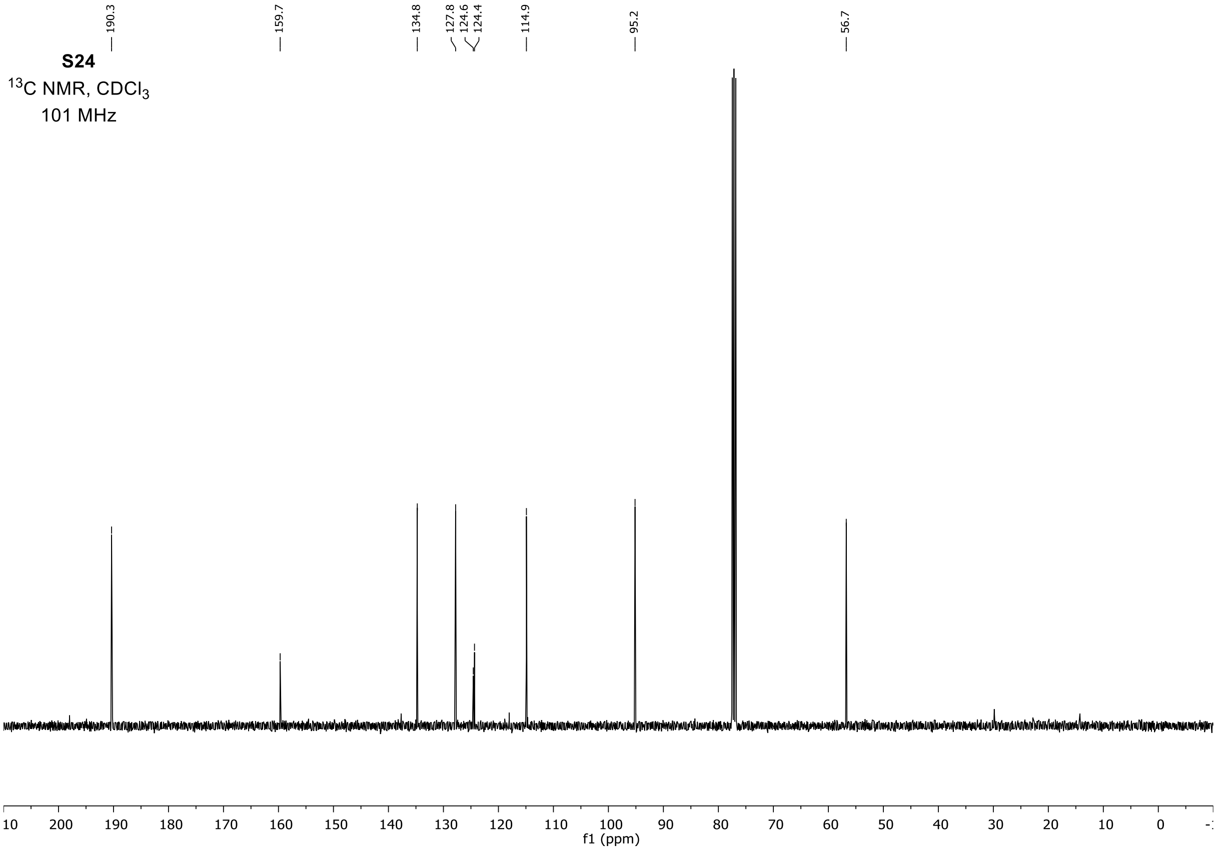

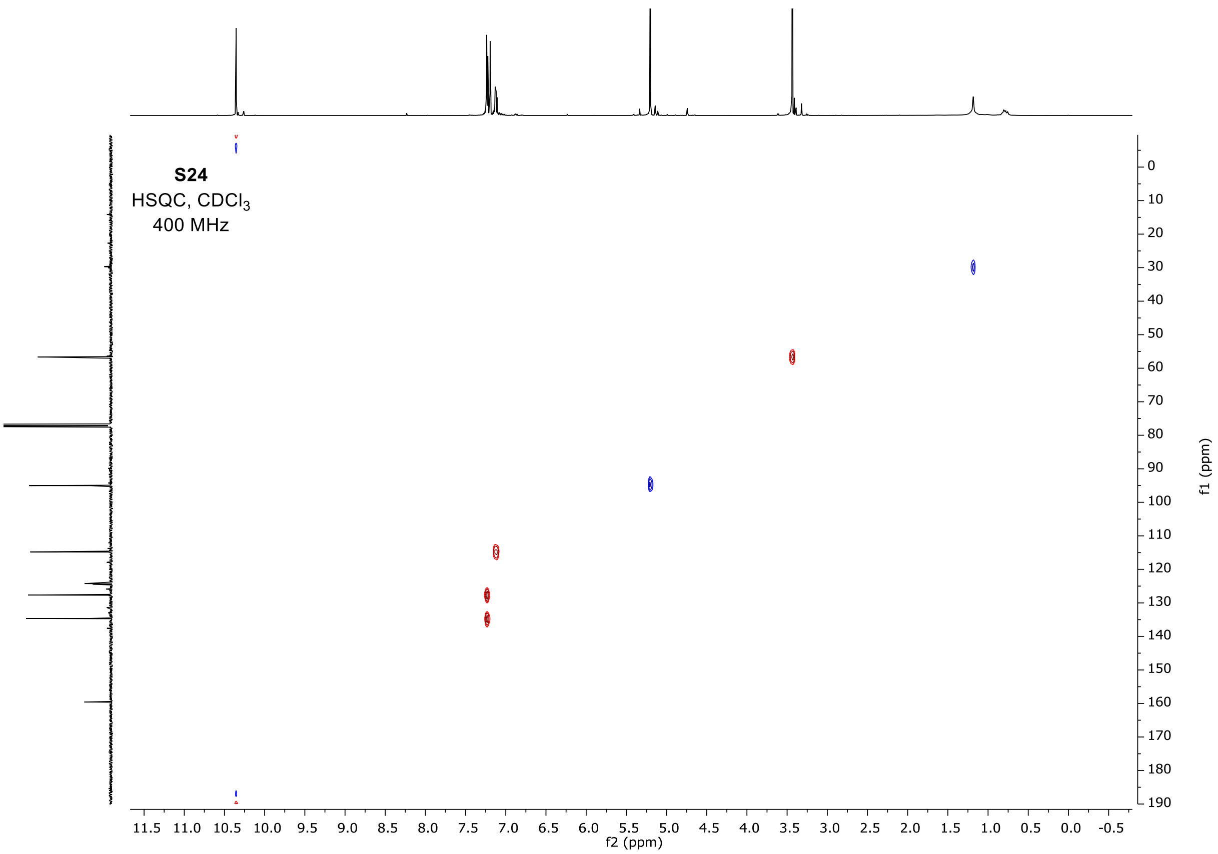
S24
HSQC, CDCl3
400 MHz
f2 (ppm)
f1 (ppm)

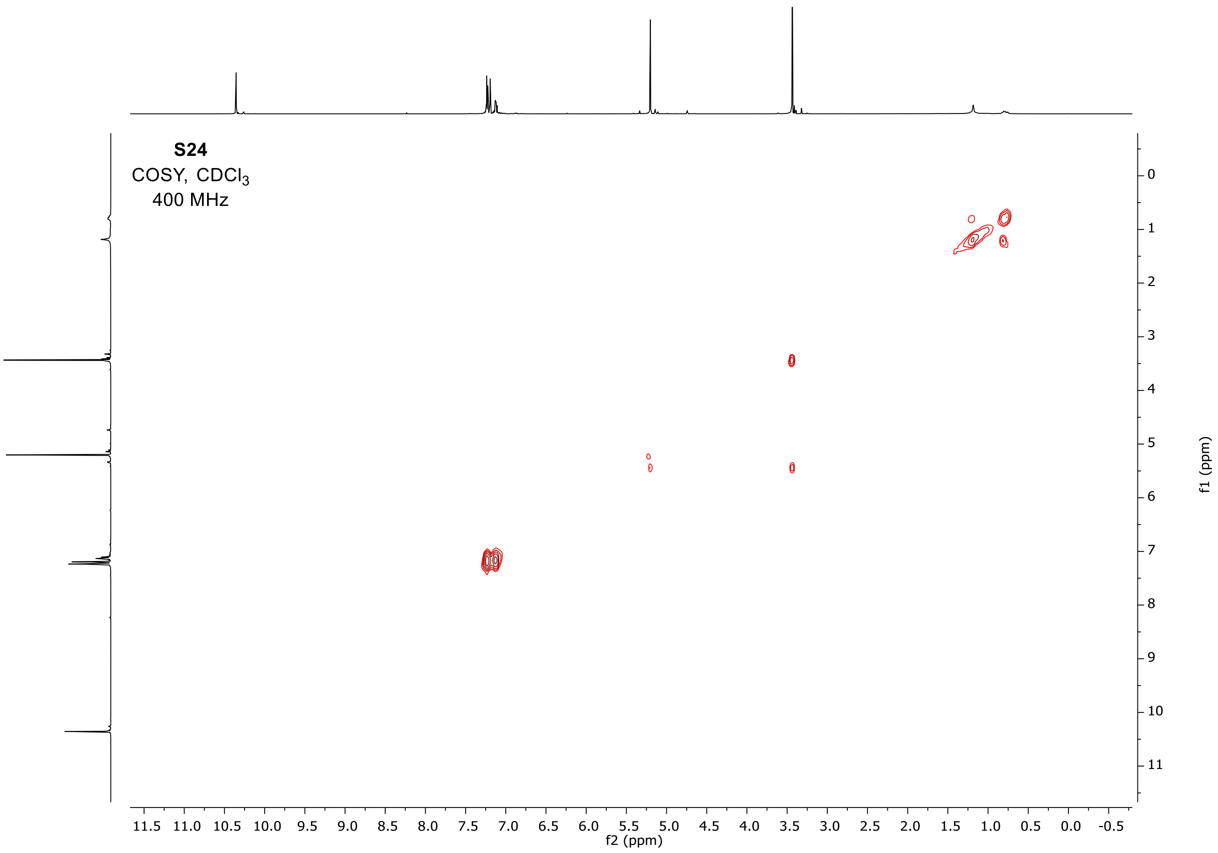
S24
COSY, CDCl3
400 MHz
f2 (ppm)
f1 (ppm)

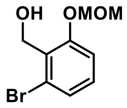

S25
^{1}H NMR, $CDCl_3$
400 MHz

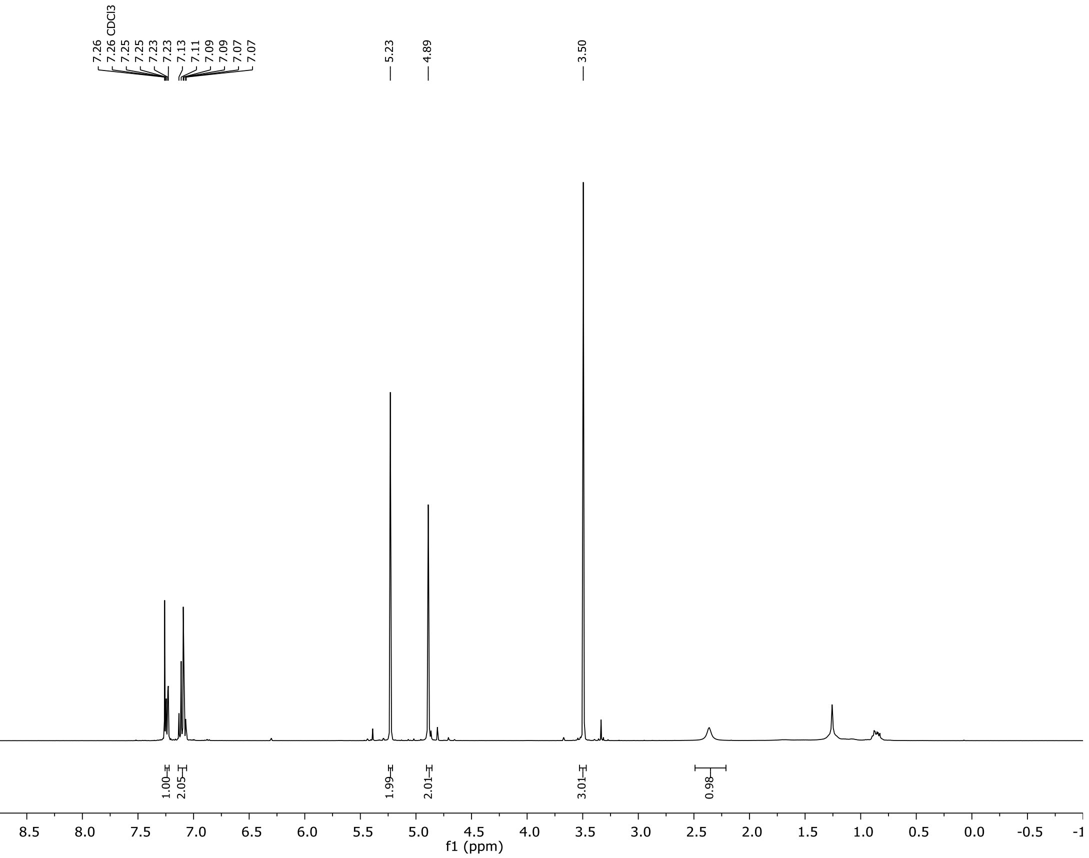

S25
^{13}C NMR, $CDCl_3$
101 MHz

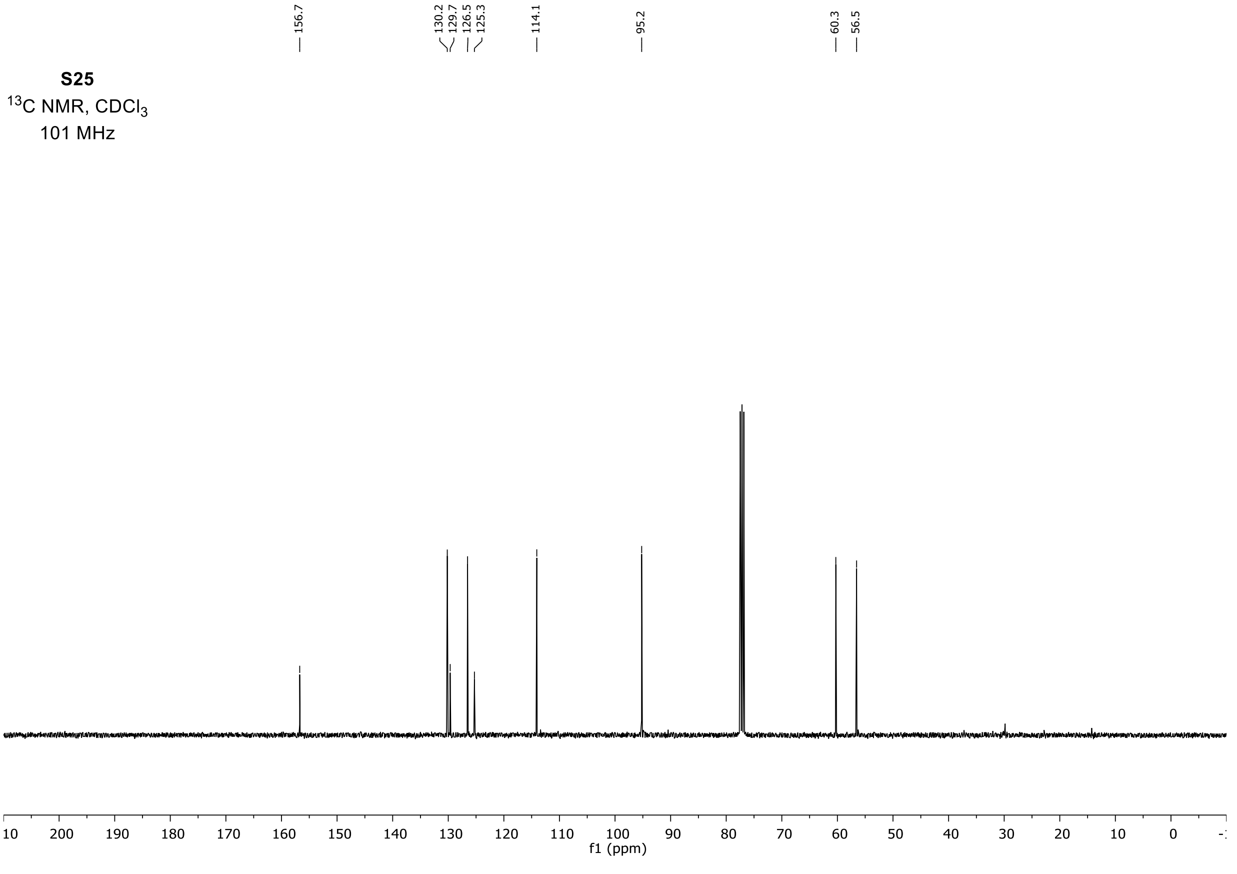

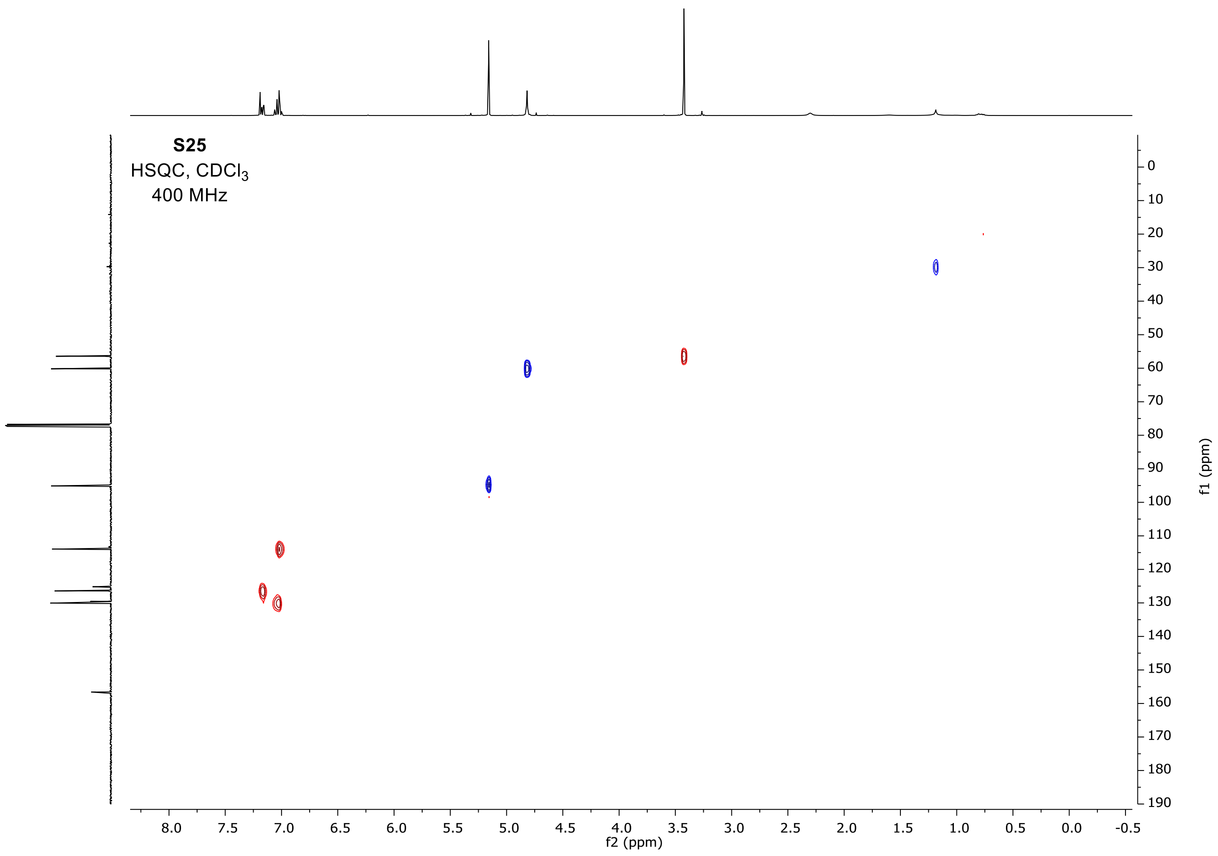
S25
HSQC, CDCl3
400 MHz
f2 (ppm)
f1 (ppm)

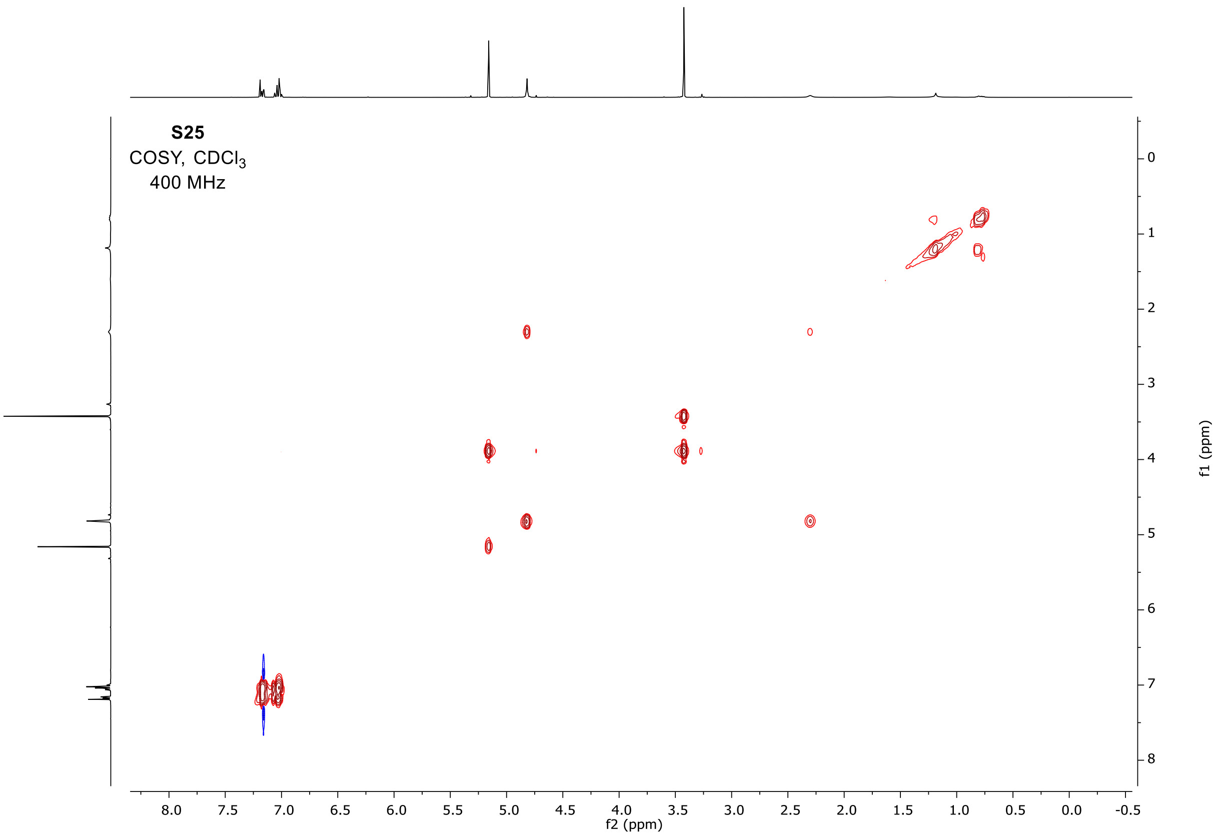
S25
COSY, CDCl3
400 MHz
f2 (ppm)
f1 (ppm)

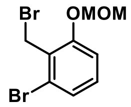

S26
^{1}H NMR, $CDCl_3$
400 MHz

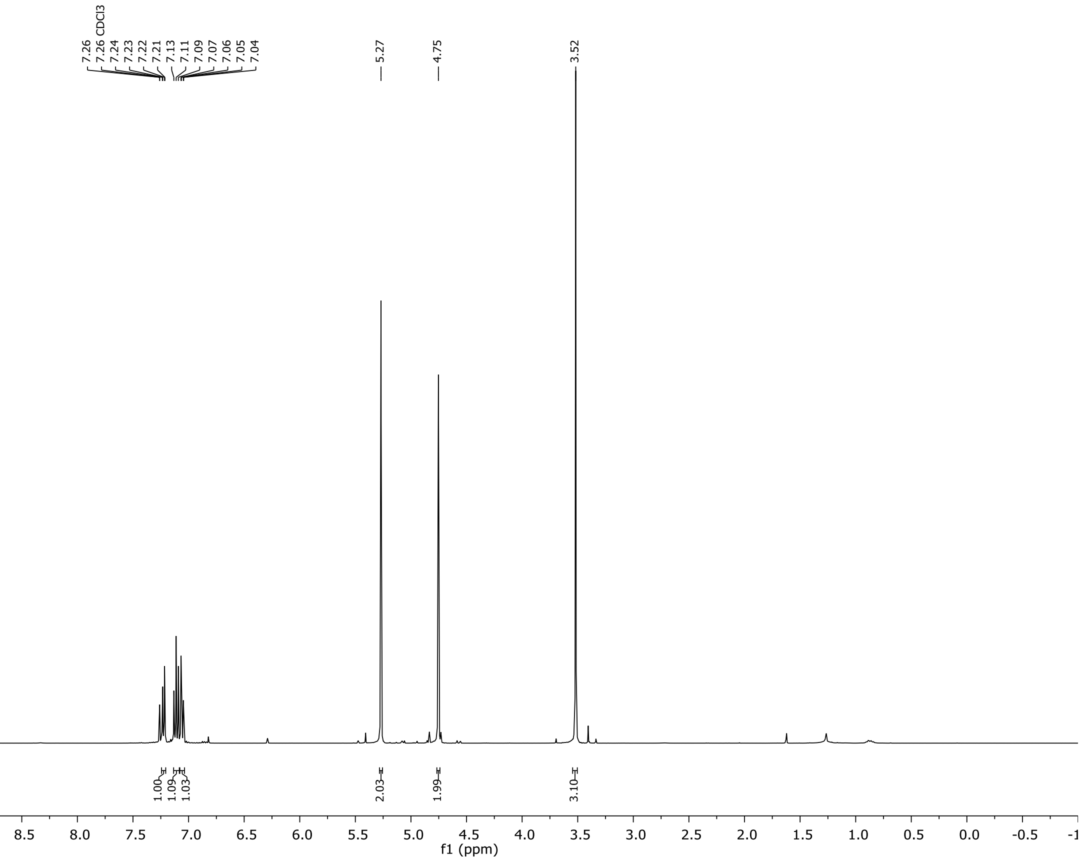

S26
^{13}C NMR, $CDCl_3$
101 MHz

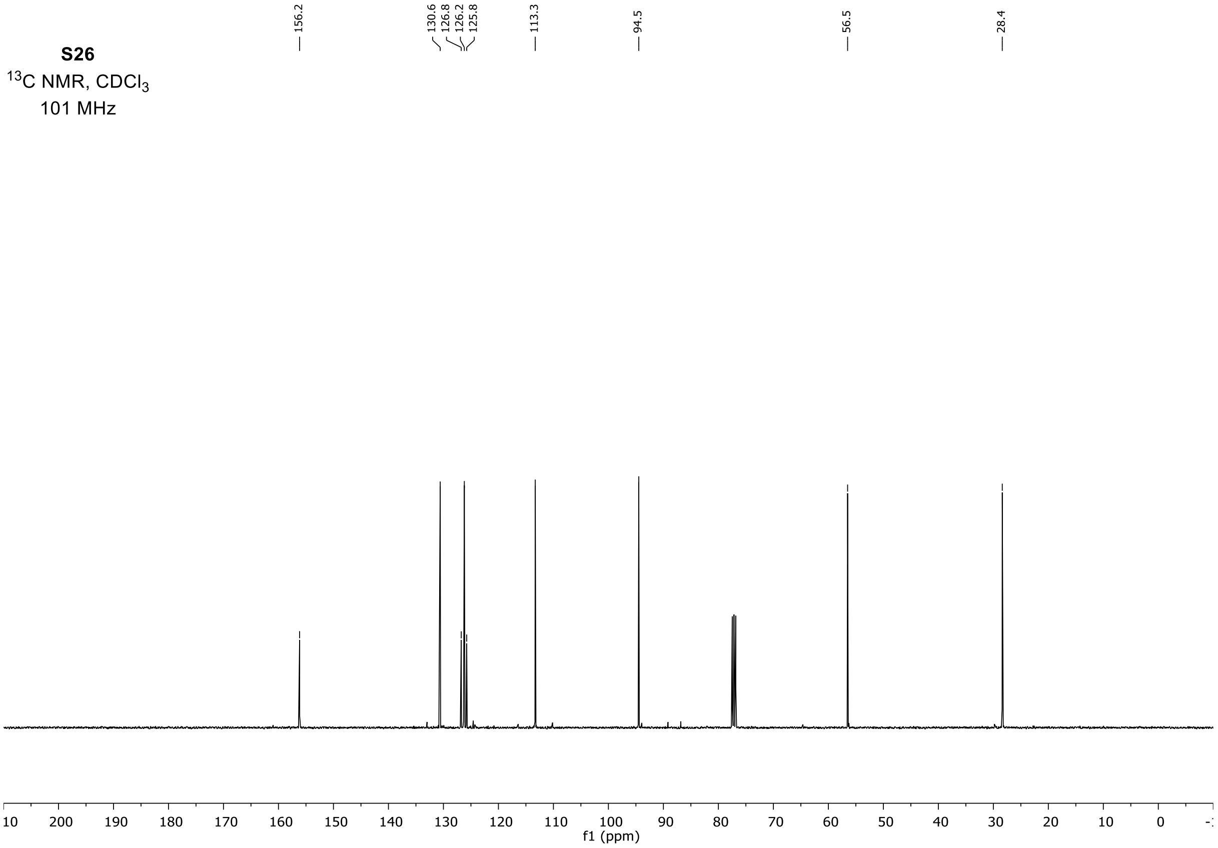

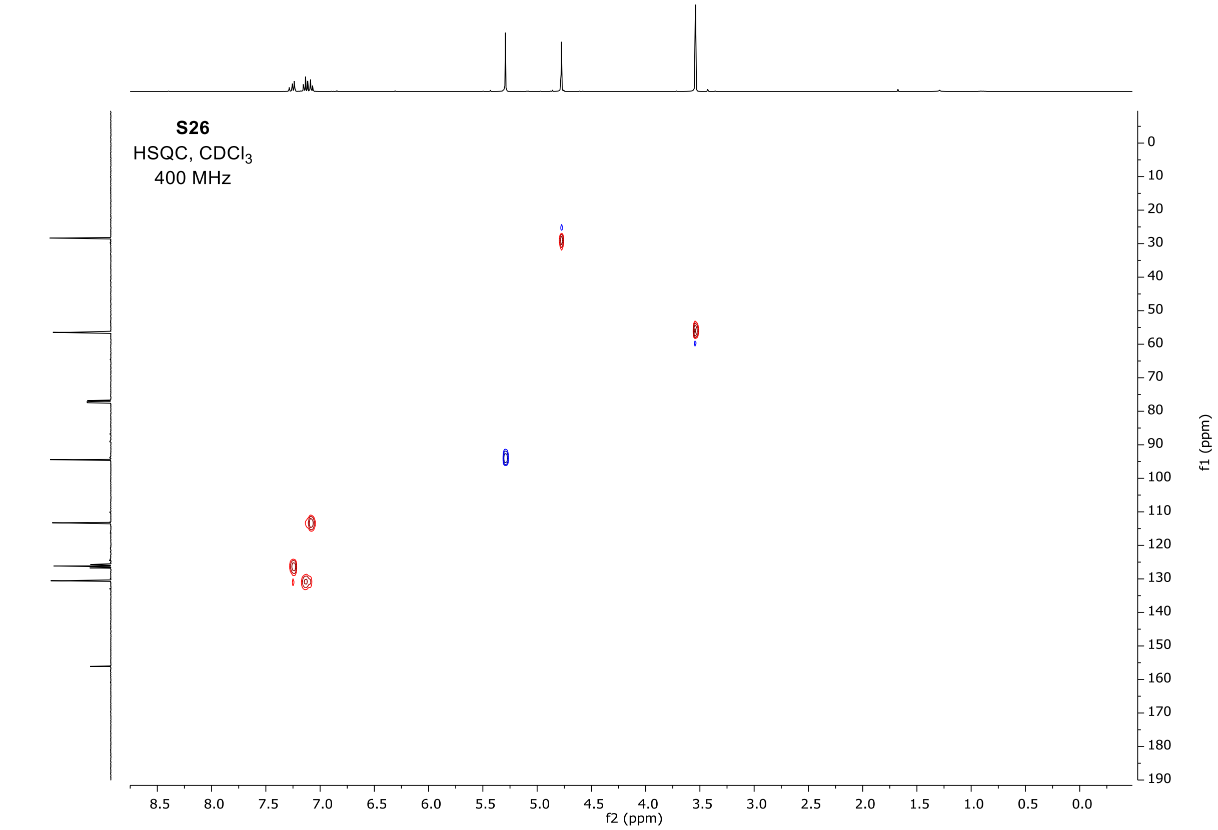
S26
HSQC, CDCl3
400 MHz
f2 (ppm)
f1 (ppm)

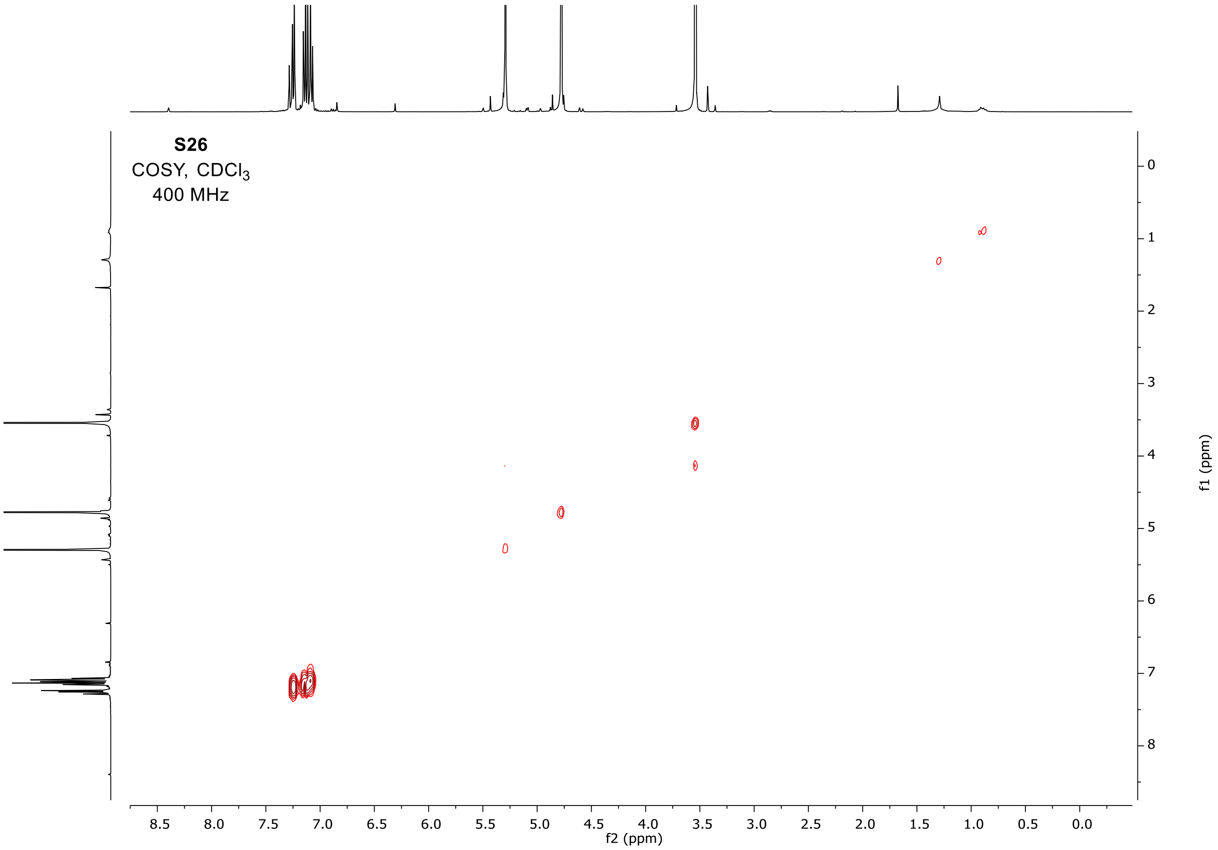
S26
COSY, CDCl3
400 MHz
f2 (ppm)
f1 (ppm)

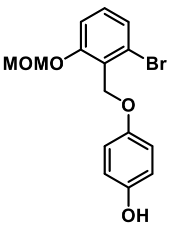

S27
^{1}H NMR, $CDCl_3$
400 MHz

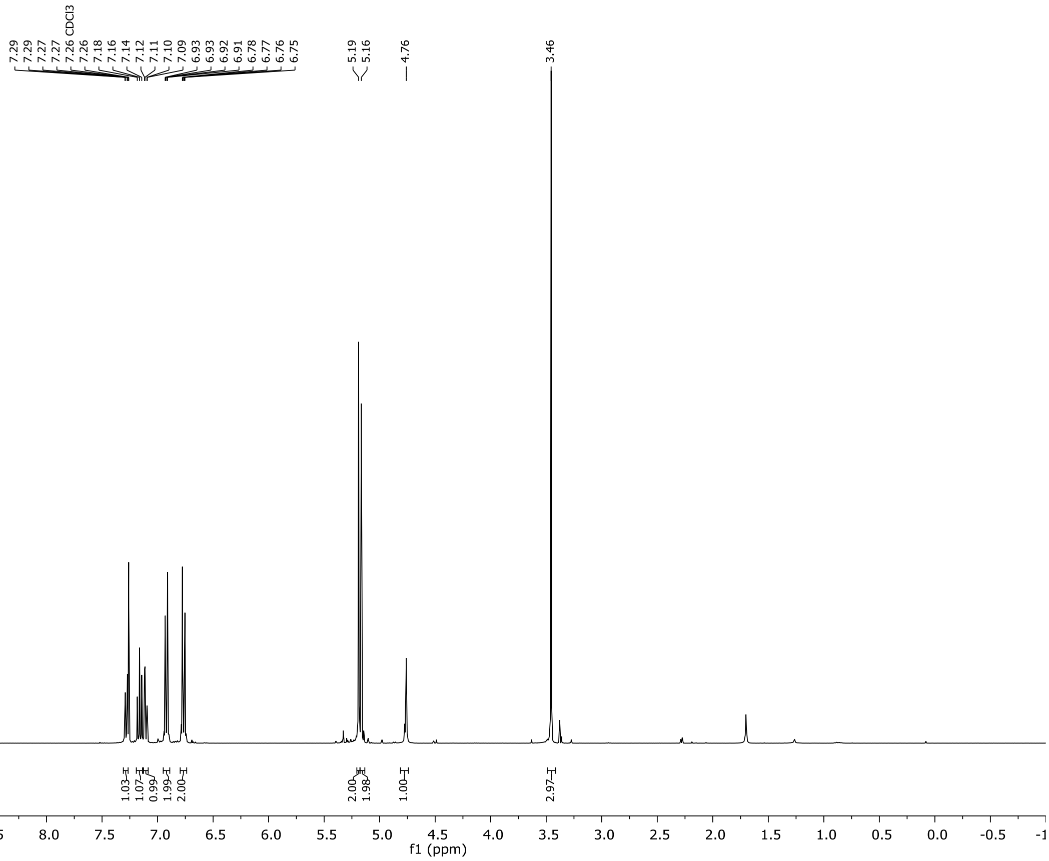

S27
^{13}C NMR, $CDCl_3$
101 MHz

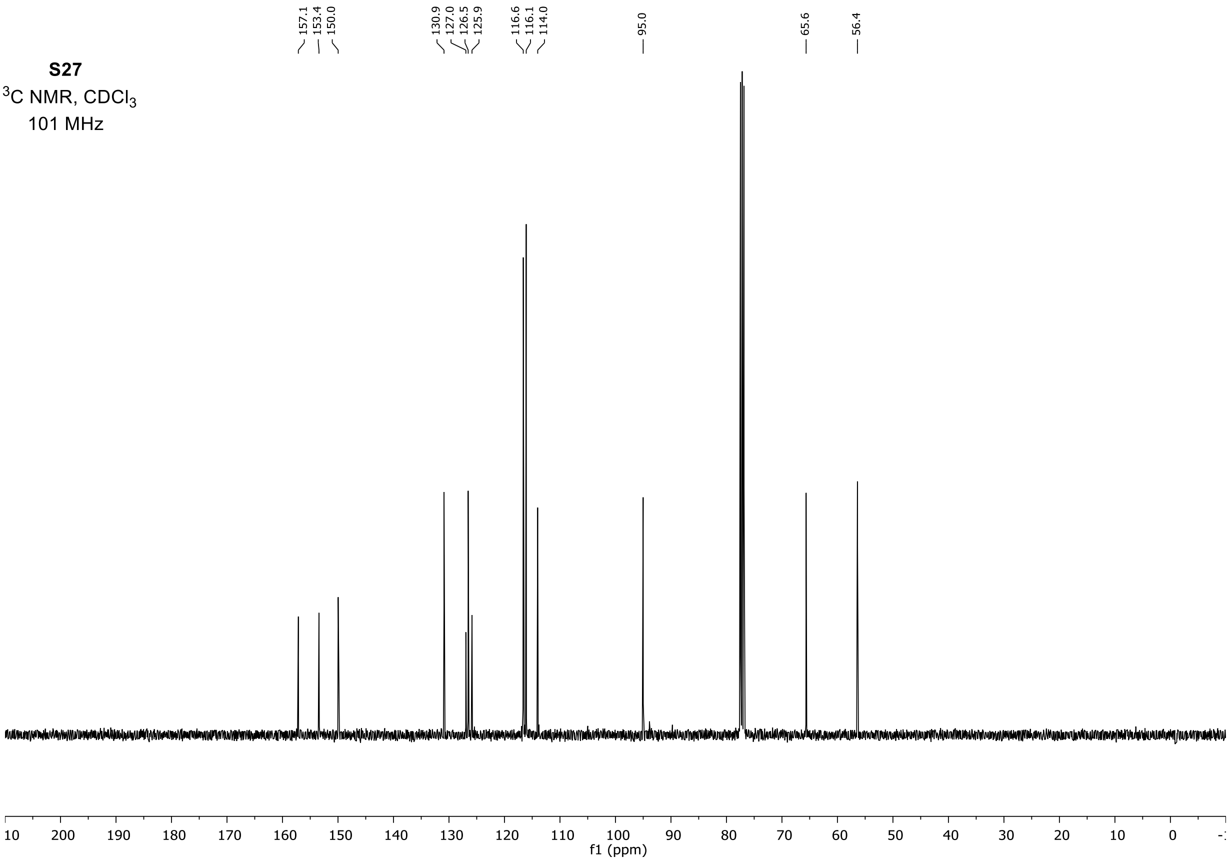

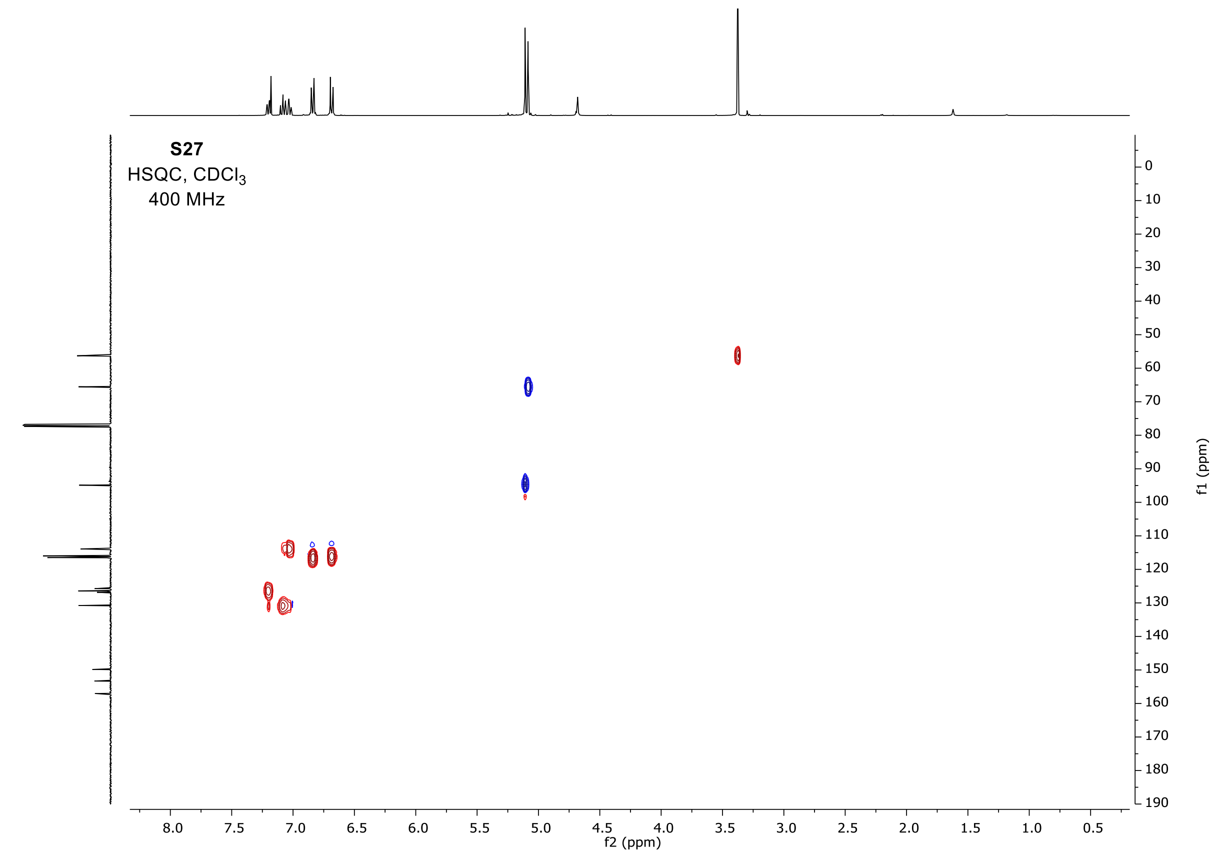
S27
HSQC, $CDCl_3$
400 MHz
f2 (ppm)
f1 (ppm)

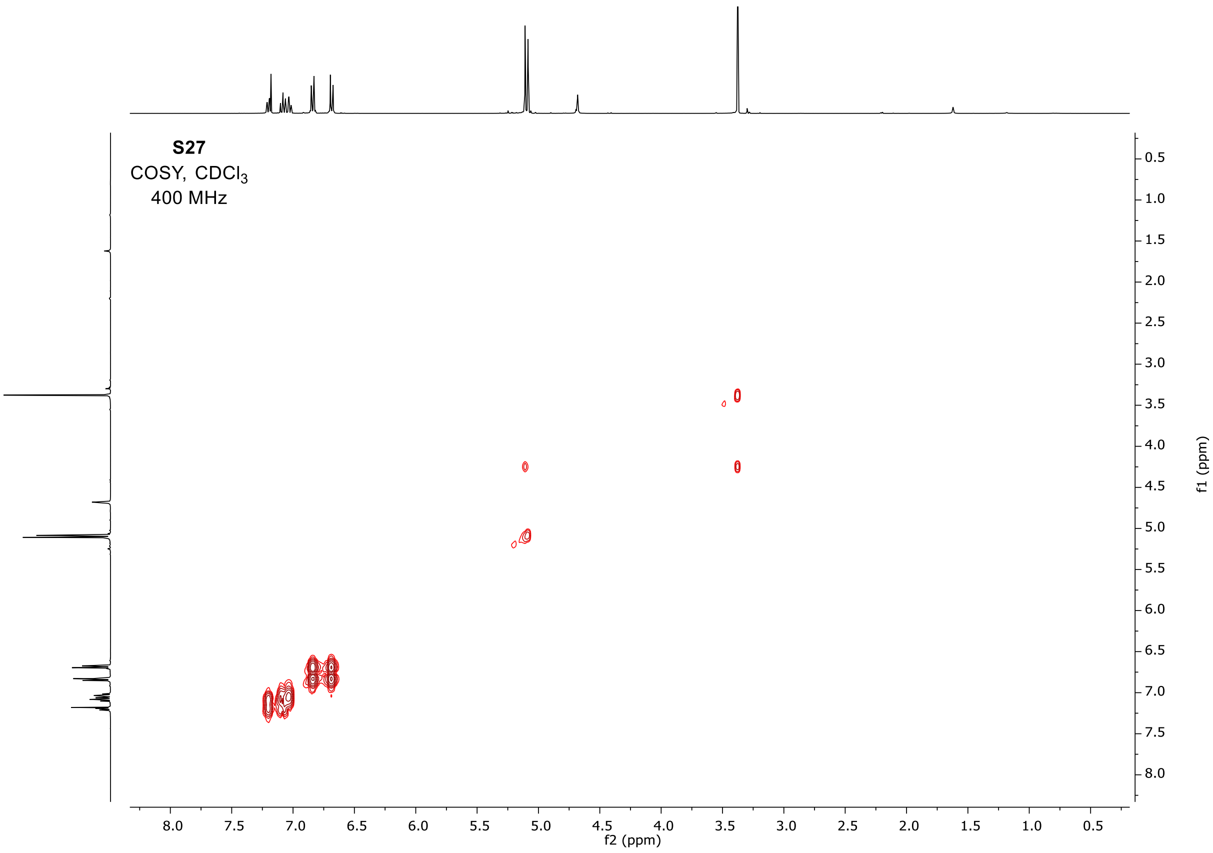
S27
COSY, $CDCl_3$
400 MHz
f2 (ppm)
f1 (ppm)

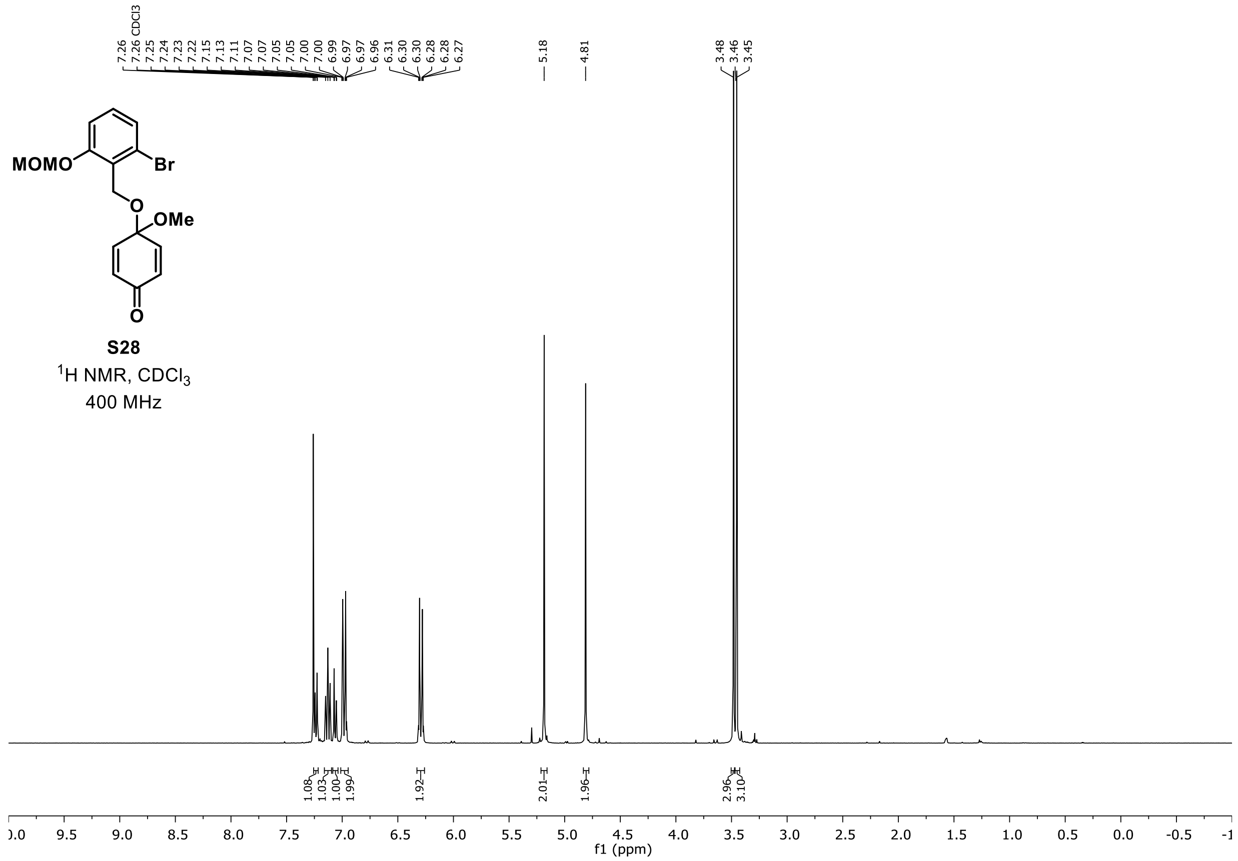
MOMO
Br
O
OMe
O
S28
1H NMR, CDCl3
400 MHz
7.26 CDCl3
5.18
4.81
3.48
3.46
3.45
f1 (ppm)

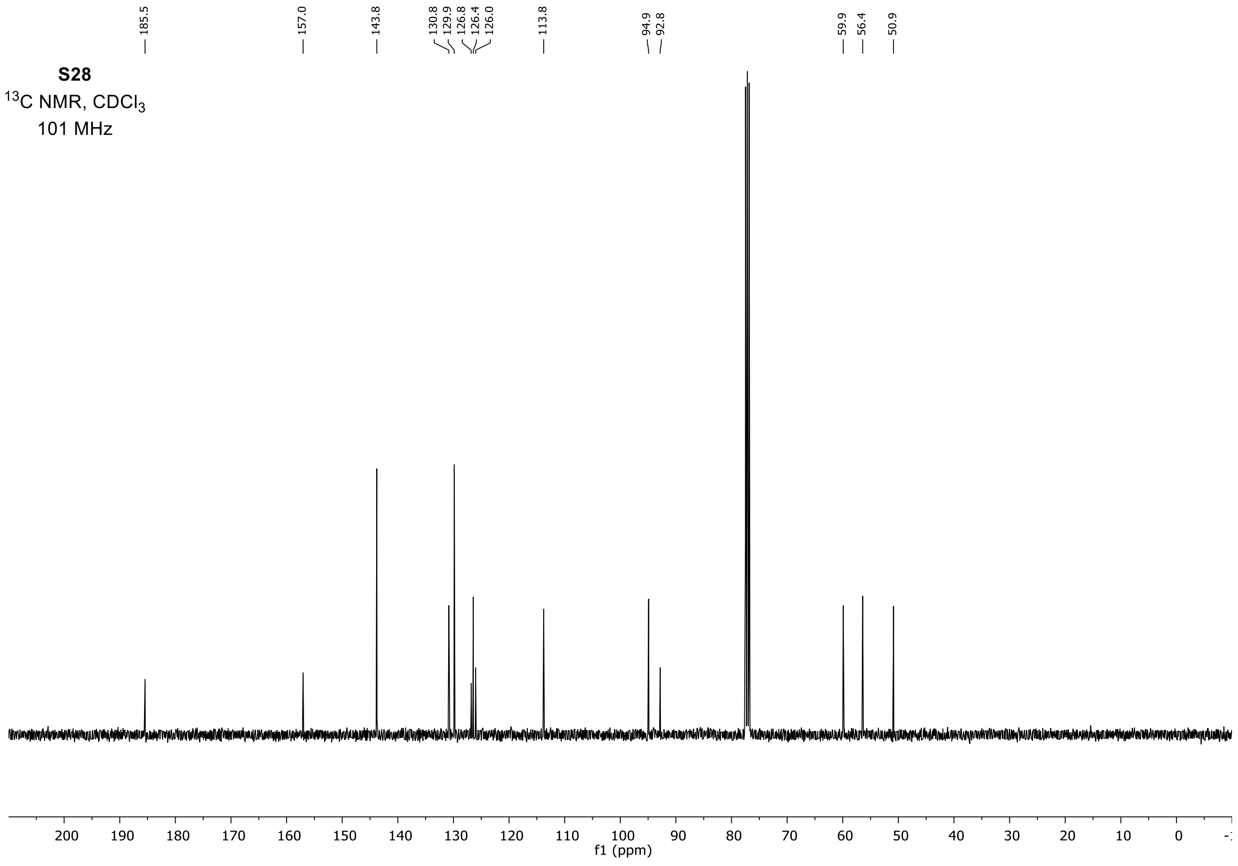
S28
13C NMR, CDCl3
101 MHz
185.5
157.0
143.8
130.8
129.9
126.8
126.4
126.0
113.8
94.9
92.8
59.9
56.4
50.9
f1 (ppm)

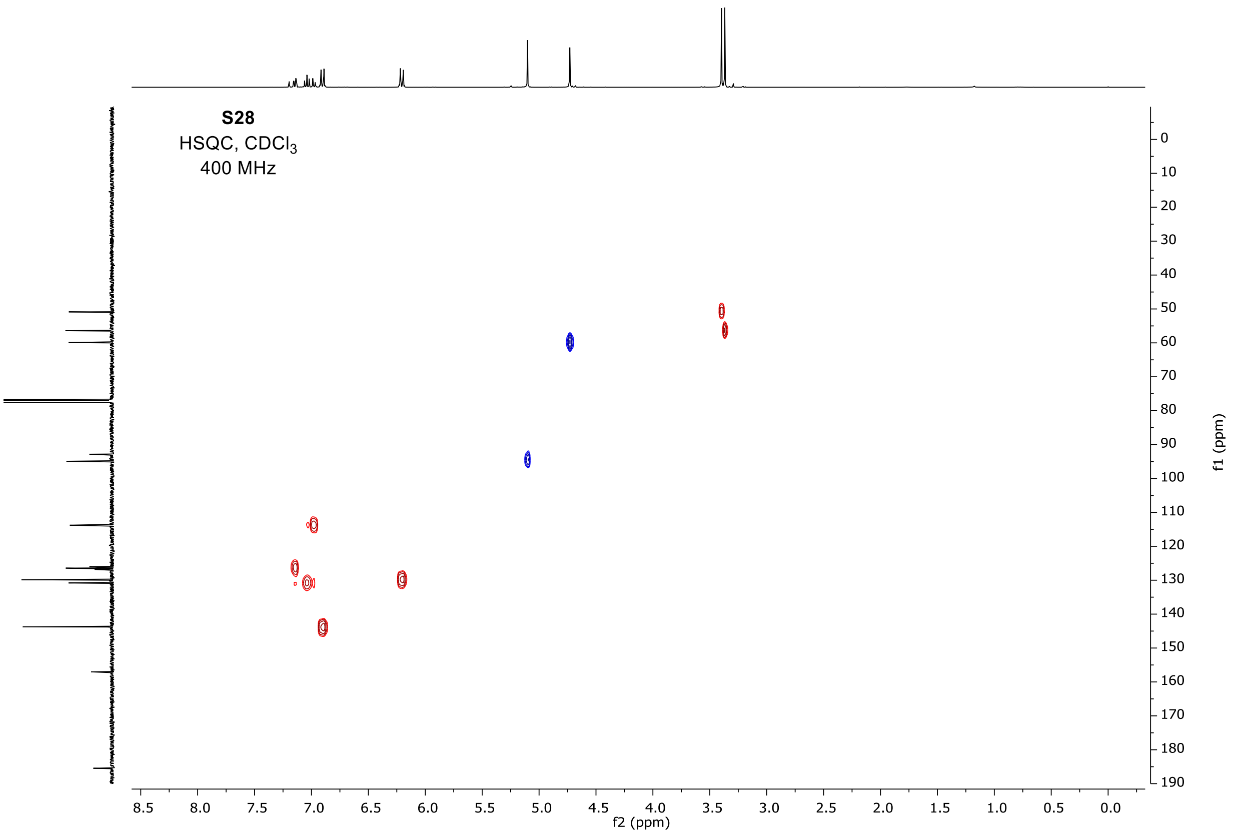
S28
HSQC, CDCl3
400 MHz
f2 (ppm)
f1 (ppm)

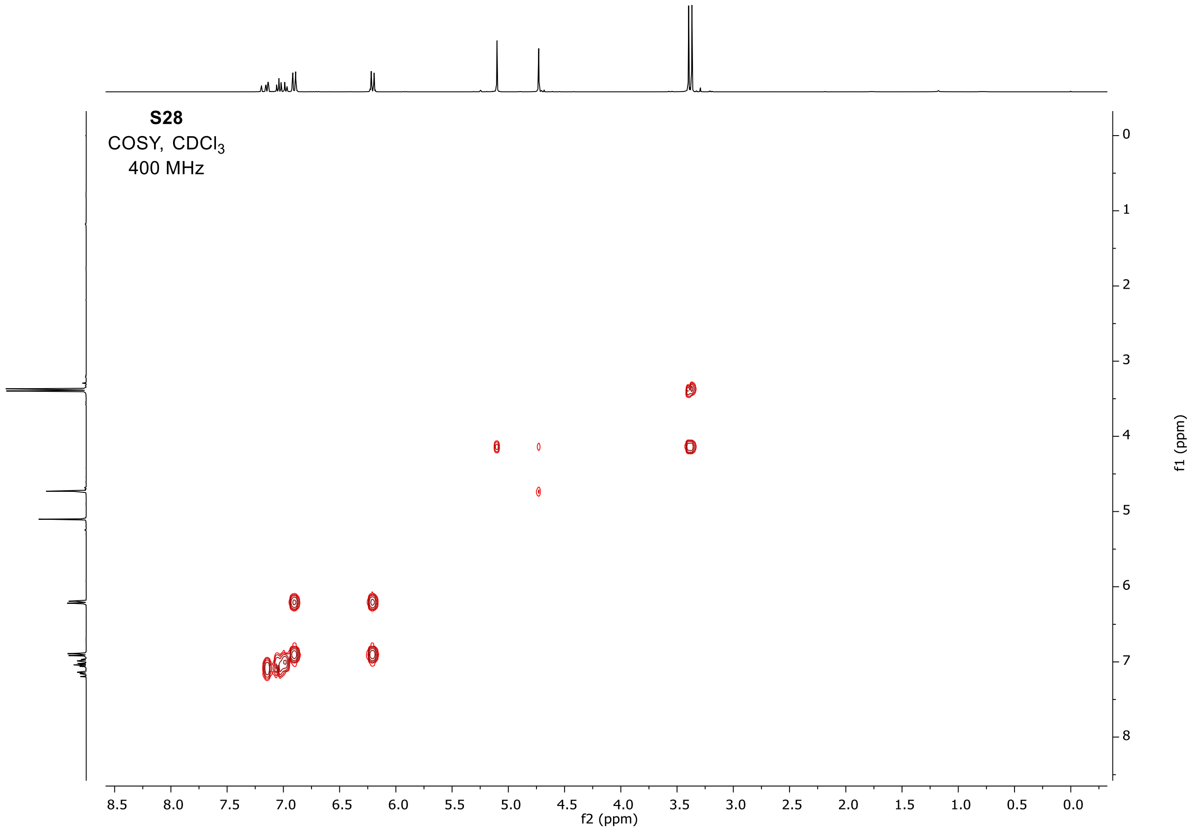
S28
COSY, CDCl3
400 MHz
f2 (ppm)
f1 (ppm)

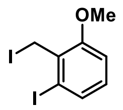

S33
^{1}H NMR, $CDCl_3$
400 MHz

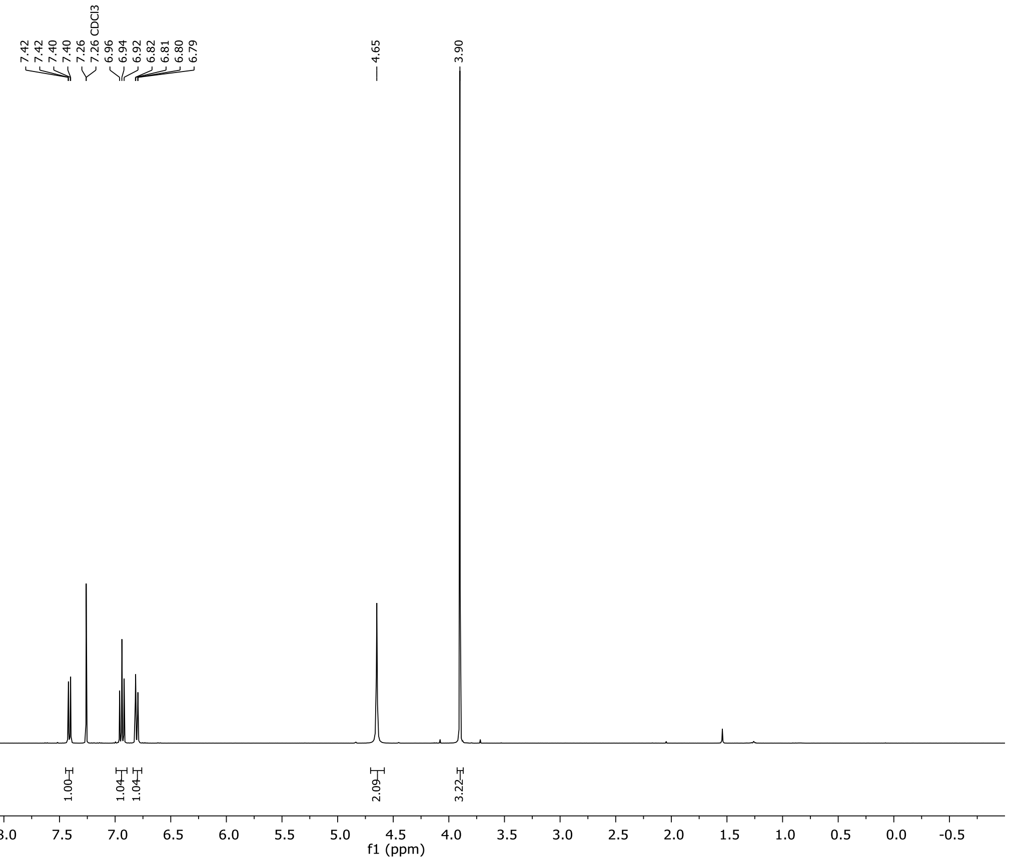

S33
^{13}C NMR, $CDCl_3$
101 MHz

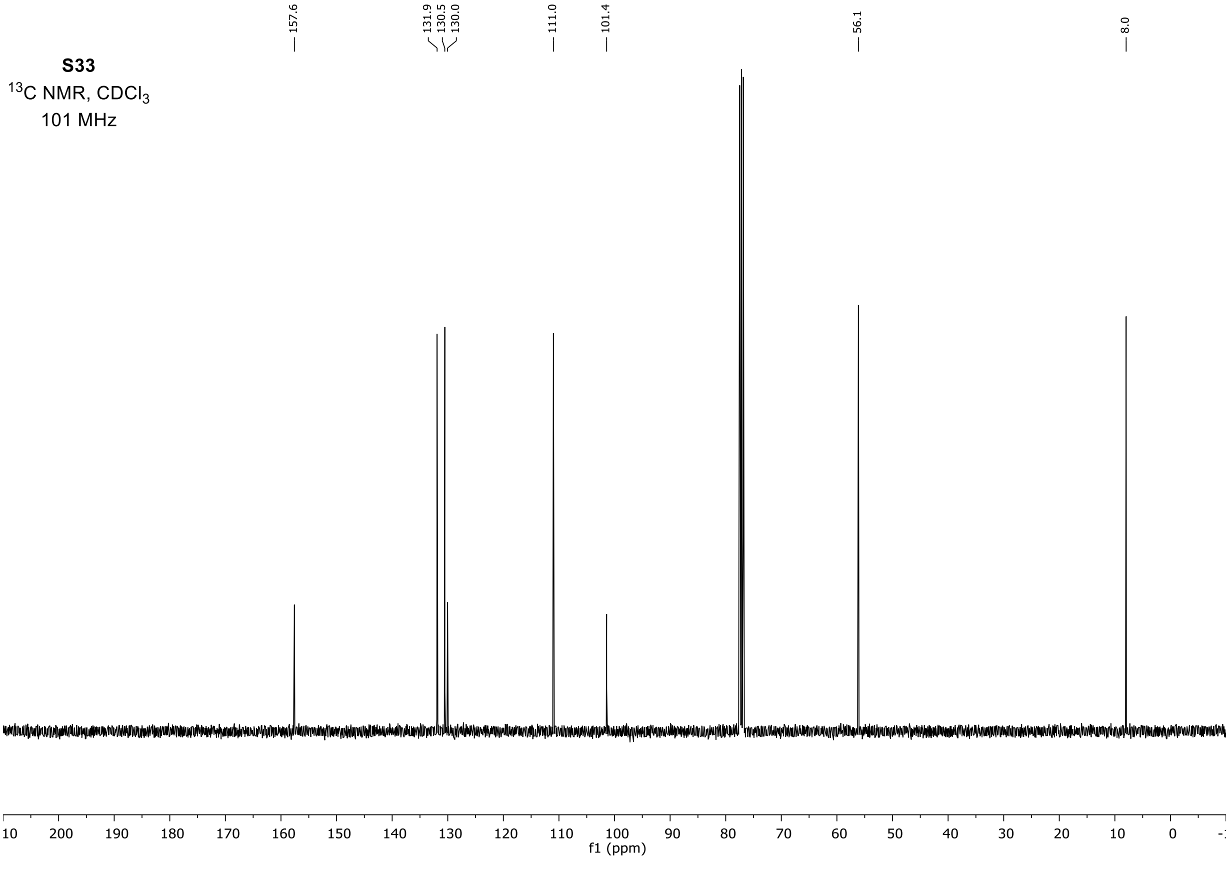

S34
^{1}H NMR, $CDCl_3$
400 MHz

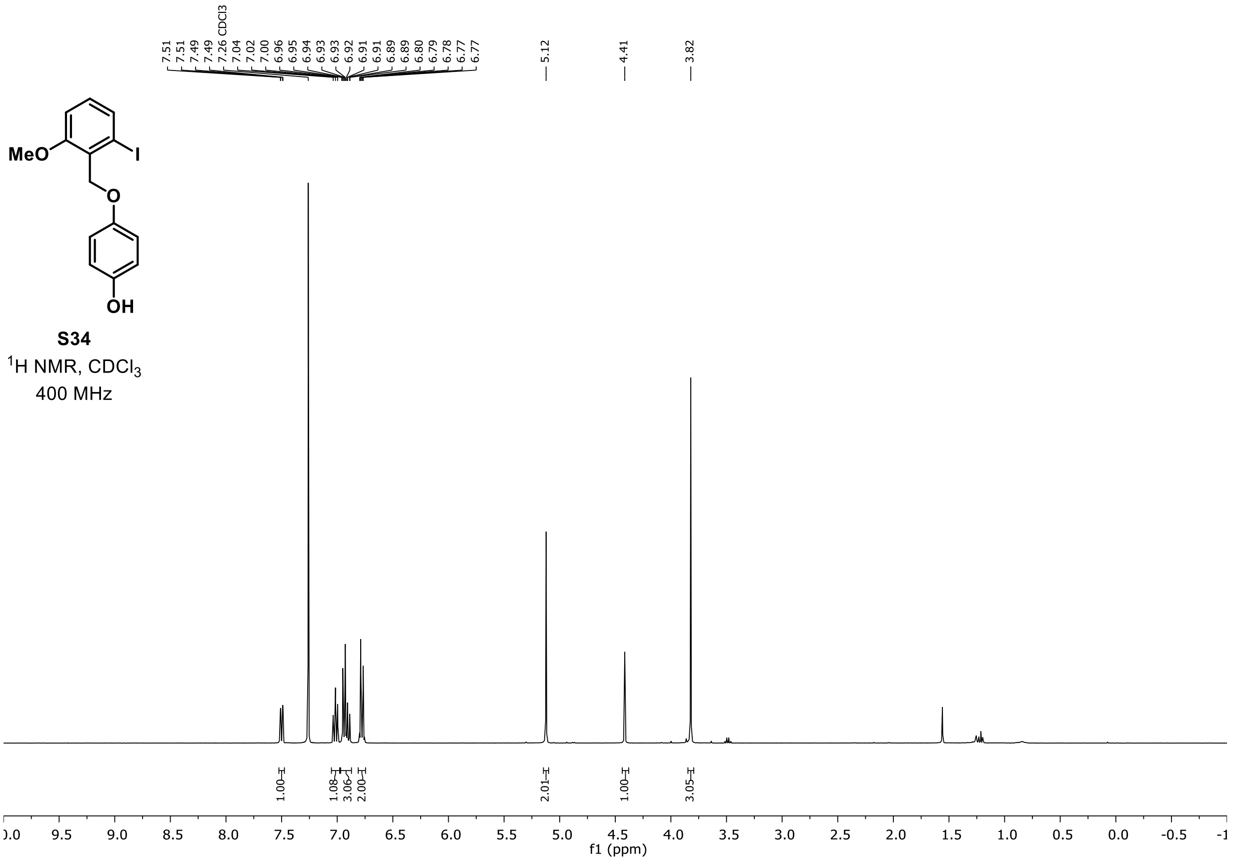

S34
^{13}C NMR, $CDCl_3$
101 MHz

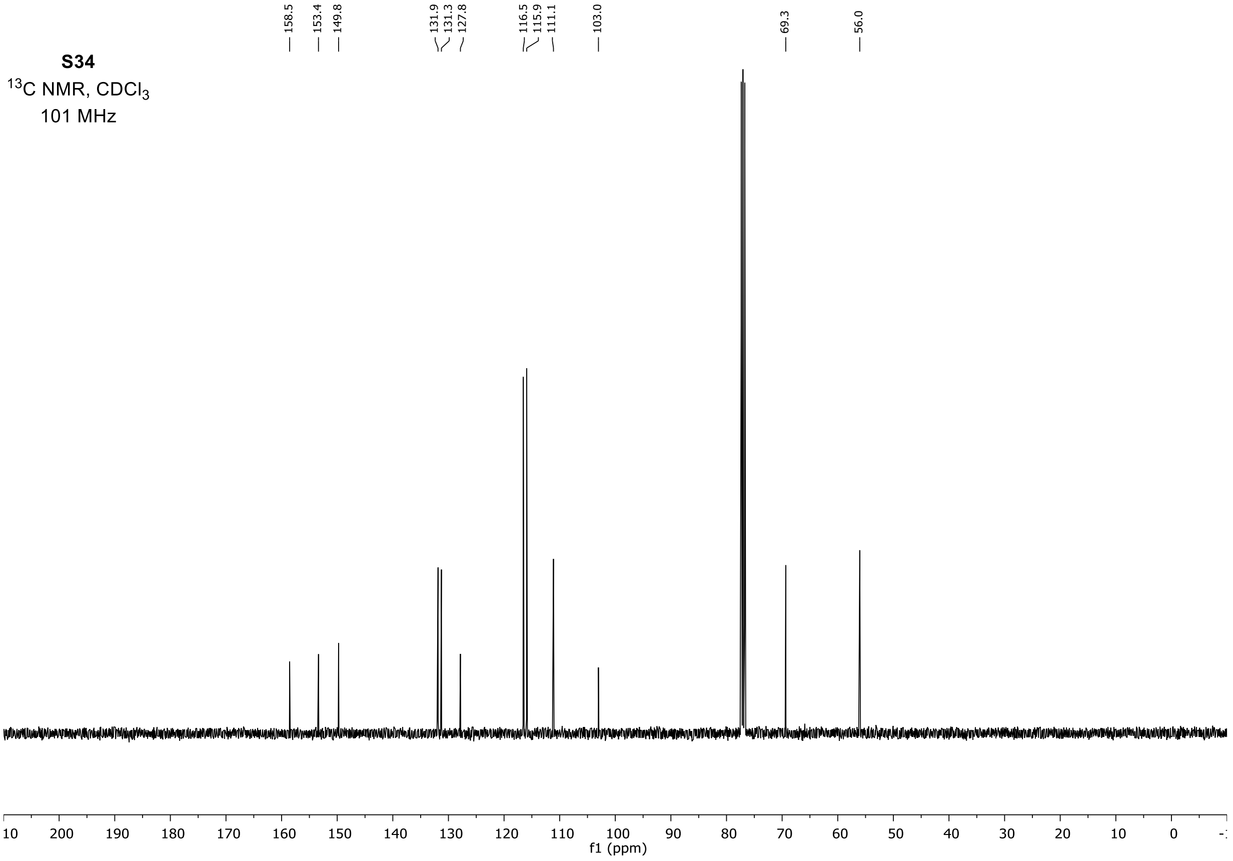

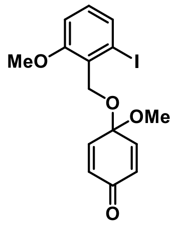

S35
^{1}H NMR, $CDCl_3$
400 MHz

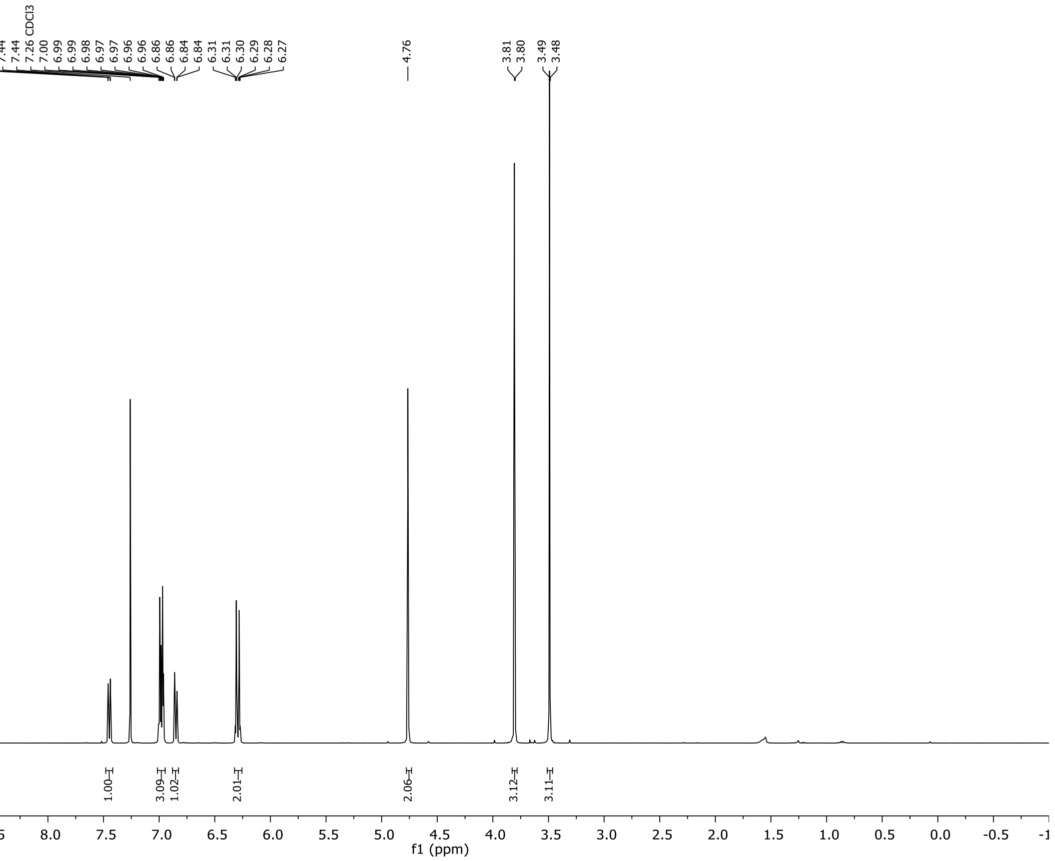

S35
^{13}C NMR, $CDCl_3$
101 MHz

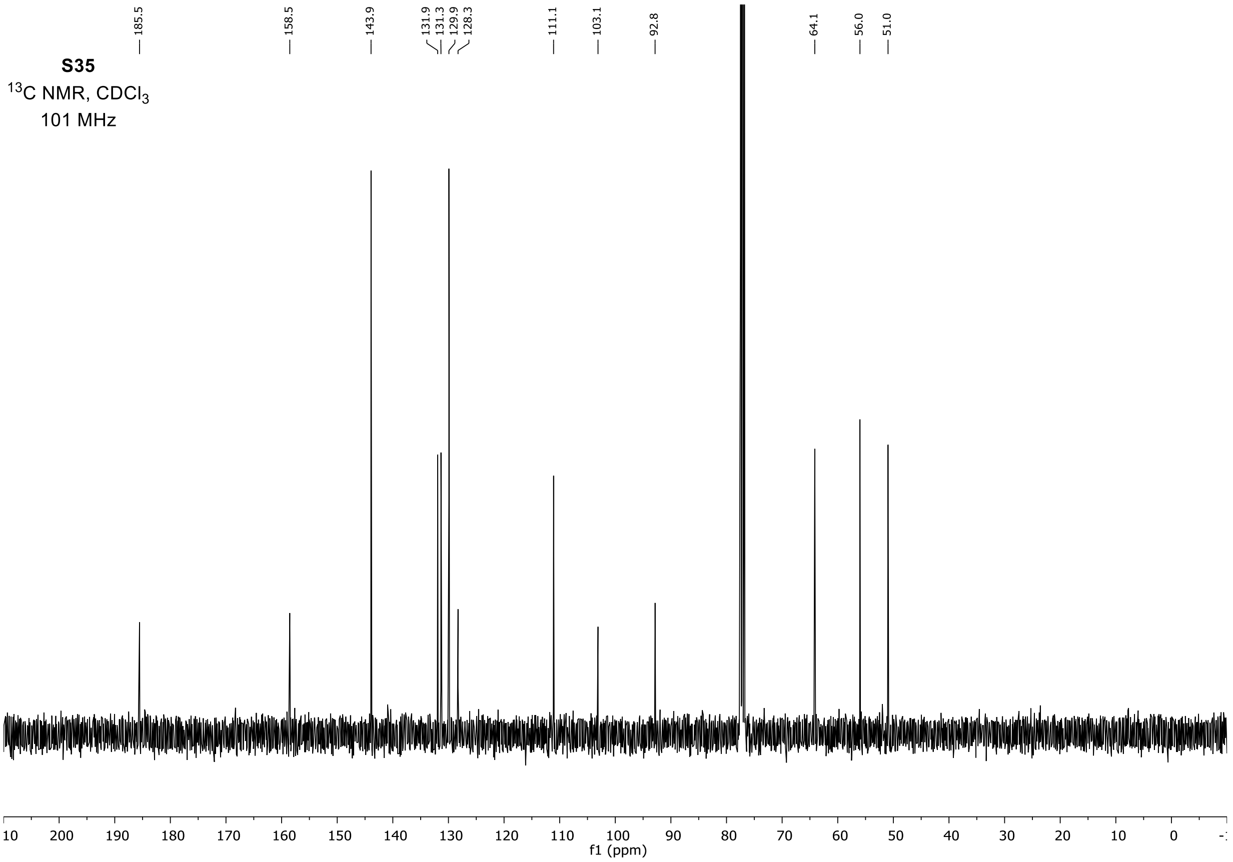

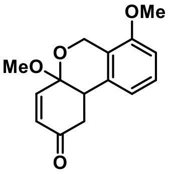

S36

^{1}H NMR, $CDCl_3$

400 MHz

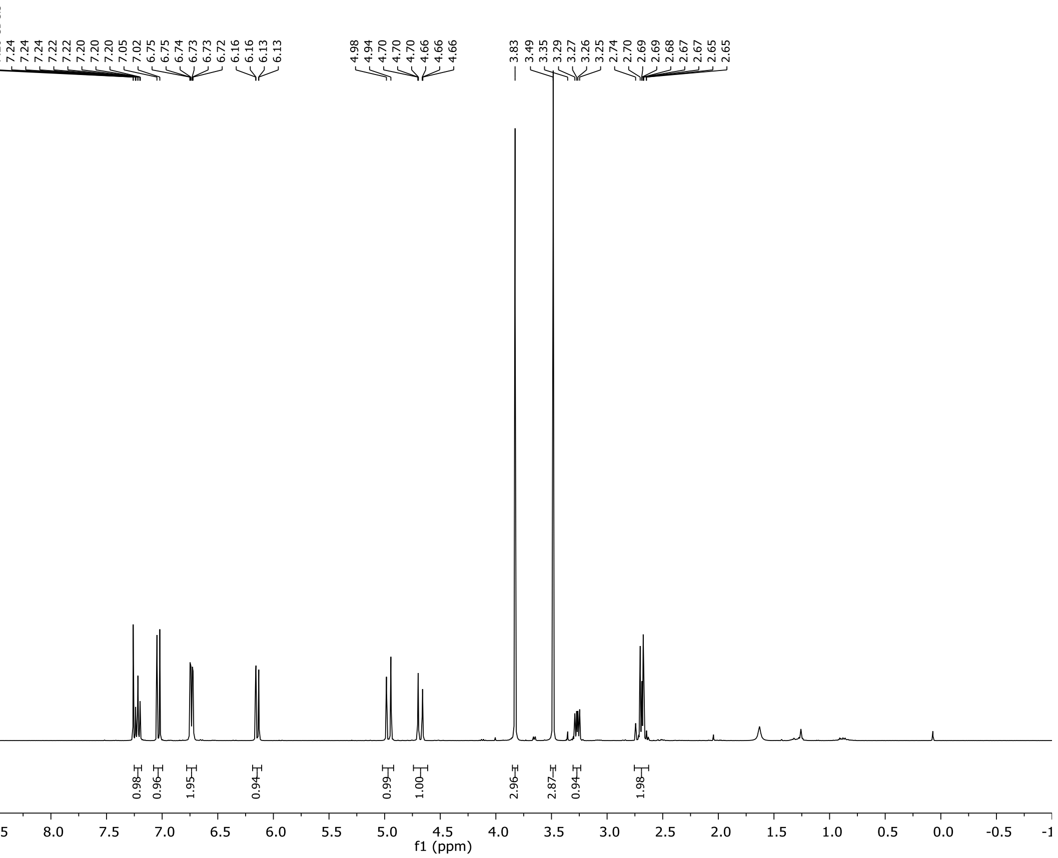

S36

^{13}C NMR, $CDCl_3$

101 MHz

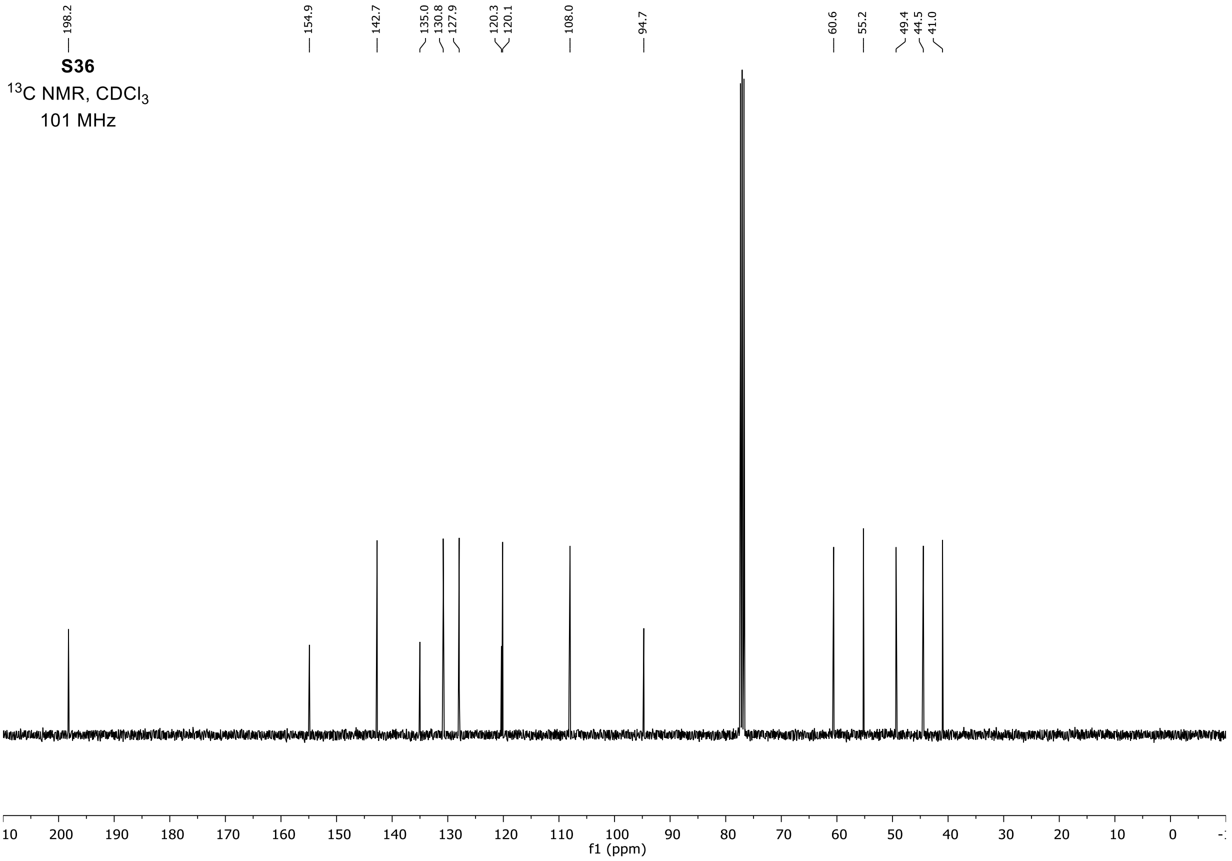

Supplement: Supplementary file 1 — ja4c08291_si_001.pdf [file ja4c08291_si_001.pdf]
